# Photochemical Construction of Trifluoromethyl Bicyclo[1.1.1]pentyl-heterocycles

**DOI:** 10.1021/acs.orglett.5c04624

**Published:** 2025-12-23

**Authors:** Marta Gil-Ordóñez, Albert Gallego-Gamo, Yingmin Ji, Tapas Maity, Remy Lalisse, Elies Molins, Roser Pleixats, Carolina Gimbert-Suriñach, Adelina Vallribera, Osvaldo Gutierrez, Albert Granados

**Affiliations:** ‡ Department of Chemistry and Centro de Innovación en Química Avanzada (ORFEO−CINQA), 16719 Universitat Autònoma de Barcelona, Cerdanyola del Vallès, 08193 Barcelona, Spain; § Department of Chemistry and Biochemistry, University of California, Los Angeles, Los Angeles, California 90095, United States; ∥ 54449 Institut de Ciència de Materials de Barcelona (ICMAB)−Consejo Superior de Investigaciones Científicas (CSIC), Campus UAB, 08193 Bellaterra, Spain

## Abstract

We describe a transition-metal-free photocatalytic synthesis of CF_3_–BCP–oxindoles via radical cascade annulation. This mild and sustainable protocol employs a bench-stable thianthrenium CF_3_–BCP reagent and activated anilides under visible-light irradiation to efficiently assemble complex scaffolds bearing dual (CF_3_ and BCP) bioisosteric features. Mechanistic studies reveal that CF_3_–BCP radical generation proceeds through a previously unreported electron donor–acceptor (EDA) complex between 4CzIPN and the thianthrenium salt, initiating a radical chain process. This method provides a practical photochemical platform for the late-stage incorporation of CF_3_–BCP motifs into oxindole frameworks and expands the accessible bioisosteric chemical space for drug discovery.

## Graphical abstract

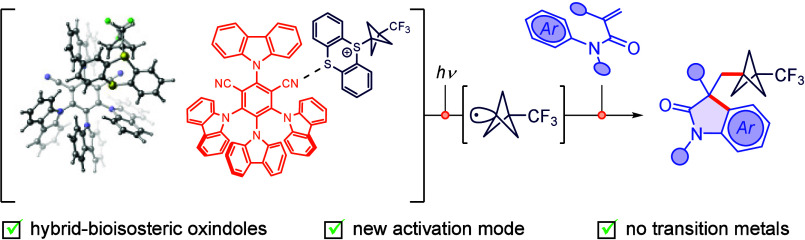

Within medicinal chemistry settings, the 100% C­(sp^3^)-hybridized and rigid bicyclo[1.1.1]­pentane (BCP) scaffold has gained recognition as a competent bioisostere for monosubstituted phenyl and 1,4-disubstituted phenyl motifs in numerous cases, attracting increasing attention.^1^ Particularly, 1,3-disubstituted BCPs can mimic the spatial orientation of *para*-substituted phenyl rings, albeit with a slightly shortened distance between the two substituents (Scheme 1A).^2^ This arises from the high fraction of sp^3^ carbons, which confers enhanced metabolic stability due to the absence of π bonds that are prone to oxidative metabolism. Additionally, the three-dimensional structure of BCPs reduces the likelihood of π–π stacking interactions observed with phenyl rings, resulting in improved solubility in physiochemical environments.^3^ Pioneering works by Pellicciari^4^ and Pfizer^5^ highlighted the unprecedented bioisosteric properties of BCP units. Since then, interest in incorporating BCP scaffolds into small-molecule drugs has skyrocketed, alongside significant advances in synthetic methods for accessing a variety of BCP-containing molecules,^6^ including photoinduced methods.^7^ The trifluoromethyl (CF_3_) group is likewise a privileged bioisostere widely used to modulate lipophilicity, metabolic stability, and binding affinity and is routinely incorporated during fluorine scan stages of drug discovery.^8,9^ Combining BCP and CF_3_ units offers the potential to generate new chemotypes with enhanced pharmacological profiles. However, general methods for assembling such dual bioisosteres remain limited.^10^

**Scheme 1:**
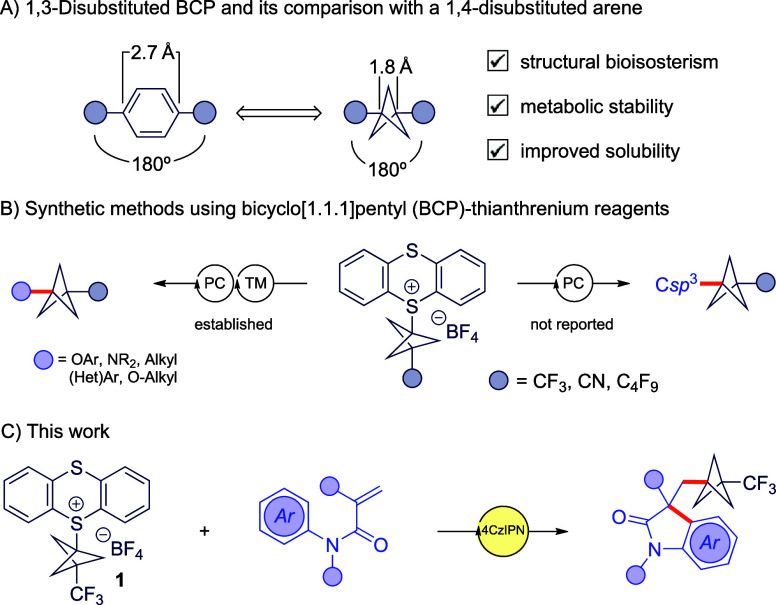
(A) Comparison of *para*-Substituted Arenes and 1,3-Disubstituted BCPs, (B) Photochemical Methods Using Bicyclo[1.1.1]­pentyl-thianthrenium Reagents, and (C) Our Work^a^

a PC, photocatalyst; TM, transition metal.

Herein, we report a photocatalytic strategy for accessing CF_3_–BCP-containing oxindoles (Scheme 1C). Oxindoles are prevalent in pharmaceuticals and natural products,^11^ yet integration of the BCP motif into this framework has not been explored.^12^ The transformation proceeds through a photochemical radical cascade between activated anilides and bench-stable thianthrenium CF_3_–BCP reagent **1**. Although **1** has been applied in metallaphotoredox cross-couplings to construct C–O, C–N, and C–C bonds (Scheme 1B),^13^ its addition to π systems has not been demonstrated. This method therefore expands both the synthetic space of oxindole scaffolds and the reactivity profile of the CF_3_–BCP radical under metal-free conditions.

We began our study using thianthrenium salt **1** and *N*-arylacrylamide **2a** as model substrates (Table S1). Preliminary experiments indicated that a photocatalyst was required to enable productive reactivity. Systematic variation of the photocatalyst, base, and reaction parameters (see Table S1) identified 4CzIPN and NaHCO_3_ as optimal, delivering oxindole **3** in up to 76% yield after 4 h of irradiation, and control studies confirmed that light is essential for the transformation. The molecular structure of oxindole **3** was unambiguously established by single-crystal X-ray diffraction, definitively confirming the presence of the CF_3_–BCP motif tethered in the oxindole core. The folded conformation of the molecule is favored by an intramolecular H···π interaction among BCP and the five-membered ring (see section 5 of the Supporting Information). To the best of our knowledge, this represents the first example of reagent **1** being employed exclusively with a photocatalyst, without the participation of other metals, such as nickel or copper, in an efficient fashion.

With the optimized conditions in hand, we evaluated the substrate scope (Table 1). A variety of *N*-alkyl-*N*-arylacrylamides bearing *para* substituents of differing electronic character afforded the annulated products in consistently good yields (**4**–**11**), indicating that arene electronics exert minimal influence on radical cyclization. The reaction was also readily scalable to 2 mmol without a loss of efficiency (see Figure S2). In contrast, *ortho* substitution led to diminished yields (**12** and **13**), consistent with steric hindrance, and an acetal-protected arene remained compatible, delivering **14** in 31% yield. *meta* substitution resulted in the formation of a complex, non-separable mixture of isomeric products. Substitution at the alkene and nitrogen positions was likewise tolerated. Acrylamides bearing α-CF_3_ (**15**) or α-benzyl (**16**) groups provided the corresponding oxindoles in 77 and 64% yields, respectively, and other *N*-alkyl groups furnished products **17** and **18** in moderate yields. Notably, β-phenyl acrylamide underwent annulation to give a six-membered product (**19**). Furthermore, tricyclic CF_3_–BCP scaffolds were accessed under the same conditions in good yields (**20**–**22**). Finally, the extension to other radical acceptors demonstrated the generality of the transformation. An imide substrate afforded product **23** in 66% yield, while hydrazone derivatives underwent cyclization to furnish pyrazolones **24**–**26** in 37–43% yields, providing access to diverse heterocyclic architectures.

**Table 1:**
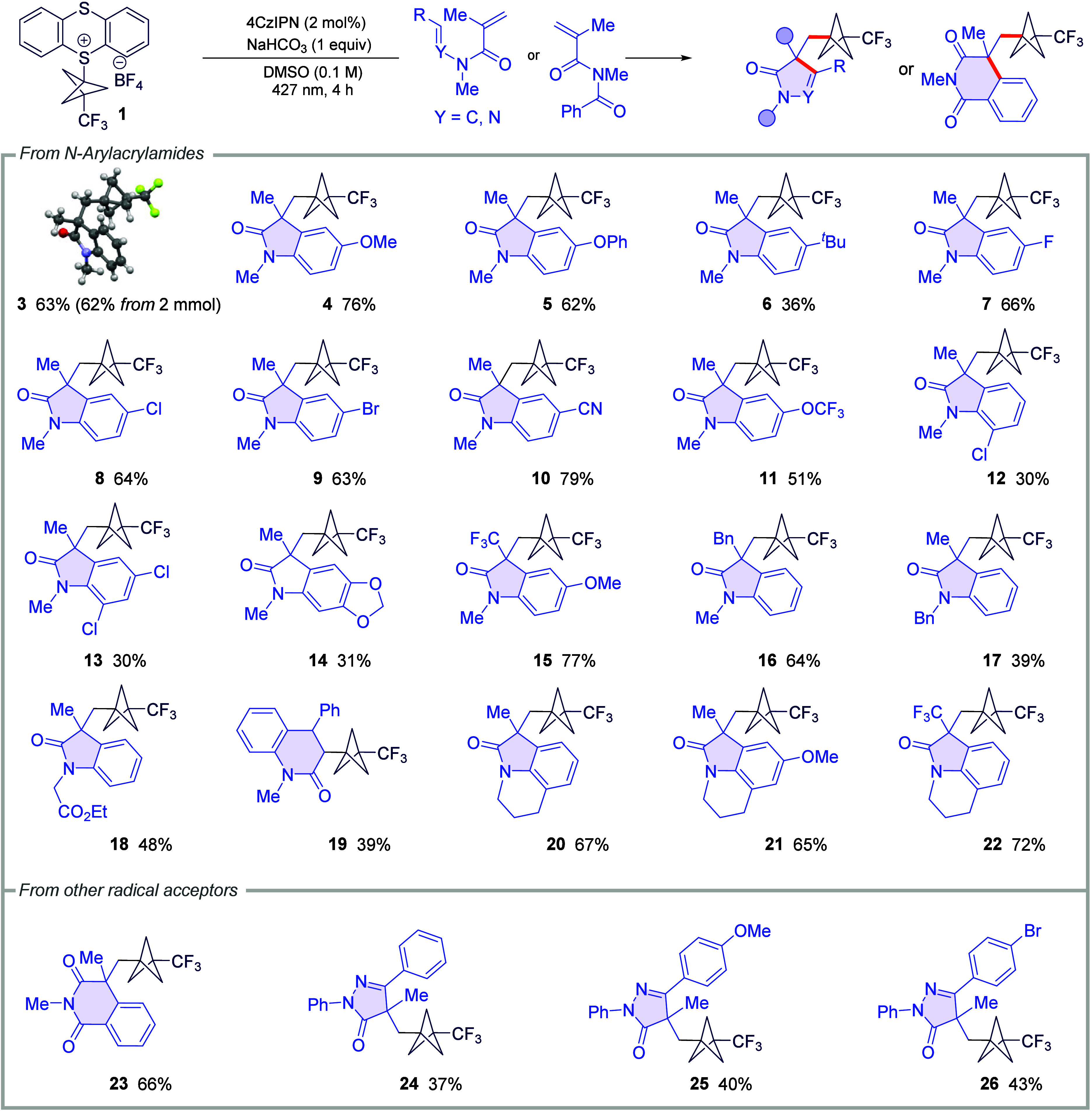
Substrate Scope Study^a^

a Reaction conditions: **1** (0.25 mmol, 1 equiv), **2a** (2 equiv), NaHCO_3_ (1 equiv), and 4CzIPN (2 mol %) in 2.5 mL of DMSO (0.1 M) under violet Kessil lamp irradiation (λ_max_ = 427 nm) at rt for 4 h.

We next investigated the reaction mechanism. The addition of TEMPO completely suppressed the formation of **3**, and the TEMPO–BCP–CF_3_ adduct was detected by HRMS, confirming radical involvement (see section 6.1 of the Supporting Information). To probe the origin of the radical, we examined the activation mode of thianthrenium salt **1**. The reduction potentials of the photocatalysts used (Figure 1A) are insufficient to reduce **1** (*E*_red_ = −1.89 V vs Fc^+^/Fc), indicating that direct single-electron transfer (SET) from the excited photocatalyst is unlikely. Notably, the comparable efficiencies of 4CzIPN and 5CzBN, despite a 380 mV difference in the reduction potential, further support this conclusion. Next, Stern–Volmer analysis showed efficient quenching of 4CzIPN by **1** (*K*_SV_ = 64.3 M^–1^), whereas no interaction was observed between 4CzIPN and acrylamide **2a** (Figure 1B). UV–Vis studies revealed that **1** absorbs at 312 nm, blue shifted relative to the 427 nm irradiation source (Figure 1C), excluding direct photoexcitation. Moreover, neither spectral overlap nor triplet energy considerations support the energy transfer. The triplet energy of **1** (*E*_T_ = 75.1 kcal mol^–1^)^12a^ significantly exceeds those of the photocatalysts. Instead, UV–Vis spectra of mixtures of **1** and 4CzIPN (Figure 1D) showed a bathochromic shift consistent with the ground-state association. These data support the formation of an electron donor–acceptor (EDA) complex^14^ between **1** and 4CzIPN, which mediates radical generation. To our knowledge, such EDA activation of a thianthrenium reagent by an organophotocatalyst has not previously been reported.^14a^ Finally, a quantum yield Φ of 4.7 was determined, which is greater than 1 and consistent with a reaction pathway proceeding via a radical chain mechanism (*vide infra*).^15^

**Figure 1:**
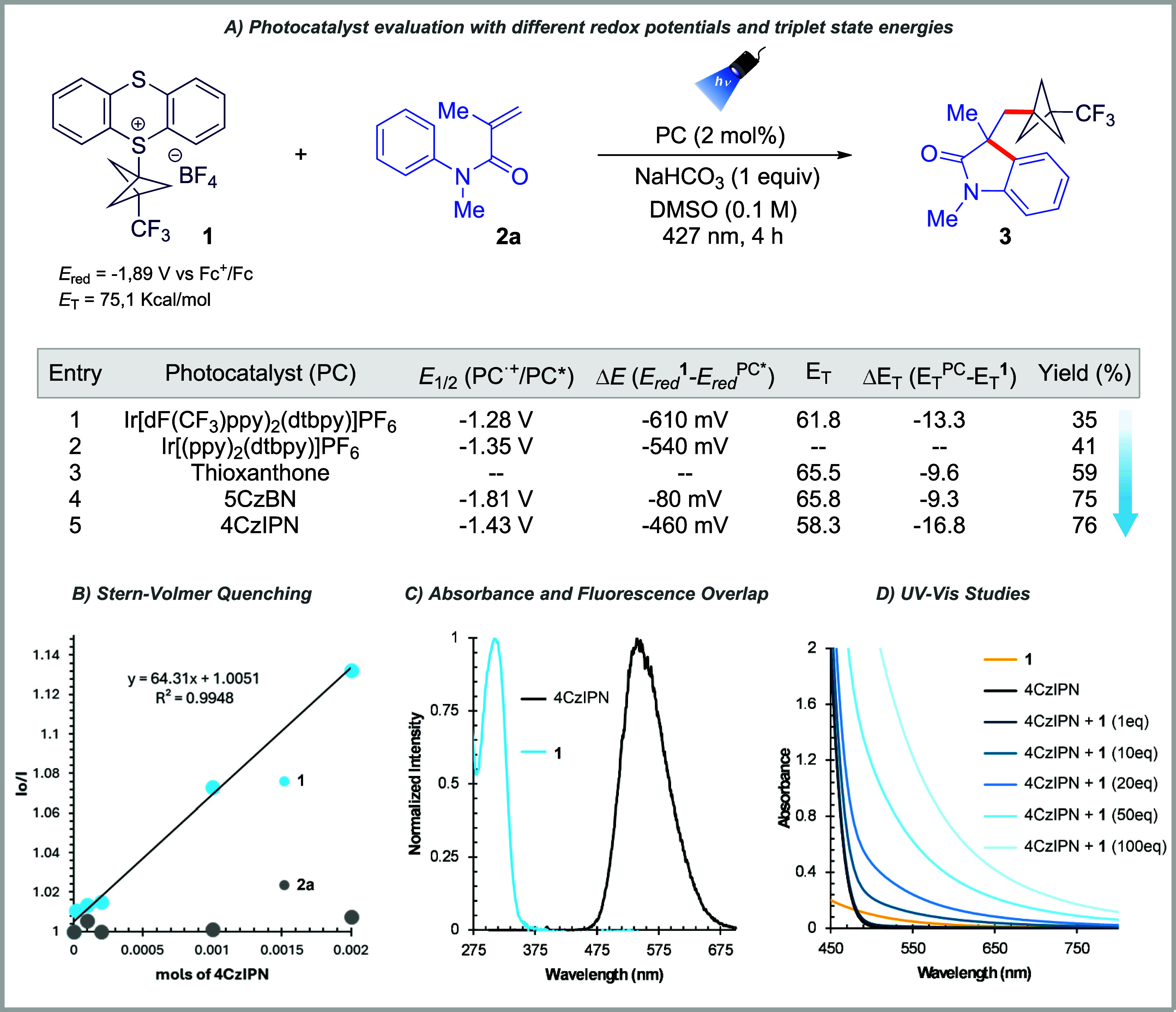
(A) Redox potentials^17^ (vs Fc^+^/Fc), triplet-state energies^18^ (*E*_T_ in kcal/mol), and reaction yields for the series of photocatalysts (PCs), (B) fluorescence quenching experiment using 4CzIPN (2 mM in DMSO), (C) normalized UV–vis of **1** in DMSO (blue line) and fluorescence spectra of 4CzIPN in DMSO (black line), and (D) UV–vis studies performed using 4CzIPN (2 mM in DMSO).

To further evaluate the mechanism for this transformation, we turned to dispersion-corrected DFT studies. Initially, we considered potential ground-state complexation (i.e., to form EDA complexes) between the 4CzIPN species (^**1**^ **PC**) and **1**. In total, we found the formation of five unique EDA complexes energetically favorable by ∼30 kcal/mol (Figure S11), supporting a charge transfer complex as the plausible activation pathway. Subsequent studies revealed that, after photoexcitation of 4CzIPN (^**1**^ **PC**), Dexter energy transfer^16^ to **1** is not feasible due to a substantial energy gap of approximately 22 kcal/mol (Figure S12), consistent with the experimental findings. The electron density difference map (EDDM) obtained after the vertical excitation of the ground-state EDA complex revealed an electron depletion in the blue region (corresponding to the CF_3_–BCP fragment) and an accumulation in the red region (corresponding to the 4CzIPN fragment), indicating a charge-transfer process within the complex (Figure 2 and Figure S13), which is in agreement with the experimental UV–vis experiment. This charge redistribution facilitates the generation of the BCP–CF_3_ radical (**rad** ^•^) and a 4CzIPN–thianthrenium intermediate, with Mulliken spin density localized on sulfur, supporting assignment of the radical cation to the thianthrenium fragment. Next, **rad** ^•^ leads to regioselective and irreversible Giese-type radical addition to substrate **2a** via **TS1**, with a barrier of 7.9 kcal/mol to form **Int1** ^•^ (downhill by 31.5 kcal/mol). In turn, **Int1** ^•^ can undergo SET to form a carbocation or undergo radical cyclization. We found that radical cyclization (via **TS2**) proceeds via a modest barrier of 14.9 kcal/mol to generate cyclized **Int2** ^•^. On the other hand, the alternative pathway leading to **Int1** ^+^ was found to be higher in energy (∼10 kcal/mol) than **TS2** and likely not operable. In turn, **Int2** ^•^ can then undergo SET with **1** to form **rad** ^•^, thianthrene (TT), and **Int3** ^+^, thereby enabling the radical chain and accounting for the experimentally observed Φ value. Finally, deprotonation of **Int3** ^+^ will lead to the desired product (downhill by 68.7 kcal/mol) and, at the same time, led to **rad** ^•^ to re-enter the chain cycle. We found that a SET with another equivalent of the thianthrenium-based CF_3_–BCP reagent (**1**) produced TT, and **rad** ^•^ was only uphill by 2.7 kcal/mol with respect to **Int2** ^•^.

**Figure 2:**
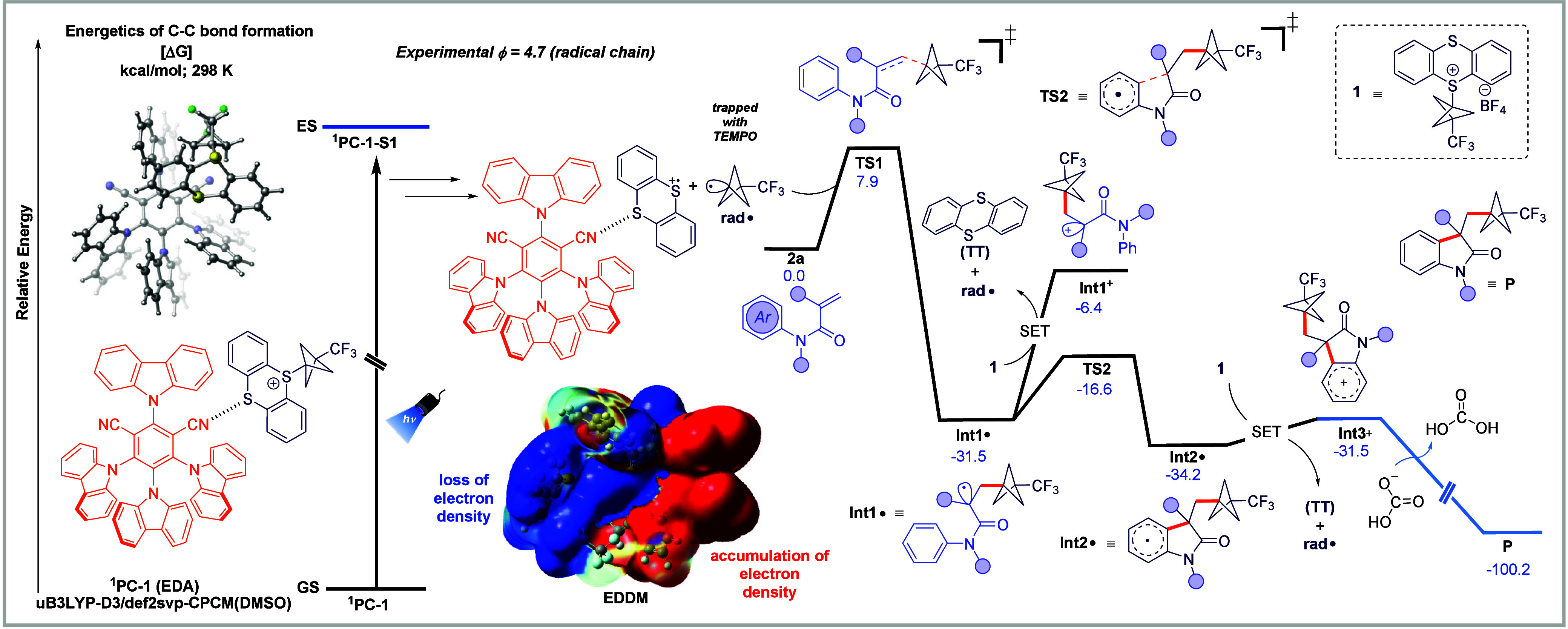
Potential energy surface of the radical chain pathway after radical activation from PC–**1** calculated at the UB3LYP/def2svp-CPCM­(DMSO) level of theory. Energies are given in blue in kcal/mol.

In summary, we have developed a novel, transition-metal-free photochemical strategy for the synthesis of hybrid CF_3_–BCP–oxindole scaffolds via radical cascade annulation. This method expands the chemical space of bioisosteric oxindoles by introducing the highly sought-after CF_3_–BCP motif into a privileged heterocyclic framework using a sustainable and operationally simple protocol. The broad substrate scope, including the formation of complex tricyclic frameworks and compatibility with diverse radical acceptors, highlights the robustness and versatility of the transformation. Mechanistic studies revealed that the CF_3_–BCP radical is generated through an unprecedented EDA complex between 4CzIPN and the thianthrenium salt, leading to a radical chain process. Experimental mechanistic studies have been found to be consistent with the DFT analysis. This approach represents a powerful platform for the late-stage introduction of two distinct and strategically valuable bioisosteres, bicyclo[1.1.1]­pentane and trifluoromethyl groups, into bioactive scaffolds.

## Supplementary Material



## Data Availability

The data underlying this study are available in the published article and its Supporting Information.

## Notes

Experimental section, including general experimental information, experimental procedures, characterization data, NMR, and DFT (PDF)
