# Supplementary material for: Photochemical Construction of Trifluoromethyl Bicyclo[1.1.1]pentyl-heterocycles
Source: Org Lett. 2025 Dec 23;28(1):248–53. doi: 10.1021/acs.orglett.5c04624 (PMC12797329; doi:10.1021/acs.orglett.5c04624)
Supplement: Supplementary file 1 [file ol5c04624_si_001.pdf]

Supporting Information

Photochemical Construction of Trifluoromethyl Bicyclo[1.1.1]pentyl-heterocycles

Marta Gil-Ordóñez,[a] Albert Gallego-Gamo,[‡,a] Yingmin Ji,[‡,a] Tapas Maity,[b] Remy Lalisse,[b] Elies Molins,[c] Roser Pleixats,[a] Carolina Gimbert-Suriñach,[a,*] Adelina Vallribera,[a,*] Osvaldo Gutierrez[b,*] and Albert Granados[a,*]

[a]*Department of Chemistry and Centro de Innovación en Química Avanzada (ORFEO-CINQA), Universitat Autònoma de Barcelona, Cerdanyola del Vallès, 08193 Barcelona, Spain*

[b]*Department of Chemistry and Biochemistry, University of California, California, Los Angeles 90095, United States*

[c]*Institut de Ciència de Materials de Barcelona (ICMAB-CSIC), Campus UAB, 08193 Bellaterra, Spain*

[‡]These authors contributed equally

*To whom correspondence should be addressed. E-mail:

carolina.gimbert@uab.es adelina.vallribera@uab.es

o.gutierrez@ucla.edu albert.granados@uab.es

Table of Content

1. General considerations S3

2. List of Used *N*-Alkyl-*N*-Arylacrylamides and *N*-Methacrylaldehyde Hydrazones S4

3. Synthesis of Bicyclo[1.1.1]pentyl-heterocycles: Reaction Workflow, Optimization and Characterization ... S5

   3.1. Reaction Workflow S5

   3.2. Reaction Optimization S6

   3.3. General Procedure for the Photoinduced Synthesis of Heterocycles (**3**–**26**) S8

   3.4. Characterization Data S8

4. Gram-Scale Synthesis of **3** S21

5. Data of X-Ray Structure of Compound **3** S22

6. Mechanistic Investigation S33

   6.1. TEMPO experiment S33

   6.2. Stern-Volmer Luminiscense Quenching Studies S34

   6.3. Cyclic Voltammetry Experiments and Redox Data S36

   6.4. Photochemical quantum yield S37

7. Computational Details S41

8. NMR Data S85

1. General considerations

1.1 General

All chemical transformations requiring inert atmosphere were done using Schlenk line techniques. For violet light irradiation, a Kessil PR160-violet LED lamp (30 W High Luminous DEX 2100 LED, λ_{max} = 427 nm) was placed 4 cm away from the reaction vials. Photoinduced reactions were performed using 4 or 8 mL Chemglass vials (15–425 Green Open Top Cap, TFE Septa). Reactions were monitored by TLC or NMR. TLC analysis was performed using hexanes/EtOAc mixtures as the eluent unless specified and visualized using UV light, Vanillin and PMA solution. The cyclic voltammetry (CV) experiments were performed with a BioLogic SP-50 Single Channel Potentiostat in a one-compartment three-electrode setup using a glassy carbon disk as the working electrode (ø = 3 mm), platinum wire as the auxiliary electrode, and SCE or $AgNO_3$/Ag (0.01 M $AgNO_3$, 0.1 M $[^nBu_4N]PF_6$ ($TBAPF_6$), MeCN) as reference electrodes. CV were performed at room temperature using the appropriate solvent, degassing with argon for 60 s and using $TBAPF_6$ as supporting electrolyte (0.1 M). All the experiments were referred to ferrocene as an internal standard. Polishing of the working electrode has been done using an alumina polishing pad with a solution of 0.05 µm alumina in water (purchased from BAS INC.). NMR experiments (^{1}H, ^{13}C, ^{19}F) were performed in the Servei de Ressonància Magnètica Nuclear, UAB, using NEO 300, NEO 400, NEO 500, or NEO 600 spectrometers. Chemical shifts are referenced to residual, nondeuterated $CHCl_3$ (δ 7.26 in ^{1}H NMR and 77.16 in ^{13}C NMR). The HRMS (ESI+) and elemental analyses were done by the Servei d'Anàlisi Química of UAB and Parque Científico Tecnológico of UBU. HRMS is determined by a Bruker microTOF-QII mass spectrometer (fly time analyzer) through positive electrospray ionization. IR spectra were recorded on an FT-IR PerkinElmer using either neat oil or solid products. Fluorescence measurements were obtained using septa-capped UV-Quartz cuvettes (10 mm path length) from Hellma Analytics and were recorded in a PerkinElmer LS 55 Fluorescence Spectrometer attached to a PTP 1 Peltier Temperature Programmer maintaining the temperature at 25 °C. Melting points (°C) are uncorrected.

1.2 Chemicals

Deuterated NMR solvents were purchased from Eurisotop. Dry solvents were obtained from Aldrich or Fisher and used as received. Bulk DCM, EtOAc and hexane were purchased from VWR. Chemicals were purchased from Fluorochem and Merck and used as received unless specified.

2. List of Used *N*-Alkyl-*N*-Arylacrylamides and *N*-Methacrylaldehyde Hydrazones

N-alkyl-*N*-arylacrylamides **2a-2u**[1] and *N*-methacrylaldehyde hydrazones **2v-2x**[2] were prepared according to modified reported methods.

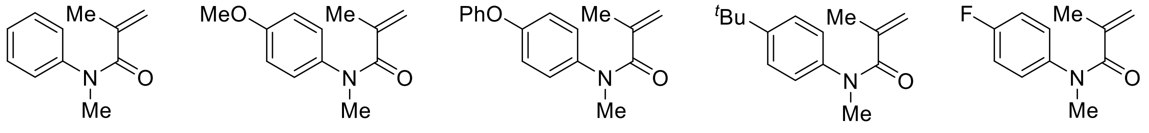

2a **2b** **2c** **2d** **2e**

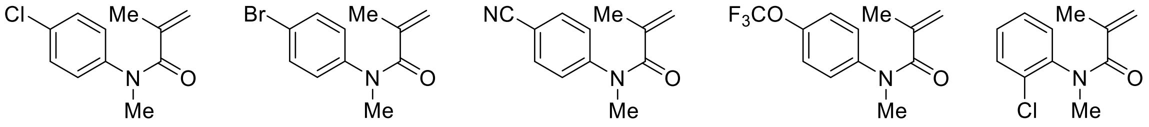

2f **2g** **2h** **2i** **2j**

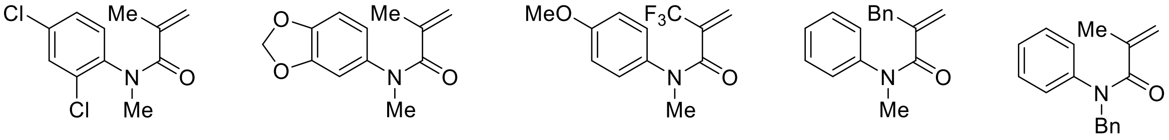

2k **2l** **2m** **2n** **2o**

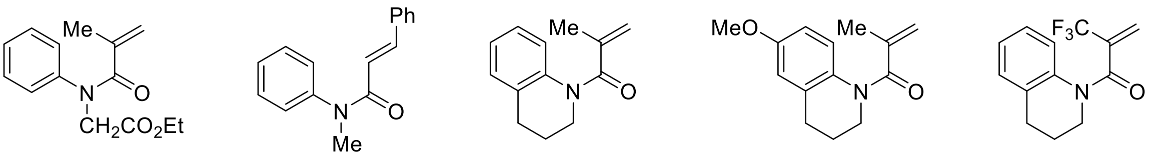

2p **2q** **2r** **2s** **2t**

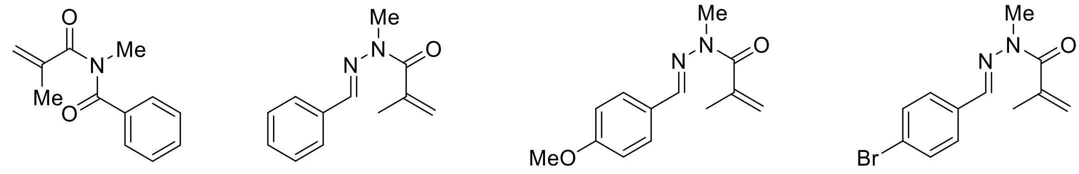

2u **2v** **2w** **2x**

[1] (a) Majhi, J.; Granados, A.; Matsuo, B.; Ciccone, V.; Dhungana, R. K.; Sharique, M.; Molander, G. A. *Chem. Sci.* **2023**, *14*, 897–902; (b) Li, X.; Zhao, H.; Meng, L.; Dong, J.; Yang, C.; Wei, Z.; Wang, X.; Xu, J.; Fan, B. *J. Org. Chem.* **2023**, *88*, 5300−5310; (c) Shaw, R.; Sihag, N.; Jain, S.; Sharma, R.; Yadav, M. R. *J. Org. Chem.* **2023**, *88*, 5652−5660.

[2] Yu, S.; Cheng, Y.; Pan, C.; Yu, J-T. *Chem. Commun.* **2025**, *61*, 1196–1199.

Compound **2s** was synthetized analogously to the other acrylamides, starting from 6-methoxy-1,2,3,4-tetrahydroquinoline.

1-(6-methoxy-3,4-dihydroquinolin-1(2H)-yl)-2-methylprop-2-en-1-one (2s)

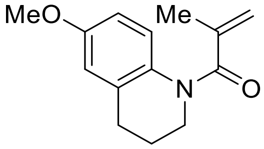

^{1}H NMR (400 MHz, $CDCl_3$), δ (ppm) = 7.18 – 7-09 (m, 1H), 6.66 (m, 2H), 5.14 (d, J = 3.8 Hz, 2H), 3.77 (m, 5H), 2.73 (t, J = 6.9 Hz, 2H), 1.96 (p, J = 6.8 Hz, 2H), 1.84 (s, 3H). **^{13}C{^{1}H} NMR** (126 MHz, $CDCl_3$), δ (ppm) = 171.3, 156.9, 141.6, 133.0, 132.2, 125.3, 118.8, 113.3, 111.6, 55.5, 44.1, 27.2, 24.1, 20.0. **HRMS** (ESI+) calcd for $C_{14}H_{18}NO_2$ $[M+H]^+$: 232.1332, found 232.1334.

3. Synthesis of Bicyclo[1.1.1]pentyl-heterocycles: Reaction Workflow, Optimization and Characterization

3.1. Reaction Workflow

All photoinduced reactions were done using a Kessil PR160-purple LED lamp (30 W High Luminous DEX 2100 LED, λmax = 427 nm). The LED was placed 4 cm away from the reaction vial within a ventilated fume hood and using a fan to maintain the temperature approximately at 25ºC.

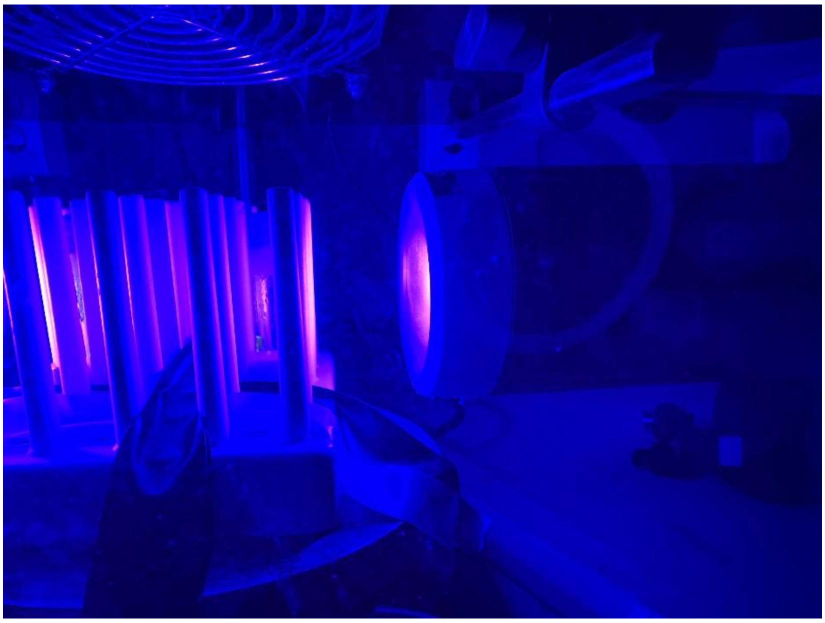

Figure S1. *Reaction setup for the photoinduced synthesis of CF_3-BCP-containing heterocycles.*

3.2. Reaction Optimization

Table S1. Exploration of the optimal reaction conditions.[a]

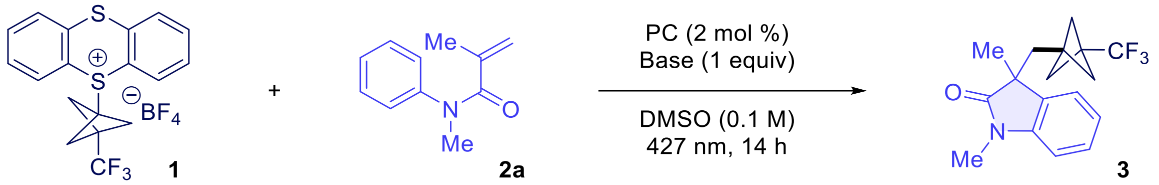

Entry	PC	Base	Yield (%)[b]
1	None	K_2CO_3	32
2	None	NEt_3	17
3	None	DIPEA	22
4	None	None	traces
5	Ir[dF(CF_3)ppy)$_2$dtbpy]PF_6	K_2CO_3	45
6	Ir[(dtbpy)(ppy)$_2$]PF_6	K_2CO_3	55
7	4CzIPN	None	60
8	4CzIPN	DABCO	58
9	4CzIPN	K_2CO_3	61
**10**	**4CzIPN**	**$NaHCO_3$**	**76**
11	5CzBN	$NaHCO_3$	75
12	Thioxanthone	$NaHCO_3$	59
13[c]	4CzIPN	$NaHCO_3$	34-55
14[d]	4CzIPN	$NaHCO_3$	0
**15[e]**	**4CzIPN**	**$NaHCO_3$**	**76**
16[f]	4CzIPN	$NaHCO_3$	26-51
17	None	DABCO	24
18[g]	None	NPh_3	29

[a]Reaction conditions: **1** (0.1 mmol, 1 equiv), **2a** (0.2 mmol, 2 equiv), PC (2 mol %) and base (0.1 mmol, 1 equiv) in 1 mL of DMSO (0.1 M) under violet Kessil lamp irradiation (λ_{max} = 427 nm) at rt for 14 h. [b]Yields were determined by ^{1}H NMR analysis using 1,3,5-trimethoxybenzene as internal standard. [c]DMA, DMF, MeCN or DCM. [d]No light. [e]Ilumination for 4h. [f]456 or 525 nm Kessil lamp. [g]0.5 equiv of base.

We initiated the investigation into the synthesis of these dual bioisosteric oxindoles using readily available thianthrenium salt **1** and *N*-arylacrylamide **2a** in Table S1 as model substrates. Initial experiments hypothesized that potassium carbonate could activate the thianthrenium salt via electron donor-acceptor (EDA) complex photoactivation. The desired product **3** was obtained in 32% yield in DMSO (Table S1, entry 1). Other aminated

electron-donors used in EDA-sulfonium chemistry were used without better success (Table S1, entries 17-18). To enhance this transformation, we employed photocatalysts to facilitate the single-electron transfer process. The use of iridium-based photocatalysts (Table S1, entries 2–3) significantly improved the yield of oxindole **3**. Further optimization with the organophotocatalyst 4CzIPN in Table 1 in the absence of base (entry 4) afforded additional enhancement. Subsequent base screening identified $NaHCO_3$ as optimal (Table S1, entry 7, and other bases such as DABCO or K_2CO_3 likely interfered with photoactivation by competing with the potential electron donor–acceptor complex, leading to diminished yields (Table S1, entries 5 and 6). The organophotocatalyst 5CzBN also delivered good efficiency, whereas thioxanthone reduced product formation (Table 1, entries 8 and 9). Remarkably, the reaction was completed after only 4 hours of illumination affording an optimal yield of 76% (Table 1, entry 10). Finally, control experiments demonstrated that light irradiation is essential, confirming the photochemical nature of the transformation (Table S1, entry 14) and other light sources resulted in less efficacy (Table S1, entry 16).

*3.3. General Procedure for the Photoinduced Synthesis of Heterocycles (**3–26**)*

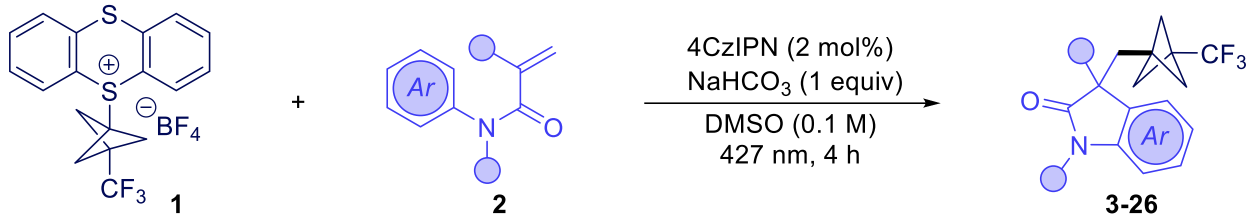

To a 4 mL Chemglass vial equipped with a magnetic stirring bar, the corresponding acrylamide **2** (0.50 mmol, 2.0 equiv), 4CzIPN (4 mg, 0.02 equiv), $NaHCO_3$ (21 mg, 0.25 mmol, 1.0 equiv) and BCP-thianthrenium salt **1** (110 mg, 0.25 mmol, 1.0 equiv) were added. Then, 2.5 mL of dry DMSO were added under inert atmosphere and the reaction was degassed with Argon for 20 seconds. The reaction mixture was irradiated for 4 h with a 427 nm Kessil PR160-purple LED as described in the "Workflow" section. The temperature of the reaction was maintained at approximately 25°C via a fan. Upon completion, the reaction mixture was diluted with EtOAc (10 mL) and washed with brine (3 × 10 mL). The organic layer was dried over anhydrous Na_2SO_4, filtered and concentrated under reduced pressure. The crude mixture was subjected to flash column chromatography purification using hexanes/EtOAc mixtures or dichloromethane to yield the desired compound.

3.4. Characterization Data

1,3-Dimethyl-3-((3-(trifluoromethyl)bicyclo[1.1.1]pentan-1-yl)methyl)indolin-2-one (3)

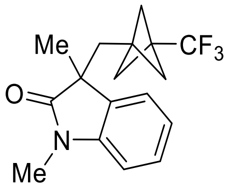

Prepared according to the *General Procedure* from the corresponding acrylamide **2a** (88 mg, 0.5 mmol, 2.0 equiv). After purification by flash column chromatography (hexane:EtOAc 9:1), the title compound **3** was obtained as a white solid (48 mg, 0.15 mmol, 63%). **R_f** = 0.30 (silica gel, *n*-hexane / EtOAc, 6:1 (v/v)); **mp**: 101 - 103ºC. **^{1}H NMR** (600 MHz, $CDCl_3$), δ (ppm) = 7.32 (td, *J* = 7.6, 1.3 Hz, 1H), 7.17 (d, *J* = 6.1 Hz, 1H), 7.09 (t, *J* = 7.4, 1.0 Hz, 1H), 6.89 (d, *J* = 7.7 Hz, 1H), 3.25 (s, 3H), 2.32 (d, *J* = 14.6 Hz, 1H), 2.05 (d, *J* = 14.6 Hz, 1H), 1.44 (dd, *J* = 9.6, 2.3 Hz, 3H), 1.35-1.33 (m, 6H). **^{13}C{^{1}H} NMR** (126 MHz, $CDCl_3$), δ (ppm) = 179.9, 143.1, 133.3, 128.2, 122.7, 122.6, 122.3 (q, *J* = 275.7 Hz), 108.1, 49.5 (q, *J* = 2.0 Hz), 47.1, 39.0, 37.1 (q, *J* = 2.6 Hz), 36.8 (q, *J* = 37.7 Hz), 26.3, 25.5. **^{19}F{^{1}H} NMR** (376 MHz, $CDCl_3$), δ (ppm) = -73.4. **FT-IR** (cm^{-1}, neat, ATR), ṽ = 3060, 2981, 1705, 1613, 1494, 1470, 1391, 1376, 1337, 1253, 1171. **HRMS** (ESI+) calcd for $C_{17}H_{19}F_3NO$ $[M+H]^+$: 310.1413, found 310.1422.

5-Methoxy-1,3-dimethyl-3-((3-(trifluoromethyl)bicyclo[1.1.1]pentan-1-yl)methyl)indolin-2-one (4)

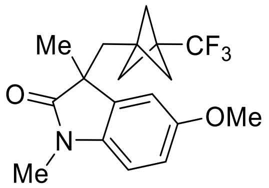

Prepared according to the *General Procedure* from the corresponding acrylamide **2b** (103 mg, 0.5 mmol, 2.0 equiv). After purification by flash column chromatography (hexane:EtOAc 10:1), the title compound **4** was obtained as a white solid (64 mg, 0.19 mmol, 76%). $\mathbf{R_f}$ = 0.35 (silica gel, *n*-hexane / EtOAc, 6:1 (v/v)); **mp**: 120 - 122ºC. **^{1}H NMR** (600 MHz, $CDCl_3$), δ (ppm) = 6.83 (dd, *J* = 8.6, 2.3 Hz, 1H), 6.80-6.77 (m, 2H), 3.82 (s, 3H), 3.22 (s, 3H), 2.31 (d, *J* = 14.4 Hz, 1H), 2.00 (d, *J* = 14.5 Hz, 1H), 1.47 (d, *J* = 9.5, 2.2 Hz, 3H), 1.37 (dd, *J* = 9.4, 2.1 Hz, 3H), 1.32 (s, 3H). **^{13}C{^{1}H} NMR** (151 MHz, $CDCl_3$), δ (ppm) = 179.6, 156.1, 136.7, 134.7, 122.3 (q, *J* = 275.9 Hz), 112.0, 110.6, 110.5, 108.3, 55.84 (q, *J* = 31.5 Hz), 49.5, 47.5, 39.2 – 38.8 (m), 36.8 (q, *J* = 37.5 Hz), 26.3 (q, *J* = 19.8 Hz), 25.5 (q, *J* = 19.1 Hz). **^{19}F{^{1}H} NMR** (376 MHz, $CDCl_3$), δ (ppm) = -73.3. **FT-IR** (cm^{-1}, neat, ATR), ṽ = 2977, 2880, 1704, 1596, 1491, 1458, 1389, 1376, 1297, 1209, 1121. **HRMS** (ESI+) calcd for $C_{18}H_{21}F_3NO_2$ $[M+H]^+$: 340.1519, found 340.1525.

1,3-Dimethyl-5-phenoxy-3-((3-(trifluoromethyl)bicyclo[1.1.1]pentan-1-yl)methyl)indolin-2-one (5)

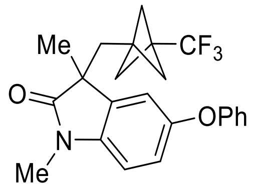

Prepared according to the *General Procedure* from the corresponding acrylamide **2c** (134 mg, 0.5 mmol, 2.0 equiv). After purification by flash column chromatography (hexane:EtOAc 8:1), the title compound **5** was obtained as an oil (62 mg, 0.15 mmol, 62%). $\mathbf{R_f}$ = 0.40 (silica gel, *n*-hexane / EtOAc, 6:1 (v/v)). **^{1}H NMR** (400 MHz, $CDCl_3$), δ (ppm) = 7.29 (dd, *J* = 8.7, 7.4 Hz, 2H), 7.04 (ddd, *J* = 7.4, 6.2, 1.2 Hz, 1H), 6.96 (dd, *J* = 8.4, 2.5 Hz, 1H), 6.90 (dd, *J* = 8.8, 1.1 Hz, 2H), 6.86 (d, *J* = 2.4 Hz, 1H), 6.79 (d, *J* = 8.4 Hz, 1H), 3.20 (s, 3H), 2.25 (d, *J* = 14.5 Hz, 1H), 1.93 (d, *J* = 14.5 Hz, 1H), 1.44 (dd, *J* = 9.9, 1.6 Hz, 3H), 1.35 (dd, *J* = 9.5, 2.0 Hz, 3H), 1.27 (s, 3H). **^{13}C{^{1}H} NMR** (126 MHz, $CDCl_3$), δ (ppm) = 179.7, 158.3, 152.6, 139.1, 135.1, 129.8, 122.8, 122.4 (q, *J* = 276.2 Hz), 119.3, 117.7, 115.3, 108.8, 49.6 (q, *J* = 2.0 Hz), 47.6, 38.9, 37.2, 36.9 (q, *J* = 38.0 Hz), 26.4, 25.5. **^{19}F{^{1}H} NMR** (376 MHz, $CDCl_3$), δ (ppm) = -73.3. **FT-IR** (cm^{-1}, neat, ATR), ṽ = 2981, 1709, 1590, 1487, 1439, 1389, 1260, 1221, 1190. **HRMS** (ESI+) calcd for $C_{23}H_{23}F_3NO_2$ $[M+H]^+$: 402.1675, found 402.1688.

5-(Tert-butyl)-1,3-dimethyl-3-((3-(trifluoromethyl)bicyclo[1.1.1]pentan-1-yl)methyl)indolin-2-one (6)

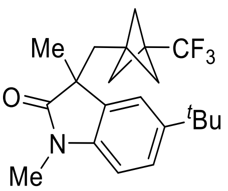

Prepared according to the *General Procedure* from the corresponding acrylamide **2d** (116 mg, 0.5 mmol, 2.0 equiv). After purification by flash column chromatography (hexane:EtOAc 10:1), the title compound **6** was obtained as a white solid (32 mg, 0.09 mmol, 36%). $\mathbf{R_f}$ = 0.55 (silica gel, *n*-hexane / EtOAc, 6:1 (v/v)); **mp**: 83 - 85ºC. ^{1}**H NMR** (400 MHz, $CDCl_3$), δ (ppm) = 7.27 (dd, *J* = 8.2, 2.0 Hz, 1H), 7.14 (d, *J* = 2.0 Hz, 1H), 6.75 (d, *J* = 8.2 Hz, 1H), 3.17 (s, 3H), 2.25 (d, *J* = 14.4 Hz, 1H), 1.98 (d, *J* = 14.6 Hz, 1H), 1.36 (d, *J* = 9.6 Hz, 3H), 1.28-1.24 (m, 15H). ^{13}**C{^{1}H} NMR** (126 MHz, $CDCl_3$), δ (ppm) = 180.1, 145.9, 140.7, 132.8, 124.4, 122.3 (q, *J* = 275.9 Hz), 120.0, 107.5, 49.4 (q, *J* = 2.2 Hz), 47.3, 39.1, 37.2 (q, *J* = 1.7 Hz), 36.8 (q, *J* = 37.9 Hz), 34.6, 31.6, 26.3, 25.5. ^{19}**F{^{1}H} NMR** (282 MHz, $CDCl_3$), δ (ppm) = -73.43. **FT-IR** (cm^{-1}, neat, ATR), ṽ = 2960, 2921, 1701, 1500, 1463, 1377, 1258, 1188. **HRMS** (ESI+) calcd for $C_{21}H_{27}F_3NO$ $[M+H]^+$: 366.2039, found 366.2047.

5-Fluoro-1,3-dimethyl-3-((3-(trifluoromethyl)bicyclo[1.1.1]pentan-1-yl)methyl)indolin-2-one (7)

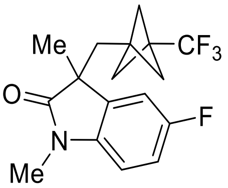

Prepared according to the *General Procedure* from the corresponding acrylamide **2e** (90 mg, 0.5 mmol, 2.0 equiv). After purification by flash column chromatography (hexane:EtOAc 10:1), the title compound **7** was obtained as a white solid (54 mg, 0.17 mmol, 66%). $\mathbf{R_f}$ = 0.38 (silica gel, *n*-hexane / EtOAc, 6:1 (v/v)); **mp**: 87 - 89ºC. ^{1}**H NMR** (600 MHz, $CDCl_3$), δ (ppm) = 7.03 (td, *J* = 8.8, 2.6 Hz, 1H), 6.93 (dd, *J* = 7.8, 2.6 Hz, 1H), 6.81 (dd, *J* = 8.5, 4.0 Hz, 1H), 3.24 (s, 3H), 2.33 (d, *J* = 14.5 Hz, 1H), 2.02 (d, *J* = 14.5 Hz, 1H), 1.48 (d, *J* = 9.4 Hz, 3H), 1.38 (d, *J* = 11.7 Hz, 3H), 1.34 (s, 3H). ^{13}**C{^{1}H} NMR** (126 MHz, $CDCl_3$), δ (ppm) = 179.6, 159.4 (d, *J* = 241.2 Hz), 139.1, 135.0 (d, *J* = 7.9 Hz), 122.3 (q, *J* = 275.7 Hz), 114.3 (d, *J* = 23.4 Hz), 110.8 (d, *J* = 24.8 Hz), 108.7 (d, *J* = 8.3 Hz), 49.5 (q, *J* = 2.3 Hz), 47.6, 39.0, 37.1-37.0 (m), 36.9 (q, *J* = 38.1 Hz), 26.4, 25.4. ^{19}**F{^{1}H} NMR** (376 MHz, $CDCl_3$), δ (ppm) = -73.4, -120.5. **FT-IR** (cm^{-1}, neat, ATR), ṽ = 2979, 2914, 1712, 1608, 1494, 1466, 1392, 1377, 1188. **HRMS** (ESI+) calcd for $C_{17}H_{18}F_4NO$ $[M+H]^+$: 328.1319, found 328.1329.

5-Chloro-1,3-dimethyl-3-((3-(trifluoromethyl)bicyclo[1.1.1]pentan-1-yl)methyl)indolin-2-one (8)

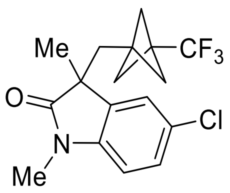

Prepared according to the *General Procedure* from the corresponding acrylamide **2f** (98 mg, 0.5 mmol, 2.0 equiv). After purification by flash column chromatography (hexane:EtOAc 7:1), the title compound **8** was obtained as a white solid (55 mg, 0.16 mmol, 64%). **R$_f$** = 0.50 (silica gel, *n*-hexane / EtOAc, 6:1 (v/v)); **mp**: 108 - 110ºC. **^{1}H NMR** (500 MHz, $CDCl_3$), δ (ppm) = 7.31 (dd, *J* = 8.2, 2.1 Hz, 1H), 7.16 (d, *J* = 2.3 Hz, 1H), 6.82 (d, *J* = 8.3 Hz, 1H), 3.24 (s, 3H), 2.33 (d, *J* = 14.4 Hz, 1H), 2.03 (d, *J* = 14.5 Hz, 1H), 1.49 (d, *J* = 9.5 Hz, 3H), 1.39 (d, *J* = 9.5 Hz, 3H), 1.34 (s, 3H). **^{13}C{^{1}H} NMR** (126 MHz, $CDCl_3$), δ (ppm) = 179.4, 141.8, 135.1, 128.1, 128.0, 123.2, 122.2 (q, *J* = 275.8 Hz), 109.1, 49.5 (q, *J* = 1.9 Hz), 47.4, 39.0, 37.1-37.0 (m), 36.9 (q, *J* = 38.1 Hz), 26.4, 25.4. **^{19}F{^{1}H} NMR** (376 MHz, $CDCl_3$), δ (ppm) = -73.4. **FT-IR** (cm^{-1}, neat, ATR), ṽ = 3057, 2924, 1714, 1607, 1490, 1425, 1391, 1376, 1332, 1270, 1241, 1190. **HRMS** (ESI+) calcd for $C_{17}H_{18}ClF_3NO$ $[M+H]^+$: 344.1024, found 344.1034.

5-Bromo-1,3-dimethyl-3-((3-(trifluoromethyl)bicyclo[1.1.1]pentan-1-yl)methyl)indolin-2-one (9)

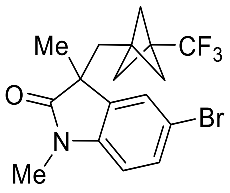

Prepared according to the *General Procedure* from the corresponding acrylamide **2g** (127 mg, 0.5 mmol, 2.0 equiv). After purification by flash column chromatography (hexane:EtOAc 10:1), the title compound **9** was obtained as a white solid (61 mg, 0.16 mmol, 63%). **R$_f$** = 0.55 (silica gel, *n*-hexane / EtOAc, 6:1 (v/v)); **mp**: 97 - 99ºC. **^{1}H NMR** (500 MHz, $CDCl_3$), δ (ppm) = 7.45 (dd, *J* = 8.2, 1.8 Hz, 1H), 7.29 (m, 1H), 6.77 (d, *J* = 8.2 Hz, 1H), 3.23 (s, 3H), 2.32 (d, *J* = 14.5 Hz, 1H), 2.03 (d, *J* = 14.5 Hz, 1H), 1.48 (d, *J* = 9.5 Hz, 3H), 1.38 (d, *J* = 9.5 Hz, 3H), 1.34 (s, 3H). **^{13}C{^{1}H} NMR** (151 MHz, $CDCl_3$), δ (ppm) = 179.3, 142.2, 135.4, 131.1, 126.1, 122.2 (q, *J* = 276.2 Hz), 115.3, 109.7, 49.5 (q, *J* = 2.5 Hz), 47.4, 39.0, 37.1-37.0 (m), 36.9 (q, *J* = 38.1 Hz), 29.7, 26.3, 25.3. **^{19}F{^{1}H} NMR** (282 MHz, $CDCl_3$), δ (ppm) = -73.4. **FT-IR** (cm^{-1}, neat, ATR), ṽ = 2978, 1715, 1604, 1487, 1393, 1331, 1190. **HRMS** (ESI+) calcd for $C_{17}H_{18}BrF_3NO$ $[M+H]^+$: 388.0518, found 388.0525.

1,3-Dimethyl-2-oxo-3-((3-(trifluoromethyl)bicyclo[1.1.1]pentan-1-yl)methyl)indoline-5-carbonitrile (10)

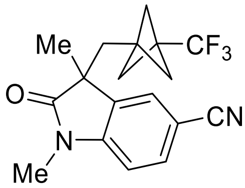

Prepared according to the *General Procedure* from the corresponding acrylamide **2h** (93 mg, 0.5 mmol, 2.0 equiv). After purification by flash column chromatography (hexane:EtOAc 10:1), the title compound **10** was obtained as a white solid (66 mg, 0.20 mmol, 79%). $\mathbf{R_f}$ = 0.30 (silica gel, *n*-hexane / EtOAc, 6:1 (v/v)); **mp**: 122 - 124ºC. **^{1}H NMR** (600 MHz, $CDCl_3$), δ (ppm) = 7.68 (dd, *J* = 8.1, 1.7 Hz, 1H), 7.44 (d, *J* = 1.7 Hz, 1H), 6.97 (d, *J* = 8.1 Hz, 1H), 3.29 (s, 3H), 2.36 (d, *J* = 14.7 Hz, 1H), 2.08 (d, *J* = 14.7 Hz, 1H), 1.47 (dd, *J* = 9.5, 2.3 Hz, 3H), 1.37 (m, 6H). **^{13}C{^{1}H} NMR** (126 MHz, $CDCl_3$), δ (ppm) = 179.5, 147.1, 134.5, 133.7, 126.0, 122.1 (q, *J* = 275.7 Hz), 119.0, 108.6, 105.8, 49.5 (q, *J* = 2.3 Hz), 47.0, 38.9, 37.0 (q, *J* = 38.2 Hz), 36.9 (q, *J* = 1.8 Hz), 26.5, 25.3. **^{19}F{^{1}H} NMR** (282 MHz, $CDCl_3$), δ (ppm) = -73.4. **FT-IR** (cm^{-1}, neat, ATR), ṽ = 2989, 2219, 1710, 1614, 1494, 1391, 1346, 1258, 1190. **HRMS** (ESI+) calcd for $C_{18}H_{18}F_3N_2O$ $[M+H]^+$: 335.1366, found 335.1371.

1,3-dimethyl-5-(trifluoromethoxy)-3-((3-(trifluoromethyl)bicyclo[1.1.1]pentan-1-yl)methyl)indolin-2-one (11)

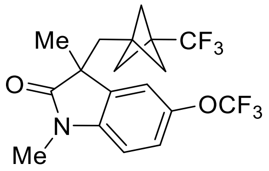

Prepared according to the *General Procedure* from the corresponding acrylamide **2i** (129.6 mg, 0.5 mmol, 2.0 equiv). After purification by flash column chromatography (hexane:EtOAc 10:1), the title compound **11** was obtained as an oil (47.8 mg, 0.19 mmol, 51%). $\mathbf{R_f}$ = 0.43 (silica gel, *n*-hexane / EtOAc, 6:1 (v/v)). **^{1}H NMR** (300 MHz, $CDCl_3$), δ (ppm) = 7.24 – 7.20 (m, 1H), 7.07 (dd, *J* = 2.4, 1.1 Hz, 1H), 6.88 (d, *J* = 8.4 Hz, 1H), 3.24 (s, 3H), 2.33 (d, *J* = 14.6 Hz, 1H), 2.03 (d, *J* = 14.6 Hz, 1H), 1.47 (dd, *J* = 9.6, 2.0 Hz, 3H), 1.38 – 1.34 (m, 6H). **^{13}C{^{1}H} NMR** (126 MHz, $CDCl_3$), δ (ppm) = 179.6, 144.8, 141.8, 134.9, 122.2 (q, *J* = 273.8 Hz), 121.4, 120.6 (q, *J* = 255.0 Hz), 116.8, 108.6, 49.5 – 49.4 (m), 47.5, 39.0, 37.6 – 36.3 (m), 26.5, 25.3. **^{19}F{^{1}H} NMR** (282 MHz, $CDCl_3$), δ (ppm) = -58.4, -73.4. **FT-IR** (cm^{-1}, neat, ATR), ṽ = 2985, 1715, 1620, 1494, 1391, 1350, 1250, 1216, 1161, 1119. **HRMS** (ESI+) calcd for $C_{18}H_{18}F_6NO_2$ $[M+H]^+$: 394.1236, found 394.1243.

7-Chloro-1,3-dimethyl-3-((3-(trifluoromethyl)bicyclo[1.1.1]pentan-1-yl)methyl)indolin-2-one (12)

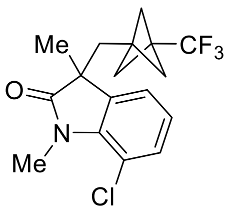

Prepared according to the *General Procedure* from the corresponding acrylamide **2j** (105 mg, 0.5 mmol, 2.0 equiv). After purification by flash column chromatography (hexane:EtOAc 10:1), the title compound **12** was obtained as an oil (25 mg, 0.07 mmol, 30%). $\mathbf{R_f}$ = 0.63 (silica gel, *n*-hexane / EtOAc, 6:1 (v/v)). ^{1}**H NMR** (300 MHz, $CDCl_3$), δ (ppm) = 7.25 (dd, *J* = 7.9, 1.5 Hz, 1H), 7.09 – 6.95 (m, 2H), 3.63 (s, 3H), 2.33 (d, *J* = 14.5 Hz, 1H), 2.03 (d, *J* = 14.3 Hz, 1H), 1.49 (dd, *J* = 9.6, 2.2 Hz, 3H), 1.40 (dd, *J* = 9.6, 2.0 Hz, 3H), 1.33 (s, 3H). ^{13}**C{^{1}H} NMR** (126 MHz, $CDCl_3$), δ (ppm) =180.2, 139.1, 136.1, 130.5, 123.3, 122.3 (q, *J* = 275.7 Hz), 121.1, 115.7, 49.5 (q, *J* = 2.3 Hz), 47.0, 39.2, 37.0 (q, *J* = 1.9 Hz), 36.9 (q, *J* = 38.1 Hz), 29.6, 25.9. ^{19}**F{^{1}H} NMR** (376 MHz, $CDCl_3$), δ (ppm) = -73.7. **FT-IR** (cm^{-1}, neat, ATR), ṽ = 2962, 1721, 1607, 1465, 1392, 1259, 1191. **HRMS** (ESI+) calcd for $C_{17}H_{18}ClF_3NO$ $[M+H]^+$: 344.1024, found 344.1029.

4,6-dichloro-1,3-dimethyl-3-((3-(trifluoromethyl)bicyclo[1.1.1]pentan-1-yl)methyl)indolin-2-one (13)

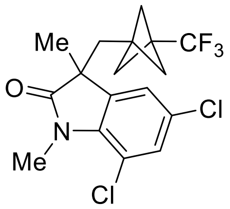

Prepared according to the *General Procedure* from the corresponding acrylamide **2k** (122.1 mg, 0.5 mmol, 2.0 equiv). After purification by flash column chromatography (hexane:EtOAc 8:1), the title compound **13** was obtained as a solid (28.3 mg, 0.07 mmol, 30%). $\mathbf{R_f}$ = 0.50 (silica gel, *n*-hexane / EtOAc, 6:1 (v/v)). ^{1}**H NMR** (400 MHz, $CDCl_3$), δ (ppm) = 7.02 (d, *J* = 1.7 Hz, 1H), 6.79 (d, *J* = 1.7 Hz, 1H), 3.21 (s, 3H), 2.58 (d, *J* = 14.6 Hz, 1H), 2.20 (d, *J* = 14.6 Hz, 1H), 1.50 (dd, *J* = 9.5, 2.0 Hz, 3H), 1.43 – 1.38 (m, 6H). ^{13}**C{^{1}H} NMR** (126 MHz, $CDCl_3$), δ (ppm) =179.4, 145.7, 134.8, 131.3, 127.7, 123.1, 122.3 (q, *J* = 275.9 Hz), 107.6, 49.3 (q, *J* = 2.3 Hz), 48.6, 37.2, 36.8 (q, *J* = 39.2 Hz), 36.0, 26.0, 22.5. ^{19}**F{^{1}H} NMR** (376 MHz, $CDCl_3$), δ (ppm) = -73.7. **FT-IR** (cm^{-1}, neat, ATR), ṽ = 3062, 2985, 1716, 1602, 1576, 1293, 1191. **HRMS** (ESI+) calcd for $C_{17}H_{17}Cl_2F_3NO$ $[M+H]^+$: 378.0634, found 378.0634.

5,7-Dimethyl-7-((3-(trifluoromethyl)bicyclo[1.1.1]pentan-1-yl)methyl)-5,7-dihydro-6H-[1,3]dioxolo[4,5-f]indolin-6-one (14)

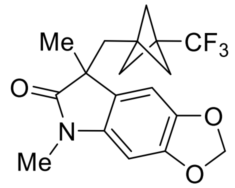

Prepared according to the *General Procedure* from the corresponding acrylamide **2l** (103 mg, 0.5 mmol, 2.0 equiv). After purification by flash column chromatography (hexane:EtOAc 8:1), the title compound **14** was obtained as a white solid (27 mg, 0.08 mmol, 31%). **R$_f$** = 0.30 (silica gel, *n*-hexane / EtOAc, 6:1 (v/v)); **mp**: 138 - 140ºC. **^{1}H NMR** (600 MHz, $CDCl_3$), δ (ppm) = 6.76 (d, *J* = 7.9 Hz, 1H), 6.31 (d, *J* = 7.9 Hz, 1H), 5.99 (d, *J* = 1.5 Hz, 1H), 5.96 (d, *J* = 1.5 Hz, 1H), 3.20 (s, 3H), 2.26 (d, *J* = 14.4 Hz, 1H), 2.21 (d, *J* = 14.5 Hz, 1H), 1.53 (dd, *J* = 9.4, 2.1 Hz, 3H), 1.44 (dd, *J* = 9.5, 2.2 Hz, 3H), 1.40 (s, 3H). **^{13}C{^{1}H} NMR** (126 MHz, $CDCl_3$), δ (ppm) = 178.9, 144.3, 143.3, 138.3, 122.4 (q, *J* = 276.2 Hz), 113.9, 106.7, 101.6, 99.9, 49.3 (q, *J* = 2.3 Hz), 46.5, 37.3 (q, *J* = 1.9 Hz), 37.2, 36.8 (q, *J* = 38.1 Hz), 26.7, 23.7 **^{19}F{^{1}H} NMR** (282 MHz, $CDCl_3$), δ (ppm) = -73.4. **FT-IR** (cm^{-1}, neat, ATR), ṽ = 2999, 2924, 1700, 1659, 1620, 1451, 1389, 1296, 1242, 1189. **HRMS** (ESI+) calcd for $C_{18}H_{19}F_3NO_3$ $[M+H]^+$: 354.1312, found 354.1323.

5-Methoxy-1-methyl-3-(trifluoromethyl)-3-((3-(trifluoromethyl)bicyclo[1.1.1]pentan-1-yl)methyl)indolin-2-one (15)

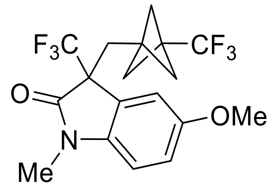

Prepared according to the *General Procedure* from the corresponding acrylamide **2m** (130 mg, 0.5 mmol, 2.0 equiv). After purification by flash column chromatography (hexane:EtOAc 10:1), the title compound **15** was obtained as a white solid (75 mg, 0.19 mmol, 77%). **R$_f$** = 0.54 (silica gel, *n*-hexane / EtOAc, 4:1 (v/v)); **mp**: 109 – 111ºC. **^{1}H NMR** (300 MHz, $CDCl_3$), δ (ppm) = 6.95 – 6.92 (m, 2H), 6.82 (d, *J* = 9.3 Hz, 1H), 3.80 (s, 3H), 3.23 (s, 3H), 2.64 (d, *J* = 14.2 Hz, 1H), 2.32 (d, *J* = 14.2 Hz, 1H), 1.50 (dd, *J* = 9.6, 1.8 Hz, 3H), 1.38 (dd, *J* = 9.5, 1.8 Hz, 3H). **^{13}C{^{1}H} NMR** (75 MHz, $CDCl_3$), δ (ppm) = 170.6, 156.3, 137.7, 126.2, 124.3 (q, *J* = 282.6 Hz), 122.1 (q, *J* = 275.5 Hz), 114.4, 112.8, 109.1, 55.9, 55.5 (q, *J* = 26.6 Hz), 49.6 (q, *J* = 2.2 Hz), 37.2 (q, *J* = 38.3 Hz), 35.7 (d, *J* = 1.8 Hz), 31.6 (d, *J* = 2.0 Hz), 26.7. **^{19}F{^{1}H} NMR** (282 MHz, $CDCl_3$), δ (ppm) = -73.3, -73.6. **FT-IR** (cm^{-1}, neat, ATR), ṽ = 1712, 1613, 1515, 1493, 1470, 1393, 1376, 1338, 1311, 1253, 1190, 1168, 1120, 1082, 1035, 1014, 906. **HRMS** (ESI+) calcd for $C_{18}H_{17}F_6NO_2Na$ $[M+Na]^+$: 416.1056, found 416.1063.

3-Benzyl-1-methyl-3-((3-(trifluoromethyl)bicyclo[1.1.1]pentan-1-yl)methyl)indolin-2-one (16)

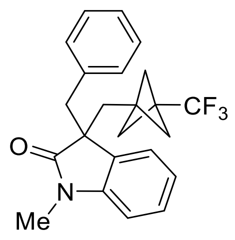

Prepared according to the *General Procedure* from the corresponding acrylamide **2n** (126 mg, 0.5 mmol, 2.0 equiv). After purification by flash column chromatography (hexane:EtOAc 10:1), the title compound **16** was obtained as a white solid (61 mg, 0.16 mmol, 64%). $\mathbf{R_f}$ = 0.36 (silica gel, *n*-hexane / EtOAc, 4:1 (v/v)); **mp**: 107–108ºC. **^{1}H NMR** (400 MHz, $CDCl_3$), δ (ppm) = 7.21 (td, *J* = 7.7, 1.7 Hz, 1H), 7.11 – 6.99 (m, 5H), 6.84 – 6.77 (m, 2H), 6.63 (d, *J* = 7.8 Hz, 1H), 3.02 (d, *J* = 12.8 Hz, 1H), 2.98 (s, 3H), 2.95 (d, *J* = 12.8 Hz, 1H), 2.42 (d, *J* = 14.4 Hz, 1H), 2.14 (d, *J* = 14.4 Hz, 1H), 1.43 (d, *J* = 9.5 Hz, 3H), 1.33 (d, *J* = 9.5 Hz, 3H). **^{13}C{^{1}H} NMR** (75 MHz, $CDCl_3$), δ (ppm) = 178.6, 143.6, 135.1, 130.4, 130.0 (2C), 128.2, 127.5 (2C), 126.6, 123.8, 122.3 (q, *J* = 275.8 Hz), 121.1, 107.9, 52.9, 49.7 (d, *J* = 2.1 Hz, 3C), 45.1, 37.5, 37.1 (q, *J* = 1.6 Hz), 37.0 (q, *J* = 37.9 Hz), 25.9. **^{19}F{^{1}H} NMR** (282 MHz, $CDCl_3$), δ (ppm) = -73.3. **FT-IR** (cm^{-1}, neat, ATR), ṽ = 1712, 1613, 1515, 1493, 1470, 1393, 1376, 1338, 1311, 1253, 1190, 1168, 1120, 1082, 1035, 1014, 906. **HRMS** (ESI+) calcd for $C_{23}H_{23}F_3NO$ $[M+H]^+$: 386.1726, found 386.1727.

1-Benzyl-3-methyl-3-((3-(trifluoromethyl)bicyclo[1.1.1]pentan-1-yl)methyl)indolin-2-one (17)

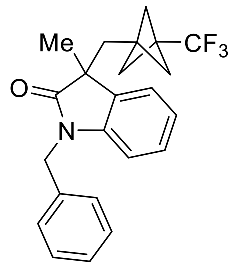

Prepared according to the *General Procedure* from the corresponding acrylamide **2o** (126 mg, 0.5 mmol, 2.0 equiv). After purification by flash column chromatography (hexane:EtOAc 10:1), the title compound **17** was obtained as a white solid (37 mg, 0.1 mmol, 39%). $\mathbf{R_f}$ = 0.65 (silica gel, *n*-hexane / EtOAc, 6:1 (v/v)); **mp**: 81 - 83ºC. **^{1}H NMR** (400 MHz, $CDCl_3$), δ (ppm) = 7.39 – 7.31 (m, 4H), 7.30 – 7.26 (m, 1H), 7.23 (td, *J* = 7.7, 1.2 Hz, 1H), 7.18 (dd, *J* = 7.3, 1.6 Hz, 1H), 7.06 (td, *J* = 7.5, 1.1 Hz, 1H), 6.87 (d, *J* = 7.8 Hz, 1H), 5.20 (d, *J* = 15.4 Hz, 1H), 4.67 (d, *J* = 15.5 Hz, 1H), 2.36 (d, *J* = 14.5 Hz, 1H), 2.09 (d, *J* = 14.5 Hz, 1H), 1.43 (dd, *J* = 9.6, 2.1 Hz, 3H), 1.40 (s, 3H), 1.34 (dd, *J* = 9.4, 2.1 Hz, 3H). **^{13}C{^{1}H} NMR** (126 MHz, $CDCl_3$), δ (ppm) = 180.0, 142.4, 135.8, 133.3, 128.8, 128.1, 127.8, 127.7, 122.8, 122.5, 122.3 (q, *J* = 275.8 Hz), 109.2, 49.6 (q, *J* = 1.8 Hz), 47.2, 43.9, 38.8, 37.2 (q, *J* = 1.8 Hz), 36.9 (q, *J* = 37.7 Hz), 26.1. **^{19}F{^{1}H} NMR** (376 MHz, $CDCl_3$), δ (ppm) = -73.4. **FT-IR** (cm^{-1}, neat, ATR), ṽ

= 3000, 2919, 1715, 1608, 1487, 1467, 1450, 1391, 1354, 1188. **HRMS** (ESI+) calcd for $C_{23}H_{23}F_3NO$ $[M+H]^+$: 386.1726, found 386.1735.

Ethyl 2-(3-methyl-2-oxo-3-((3-(trifluoromethyl)bicyclo[1.1.1]pentan-1-yl)methyl)indolin-1-yl)acetate (18)

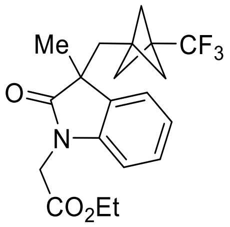

Prepared according to the *General Procedure* from the corresponding acrylamide **2p** (123.6 mg, 0.5 mmol, 2.0 equiv). After purification by flash column chromatography (hexane:EtOAc 9:1), the title compound **18** was obtained as an oil (45.7 mg, 0.12 mmol, 48%). $\mathbf{R_f}$ = 0.28 (silica gel, *n*-hexane / EtOAc, 6:1 (v/v)). **^{1}H NMR** (400 MHz, $CDCl_3$), δ (ppm) = 7.26 (td, *J* = 7.5, 1.3 Hz, 1H), 7.17 (dd, *J* = 7.5, 1.3 Hz, 1H), 7.07 (td, *J* = 7.5, 0.9 Hz, 1H), 6.75 (d, *J* = 7.8 Hz, 1H), 4.57 (d, *J* = 17.5 Hz, 1H), 4.39 (d, *J* = 17.4 Hz, 1H), 4.29 – 4.16 (m, 2H), 2.34 (d, *J* = 14.6 Hz, 1H), 2.08 (d, *J* = 14.6 Hz, 1H), 1.55 (dd, *J* = 9.5, 2.0 Hz, 3H), 1.40 (dd, *J* = 9.5, 2.0 Hz, 3H), 1.34 (s, 3H), 1.25 (t, *J* = 7.1 Hz, 3H). **^{13}C{^{1}H} NMR** (126 MHz, $CDCl_3$), δ (ppm) =179.9, 167.6, 141.8, 133.0, 128.1, 122.9, 122.8, 122.4 (q, *J* = 275.9 Hz), 108.5, 61.8, 49.5 (q, *J* = 1.8 Hz, 3C), 47.3, 41.5, 38.6, 37.1, 36.9 (q, *J* = 37.8 Hz), 26.5, 14.1. **^{19}F{^{1}H} NMR** (376 MHz, $CDCl_3$), δ (ppm) = -73.3. **FT-IR** (cm^{-1}, neat, ATR), ṽ = 2981, 1749, 1715, 1610, 1490, 1387, 1191. **HRMS** (ESI+) calcd for $C_{20}H_{23}F_3NO_3$ $[M+H]^+$: 382.1625, found 382.1624.

1-methyl-4-phenyl-3-(3-(trifluoromethyl)bicyclo[1.1.1]pentan-1-yl)-3,4-dihydroquinolin-2(1H)-one (19)

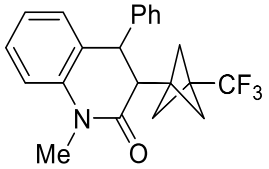

Prepared according to the *General Procedure* from the corresponding acrylamide **2q** (237 mg, 0.5 mmol, 2.0 equiv). After purification by flash column chromatography (hexane:EtOAc 9:1), the title compound **19** was obtained as a yellowish oil (35.9 mg, 0.10 mmol, 39%). $\mathbf{R_f}$ = 0.25 (silica gel, *n*-hexane / EtOAc, 9:1 (v/v)); **mp**: 106 – 108°C. **^{1}H NMR** (400 MHz, $CDCl_3$), δ (ppm) = 7.33 (td, *J* = 7.8, 1.6 Hz, 1H), 7.27 – 7.16 (m, 4H), 7.11 – 6.99 (m, 4H), 4.06 (d, *J* = 2.1 Hz, 1H), 3.38 (s, 3H), 3.13 – 3.10 (m, 1H), 1.79 (dd, *J* = 9.5, 2.0 Hz, 3H), 1.66 (dd, *J* = 9.5, 2.1 Hz, 3H). **^{13}C{^{1}H} NMR** (126 MHz, $CDCl_3$), δ (ppm) = 168.3, 141.5, 139.8, 129.6, 129.0 (2C), 128.5, 127.2, 127.1 (2C), 126.2, 125.9, 123.9, 122.6 (q, *J* = 275.7 Hz), 115.0, 49.9, 48.6 (3C), 39.8, 36.7 (q, *J* = 38.6 Hz), 29.6. **^{19}F{^{1}H} NMR** (377 MHz, $CDCl_3$), δ (ppm) = -73.1. **FT-IR** (cm^{-1}, neat, ATR), ṽ = 2918, 1711, 1666, 1602, 1461, 1362, 1167. **HRMS** (ESI+) calcd for $C_{22}H_{21}F_3NO$ $[M+H]^+$: 372.1570, found 372.1570.

1-Methyl-1-((3-(trifluoromethyl)bicyclo[1.1.1]pentan-1-yl)methyl)-5,6-dihydro-4H-pyrrolo[3,2,1-ij]quinolin-2(1H)-one (20)

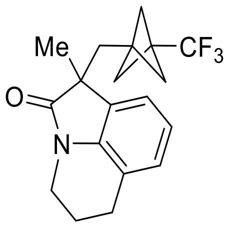

Prepared according to the *General Procedure* from the corresponding acrylamide **2r** (101 mg, 0.5 mmol, 2.0 equiv). After purification by flash column chromatography (DCM), the title compound **20** was obtained as a white solid (56 mg, 0.17 mmol, 67%). **R_f** = 0.40 (silica gel, DCM); **mp**: 97 - 99ºC. **^{1}H NMR** (500 MHz, $CDCl_3$), δ (ppm) = 7.00 (dd, *J* = 7.0, 1.3 Hz, 1H), 6.96 – 6.87 (m, 2H), 3.72 – 3.66 (m, 2H), 2.89 – 2.75 (m, 2H), 2.24 (d, *J* = 14.4 Hz, 1H), 2.08 – 1.87 (m, 3H), 1.42 (dd, *J* = 9.5, 2.2 Hz, 3H), 1.36 – 1.25 (m, 6H). **^{13}C{^{1}H} NMR** (126 MHz, $CDCl_3$), δ (ppm) = 178.9, 138.9, 131.8, 126.9, 122.3 (q, *J* = 275.8 Hz), 122.0, 120.5, 120.2, 49.6 (q, *J* = 2.3 Hz), 48.5, 39.0, 38.9, 37.4-37.3 (m), 36.8 (q, *J* = 38.1 Hz), 25.2, 24.6, 21.2. **^{19}F{^{1}H} NMR** (376 MHz, $CDCl_3$), δ (ppm) = -73.4. **FT-IR** (cm^{-1}, neat, ATR), ṽ = 2931, 1693, 1487, 1390, 1375, 1295, 1167. **HRMS** (ESI+) calcd for $C_{19}H_{21}F_3NO$ $[M+H]^+$: 336.1570, found 336.1571.

8-Methoxy-1-methyl-1-((3-(trifluoromethyl)bicyclo[1.1.1]pentan-1-yl)methyl)-5,6-dihydro-4H-pyrrolo[3,2,1-ij]quinolin-2(1H)-one (21)

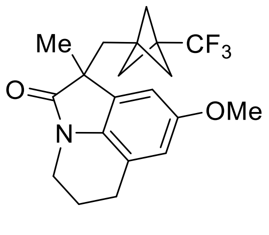

Prepared according to the *General Procedure* from the corresponding acrylamide **2s** (115.7 mg, 0.5 mmol, 2.0 equiv). After purification by flash column chromatography (DCM), the title compound **21** was obtained as a yellowish oil (56.7 mg, 0.16 mmol, 65%). **R_f** = 0.4 (silica gel, DCM); **mp**: 96 – 98ºC. **^{1}H NMR** (400 MHz, $CDCl_3$), δ (ppm) = 6.60 (s, 2H), 3.77 (s, 3H), 3.69 (t, *J* = 6.1 Hz, 2H), 2.77 (t, *J* = 6.1 Hz, 2H), 2.26 (d, *J* = 14.4 Hz, 1H), 2.11 – 1.87 (m, 3H), 1.49 (dd, *J* = 9.6, 2.0 Hz, 3H), 1.38 (dd, *J* = 9.6, 2.0 Hz, 3H), 1.31 (s, 3H). **^{13}C{^{1}H} NMR** (126 MHz, $CDCl_3$), δ (ppm) = 178.5, 156.2, 133.1, 132.6, 122.5 (q, *J* = 275.9 Hz), 120.8, 111.6, 108.4, 56.1, 49.7 (q, *J* = 2.2 Hz), 48.9, 39.0 (d, *J* = 5.2 Hz), 37.4 (d, *J* = 1.8 Hz), 37.0 (q, *J* = 37.8 Hz), 37.4, 25.4, 24.9, 21.4. **^{19}F{^{1}H} NMR** (377 MHz, $CDCl_3$), δ (ppm) = -73.3. **FT-IR** (cm^{-1}, neat, ATR), ṽ = 2931, 1693, 1487, 1390, 1375, 1295, 1167. **HRMS** (ESI+) calcd for $C_{20}H_{23}F_3NO_2$ $[M+H]^+$: 366.1675, found 366.1678.

1-(Trifluoromethyl)-1-((3-(trifluoromethyl)bicyclo[1.1.1]pentan-1-yl)methyl)-5,6-dihydro-4H-pyrrolo[3,2,1-ij]quinolin-2(1H)-one (22)

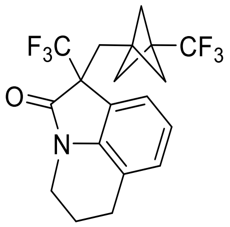

Prepared according to the *General Procedure* from the corresponding acrylamide **2t** (128 mg, 0.5 mmol, 2.0 equiv). After purification by flash column chromatography (DCM), the title compound **22** was obtained as a white solid (70 mg, 0.18 mmol, 72%). **R$_f$** = 0.75 (silica gel, DCM); **mp**: 98 - 100ºC. **^{1}H NMR** (500 MHz, $CDCl_3$), δ (ppm) = 7.18 (m, 2H), 7.03 (t, *J* = 7.6 Hz, 1H), 3.78 (t, *J* = 5.9 Hz, 2H), 2.85 (t, *J* = 6.1 Hz, 2H), 2.66 (d, *J* = 14.2 Hz, 1H), 2.37 (d, *J* = 14.2 Hz, 1H), 2.21 – 1.88 (m, 2H), 1.54 (dd, *J* = 9.6, 2.0 Hz, 3H), 1.42 (dd, *J* = 9.3, 1.8 Hz, 3H). **^{13}C{^{1}H} NMR** (126 MHz, $CDCl_3$), δ (ppm) = 169.8, 140.2, 129.2, 124.4 (q, *J* = 266.2 Hz), 123.0, 122.7, 122.2, 122.1 (q, *J* = 275.8 Hz), 120.9, 56.3 (q, *J* = 26.7 Hz), 49.7 (q, *J* = 2.3 Hz), 39.3, 37.2 (q, *J* = 38.4 Hz), 35.9 (q, *J* = 1.8 Hz), 31.4 (q, *J* = 2.3 Hz), 24.4, 20.9. **^{19}F{^{1}H} NMR** (376 MHz, $CDCl_3$), δ (ppm) = -73.4, -73.5. **FT-IR** (cm^{-1}, neat, ATR), ṽ = 2921, 1727, 1629, 1602, 1484, 1395, 1268, 1167. **HRMS** (ESI+) calcd for $C_{19}H_{18}F_6NO$ $[M+H]^+$: 390.1287, found 390.1290.

2,4-Dimethyl-4-((3-(trifluoromethyl)bicyclo[1.1.1]pentan-1-yl)methyl)isoquinoline-1,3(2H,4H)-dione (23)

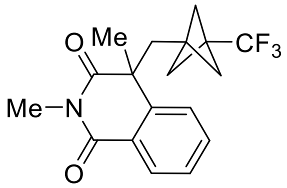

Prepared according to the *General Procedure* from the corresponding acrylamide **2u** (102 mg, 0.5 mmol, 2.0 equiv). After purification by flash column chromatography (DCM), the title compound **23** was obtained as an oil (55 mg, 0.16 mmol, 66%). **R$_f$** = 0.80 (silica gel, DCM). **^{1}H NMR** (500 MHz, $CDCl_3$), δ (ppm) = 8.24 (dd, *J* = 7.8, 2.1 Hz, 1H), 7.59 (td, *J* = 7.6, 1.4 Hz, 1H), 7.48 – 7.39 (m, 1H), 7.35 (d, *J* = 7.8 Hz, 1H), 3.35 (s, 3H), 2.68 (d, *J* = 14.5 Hz, 1H), 2.20 (d, *J* = 14.5 Hz, 1H), 1.50 (s, 3H), 1.38 (dd, *J* = 9.6, 2.2 Hz, 3H), 1.28 (dd, *J* = 9.5, 2.2 Hz, 3H). **^{13}C{^{1}H} NMR** (126 MHz, $CDCl_3$), δ (ppm) = 176.0, 164.2, 143.0, 133.9, 129.2, 127.7, 125.5, 124.5, 122.1 (q, *J* = 275.8 Hz), 49.4 (q, *J* = 2.3 Hz), 46.3, 42.1, 37.3 (q, *J* = 38.1 Hz), 37.2 (q, *J* = 1.3 Hz), 31.8, 27.3. **^{19}F{^{1}H} NMR** (376 MHz, $CDCl_3$), δ (ppm) = -73.4. **FT-IR** (cm^{-1}, neat, ATR), ṽ = 2957, 1695, 1650, 1448, 1420, 1345, 1290, 1174. **HRMS** (ESI+) calcd for $C_{18}H_{19}F_3NO_2$ $[M+H]^+$: 338.1362, found 338.1375.

4-Methyl-2,5-diphenyl-4-((3-(trifluoromethyl)bicyclo[1.1.1]pentan-1-yl)methyl)-2,4-dihydro-3H-pyrazol-3-one (24)

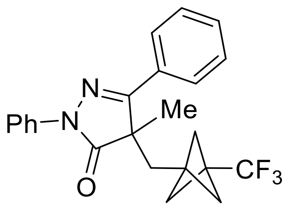

Prepared according to the *General Procedure* from the corresponding hydrazone **2v** (132 mg, 0.5 mmol, 2.0 equiv). After purification by flash column chromatography (DCM), the title compound **24** was obtained as an oil (31 mg, 0.09 mmol, 37%). **R$_f$** = 0.68 (silica gel, DCM). **^{1}H NMR** (500 MHz, $CDCl_3$), δ (ppm) = 8.07 (dd, *J* = 8.8, 1.1 Hz, 2H), 7.97 – 7.92 (m, 2H), 7.53 – 7.47 (m, 5H), 7.30 – 7.26 (m, 1H), 2.46 (s, 2H), 1.71 (dd, *J* = 9.5, 2.3 Hz, 3H), 1.63 – 1.58 (m, 6H). **^{13}C{^{1}H} NMR** (126 MHz, $CDCl_3$), δ (ppm) = 176.1, 161.2, 138.1, 131.0, 130.6, 129.1, 129.0, 126.3, 125.5, 122.2 (q, *J* = 275.8 Hz), 119.0, 53.1, 49.6 (q, *J* = 2.7 Hz), 38.7, 37.1 (q, *J* = 3.7 Hz), 37.0 (q, *J* = 38.2 Hz), 24.1. **^{19}F{^{1}H} NMR** (376 MHz, $CDCl_3$), δ (ppm) = -73.3. **FT-IR** (cm^{-1}, neat, ATR), ṽ = 2988, 1711, 1595, 1492, 1452, 1388, 1192. **HRMS** (ESI+) calcd for $C_{23}H_{22}F_3N_2O$ $[M+H]^+$: 399.1679, found 399.1680.

5-(4-Methoxyphenyl)-4-methyl-2-phenyl-4-((3-(trifluoromethyl)bicyclo[1.1.1]pentan-1-yl)methyl)-2,4-dihydro-3H-pyrazol-3-one (25)

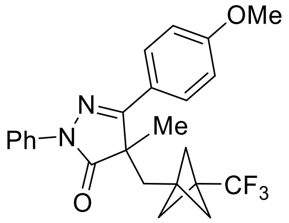

Prepared according to the *General Procedure* from the corresponding hydrazone **2w** (147 mg, 0.5 mmol, 2.0 equiv). After purification by flash column chromatography (DCM), the title compound **25** was obtained as an oil (36 mg, 0.10 mmol, 40%). **R$_f$** = 0.65 (silica gel, DCM). **^{1}H NMR** (500 MHz, $CDCl_3$), δ (ppm) = 8.06 (dd, *J* = 8.7, 1.2 Hz, 2H), 7.94 – 7.84 (m, 2H), 7.53 – 7.45 (m, 2H), 7.30 – 7.22 (m, 1H), 7.04 – 6.97 (m, 2H), 3.91 (s, 3H), 2.43 (s, 2H), 1.71 (dd, *J* = 9.5, 2.2 Hz, 3H), 1.61 (dd, *J* = 10.1, 2.6 Hz, 3H), 1.57 (s, 3H).**^{13}C{^{1}H} NMR** (126 MHz, $CDCl_3$), δ (ppm) = 176.0, 161.4, 161.0, 138.2, 129.0, 127.9, 125.3, 123.6, 122.2 (q, *J* = 275.7 Hz), 119.0, 114.4, 55.4, 53.0, 49.6 (q, *J* = 4.6 Hz), 38.7, 37.1 (q, *J* = 2.3 Hz), 37.0 (q, *J* = 38.6 Hz), 24.2. **^{19}F{^{1}H} NMR** (376 MHz, $CDCl_3$), δ (ppm) = -73.7. **FT-IR** (cm^{-1}, neat, ATR), ṽ = 2918, 1710, 1607, 1497, 1454, 1387, 1254, 1192. **HRMS** (ESI+) calcd for $C_{24}H_{24}F_3N_2O_2$ $[M+H]^+$: 429.1784, found 429.1784.

5-(4-bromophenyl)-4-methyl-2-phenyl-4-((3-(trifluoromethyl)bicyclo[1.1.1]pentan-1-yl)methyl)-2,4-dihydro-3H-pyrazol-3-one (26)

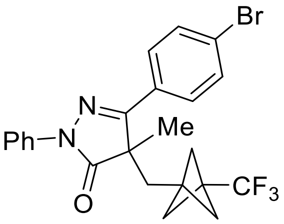

Prepared according to the *General Procedure* from the corresponding hydrazone **2x** (171 mg, 0.5 mmol, 2.0 equiv). After purification by flash column chromatography (DCM), the title compound **26** was obtained as an oil (51.3 mg, 0.11 mmol, 43%). **R_f** = 0.6 (silica gel, DCM); **mp**: 132 – 134ºC. **^{1}H NMR** (600 MHz, $CDCl_3$), δ (ppm) = 8.01 (d, *J* = 8.6 Hz, 2H), 7.79 (d, *J* = 8.6 Hz, 2H), 7.62 (d, *J* = 8.6 Hz, 2H), 7.47 (dd, *J* = 8.6, 7.3 Hz, 2H), 7.36 – 7.17 (m, 1H), 2.43 (d, *J* = 15.0 Hz, 1H), 2.37 (d, *J* = 15.0 Hz, 1H), 1.68 (dd, *J* = 9.5, 2.1 Hz, 3H), 1.59 (dd, *J* = 9.5, 2.1 Hz, 3H), 1.54 (s, 3H). **^{13}C{^{1}H} NMR** (126 MHz, $CDCl_3$), δ (ppm) = 176.0, 160.3, 138.0, 132.5 (2C), 130.0, 129.5 (2C), 127.8 (2C), 125.8, 125.2, 124,6 122.3 (q, *J* = 276.0 Hz), 119.1 (2C), 53.0, 49.7 (d, *J* = 2.4 Hz, 3C), 38.8, 37.5 – 36.3 (m), 24.1. **^{19}F{^{1}H} NMR** (377 MHz, $CDCl_3$), δ (ppm) = -73.3. **FT-IR** (cm^{-1}, neat, ATR), ṽ = 2918, 1710, 1607, 1497, 1454, 1387, 1254, 1192. **HRMS** (ESI+) calcd for $C_{23}H_{21}BrF_3N_2O$ $[M+H]^+$: 477.0784, found 477.0785.

4. Gram-Scale Synthesis of 3

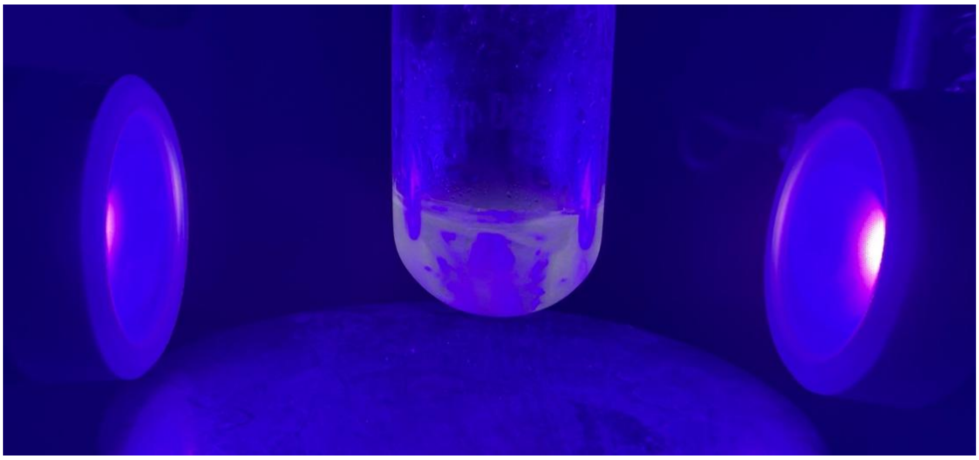

Figure S2. Gram scale synthesis reaction setup of **3**.

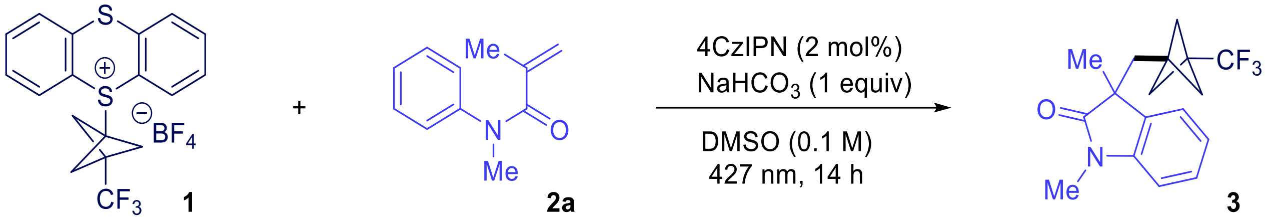

To a 25 mL Schlenk with a magnetic stir bar, the corresponding acrylamide **2a** (700.9 mg, 4.0 mmol, 2.0 equiv), 4CzIPN (32 mg, 0.02 equiv), $NaHCO_3$ (168 mg, 2.0 mmol, 1.0 equiv) and BCP-thianthrenium salt **1** (880 mg, 2.0 mmol, 1.0 equiv) were added. Then, 20 mL of dry DMSO were added under inert atmosphere and the reaction was degassed with Argon for 20 seconds. The reaction mixture was irradiated for 14 h with a 427 nm Kessil PR160-purple LED as described in the "Workflow" section. The temperature of the reaction was maintained at approximately 25°C via a fan. Upon completion, the reaction mixture was diluted with EtOAc (20 mL) and washed with brine (3 × 20 mL). The organic layer was dried over anhydrous Na_2SO_4, filtered and concentrated under reduced pressure. After purification by flash column chromatography (hexane:EtOAc 9:1), the title compound **3** was obtained as a white solid (365.6 mg, 1.2 mmol, 62%). $\mathbf{R_f}$ = 0.30 (silica gel, *n*-hexane / EtOAc, 6:1 (v/v)).

5. Data of X-Ray Structure of Compound 3

Colorless crystals of **3** were obtained from slow evaporation of DCM/pentane solutions. The single crystal X-Ray diffraction data set was collected at 295 K on a Bruker Smart APEX II diffractometer, using monochromatic MoKα radiation λ=0.71073 Å and 0.3° separation between frames. Data integrations were performed using SAINT V6.45A and SORTAV[3] in the diffractometer package[4]. The crystal and collection data and structural refinement parameters are given in Table S2. The structure was solved by direct methods using SHELXT-2014 and Fourier's difference methods and refined by least squares on F^2 using SHELXL-2014/7 inside the WinGX program environment[5]. Anisotropic displacement parameters were used for non-H atoms and the H-atoms were positioned in calculated positions and refined riding on their parent atoms (Fig. S3 and Tables S3, S4, S5).

Although a chiral center is present in the molecule (at C2) and the space group is non-centrosymmetric, the crystal was racemic, as indicated by the Flack parameter, approaching to 0.5. So, the refinement was finally conducted assuming a twinned crystal formed by enantiomorphic domains. This is not unexpected as the synthetic procedure does not impel enantioselectivity.

The folded conformation of the molecule is favored by an intramolecular H···π interaction among the BCP and the 5-membered ring [d(H13A···Cg) = 2.97 Å, γ= 19.9º, d(C13···Cg) = 3.347(4) Å]. The shortest intermolecular contact is of C-H···O type (see Table S6).

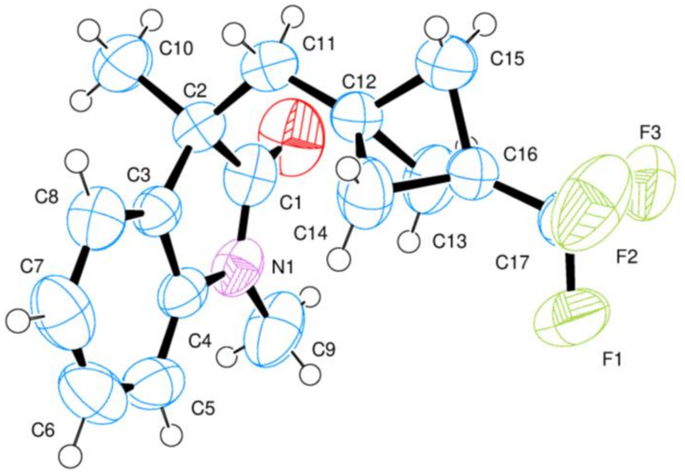

Figure S3. *Ortep view of **3** showing the atom labelling and the thermal vibration ellipsoids at 50% probability.*

---

[3] R. H. Blessing, *Acta Cryst. A*, **1995**, 51, 33–38.
[4] Bruker AXS Inc., SAINT, Madison, Wisconsin, USA.
[5] a) L. J. Farrugia, WinGX and ORTEP for Windows: an update, *J. Appl. Cryst.* 45 (**2012**) 849–854. b) G. M. Sheldrick, *Acta Cryst. A*, **2015**, 71, 3–8.

Table S2. Crystal data and structure refinement for **3**.

Identification code	**3**	
Empirical formula	$C_{17}H_{18}F_3NO$	
Formula weight	309.32	
Temperature	295(2) K	
Wavelength	0.71073 Å	
Crystal system	Orthorhombic	
Space group	P 21 21 21	
Unit cell dimensions	a = 9.684(2) Å	α= 90°.
	b = 12.495(3) Å	β= 90°.
	c = 13.311(3) Å	γ = 90°.
Volume	1610.6(6) $Å^3$	
Z	4	
Density (calculated)	1.276 Mg/m^3	
Absorption coefficient	0.103 mm^{-1}	
F(000)	648	
Crystal size	0.18 x 0.16 x 0.07 mm^3	
Theta range for data collection	2.236 to 28.496°.	
Index ranges	-12<=h<=12, -16<=k<=16, -17<=l<=17	
Reflections collected	47721	
Independent reflections	4064 [R(int) = 0.0650]	
Completeness to theta = 25.242°	100.0 %	
Refinement method	Full-matrix least-squares on F^2	
Data / restraints / parameters	4064 / 0 / 201	
Goodness-of-fit on F^2	1.023	
Final R indices [I>2sigma(I)]	R1 = 0.0475, wR2 = 0.1057	
R indices (all data)	R1 = 0.0958, wR2 = 0.1280	
Absolute structure parameter	0.5	
Largest diff. peak and hole	0.122 and -0.128 e.$Å^{-3}$	

Table S3. Atomic coordinates (x 10^4) and equivalent isotropic displacement parameters ($Å^2$x 10^3) for **3**. U(eq) is defined as one third of the trace of the orthogonalized U^{ij} tensor.

	x	y	z	U(eq)
F(1)	5249(3)	3442(2)	9597(2)	103(1)
F(2)	7179(2)	2872(3)	9106(2)	121(1)
F(3)	5908(3)	1903(2)	10024(2)	110(1)
O(1)	913(3)	550(2)	7214(2)	105(1)
N(1)	768(3)	2346(2)	6904(2)	65(1)
C(1)	1280(3)	1345(3)	6765(2)	68(1)
C(2)	2385(3)	1385(2)	5934(2)	57(1)
C(3)	2380(3)	2553(2)	5672(2)	50(1)
C(4)	1415(3)	3083(3)	6263(2)	53(1)
C(5)	1185(4)	4169(3)	6178(3)	74(1)
C(6)	1958(4)	4712(3)	5468(4)	83(1)
C(7)	2900(4)	4212(3)	4876(3)	84(1)
C(8)	3126(3)	3120(3)	4977(3)	67(1)
C(9)	-292(4)	2613(4)	7638(3)	101(2)

C(10)	1913(4)	688(3)	5040(3)	78(1)
C(11)	3777(4)	969(3)	6332(2)	68(1)
C(12)	4379(3)	1518(2)	7231(2)	55(1)
C(13)	3733(4)	1719(3)	8272(2)	76(1)
C(14)	5020(4)	2643(3)	7327(2)	77(1)
C(15)	5657(4)	1110(3)	7807(3)	90(1)
C(16)	5216(3)	2120(2)	8355(2)	55(1)
C(17)	5880(4)	2582(3)	9256(2)	69(1)

Table S4. Bond lengths [Å] and angles [°] for **3**.

F(1)-C(17)	1.318(4)
F(2)-C(17)	1.324(4)
F(3)-C(17)	1.329(4)
O(1)-C(1)	1.212(4)
N(1)-C(1)	1.358(4)
N(1)-C(4)	1.403(4)
N(1)-C(9)	1.455(4)
C(1)-C(2)	1.540(4)
C(2)-C(3)	1.502(4)
C(2)-C(11)	1.538(4)
C(2)-C(10)	1.543(4)
C(3)-C(8)	1.371(4)
C(3)-C(4)	1.389(4)
C(4)-C(5)	1.380(5)
C(5)-C(6)	1.384(5)
C(6)-C(7)	1.357(5)
C(7)-C(8)	1.388(5)
C(11)-C(12)	1.497(4)
C(12)-C(13)	1.541(4)
C(12)-C(14)	1.542(4)

C(12)-C(15)	1.543(4)
C(12)-C(16)	1.861(4)
C(13)-C(16)	1.525(5)
C(14)-C(16)	1.528(4)
C(15)-C(16)	1.520(5)
C(16)-C(17)	1.477(4)

C(1)-N(1)-C(4)	111.0(3)
C(1)-N(1)-C(9)	124.1(3)
C(4)-N(1)-C(9)	125.0(3)
O(1)-C(1)-N(1)	125.5(3)
O(1)-C(1)-C(2)	125.7(4)
N(1)-C(1)-C(2)	108.8(3)
C(3)-C(2)-C(11)	114.3(3)
C(3)-C(2)-C(1)	101.3(2)
C(11)-C(2)-C(1)	110.5(3)
C(3)-C(2)-C(10)	111.6(3)
C(11)-C(2)-C(10)	109.5(3)
C(1)-C(2)-C(10)	109.3(3)
C(8)-C(3)-C(4)	119.4(3)
C(8)-C(3)-C(2)	131.1(3)
C(4)-C(3)-C(2)	109.5(3)

C(5)-C(4)-C(3)	122.0(3)
C(5)-C(4)-N(1)	128.5(3)
C(3)-C(4)-N(1)	109.5(3)
C(4)-C(5)-C(6)	116.8(3)
C(7)-C(6)-C(5)	122.3(3)
C(6)-C(7)-C(8)	120.1(4)
C(3)-C(8)-C(7)	119.3(3)
C(12)-C(11)-C(2)	117.5(3)
C(11)-C(12)-C(13)	129.5(3)
C(11)-C(12)-C(14)	129.8(3)
C(13)-C(12)-C(14)	86.5(2)
C(11)-C(12)-C(15)	123.9(3)
C(13)-C(12)-C(15)	86.1(2)
C(14)-C(12)-C(15)	86.4(3)
C(11)-C(12)-C(16)	175.9(3)
C(13)-C(12)-C(16)	52.23(18)
C(14)-C(12)-C(16)	52.33(18)
C(15)-C(12)-C(16)	52.01(18)
C(16)-C(13)-C(12)	74.8(2)
C(16)-C(14)-C(12)	74.6(2)
C(16)-C(15)-C(12)	74.9(2)
C(17)-C(16)-C(15)	126.3(3)

C(17)-C(16)-C(13)	126.7(3)
C(15)-C(16)-C(13)	87.5(3)
C(17)-C(16)-C(14)	127.9(3)
C(15)-C(16)-C(14)	87.7(3)
C(13)-C(16)-C(14)	87.6(3)
C(17)-C(16)-C(12)	179.1(3)
C(15)-C(16)-C(12)	53.13(18)
C(13)-C(16)-C(12)	53.00(18)
C(14)-C(16)-C(12)	53.03(18)
F(1)-C(17)-F(2)	105.6(3)
F(1)-C(17)-F(3)	105.4(3)
F(2)-C(17)-F(3)	105.8(3)
F(1)-C(17)-C(16)	113.3(3)
F(2)-C(17)-C(16)	113.5(3)
F(3)-C(17)-C(16)	112.6(3)

Table S5. Anisotropic displacement parameters ($Å^2$x 10^3) for **3**. The anisotropic displacement factor exponent takes the form: $-2\pi^2[h^2a^{*2}U^{11} + ... + 2 h k a^* b^* U^{12}]$

	U^{11}	U^{22}	U^{33}	U^{23}	U^{13}	U^{12}
F(1)	115(2)	89(2)	104(2)	-38(1)	-8(1)	25(1)
F(2)	75(2)	187(3)	101(2)	-51(2)	-1(1)	-26(2)
F(3)	166(2)	104(2)	61(1)	3(1)	-23(2)	12(2)
O(1)	113(2)	103(2)	99(2)	24(2)	10(2)	-40(2)
N(1)	50(1)	92(2)	53(1)	-10(2)	8(1)	0(2)
C(1)	61(2)	82(3)	62(2)	1(2)	0(2)	-16(2)
C(2)	60(2)	55(2)	55(2)	-7(1)	2(1)	-1(1)
C(3)	45(1)	56(2)	48(2)	-5(1)	-1(1)	0(1)
C(4)	43(2)	65(2)	51(2)	-13(2)	-4(1)	4(1)
C(5)	60(2)	76(2)	87(2)	-25(2)	-19(2)	18(2)
C(6)	75(3)	63(2)	112(3)	7(2)	-29(3)	0(2)
C(7)	79(3)	79(3)	93(3)	25(2)	-17(2)	-15(2)
C(8)	58(2)	78(2)	63(2)	0(2)	3(2)	-5(2)
C(9)	66(2)	170(4)	69(2)	-20(3)	17(2)	6(3)
C(10)	88(3)	74(2)	73(2)	-17(2)	-10(2)	-1(2)
C(11)	77(2)	61(2)	65(2)	-12(2)	-3(2)	12(2)

C(12)	60(2)	54(2)	52(2)	0(1)	1(2)	7(1)
C(13)	76(2)	95(3)	58(2)	0(2)	5(2)	-14(2)
C(14)	96(3)	73(2)	61(2)	13(2)	-8(2)	-19(2)
C(15)	97(3)	86(3)	86(2)	-24(2)	-27(2)	35(2)
C(16)	58(2)	55(2)	50(2)	1(1)	1(1)	5(1)
C(17)	72(2)	72(2)	63(2)	-3(2)	5(2)	6(2)

Table S6. Hydrogen bonds for **3** [Å and °].

D-H...A	d(D-H)	d(H...A)	d(D...A)	<(DHA)
C(5)-H(5)...O(1)#1	0.93	2.49	3.418(4)	172.3

Symmetry transformations used to generate equivalent atoms:

#1 -x,y+1/2,-z+3/2

6. Mechanistic Investigation

6.1. TEMPO experiment

To a 4 mL Chemglass vial equipped with a magnetic stirring bar, acrylamide **2a** (44 mg, 0.25 mmol, 2.0 equiv), 4CzIPN (1.6 mg, 0.02 equiv), $NaHCO_3$ (8.4 mg, 0.1 mmol, 1.0 equiv), TEMPO (78 mg, 5 equiv) and BCP-thianthrenium salt **1** (44 mg, 0.1 mmol, 1.0 equiv) were added. Then, 0.5 mL of dry DMSO were added under inert atmosphere and the reaction was degassed with Argon for 20 seconds. The reaction mixture was irradiated for 4 h with a 427 nm Kessil PR160-purple LED as described in the "Workflow" section. The temperature of the reaction was maintained at approximately 25 °C via a fan. Upon completion, the reaction mixture was diluted with AcOEt (5 mL) and washed with brine (3 × 5 mL). The organic layer was dried over anhydrous Na_2SO_4, filtered and concentrated under reduced pressure. The crude mixture was analyzed by ^{1}H-NMR using 1,3,5-trimethoxybenzene as internal standard resulting in 0% yield of compound **3**. The mixture was also analyzed by HRMS, indicating the formation of a TEMPO adduct identified as **X**.

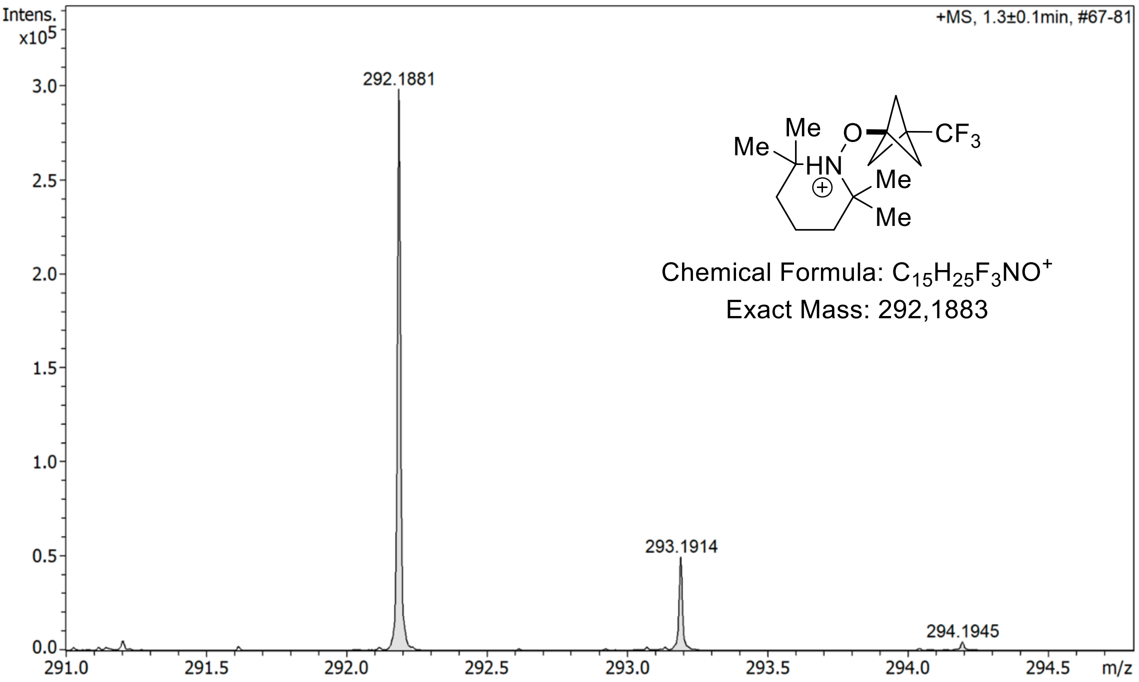

#	m/z	I	I %
1	292.1881	298261	100.0
2	293.1914	49695	16.7
3	294.1945	4805	1.6

Figure S4. *HRMS isotopic distribution of identified TEMPO-BCP-CF_3 adduct (**X**).*

6.2. Stern-Volmer Luminiscense Quenching Studies

Fluorescence measurements were obtained using septa-capped UV-Quartz cuvettes (10 mm pathlength) obtained from Hellma Analytics. Excitation was performed at 400 nm; fluorescence spectra were obtained from 300-700 nm. The stock solutions were prepared as follows:

1. 4CzIPN photocatalyst solution (0.0002 mM): To a 25 mL volumetric flask 3.86 mg of 4CzIPN were dissolved in DMSO. Subsequently, 10 µL of the previous solution were diluted in a 10 mL volumetric flask with more DMSO obtaining a $1.96.10^{-6}$ M stock solution.
2. Reagent **1** (thianthrenium-BCP-CF_3) solution (4 mM): To a 10 mL volumetric flask 19.3 mg of **1** were dissolved in DMSO obtaining a $4.00.10^{-3}$ M stock solution.
3. Acrylamide **2a** solution (4 mM): To a 10 mL volumetric flask 7.0 mg of **2a** and was dissolved in DMSO obtaining a $4.13.10^{-3}$ M stock solution.

Once the stock solutions were prepared, they were kept in the dark using aluminum foil. Then, the solutions were allocated to cuvettes and fluorescence quenching was determined with individual quenchers (reagent **1** and acrylamide **2a**). First, 1 mL of the 4CzIPN photocatalyst solution was added along with 1 mL of DMSO for the initial measurement. For the experiments with quencher, 1 mL of photocatalyst solution was added together with increasing amounts of quencher (0, 10, 50, 500 and 1000 µL) and adjusting concentration with DMSO to reach 2 mL. Degassing of each individual solution for 30 seconds was performed prior to recording the data. Linear regression of I_0/I against concentration was carried out to yield the Stern-Volmer quenching rate constant (K_{SV}). The following Stern-Volmer plot for luminescence quenching of 4CzIPN by two different quenchers was obtained. As depicted above, the excited catalyst (4CzIPN*) is quenched by the reagent **1** with a Stern-Volmer quenching rate constant of 64.3 M^{-1} rather than by the acrylamide substrate.

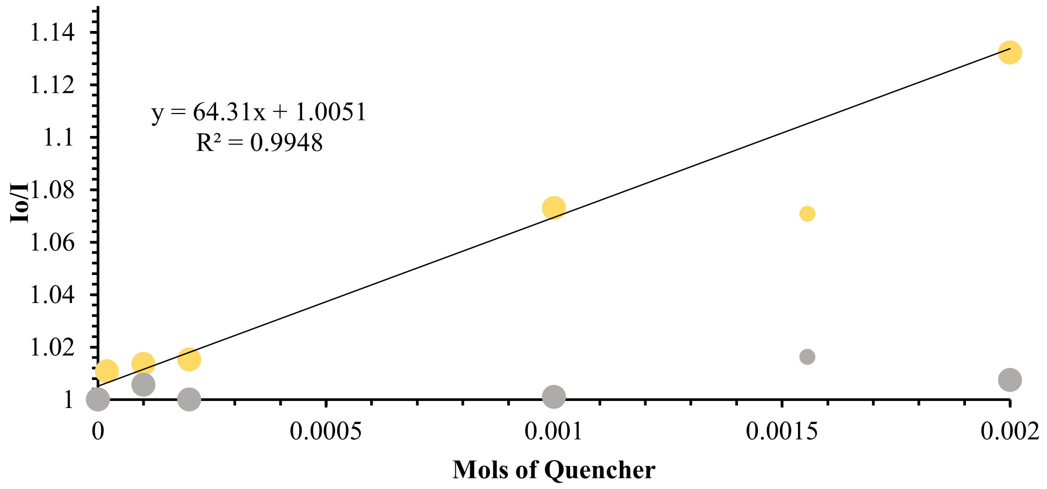

Figure S5. *Stern-Volmer plots for luminescence quenching of 4CzIPN ($2.0.10^{-6}$ M in degassed DMSO) by reagent **1** (orange) and oxindole **2a** (grey), $\lambda_{exc.}$= 400 nm, $\lambda_{em.}$= 546 nm. K_{SV} = Stern-Volmer constant.*

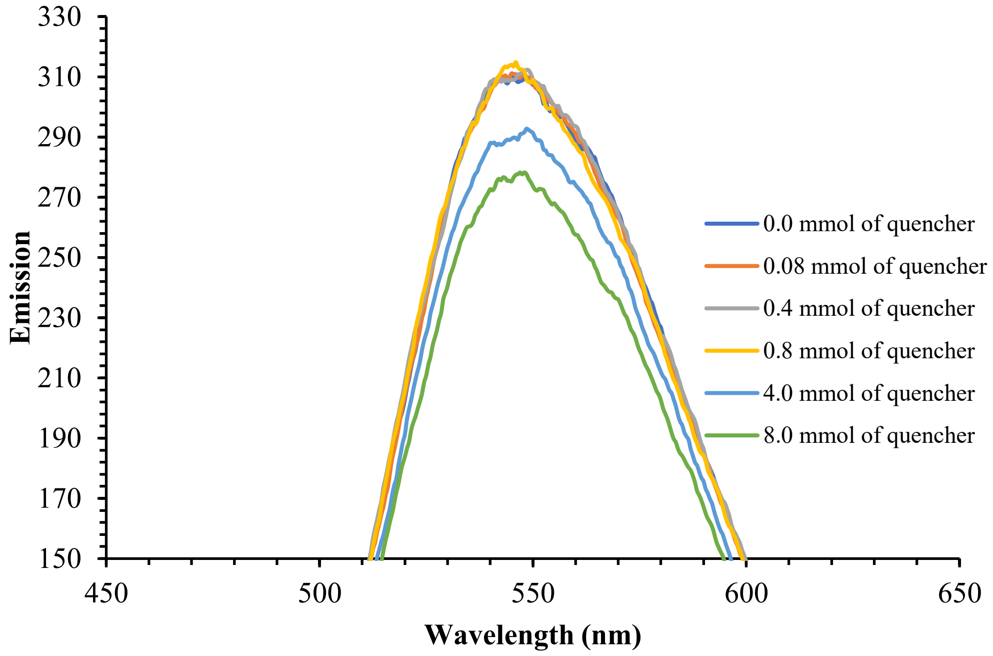

Figure S6. *Raw data for luminescence quenching of 4CzIPN (2.0 x 10^{-5} M in degassed DCM) by reagent* ***1****,* $\lambda_{exc.}$ *= 400 nm,* $\lambda_{em.}$ *= 546 nm).*

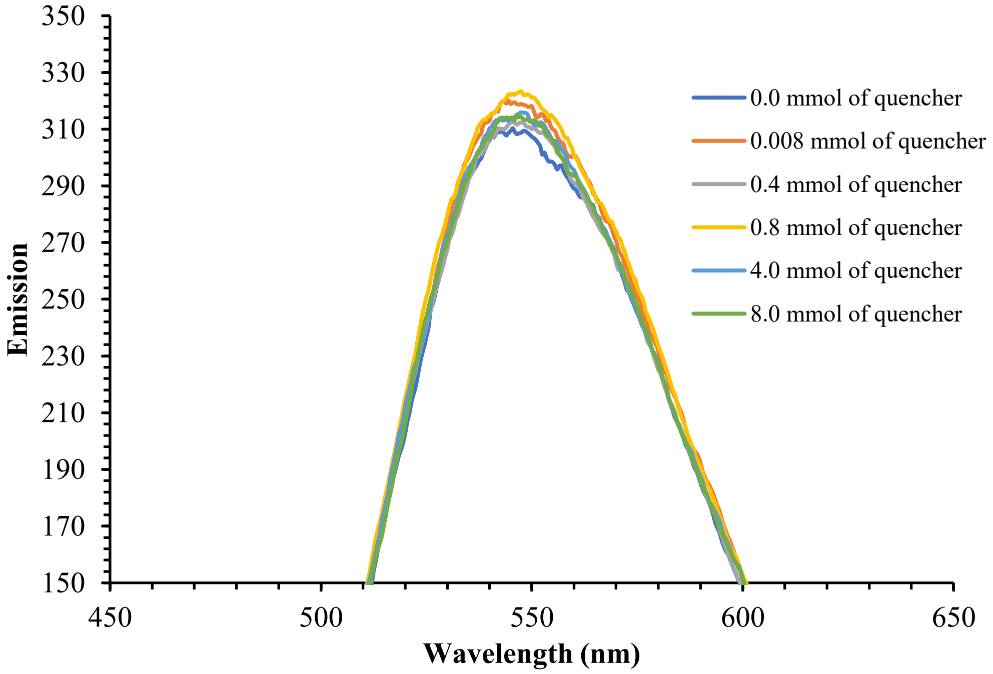

Figure S7. *Raw data for luminescence quenching of 4CzIPN (2.0 x 10^{-5} M in degassed DCM) by reagent* ***2a****,* $\lambda_{exc.}$ *= 400 nm,* $\lambda_{em.}$ *= 546 nm).*

6.3. Cyclic Voltammetry Experiments and Redox Data

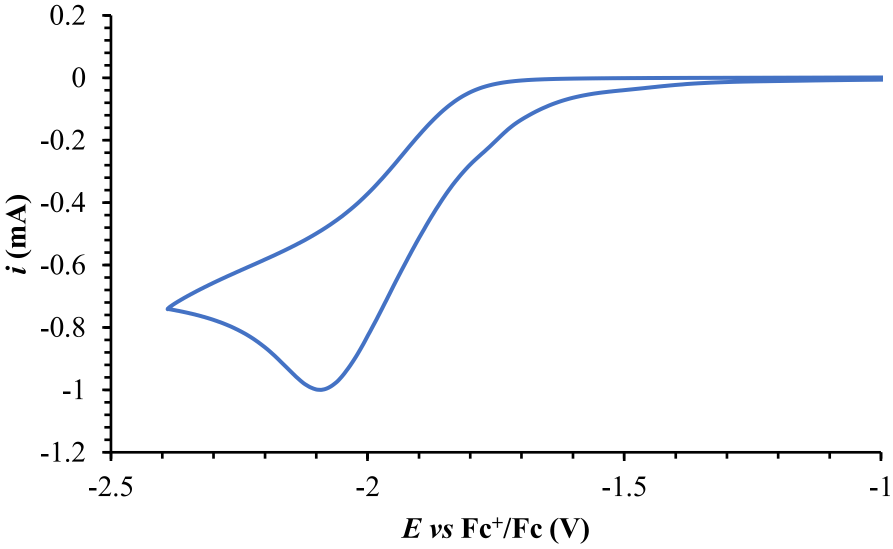

Figure S8. *Cyclic voltammetry experiment of reagent* ***1***. *Conditions: reagent* ***1*** *2.5 mM (100mV/s) in MeCN, TBAPF$_6$ 0.1 M, r.t. Scan direction to negative potentials. IUPAC plot.*

Table S7. Collected redox data of the presented work relevant compounds.

Compound	***E_c (V) vs Fc^+/Fc***
1	-1.89

6.4. Photochemical quantum yield

BCP-thianthrenium salt **1**, acrylamide **2a**, PC, Base were used as model substrates to determinate the quantum yield of this transformation, using 1,3,5-trimethoxybenzene as internal standard in a proportion 1:1 with **1.**

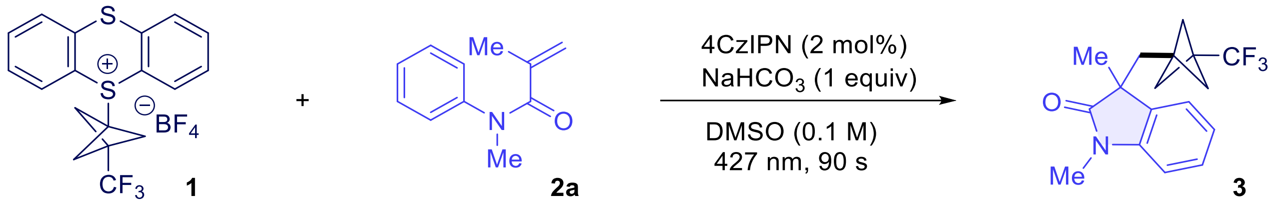

The quantum yield of the reaction is defined as:

$$\Phi(\text{reaction at 427 nm}) = \frac{\text{mol of formed product}}{\text{mol of photon flux} \cdot \text{t} \cdot \text{f}} \qquad (1)$$

where Φ is the quantum yield of the reaction, t is the time of the reaction (s), and f is the incident light absorbed by the EDA complex at 427 nm. The photon flux is calculated by standard ferrioxalate actinometry[4] (see Section *B.3*).

B.1. Incident light absorbed by the EDA complex

The fraction of light, f, absorbed was determined according to equation 2:

$$\text{F} = 1 - 10^{-\text{A}} \qquad (2)$$

Where A is the absorbance of the EDA complex in DMSO at 427 nm. The wavelength of 427nm was chosen based on the known absolute $\Phi(Fe^{+2})$ value. The absorbance of EDA complex was measured (0.1 M **1a**, 0.2 M **2a**) in DMSO (1 mL) to a cuvette equipped with a Teflon-coated magnetic stir bar and stirred for 90 seconds. The absorbance was recorded. The absorbance (A) at 427 nm was determined to be >4, thus indicating the fraction of light absorbed is ~1 according to equation 2.

B.2. The photoredox reaction

The photoredox transformation was developed using the general procedure for 30 min (0.5 h). Afterwards, 1,3,5-trimethoxybenzene was added as internal standard, and the reaction was worked up. The yield of the reaction was determined by ^{1}H NMR, where 0.026 mmols (26%) of the desired compound were obtained.

B.3. Photon flux at 427 nm

Standard ferrioxalate actinometry was used to determine the photon flux of the spectrophotometer using equations 3 and 4. For the ferrioxalate actinometer, the production of iron(II) ions proceeds by the following reactions:[3]

$$[Fe(C_2O_4)n]^{+(3-2n)} \xrightarrow{\text{light}} Fe^{+2} + (n-1)(C_2O_4)^{-2} + C_2O_4^{-}$$

$$[Fe(C_2O_4)n]^{+(3-2n)} + C_2O_4^{-} \longrightarrow Fe^{+2} + n(C_2O_4)^{-2} + 2\,CO_2$$

The moles of Fe^{+2} formed are determined spectrophotometrically by development with 1,10-phenanthroline (phen) to form the red $[Fe(phen)_3]^{+2}$ moiety (λ = 427 nm).[3] The photon flux is defined as shown in equation 3:

$$\text{Photon flux} = \frac{\text{mol Fe}^{+2}}{\Phi(\text{Fe}^{+2}) \cdot \text{t} \cdot \text{f}} \qquad (3)$$

where Φ is the quantum yield for the ferrioxalate actinometer (1.01 at λ = 427 nm),[4] t is the time (s), f ~1, and the mol of Fe^{+2} are calculated according to equation 4.

$$\text{mol (Fe}^{+2}) = \frac{\text{V} \cdot \Delta\text{A}}{\text{l} \cdot \epsilon} \qquad (4)$$

where V is the total volume of the solution, ΔA is the difference in absorbance between irradiated and nonirradiated solutions, l is the path length (1.0 cm), and ε is the molar absorptivity at 427 nm (11110 L mol^{-1} cm^{-1}).[3]

B.4. Experimental

The following solutions were prepared in the dark (flasks were wrapped in aluminum foil) and stored in the dark at room temperature:

– Ferrioxalate solution (0.15 M): ammonium ferrioxalate hydrate (1.284 g) was added to a flask wrapped in aluminum foil containing H_2SO_4 (20 mL, 0.05 M). The flask was stirred for complete solvation of the green solid in complete darkness. It is noteworthy that the solution should not be exposed to any incident light.

– Developer solution: 1,10-Phenanthroline (50 mg) and NaOAc (11.25 g) was added to a flask containing H_2SO_4 (50 mL, 0.5 M) and sonicated until completely solvated.

The absorbance of the non-irradiated sample. The buffered solution of phen (350 µL) was added to a ferrioxalate solution (2.0 mL) in a vial that had been covered with aluminum foil and with the lights of the laboratory switched off. The vial was capped and allowed to rest for 1 h and then transferred to a cuvette. The absorbance of the non-irradiated solution was measured at 427 nm to be 1.31 (average of two determinations, see *Figure S9*).

The absorbance of the irradiated sample. In a cuvette equipped with a stir bar was added the ferrioxalate solution (2.0 mL), and the stirred solution was irradiated for 90 s at λ = 427 nm with an excitation slit width = 10.0 nm. After irradiation, the buffered phen solution (350 µL) was added to the cuvette and allowed to rest for 1 h in the dark to allow the ferrous ions to coordinate completely to phen. The absorbance was measured at 427 nm to be 0.06 (average of two determinations, *Figure S9*).

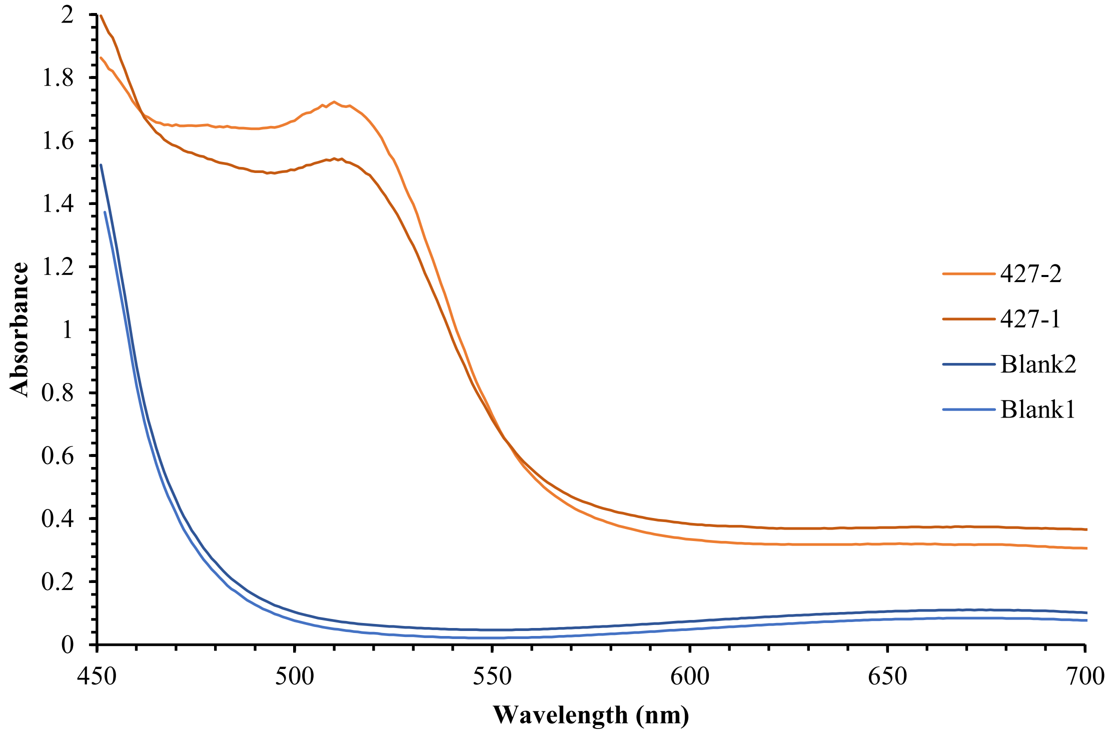

Figure S9. *Absorption spectra for irradiated and non-irradiated samples of red [Fe(phen)$_3$]$^{2+}$.*

Photon flux sample calculation. Sample calculation:

$$\text{mol}\ (\text{Fe}^{+2}) = \frac{\text{V} \cdot \Delta\text{A}}{\text{l} \cdot \epsilon} \qquad (4)$$

$$\text{mol}\ (\text{Fe}^{+2}) = \frac{0.00235\ \text{L} \cdot 1.57}{1.0\ \text{cm} \cdot 11100\ \text{L} \cdot \text{mol}^{-1}\text{cm}^{-1}} = 3.32\text{x}10^{-7}\text{mol}$$

$$\text{Photon flux} = \frac{\text{mol Fe}^{+2}}{\Phi(\text{Fe}^{+2}) \cdot \text{t} \cdot \text{f}} \qquad (3)$$

$$\text{Photon flux} = \frac{3.32\text{x}10^{-7}\text{mol}}{1.19 \cdot 90\ \text{s} \cdot 1} = \mathbf{3.09x10^{-9}\ einstein\ s^{-1}}$$

B.5. Quantum yield of the photoinduced transformation

Therefore, the quantum yield of the reaction was determined to be:

$$\Phi(\text{reaction at 427 nm}) = \text{mol of formed} \frac{\text{product}}{\text{mol of photon flux} \cdot \text{t} \cdot \text{f}} \qquad (1)$$

$$\boldsymbol{\Phi}(\textbf{reaction at 427 nm}) = \frac{2.6\text{x}10^{-5}\ \text{mol}}{3.09\text{x}10^{-9}\ \text{einstein s}^{-1} \cdot 1800\ \text{s} \cdot 1} = \mathbf{4.7}$$

6.5. Light on/off experiment

The model reaction was submitted to light on/off experiment, and the results are shown in *Graph S1*.

Light on/off experiments demonstrated that continuous irradiation is required to maintain productive reactivity, in line with the short-lived radical chain implied by the measured photochemical quantum yield (Φ = 4.7).

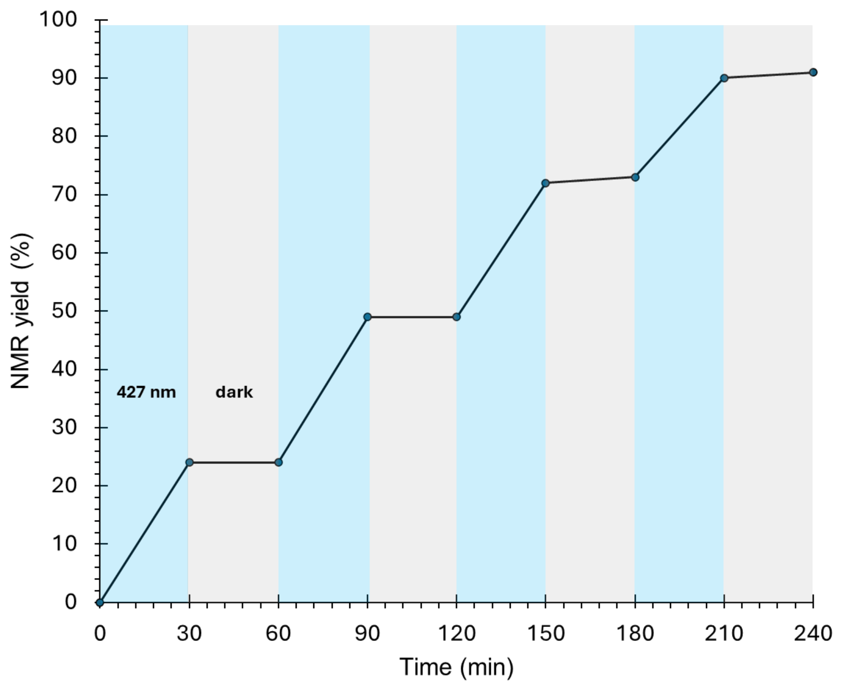

Graph S1. *Light on/off experiments for the model reaction. Reaction progress monitored under alternating periods of irradiation (λ_{max} = 427 nm) and darkness.*

7. Computational Details

All geometry optimizations of intermediates and transition states were achieved using spin-unrestricted B3LYP[6]-D3[7]/def2-SVP[8]method, in dimethylsulfoxide solvent using the CPCM solvent model[9] with “opt=noeigen” and “guess=mix” keywords as implemented in Gaussian16.[10] Frequency calculations were also conducted at the same level of theory to obtain vibrational frequencies to determine the identity of stationary points as intermediates (no imaginary frequencies) or transition states (only one imaginary frequency), as well as obtaining the thermochemistry: enthalpy (ΔH) and free energy (ΔG) at the temperature of 298 K. DFT optimization were also performed, with the same method stated previously, using 20 excitations above the excitation of interest. All structural figures were generated with CYLview.[11] Distances in structural figures are shown in Å and energies are in kcal/mol.

Single-point calculations were also performed at different levels of theory using the geometry from the optimization at:

uB3LYP-D3/def2tzvpp-CPCM(DCM)//uB3LYP-D3/def2svp-CPCM(DCM)[12]

uM06-d3/def2tzvpp-CPCM(DCM)//uB3LYP-D3/def2svp-CPCM(DCM)[13]

uM06-2X/def2tzvpp-CPCM(DCM)//uB3LYP-D3/def2svp-CPCM(DCM)[13]

---

[6] (a) Lee, C.; Yang, W.; Parr, R. G. *Phys. Rev. B* **1988**, *37*, 785–789. (b) Becke, A. D. *J. Chem. Phys.* **1993**, *98*, 5648–5652.

[7] (a) Grimme, S. *J. Comput. Chem.* **2004**, *25*, 1463-1473. (b) Grimme, S.; Antony, J.; Ehrlich, S.; Krieg, H. *J. Chem. Phys.* **2010**, *132*, 154104. (c) Grimme, S. *WIRES Comput. Mol. Sci.* **2011**, *1*, 211-228. (d) Ehrlich, S.; Moellmann, J.; Grimme, S. *Acc. Chem. Res.* **2012**, *46*, 916-926.

[8] (a) Weigend, F.; Ahlrichs, R. *Phys. Chem. Chem. Phys.* **2005**, *7*, 3297-3305. (b) Weigend, F. *Phys. Chem. Chem. Phys.* **2006**, *8*, 1057-1065.

[9] (a) Klamt, A.; Schüürmann, G. *J. Chem. Soc. Perkin Trans. 2* **1993**, *0*, 799-805. (b) Tomasi, J.; Persico, M. *Chem. Rev.* **1994**, *94*, 2027-2094. (c) Andzelm, J.; Kölmel, C.; Klamt, A. *J. Chem. Phys.* **1995**, *103*, 9312-9320. (d) Barone, V.; Cossi, M. *J. Phys. Chem. A* **1998**, *102*, 1995-2001. (e) Cossi, M.; Rega, N.; Scalmani, G.; Barone, V. *J. Comput. Chem.* **2003**, *24*, 669-681.

[10] Gaussian 16, Revision C.01, Frisch, M. J.; Trucks, G. W.; Schlegel, H. B.; Scuseria, G. E.; Robb, M. A.; Cheeseman, J. R.; Scalmani, G.; Barone, V.; Petersson, G. A.; Nakatsuji, H.; Li, X.; Caricato, M.; Marenich, A. V.; Bloino, J.; Janesko, B. G.; Gomperts, R.; Mennucci, B.; Hratchian, H. P.; Ortiz, J. V.; Izmaylov, A. F.; Sonnenberg, J. L.; Williams-Young, D.; Ding, F.; Lipparini, F.; Egidi, F.; Goings, J.; Peng, B.; Petrone, A.; Henderson, T.; Ranasinghe, D.; Zakrzewski, V. G.; Gao, J.; Rega, N.; Zheng, G.; Liang, W.; Hada, M.; Ehara, M.; Toyota, K.; Fukuda, R.; Hasegawa, J.; Ishida, M.; Nakajima, T.; Honda, Y.; Kitao, O.; Nakai, H.; Vreven, T.; Throssell, K.; Montgomery, J. A., Jr.; Peralta, J. E.; Ogliaro, F.; Bearpark, M. J.; Heyd, J. J.; Brothers, E. N.; Kudin, K. N.; Staroverov, V. N.; Keith, T. A.; Kobayashi, R.; Normand, J.; Raghavachari, K.; Rendell, A. P.; Burant, J. C.; Iyengar, S. S.; Tomasi, J.; Cossi, M.; Millam, J. M.; Klene, M.; Adamo, C.; Cammi, R.; Ochterski, J. W.; Martin, R. L.; Morokuma, K.; Farkas, O.; Foresman, J. B.; Fox, D. J. Gaussian, Inc., Wallingford CT, 2016.

[11] Legault, C. Y. (2009) CYLview, 1.0b, Université de Sherbrooke: Sherbrooke, Canada, http://www.cylview.org.

[12] Weigend, F.; Ahlrichs, R. *Phys. Chem. Chem. Phys.* **2005**, 7, 3297.

[13] Zhao, Y.; Truhlar, D. G. *Theor. Chem. Acc.* **2008**, *120*, 215-41.

uWb97xd/def2tzvpp-CPCM(DCM)//uB3LYP-D3/def2svp-CPCM(DCM)[14]

uPE1PBE/def2tzvpp-CPCM(DMSO)//uB3LYP-D3/def2svp-CPCM(DCM)[15]

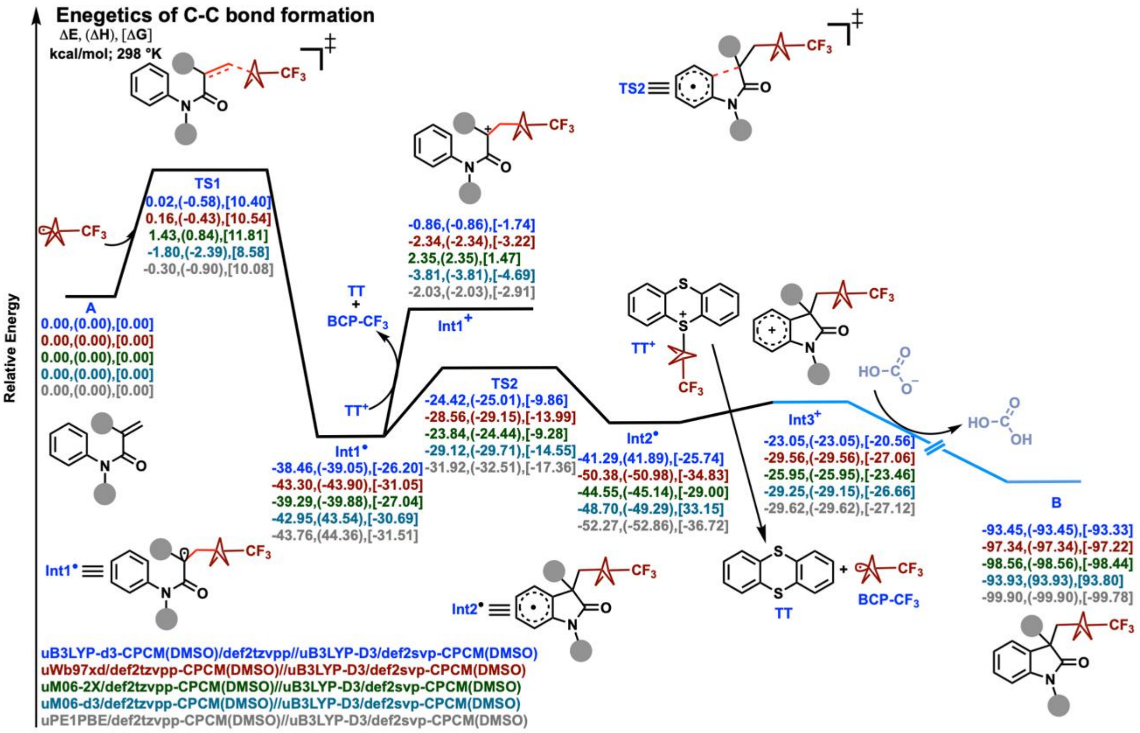

Figure S10: Full potential energy surface for radical cyclization at various levels of theory.

---

14 Chai, J.-D.; Head-Gordon, M. *Phys. Chem. Chem. Phys.* **2008**, *10*, 6615-6620.

15 a) Perdew, J. P.; Burke, K.; Ernzerhof, M. *Phys. Rev. Lett.* **1996**, *77*, 3865-3868. b) Adamo, C.; Barone, V. *J. Chem. Phys.* **1999**, *110*, 6158-6169.

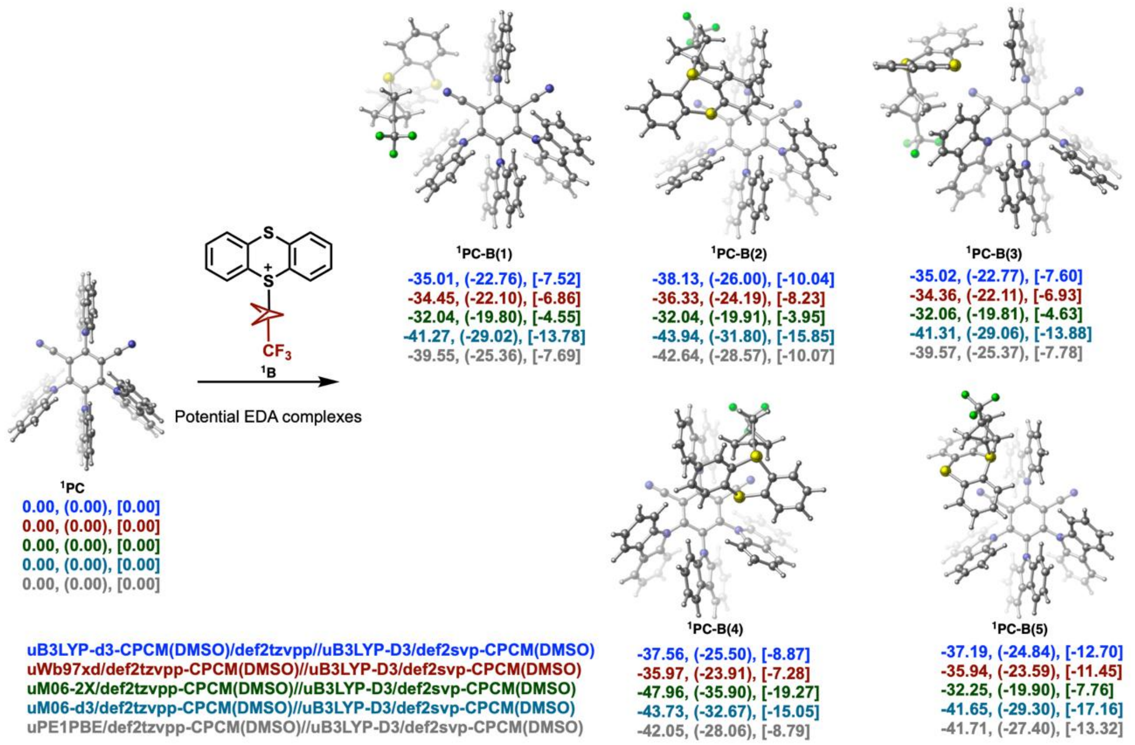

Figure S11: Potential EDA complexes calculated at different levels of theory. Calculated energies, enthalpy (parentheses) and Gibbs free energies [brackets] are given in kcal/mol.

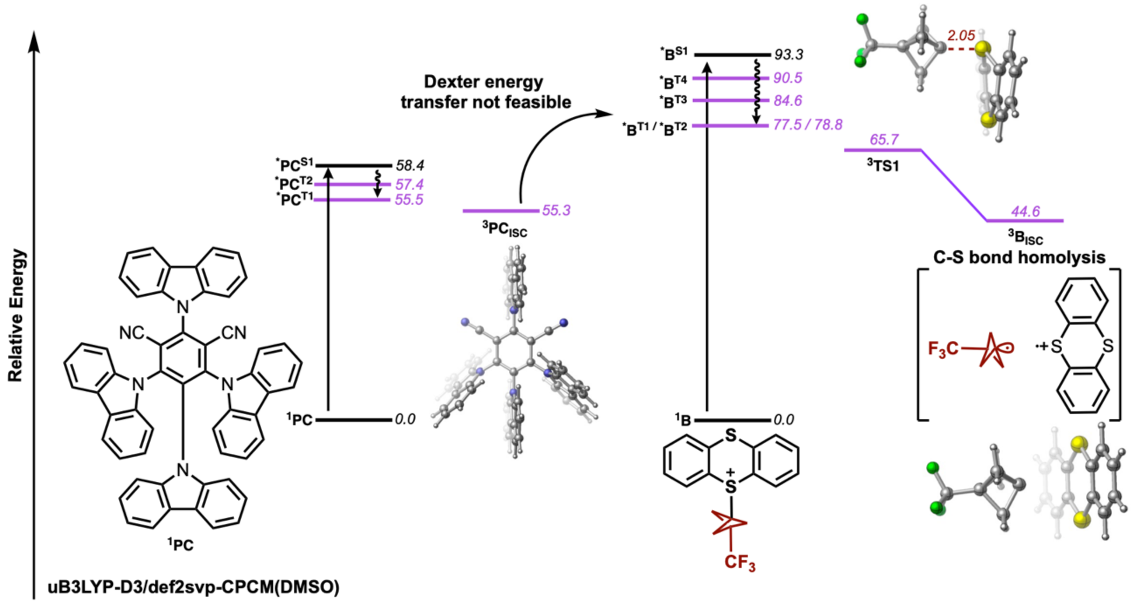

Figure S12: Dexter energy transfer process is not feasible as the triplet states of the two species (^{1}PC and ^{1}B) are not energitically aligned.

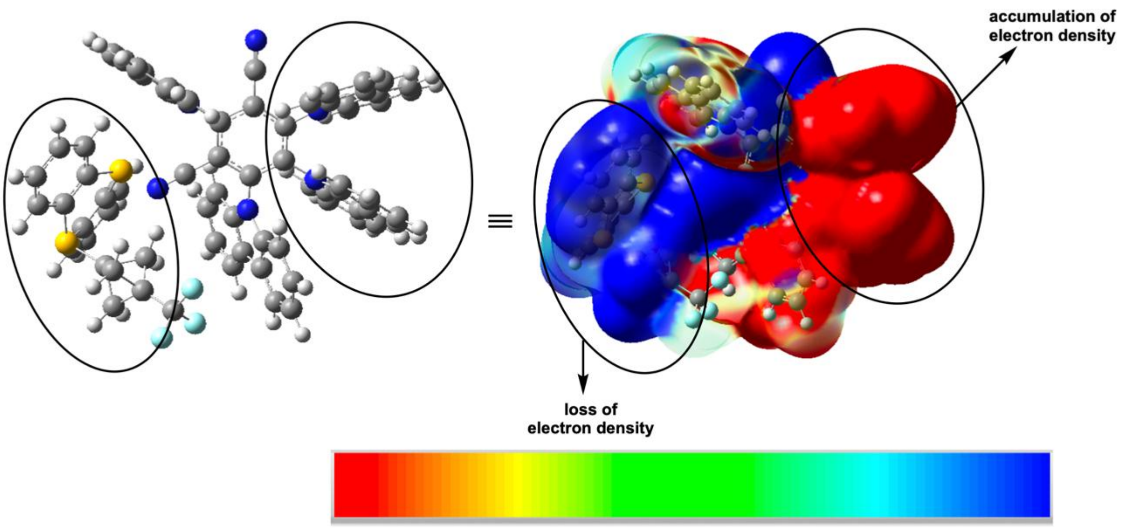

Figure S13: Electron density difference plot of EDA complex(isovalue = 0.0004).

Table S8. Cartesian coordinates (xyz format) and energies of all the structures involved in each reaction mechanism studied calculated at the CPCM(DCM) uB3LYP-d3/def2svp level of theory.

A

E(scf) = -556.592519722 a.u.

ν_{min} = 43.5544cm^{-1}

C	0.956877	-2.403589	1.339526
C	-0.375388	-2.696302	1.664426
C	1.983783	-2.786948	2.214081
C	-0.674473	-3.390619	2.839956
H	-1.173807	-2.380505	0.989443
C	1.679631	-3.466952	3.395987
H	3.021165	-2.562622	1.955601
C	0.350860	-3.776189	3.710175
H	-1.715156	-3.621581	3.080683
H	2.486454	-3.766102	4.069635
H	0.115675	-4.313566	4.632024
C	0.404682	-3.498466	-1.331965
C	0.810781	-2.062067	-1.102460
C	1.191984	-4.509720	-0.938983
H	0.948902	-5.545705	-1.193768
H	2.100135	-4.338200	-0.356316
C	-0.843919	-3.674684	-2.152203
H	-1.722854	-3.278049	-1.614648
H	-0.762055	-3.103564	-3.090252
H	-1.027376	-4.732730	-2.387280
O	0.776504	-1.267235	-2.039054
N	1.268535	-1.689182	0.139786
C	1.795402	-0.330465	0.282572
H	2.358414	-0.056551	-0.618133
H	0.979590	0.400674	0.418620
H	2.457041	-0.284673	1.156601

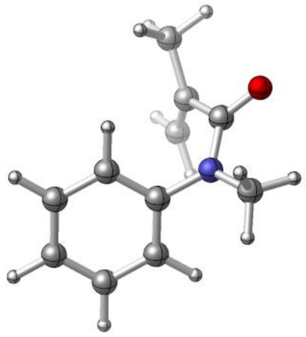

Zero-point correction= 0.215494 (Hartree/Particle)

Thermal correction to Energy= 0.228259

Thermal correction to Enthalpy= 0.229203

Thermal correction to Gibbs Free Energy= 0.175525

Sum of electronic and zero-point Energies= -556.377026

Sum of electronic and thermal Energies= -556.364261

Sum of electronic and thermal Enthalpies= -556.363317

Sum of electronic and thermal Free Energies= -556.416995

uB3LYP-D3/def2tzvpp-CPCM(DCM)

E(scf)= -557.211367471

uM06-d3/def2tzvpp-CPCM(DCM)

E(scf)= -556.775817422

uM06-2X/def2tzvpp-CPCM(DCM)
E(scf)= -556.941189225

uWb97xd/def2tzvpp-CPCM(DCM)

E(scf)= -556.994616233

uPE1PBE/def2tzvpp-CPCM(DMSO)

E(scf)= -556.531454022

BCP-CF3-radical

E(scf) = -531.247435172 a.u.

ν_{min} = 53.3605 cm^{-1}

C	0.599767	1.315733	-0.323282
C	2.132942	1.128873	-0.170926
C	1.614256	2.096112	0.921286
C	0.767170	2.846798	-0.135467
H	2.755710	1.553946	-0.969346
H	2.470980	0.135097	0.151742
H	1.305543	3.377609	-0.931925
H	-0.117785	3.391899	0.219124
C	0.426442	1.146032	1.209127
H	-0.478727	1.587175	1.647590
H	0.659707	0.153371	1.617102
C	-0.258260	0.655655	-1.376916
F	-0.195831	-0.685206	-1.289199
F	0.136997	1.000131	-2.615708
F	-1.549350	1.011032	-1.249403

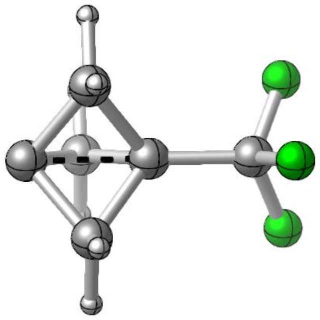

Zero-point correction= 0.108785 (Hartree/Particle)

Thermal correction to Energy= 0.116216

Thermal correction to Enthalpy= 0.117160

Thermal correction to Gibbs Free Energy= 0.075574

Sum of electronic and zero-point Energies= -531.138651

Sum of electronic and thermal Energies= -531.131220

Sum of electronic and thermal Enthalpies= -531.130275

Sum of electronic and thermal Free Energies= -531.171861

uB3LYP-D3/def2tzvpp-CPCM(DCM)

E(scf)= -531.866257375

uM06-d3/def2tzvpp-CPCM(DCM)

E(scf)= -531.589020230

uM06-2X/def2tzvpp-CPCM(DCM)

E(scf)= -531.665676123

uWb97xd/def2tzvpp-CPCM(DCM)

E(scf)= -531.707052338

uPE1PBE/def2tzvpp-CPCM(DMSO)

E(scf)= -531.313799838

TS1

E(scf) = -1087.84572933 a.u.

ν_{min} = -162.0412 cm^{-1}

C	1.534990	-2.350202	-0.107305	N	0.804544	-1.137406	-0.296392
C	1.474287	-3.032811	1.116551	C	1.555096	0.099136	-0.525789
C	2.336068	-2.855024	-1.142464	H	2.542166	-0.146034	-0.934652
C	2.188011	-4.221185	1.292918	H	1.015878	0.742414	-1.234523
H	0.863399	-2.627956	1.925982	H	1.687316	0.662101	0.413559
C	3.061007	-4.034823	-0.956085	C	-3.063176	-0.434565	-3.697006
H	2.375438	-2.326498	-2.097647	C	-1.584221	-0.744551	-3.348591
C	2.984867	-4.725401	0.259128	C	-2.491745	-1.838856	-2.737843
H	2.129091	-4.749032	2.248039	C	-3.456254	-0.745169	-2.227803
H	3.679391	-4.422277	-1.769560	H	-1.098800	-0.051685	-2.649072
H	3.547283	-5.651406	0.400661	H	-0.934133	-1.035232	-4.185370
C	-1.410299	-2.219198	0.033037	H	-3.060236	-0.068462	-1.459694
C	-0.533224	-1.002255	-0.004341	H	-4.501841	-1.035238	-2.054238
C	-1.345525	-3.139771	-0.960487	C	-3.285080	-1.924584	-4.069759
H	-2.047551	-3.976500	-0.991615	H	-4.319795	-2.290210	-4.017447
H	-0.513349	-3.168497	-1.663829	H	-2.748498	-2.289895	-4.956191
C	-2.518782	-2.182137	1.046978	C	-3.542374	0.746849	-4.501554
H	-3.176927	-3.057585	0.952211	F	-3.019741	0.743636	-5.742334
H	-2.115755	-2.158425	2.074420	F	-3.192791	1.909707	-3.919724
H	-3.122241	-1.268286	0.923854	F	-4.882621	0.746159	-4.628511
O	-1.030851	0.115769	0.145043				

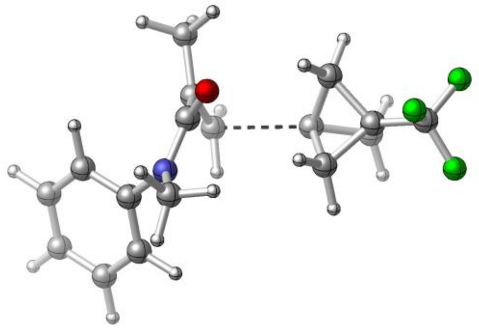

Zero-point correction= 0.324958 (Hartree/Particle)

Thermal correction to Energy= 0.346370

Thermal correction to Enthalpy= 0.347314

Thermal correction to Gibbs Free Energy= 0.269540

Sum of electronic and zero-point Energies= -1087.520772

Sum of electronic and thermal Energies= -1087.499359

Sum of electronic and thermal Enthalpies= -1087.498415

Sum of electronic and thermal Free Energies= -1087.576189

uB3LYP-D3/def2tzvpp-CPCM(DCM)

E(scf)= -1089.07949280

uM06-d3/def2tzvpp-CPCM(DCM)

E(scf)= -1088.36960368

uM06-2X/def2tzvpp-CPCM(DCM)
E(scf)= -1088.60648376

uWb97xd/def2tzvpp-CPCM(DCM)

E(scf)= -1088.70331160

uPE1PBE/def2tzvpp-CPCM(DMSO)

E(scf)= -1087.84763302

Int1_radical

E(scf) = -1087.91430483 a.u.

ν_{min} = 21.9625 cm^{-1}

C	1.221859	-2.023697	0.070234	N	0.300432	-0.963190	0.296816
C	1.290001	-3.109845	0.957970	C	0.829034	0.381982	0.519160
C	2.076275	-1.991241	-1.044455	H	0.121216	1.130688	0.138045
C	2.175150	-4.163640	0.713043	H	0.986161	0.577537	1.593869
H	0.643293	-3.125144	1.837425	H	1.786836	0.489602	-0.003558
C	2.973566	-3.036495	-1.272627	C	-2.393667	-0.948685	-3.612387
H	2.018385	-1.153141	-1.742023	C	-1.840417	-2.388540	-3.756343
C	3.021262	-4.131039	-0.400354	C	-1.882405	-2.086457	-2.226036
H	2.212322	-5.007946	1.405959	C	-1.288422	-0.680423	-2.562337
H	3.629214	-3.002649	-2.146259	H	-0.844123	-2.488200	-4.213143
H	3.717434	-4.952228	-0.586535	H	-2.542426	-3.149546	-4.129669
C	-1.709103	-2.401243	0.246300	H	-0.252429	-0.656908	-2.931257
C	-1.013727	-1.181035	0.690307	H	-1.484095	0.127599	-1.842565
C	-1.462845	-3.018981	-1.103173	C	-3.293711	-1.460270	-2.460122
H	-2.042382	-3.952066	-1.189986	H	-3.627950	-0.709016	-1.728773
H	-0.406965	-3.283033	-1.261033	H	-4.103160	-2.152388	-2.737613
C	-2.947047	-2.796271	0.980101	C	-2.802625	-0.036004	-4.725157
H	-2.973010	-3.889546	1.133670	F	-3.797024	-0.566315	-5.465724
H	-3.033782	-2.286502	1.948288	F	-1.775534	0.219641	-5.561094
H	-3.849593	-2.551031	0.385777	F	-3.241924	1.150670	-4.258541
O	-1.616582	-0.313348	1.334134				

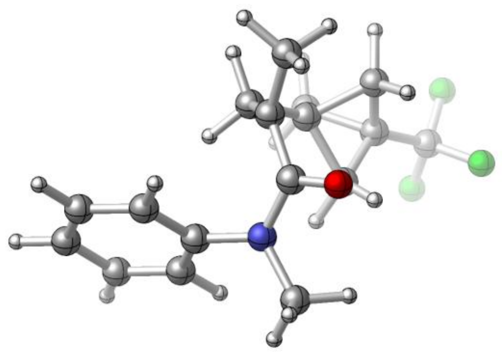

Zero-point correction= 0.328195 (Hartree/Particle)

Thermal correction to Energy= 0.349088

Thermal correction to Enthalpy= 0.350033

Thermal correction to Gibbs Free Energy= 0.275243

Sum of electronic and zero-point Energies= -1087.586110

Sum of electronic and thermal Energies= -1087.565216

Sum of electronic and thermal Enthalpies= -1087.564272

Sum of electronic and thermal Free Energies= -1087.639062

uB3LYP-D3/def2tzvpp-CPCM(DCM)
E(scf)= -1089.14352790

uM06-d3/def2tzvpp-CPCM(DCM)

E(scf)= -1088.43789554

uM06-2X/def2tzvpp-CPCM(DCM)

E(scf)= -1088.67409291

uWb97xd/def2tzvpp-CPCM(DCM)

E(scf)= -1088.77529053

uPE1PBE/def2tzvpp-CPCM(DMSO)

E(scf)= -1087.91961036

TS2

E(scf) = -1087.89254588 a.u.

ν_{min} = -474.0229 cm^{-1}

C	1.063631	-1.856623	0.406750	N	-0.002654	-1.010267	0.757541
C	0.717457	-3.248848	0.509735	C	0.178176	0.353629	1.219596
C	2.235796	-1.438527	-0.205986	H	0.502556	1.015956	0.398881
C	1.594331	-4.191473	-0.101292	H	-0.783356	0.715381	1.604190
H	0.139234	-3.576573	1.379494	H	0.931711	0.384674	2.019609
C	3.099618	-2.400087	-0.770566	C	-3.061494	-0.690050	-3.519927
H	2.461195	-0.373835	-0.294841	C	-3.430485	-1.808348	-2.514188
C	2.750488	-3.761372	-0.750874	C	-1.869738	-1.774555	-2.577606
H	1.374942	-5.258599	-0.016084	C	-2.008486	-1.649096	-4.127847
H	4.025696	-2.077728	-1.251777	H	-3.925764	-2.699169	-2.929758
H	3.418030	-4.495763	-1.209487	H	-3.881361	-1.486671	-1.564812
C	-1.172681	-2.801598	-0.352494	H	-2.396058	-2.530965	-4.660792
C	-1.249427	-1.513198	0.432580	H	-1.169803	-1.180363	-4.665092
C	-0.878592	-2.647903	-1.833629	C	-1.980825	-0.214041	-2.519598
H	-0.868669	-3.651035	-2.291368	H	-1.137300	0.352951	-2.942681
H	0.125591	-2.218215	-1.988358	H	-2.341211	0.219752	-1.577270
C	-2.178725	-3.849936	0.029731	C	-4.017146	0.178270	-4.275298
H	-2.266230	-3.953875	1.122545	F	-4.801387	0.895224	-3.444338
H	-3.182839	-3.600716	-0.357396	F	-4.833863	-0.548408	-5.065540
H	-1.895330	-4.826544	-0.394396	F	-3.371787	1.056366	-5.070296
O	-2.300807	-0.959579	0.731538				

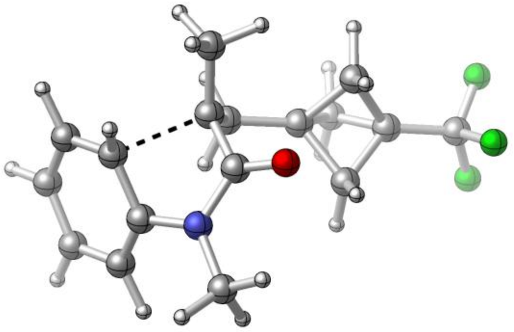

Zero-point correction= 0.327457 (Hartree/Particle)

Thermal correction to Energy= 0.347376

Thermal correction to Enthalpy= 0.348320

Thermal correction to Gibbs Free Energy= 0.277210

Sum of electronic and zero-point Energies= -1087.565089

Sum of electronic and thermal Energies= -1087.545170

Sum of electronic and thermal Enthalpies= -1087.544226

Sum of electronic and thermal Free Energies= -1087.615336

uB3LYP-D3/def2tzvpp-CPCM(DCM)

E(scf)= -1089.11944222

uM06-d3/def2tzvpp-CPCM(DCM)

E(scf)= -1088.41413926

uM06-2X/def2tzvpp-CPCM(DCM)

E(scf)= -1088.64776455

uWb97xd/def2tzvpp-CPCM(DCM)

E(scf)= -1088.75007951

uPE1PBE/def2tzvpp-CPCM(DMSO)

E(scf)= -1087.89902364

Int1_radical_cation

E(scf) = -1087.72702340 a.u.

ν_{min} = 15.0651 cm^{-1}

C	0.984927	-1.697224	0.206000	N	0.156904	-0.754282	0.916395
C	0.885529	-3.061251	0.502672	C	0.765645	0.265824	1.779317
C	1.866349	-1.235100	-0.778912	H	-0.031180	0.858500	2.245279
C	1.659997	-3.976895	-0.216598	H	1.366129	-0.229560	2.554350
H	0.219092	-3.400661	1.297583	H	1.408969	0.924639	1.180101
C	2.645228	-2.156802	-1.480834	C	-2.153530	1.904298	-2.412440
H	1.932731	-0.168832	-1.001517	C	-2.377242	0.645711	-3.287426
C	2.538966	-3.526040	-1.205466	C	-2.215596	0.142806	-1.823479
H	1.580809	-5.042710	0.007461	C	-1.018796	1.139126	-1.687217
H	3.331345	-1.803791	-2.253622	H	-1.571854	0.384281	-3.989419
H	3.146821	-4.242311	-1.762689	H	-3.373787	0.531244	-3.738212
C	-2.016320	-1.678919	-0.016562	H	-0.120265	0.915345	-2.280087
C	-1.150435	-0.821454	0.787085	H	-0.768275	1.497977	-0.679496
C	-2.316409	-1.314066	-1.416683	C	-3.152724	1.310641	-1.385712
H	-3.286179	-1.735791	-1.720896	H	-3.056725	1.653473	-0.346928
H	-1.541243	-1.886133	-1.975485	H	-4.202150	1.243026	-1.707113
C	-2.659717	-2.846330	0.612056	C	-2.098268	3.324330	-2.884962
H	-2.335148	-3.728064	0.025948	F	-3.249051	3.691560	-3.480166
H	-2.382137	-2.986849	1.662831	F	-1.106160	3.508694	-3.777326
H	-3.753887	-2.793052	0.492355	F	-1.883309	4.175160	-1.862850
O	-2.073616	-0.141560	1.273021				

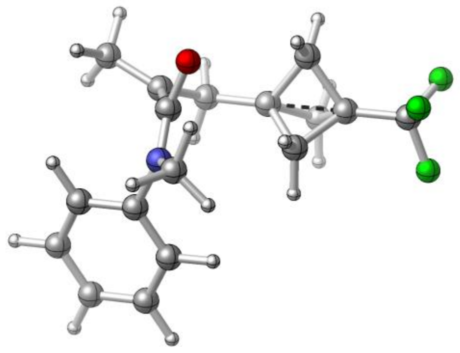

Zero-point correction= 0.328882 (Hartree/Particle)

Thermal correction to Energy= 0.349922

Thermal correction to Enthalpy= 0.350866

Thermal correction to Gibbs Free Energy= 0.276366

Sum of electronic and zero-point Energies= -1087.398142

Sum of electronic and thermal Energies= -1087.377102

Sum of electronic and thermal Enthalpies= -1087.376157

Sum of electronic and thermal Free Energies= -1087.450657

uB3LYP-D3/def2tzvpp-CPCM(DCM)

E(scf)= -1088.95587237

uM06-d3/def2tzvpp-CPCM(DCM)

E(scf)= -1088.24823387

uM06-2X/def2tzvpp-CPCM(DCM)

E(scf)= -1088.47753363

uWb97xd/def2tzvpp-CPCM(DCM)

E(scf)= -1088.58575850

uPE1PBE/def2tzvpp-CPCM(DMSO)

E(scf)= -1087.73068737

Int2_radical

E(scf) = -1087.92420326 a.u.

ν_{min} = 31.9706 cm^{-1}

C	1.283355	-1.846401	0.566243	N	0.249226	-0.949077	0.794247
C	0.662323	-3.194064	0.317180	C	0.429228	0.404380	1.278474
C	2.632524	-1.609207	0.515817	H	-0.562419	0.850056	1.424528
C	1.589219	-4.131847	-0.393953	H	0.976577	0.399990	2.233253
H	0.437324	-3.631374	1.319616	H	0.995439	1.005602	0.548618
C	3.486582	-2.651994	0.059953	C	-2.928024	-0.839090	-3.496769
H	3.040620	-0.626324	0.761266	C	-3.217128	-1.900544	-2.407506
C	2.936215	-3.868081	-0.438083	C	-1.659671	-1.813430	-2.527637
H	1.186682	-5.070698	-0.782813	C	-1.859987	-1.795962	-4.077182
H	4.565054	-2.486781	0.018795	H	-3.689838	-2.834696	-2.747596
H	3.612674	-4.606045	-0.879432	H	-3.648737	-1.533060	-1.466810
C	-0.729393	-2.821031	-0.282891	H	-2.228866	-2.726797	-4.535238
C	-0.976597	-1.464515	0.407108	H	-1.059220	-1.334273	-4.675319
C	-0.586626	-2.605553	-1.816527	C	-1.832388	-0.256738	-2.572561
H	-0.522632	-3.605135	-2.276959	H	-1.027794	0.309097	-3.067474
H	0.378855	-2.113513	-2.022695	H	-2.174370	0.228594	-1.649567
C	-1.827528	-3.828464	0.051327	C	-3.943118	-0.060434	-4.271368
H	-1.914449	-3.969376	1.139850	F	-4.726597	0.680978	-3.461119
H	-2.802805	-3.492434	-0.324066	F	-4.757224	-0.868643	-4.981393
H	-1.599666	-4.804342	-0.404994	F	-3.361732	0.786108	-5.146249
O	-2.047595	-0.903227	0.560259				

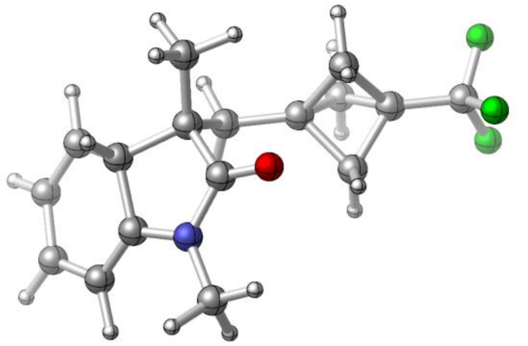

Zero-point correction= 0.329771 (Hartree/Particle)

Thermal correction to Energy= 0.349414

Thermal correction to Enthalpy= 0.350359

Thermal correction to Gibbs Free Energy= 0.280818

Sum of electronic and zero-point Energies= -1087.594432

Sum of electronic and thermal Energies= -1087.574789

Sum of electronic and thermal Enthalpies= -1087.573845

Sum of electronic and thermal Free Energies= -1087.643385

uB3LYP-D3/def2tzvpp-CPCM(DCM)

E(scf)= -1089.14837066

uM06-d3/def2tzvpp-CPCM(DCM)

E(scf)= -1088.44738121

uM06-2X/def2tzvpp-CPCM(DCM)

E(scf)= -1088.68279954

uWb97xd/def2tzvpp-CPCM(DCM)

E(scf)= -1088.78689873

uPE1PBE/def2tzvpp-CPCM(DMSO)

E(scf)= -1087.93348376

TT-cation-BCP

E(scf) -1789.07990287 a.u.

ν_{min} = 34.6659 cm^{-1}

C	-3.398758	-1.987581	3.709448	H	-2.615246	4.396723	1.632983
C	-2.095816	-1.486760	3.641536	S	-2.501971	1.579651	0.958089
C	-1.835016	-0.414857	2.787225	S	-0.192487	0.275233	2.480002
C	-2.851518	0.188297	2.026652	C	2.190457	-1.086116	4.883952
C	-4.152474	-0.322193	2.113113	C	2.435497	0.040348	3.846543
C	-4.417537	-1.415094	2.941942	C	0.934924	-0.371943	3.765288
C	-1.535219	2.582200	2.080477	C	1.398304	-1.858748	3.797424
C	-0.497211	2.019615	2.843134	H	3.058418	-0.215210	2.980062
C	0.250413	2.783154	3.740163	H	2.667415	1.033916	4.248732
H	1.043767	2.344612	4.337876	H	1.968782	-2.210601	2.928569
C	-0.024738	4.148924	3.849344	H	0.679492	-2.605420	4.155428
C	-1.037151	4.730174	3.080092	C	0.872521	-0.378121	5.316364
C	-1.801752	3.950853	2.208465	H	0.109071	-1.010568	5.785862
H	-3.610780	-2.831588	4.368271	H	0.979627	0.586408	5.827484
H	-1.311592	-1.944234	4.237293	C	3.214061	-1.670002	5.816599
H	-4.953630	0.147298	1.539155	F	3.711002	-0.728776	6.636065
H	-5.433582	-1.810637	3.000578	F	4.239526	-2.206195	5.135602
H	0.561226	4.756027	4.541711	F	2.675372	-2.636412	6.578284
H	-1.247407	5.797799	3.170718				

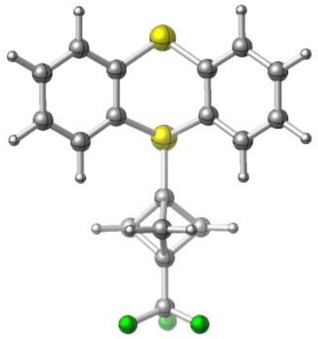

Zero-point correction= 0.276219 (Hartree/Particle)

Thermal correction to Energy= 0.295182

Thermal correction to Enthalpy= 0.296127

Thermal correction to Gibbs Free Energy= 0.228198

Sum of electronic and zero-point Energies= -1788.803684

Sum of electronic and thermal Energies= -1788.784720

Sum of electronic and thermal Enthalpies= -1788.783776

Sum of electronic and thermal Free Energies= -1788.851705

uB3LYP-D3/def2tzvpp-CPCM(DCM)

E(scf)= -1790.47376463

uM06-d3/def2tzvpp-CPCM(DCM)

E(scf)= -1789.74321774

uM06-2X/def2tzvpp-CPCM(DCM)

E(scf)= -1789.99709378

uWb97xd/def2tzvpp-CPCM(DCM)

E(scf)= -1790.09150963

uPE1PBE/def2tzvpp-CPCM(DMSO)

E(scf)= -1789.06049230

TT

E(scf) = -1257.95399156 a.u.

ν_{min} = 53.4820 cm^{-1}

C	-3.411129	-2.128788	3.605273	C	-0.937103	4.821010	3.000658
C	-2.145368	-1.537539	3.635199	C	-1.787448	4.017886	2.236125
C	-1.889640	-0.379584	2.885870	H	-3.595765	-3.039604	4.179500
C	-2.924260	0.206786	2.132203	H	-1.345633	-1.976636	4.236241
C	-4.202151	-0.371715	2.137142	H	-5.007762	0.099208	1.568974
C	-4.439287	-1.546008	2.856451	H	-5.433843	-1.997673	2.840817
C	-1.591598	2.629712	2.186941	H	0.749548	4.861373	4.359555
C	-0.557163	2.043527	2.941041	H	-1.087993	5.902797	3.019610
C	0.268778	2.852606	3.735372	H	-2.605216	4.466111	1.666935
H	1.055857	2.391263	4.336408	S	-2.629198	1.646019	1.115688
C	0.090719	4.238488	3.750160	S	-0.232824	0.288378	2.862104

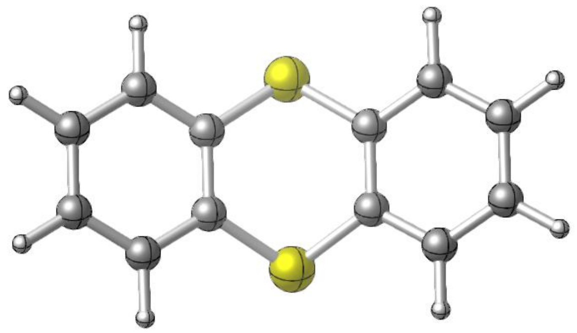

Zero-point correction= 0.162593 (Hartree/Particle)

Thermal correction to Energy= 0.173244

Thermal correction to Enthalpy= 0.174188

Thermal correction to Gibbs Free Energy= 0.125677

Sum of electronic and zero-point Energies= -1257.791398

Sum of electronic and thermal Energies= -1257.780748

Sum of electronic and thermal Enthalpies= -1257.779804

Sum of electronic and thermal Free Energies= -1257.828315

uB3LYP-D3/def2tzvpp-CPCM(DCM)

E(scf)= -1258.73035023

uM06-d3/def2tzvpp-CPCM(DCM)

E(scf)= -1258.27659228

uM06-2X/def2tzvpp-CPCM(DCM)

E(scf)= -1258.45672195

uWb97xd/def2tzvpp-CPCM(DCM)

E(scf)= -1258.50381783

uPE1PBE/def2tzvpp-CPCM(DMSO)

E(scf)= -1257.86421623

Int3_cation

E(scf) = -1087.77005500 a.u.

ν_{min} = 27.9641 cm^{-1}

C	1.267252	-1.875318	0.617528	N	0.300120	-0.977163	0.818893
C	0.676476	-3.188673	0.233188	C	0.470645	0.367224	1.358563
C	2.656951	-1.682416	0.693319	H	-0.528146	0.795664	1.501011
C	1.602340	-4.121509	-0.456652	H	0.995841	0.319507	2.321696
H	0.505600	-3.693176	1.214307	H	1.040744	0.985972	0.650854
C	3.455823	-2.677030	0.159455	C	-2.956035	-0.816160	-3.518407
H	3.083384	-0.753294	1.070410	C	-3.230871	-1.901141	-2.448000
C	2.938693	-3.875867	-0.445962	C	-1.675306	-1.776891	-2.556521
H	1.196207	-5.045052	-0.874749	C	-1.863936	-1.741469	-4.105966
H	4.539007	-2.529601	0.161205	H	-3.679686	-2.840159	-2.805097
H	3.640654	-4.585859	-0.885416	H	-3.685242	-1.555092	-1.509167
C	-0.725341	-2.812979	-0.331772	H	-2.209132	-2.673457	-4.578743
C	-0.968662	-1.481972	0.386812	H	-1.067915	-1.254374	-4.689235
C	-0.584354	-2.563138	-1.865256	C	-1.879205	-0.223750	-2.578019
H	-0.511061	-3.556556	-2.336052	H	-1.083240	0.366080	-3.057459
H	0.371070	-2.050340	-2.065791	H	-2.241691	0.246564	-1.654591
C	-1.821285	-3.827507	-0.005762	C	-3.983027	-0.046450	-4.287937
H	-1.906635	-3.984582	1.079486	F	-4.785943	0.663774	-3.470153
H	-2.792770	-3.480306	-0.377809	F	-4.773128	-0.861483	-5.015224
H	-1.594600	-4.792543	-0.482110	F	-3.412071	0.824962	-5.143604
O	-2.004032	-0.902780	0.569062				

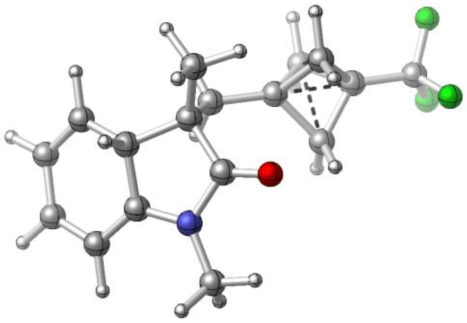

Zero-point correction= 0.332409 (Hartree/Particle)

Thermal correction to Energy= 0.352000

Thermal correction to Enthalpy= 0.352944

Thermal correction to Gibbs Free Energy= 0.283830

Sum of electronic and zero-point Energies= -1087.437646

Sum of electronic and thermal Energies= -1087.418055

Sum of electronic and thermal Enthalpies= -1087.417111

Sum of electronic and thermal Free Energies= -1087.486225

uB3LYP-D3/def2tzvpp-CPCM(DCM)

E(scf)= -1088.99332248

uM06-d3/def2tzvpp-CPCM(DCM)

E(scf)= -1088.29070504

uM06-2X/def2tzvpp-CPCM(DCM)

E(scf)= -1088.52472410

uWb97xd/def2tzvpp-CPCM(DCM)

E(scf)= -1088.63122000

uPE1PBE/def2tzvpp-CPCM(DMSO)

E(scf)= -1087.77673692

HCO3

E(scf) = -264.341052199 a.u.

ν_{min} = 525.8619 cm^{-1}

C	-0.355561	-0.327386	0.286985	O	0.165889	-1.390673	-0.085516
O	-1.666944	-0.123119	-0.208212	O	0.084347	0.583039	1.020114
H	-1.939717	0.731410	0.155976				

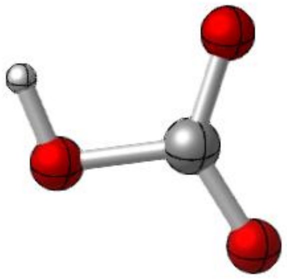

Zero-point correction= 0.026530 (Hartree/Particle)

Thermal correction to Energy= 0.030025

Thermal correction to Enthalpy= 0.030969

Thermal correction to Gibbs Free Energy= 0.000785

Sum of electronic and zero-point Energies= -264.314522

Sum of electronic and thermal Energies= -264.311027

Sum of electronic and thermal Enthalpies= -264.310083

Sum of electronic and thermal Free Energies= -264.340267

uB3LYP-D3/def2tzvpp-CPCM(DCM)

E(scf)= -264.679706692

uM06-d3/def2tzvpp-CPCM(DCM)

E(scf)= -264.549665243

uM06-2X/def2tzvpp-CPCM(DCM)

E(scf)= -264.578751634

uWb97xd/def2tzvpp-CPCM(DCM)

E(scf)= -264.589489470

uPE1PBE/def2tzvpp-CPCM(DMSO)

E(scf)= -264.395302314

H_2CO_3

E(scf) -264.837633421 a.u.

ν_{min} = 63.9290 cm^{-1}

C	-0.242570	-0.145833	0.028614	O	0.098794	0.439630	1.101879
O	-1.628856	-0.015488	-0.360183	O	0.093404	-1.538143	-0.083316
H	-1.992384	0.561478	0.333680	H	0.377356	-1.774342	0.819906

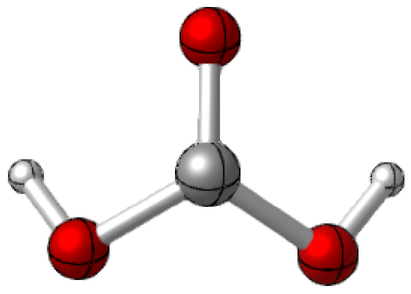

Zero-point correction= 0.033665 (Hartree/Particle)

Thermal correction to Energy= 0.038630

Thermal correction to Enthalpy= 0.039574

Thermal correction to Gibbs Free Energy= 0.005345

Sum of electronic and zero-point Energies= -264.803968

Sum of electronic and thermal Energies= -264.799003

Sum of electronic and thermal Enthalpies= -264.798059

Sum of electronic and thermal Free Energies= -264.832288

uB3LYP-D3/def2tzvpp-CPCM(DCM)

E(scf)= -265.182777993

uM06-d3/def2tzvpp-CPCM(DCM)

E(scf)= -265.042040960

uM06-2X/def2tzvpp-CPCM(DCM)

E(scf)= -265.074680592

uWb97xd/def2tzvpp-CPCM(DCM)

E(scf)= -265.088566937

uPE1PBE/def2tzvpp-CPCM(DMSO)

E(scf)= -264.895354390

B

E(scf) -1087.37559358 a.u.

ν_{min} = - 28.0045 cm^{-1}

C	1.465801	-2.094749	0.626697	H	-2.265313	-2.600367	-4.798922
C	1.058393	-3.039240	-0.331815	H	-1.373159	-1.015432	-4.935908
C	2.749945	-2.098925	1.167951	C	-2.123495	-0.210730	-2.706707
C	1.947648	-4.012666	-0.766704	H	-1.478592	0.515391	-3.226122
C	3.639635	-3.090380	0.720955	H	-2.451975	0.150953	-1.724743
H	3.055859	-1.362382	1.912766	C	-4.384424	-0.303375	-4.211697
C	3.249182	-4.036971	-0.232191	F	-5.208863	0.238094	-3.291205
H	1.642005	-4.751360	-1.512296	F	-5.105846	-1.204515	-4.910283
H	4.653237	-3.118949	1.128876	F	-4.042693	0.681666	-5.067993
H	3.957853	-4.799584	-0.563484				
C	-0.375190	-2.747307	-0.731402				
C	-0.724967	-1.561840	0.198042				
C	-0.418166	-2.335688	-2.231341				
H	-0.198548	-3.247658	-2.811785				
H	0.412510	-1.638504	-2.430298				
C	-1.316610	-3.926243	-0.429070				
H	-1.220012	-4.241297	0.621084				
H	-2.363460	-3.649860	-0.609245				
H	-1.063760	-4.784619	-1.069985				
O	-1.812818	-1.021936	0.313652				
N	0.404403	-1.224937	0.913245				
C	0.457062	-0.160844	1.891531				
H	0.690262	-0.560165	2.891898				
H	1.225952	0.580163	1.620685				
H	-0.525404	0.326994	1.916191				
C	-3.182422	-0.927528	-3.577789				
C	-3.180958	-2.081450	-2.545985				
C	-1.680422	-1.709966	-2.783893				
C	-2.023127	-1.644701	-4.308382				
H	-3.514569	-3.064917	-2.910961				
H	-3.581197	-1.847626	-1.550432				

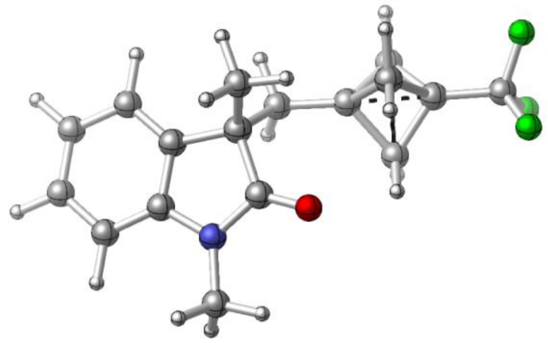

Zero-point correction= 0.320445 (Hartree/Particle)

Thermal correction to Energy= 0.339828

Thermal correction to Enthalpy= 0.340772

Thermal correction to Gibbs Free Energy= 0.271915

Sum of electronic and zero-point Energies= -1087.055148

Sum of electronic and thermal Energies= -1087.035765

Sum of electronic and thermal Enthalpies= -1087.034821

Sum of electronic and thermal Free Energies= -1087.103678

uB3LYP-D3/def2tzvpp-CPCM(DCM)

E(scf)= -1088.59887164

uM06-d3 /def2tzvpp-CPCM(DCM)

E(scf)= -1087.89798294

uM06-2X/def2tzvpp-CPCM(DCM)

E(scf)= -1088.14093587

uWb97xd/def2tzvpp-CPCM(DCM)

E(scf)= -1088.23658979

uPE1PBE/def2tzvpp-CPCM(DMSO)

E(scf)= -1087.38511451

[1]PC

E(scf) -2480.03728099 a.u.

ν_{min} = 10.8082 cm^{-1}

N	7.050688	6.131497	12.965845	C	10.886643	6.070299	14.303812
N	10.495674	8.277371	18.509202	H	10.520455	5.187293	14.830656
N	9.203644	7.693341	15.279751	C	7.786420	7.098629	20.530758
N	8.055155	5.990467	19.702176	C	9.645724	7.626966	18.061573
N	6.189230	4.150223	18.525822	C	5.226480	4.516894	19.486687
N	5.876579	4.152773	15.669423	C	9.129061	3.727885	20.113691
C	7.519763	5.933659	15.510238	H	9.123502	3.398660	19.075182
C	8.618042	6.808014	17.485208	C	8.143514	6.769572	21.862855
C	6.935770	5.035753	17.718633	C	10.780092	8.482499	13.811180
C	7.852389	5.946110	18.308369	C	5.041547	2.491143	14.318663
C	8.987793	9.074650	15.141073	C	7.123396	1.913414	17.765212
C	6.781399	5.024544	16.306719	H	7.839320	2.314073	17.048230
C	6.282080	2.744739	18.510920	C	9.220087	4.582621	22.825395
C	8.449818	6.816976	16.088484	H	9.271007	4.914129	23.865240
C	7.263562	6.027003	14.101452	C	8.006379	9.882349	15.724424
C	10.292704	7.325138	14.472255	H	7.255644	9.474343	16.403305
C	4.703674	3.341235	20.083806				
C	6.225111	3.140468	14.752321				
C	8.617512	4.971504	20.494763				
C	8.675444	5.419012	21.839792				
C	4.749743	5.784118	19.836316				
H	5.125838	6.691415	19.364800				
C	9.949226	9.595719	14.236125				
C	4.474136	4.176305	15.790497				

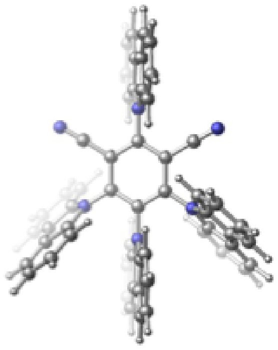

Zero-point correction= 0.725119 (Hartree/Particle)

Thermal correction to Energy= 0.770272

Thermal correction to Enthalpy= 0.771216

Thermal correction to Gibbs Free Energy= 0.644256

Sum of electronic and zero-point Energies= -2479.312162

Sum of electronic and thermal Energies= -2479.267009

Sum of electronic and thermal Enthalpies= -2479.266065

Sum of electronic and thermal Free Energies= -2479.393025

uB3LYP-D3/def2tzvpp-CPCM(DCM)

E(scf)= -2482.81874805

uM06-d3 /def2tzvpp-CPCM(DCM)

E(scf)= -2480.86816546

uM06-2X/def2tzvpp-CPCM(DCM)

E(scf)= -2481.74758393

uWb97xd/def2tzvpp-CPCM(DCM)

E(scf)= -2481.84252052

uPE1PBE/def2tzvpp-CPCM(DMSO)

E(scf)= -2479.86459826

[1]PC-B(1)

E(scf) -4269.30209195 a.u.

ν_{min} = 13.6392 cm^{-1}

N	6.226377	7.566399	13.269320	C	9.280912	10.798696	14.964507
N	10.217574	8.714739	18.741337	C	4.348498	4.481584	15.886729
N	8.644138	8.682839	15.573143	C	10.134344	7.387565	13.991742
N	8.038879	6.045090	19.623708	H	9.811100	6.397362	14.316178
N	6.244551	4.316081	18.239681	C	7.736843	6.932792	20.677424
N	5.648614	4.853476	15.496493	C	9.374050	8.099860	18.235690
C	7.096902	6.799868	15.584176	C	5.404292	4.386727	19.364436
C	8.345101	7.339064	17.591841	C	9.025532	3.714835	19.532496
C	6.830244	5.410741	17.581508	H	9.067344	3.650539	18.446157
C	7.724970	6.264950	18.274490	C	8.017178	6.287634	21.907656
C	8.446989	10.047395	15.832253	C	10.021658	9.845211	14.156679
C	6.526872	5.673096	16.221624	C	4.969242	3.153823	14.107127
C	6.387054	2.966352	17.874515	C	7.164888	2.398060	16.863457
C	8.029504	7.615005	16.250181	H	7.778638	3.008464	16.202896
C	6.632830	7.200229	14.291605	C	8.980483	3.894437	22.372672
C	9.600563	8.549379	14.555447	H	8.978599	3.968186	23.462603
C	5.009911	3.072619	19.721545	C	7.581777	10.655504	16.745342
C	6.026096	4.053331	14.396133	H	6.935716	10.064948	17.397348
C	8.531404	4.849844	20.177974	C	5.631233	2.169330	18.770677
C	8.523998	4.964427	21.590525	C	3.529118	5.044666	16.866196
C	4.926333	5.507617	20.046765	H	3.864183	5.882894	17.476061
H	5.194840	6.518939	19.745246	C	7.191818	8.218349	20.626938

H	6.956964	8.705335	19.679851	C	4.149053	2.880012	20.81092
C	7.570463	12.051832	16.787473	H	3.83888	1.871958	21.09589
H	6.906481	12.557217	17.492747	C	8.393198	12.817099	15.937733
C	3.904365	3.426008	15.054498	H	8.357876	13.907349	15.995424
C	7.231544	4.022966	13.691721	C	7.142176	1.007988	16.729281
H	8.049187	4.705385	13.922039	H	7.734628	0.542776	15.93798
C	9.247795	12.199411	15.022822	C	11.536319	8.816589	12.586702
H	9.880907	12.794107	14.360348	H	12.294737	8.901178	11.805496
C	2.621789	2.894219	15.237508	C	7.224679	8.243819	23.071823
H	2.263492	2.087289	14.595213	H	7.018078	8.776098	24.003026
C	5.625103	0.775646	18.620655	C	6.942432	8.864638	21.840561
H	5.045156	0.150926	19.303988	H	6.518773	9.871508	21.827808
C	9.469288	2.657846	20.330338	C	1.808528	3.425591	16.239492
H	9.847712	1.752946	19.849086	H	0.806969	3.019224	16.398031
C	9.440446	2.740377	21.735551	C	3.804654	7.064475	10.737645
H	9.794065	1.895975	22.331593	C	3.411568	5.599993	11.091085
C	2.256901	4.494905	17.038426	C	4.648816	6.103345	9.864677
H	1.599831	4.908299	17.807205	H	3.046767	7.665082	10.217703
C	11.000880	9.972109	13.160575	H	4.353713	7.620098	11.5071
H	11.337009	10.960905	12.840553	C	4.920207	5.228279	11.111104
C	6.321924	2.180974	12.365475	H	5.167953	4.176992	10.927075
H	6.455989	1.457634	11.558234	H	5.523656	5.674254	11.906036
C	6.375055	0.202436	17.591978	C	3.377076	5.267916	9.57315
H	6.376609	-0.881949	17.460118	H	3.534029	4.216634	9.298343
C	7.759223	6.954858	23.112892	H	2.593138	5.755354	8.978801
H	7.967315	6.466461	24.067703	C	2.398942	5.190006	12.12011
C	4.072236	5.293256	21.130973	F	2.327489	3.850476	12.226056
H	3.693595	6.155337	21.685112	F	2.716167	5.683651	13.330227
C	3.691476	3.994050	21.516862	F	1.170854	5.638699	11.807528
H	3.023195	3.859482	22.370426	S	5.831853	6.657848	8.567842
C	7.364258	3.073556	12.675208	C	7.004575	5.31739	8.497936
H	8.296997	3.027399	12.110578	C	6.695997	7.991327	9.374303
C	5.120717	2.216989	13.076461	C	7.996523	5.133553	9.478957
H	4.309950	1.524764	12.840108	C	6.851613	4.424331	7.427796
C	11.106026	7.540278	12.998818	C	7.680364	7.773378	10.352749
H	11.533698	6.650204	12.532559	C	6.282949	9.284352	9.020604

S	8.274054	6.188225	10.87536
C	8.82579	4.003875	9.369955
C	7.672539	3.300783	7.350603
H	6.08403	4.604581	6.672403
C	8.221801	8.893009	11.003904
C	6.828323	10.382033	9.684063
H	5.527742	9.423379	8.244182
C	8.653169	3.093197	8.327819
H	9.621695	3.849942	10.101802
H	7.549838	2.593974	6.528275
C	7.790304	10.178208	10.680795
H	8.988656	8.751809	11.763531
H	6.502106	11.390399	9.423556
H	9.306897	2.220446	8.269607
H	8.224108	11.03012	11.209018

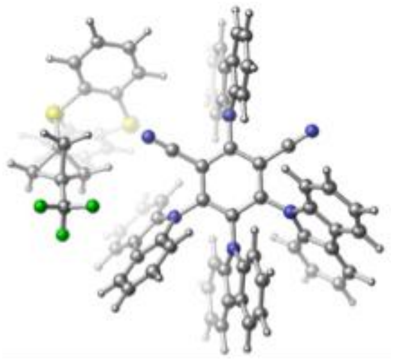

Zero-point correction= 1.004213 (Hartree/Particle)

Thermal correction to Energy= 1.069821

Thermal correction to Enthalpy= 1.070765

Thermal correction to Gibbs Free Energy= 0.900168

Sum of electronic and zero-point Energies= -4268.297879

Sum of electronic and thermal Energies= -4268.232271

Sum of electronic and thermal Enthalpies= -4268.231327

Sum of electronic and thermal Free Energies= -4268.401924

uB3LYP-D3/def2tzvpp-CPCM(DCM)

E(scf)= -2482.81874805

uM06-d3 /def2tzvpp-CPCM(DCM)

E(scf)= -2480.86816546

uM06-2X/def2tzvpp-CPCM(DCM)

E(scf)= -2481.74758393

uWb97xd/def2tzvpp-CPCM(DCM)

E(scf)= -2481.84252052

uPE1PBE/def2tzvpp-CPCM(DMSO)

E(scf)= -2479.77620560

[1]PC-B(2)

E(scf) -4269.30759656 a.u.

ν_{min} = 17.2714 cm^{-1}

N	8.260302	4.931381	12.381202	C	10.062156	9.285393	13.413174
N	10.003497	8.777927	17.857969	C	5.418247	3.151903	15.121687
N	9.521610	7.413097	14.626352	C	11.385602	5.908338	13.803103
N	7.623865	6.407181	18.987462	H	11.112639	5.043906	14.410064
N	6.371528	4.068319	17.920279	C	6.993894	7.590718	19.428060
N	6.800657	3.409973	15.188043	C	9.379306	7.916495	17.395336
C	8.192146	5.382295	14.931697	C	5.211458	4.485999	18.596073
C	8.628743	6.841311	16.819882	C	8.493554	4.347776	20.177372
C	7.190561	4.877595	17.114123	H	8.921184	3.876728	19.293233
C	7.803159	6.041749	17.645209	C	6.786903	7.500820	20.826950
C	9.151947	8.742767	14.354496	C	11.014524	8.237229	13.091117
C	7.408816	4.538881	15.757660	C	6.533157	1.392559	14.124527
C	6.588228	2.708938	18.208587	C	7.634962	1.872861	17.813411
C	8.799175	6.540281	15.453082	H	8.445314	2.230171	17.180003
C	8.243335	5.127828	13.524213	C	7.432204	5.594281	22.505896
C	10.654694	7.098023	13.855747	H	7.039027	6.078503	23.402710
C	4.686558	3.388331	19.323479	C	8.049732	9.465078	14.817765
C	7.483875	2.339799	14.577763	H	7.335053	9.028206	15.512195
C	7.841226	5.579985	20.105218	C	5.560892	2.257045	19.074552
C	7.328289	6.224363	21.258144	C	4.344048	3.945062	15.531347
C	4.564424	5.723483	18.569867	H	4.495268	4.916232	15.994744
H	4.944373	6.554498	17.979156	C	6.549167	8.690622	18.690399

H	6.697510	8.756473	17.613852	C	3.512243	3.550846	20.071472
C	7.874650	10.764308	14.338768	H	3.099978	2.712249	20.637403
H	7.010984	11.341586	14.675760	C	8.783094	11.330526	13.423851
C	5.220318	1.912871	14.460381	H	8.622046	12.350238	13.067451
C	8.858541	2.127621	14.441210	C	7.621	0.552972	18.270399
H	9.588890	2.849390	14.809430	H	8.425839	-0.120743	17.967074
C	9.875898	10.598100	12.956822	C	12.843119	6.977856	12.156023
H	10.561830	11.028166	12.225738	H	13.703153	6.907395	11.48624
C	3.916401	1.453542	14.225440	C	5.688357	9.646492	20.768288
H	3.751883	0.504820	13.709799	H	5.174693	10.464515	21.278132
C	5.567466	0.929418	19.524265	C	5.895657	9.716243	19.378236
H	4.780766	0.570567	20.192002	H	5.544457	10.587952	18.821182
C	8.580767	3.734041	21.428943	C	2.838649	2.232027	14.650957
H	9.074148	2.762638	21.509767	H	1.817256	1.887885	14.474189
C	8.049411	4.344182	22.581090	C	6.635001	8.157542	9.207441
H	8.133188	3.837748	23.545346	C	7.857757	8.777012	9.942653
C	3.054414	3.465504	15.293801	C	6.150309	8.73158	10.562353
H	2.200117	4.070167	15.606456	H	6.292156	8.673733	8.301354
C	12.117904	8.167894	12.229078	H	6.623064	7.064237	9.109267
H	12.396034	9.030924	11.621271	C	7.377133	8.090881	11.253593
C	8.341876	0.000011	13.352419	H	7.698749	8.547793	12.195223
H	8.698739	-0.913291	12.871166	H	7.423829	6.996507	11.275401
C	6.596020	0.080522	19.111785	C	6.982365	10.012547	10.299902
H	6.612909	-0.957619	19.451225	H	7.288994	10.584302	11.185006
C	6.130434	8.541432	21.498046	H	6.658692	10.646716	9.464285
H	5.961576	8.480294	22.575585	C	9.26338	8.793773	9.409995
C	3.396951	5.865407	19.323077	F	10.073164	9.558338	10.1658
H	2.881606	6.828662	19.323598	F	9.784337	7.555562	9.382843
C	2.877869	4.794528	20.074559	F	9.300996	9.282531	8.15868
H	1.964134	4.936956	20.655950	S	4.379956	8.719278	11.042815
C	9.272829	0.946937	13.820114	C	4.349401	9.213864	12.757574
H	10.342009	0.758333	13.698926	C	4.025106	6.970384	11.062879
C	6.971073	0.214302	13.504311	C	4.708019	8.337527	13.796705
H	6.247346	-0.524604	13.152804	C	3.929299	10.525887	13.017161
C	12.482505	5.864279	12.939478	C	4.45979	6.128451	12.10214
H	13.069211	4.945101	12.87244	C	3.348319	6.466987	9.943646

S	5.291575	6.682807	13.567387
C	4.643853	8.812346	15.116467
C	3.903725	10.988341	14.332442
H	3.637051	11.179799	12.19297
C	4.236628	4.747914	11.975236
C	3.138363	5.092726	9.834136
H	3.006762	7.145599	9.15939
C	4.261084	10.128063	15.377346
H	4.88757	8.138257	15.939383
H	3.594628	12.013976	14.540279
C	3.593727	4.239865	10.845845
H	4.552898	4.072919	12.770896
H	2.621731	4.691672	8.960524
H	4.2266	10.477427	16.411251
H	3.431331	3.162763	10.769383
C	-0.242570	-0.145833	0.028614
O	-1.628856	-0.015488	-0.360183
H	-1.992384	0.561478	0.333680
O	0.098794	0.439630	1.101879
O	0.093404	-1.538143	-0.083316
H	0.377356	-1.774342	0.819906

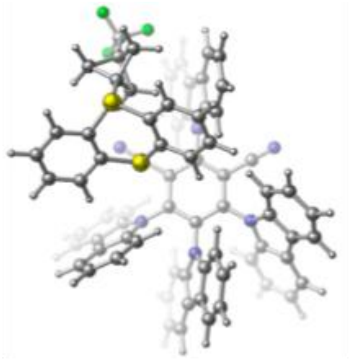

Zero-point correction= 1.004808 (Hartree/Particle)

Thermal correction to Energy= 1.070247

Thermal correction to Enthalpy= 1.071192

Thermal correction to Gibbs Free Energy= 0.901729

Sum of electronic and zero-point Energies= -4268.302789

Sum of electronic and thermal Energies= -4268.237349

Sum of electronic and thermal Enthalpies= -4268.236405

Sum of electronic and thermal Free Energies= -4268.405868

uB3LYP-D3/def2tzvpp-CPCM(DCM)

E(scf)= -4273.33778779

uM06-d3/def2tzvpp-CPCM(DCM)

E(scf)= -4270.66591433

uM06-2X/def2tzvpp-CPCM(DCM)

E(scf)= -4271.78024924

uWb97xd/def2tzvpp-CPCM(DCM)

E(scf)= -4271.97642688

uPE1PBE/def2tzvpp-CPCM(DMSO)

E(scf)= -4268.96820913

[1]PC-B(3)

E(scf) -4269.30209203 a.u.

ν_{min} = 13.4194 cm^{-1}

N	6.224002	7.551510	-13.258129	C	9.244901	10.814831	-14.950410
N	10.175058	8.760579	-18.745543	C	4.356396	4.464614	-15.882483
N	8.620347	8.697924	-15.567893	C	10.130628	7.404458	-14.004183
N	8.015127	6.076750	-19.629413	H	9.812489	6.413830	-14.332345
N	6.242711	4.325699	-18.244991	C	7.700281	6.966460	-20.677668
N	5.655412	4.844828	-15.496520	C	9.339653	8.136139	-18.238141
C	7.087895	6.803109	-15.581326	C	5.396780	4.394140	-19.365650
C	8.320744	7.363262	-17.592702	C	9.023022	3.755123	-19.553208
C	6.822134	5.422253	-17.584417	H	9.070341	3.686399	-18.467344
C	7.706068	6.287475	-18.277603	C	7.980427	6.329243	-21.912098
C	8.410915	10.062439	-15.817144	C	9.998432	9.862161	-14.153521
C	6.523530	5.675127	-16.221667	C	4.995100	3.134604	-14.110933
C	6.399226	2.975520	-17.887169	C	7.186635	2.409339	-16.882339
C	8.010252	7.629314	-16.247876	H	7.797492	3.021948	-16.221151
C	6.627736	7.193336	-14.284298	C	8.963116	3.946929	-22.392338
C	9.584540	8.565592	-14.557422	H	8.955521	4.025514	-23.481908
C	5.012864	3.078245	-19.727496	C	7.534733	10.669330	-16.720486
C	6.043859	4.043199	-14.401042	H	6.888853	10.077667	-17.371652
C	8.515719	4.888475	-20.191340	C	5.646925	2.176014	-18.784074
C	8.500605	5.009249	-21.603303	C	3.528372	5.025234	-16.856083
C	4.905312	5.513897	-20.040200	H	3.854310	5.868227	-17.464320
H	5.165996	6.526157	-19.735015	C	7.144375	8.247006	-20.618907

H	6.909938	8.727915	-19.668628	C	4.148578	2.883057	-20.813691
C	7.512250	12.065764	-16.753843	H	3.846239	1.8736	-21.102102
H	6.839493	12.570298	-17.451370	C	8.334834	12.832184	-15.904983
C	3.923984	3.402439	-15.052484	H	8.290606	13.922459	-15.955625
C	7.252613	4.019094	-13.702049	C	7.177578	1.018437	-16.755317
H	8.064115	4.708556	-13.933141	H	7.777789	0.554768	-15.968971
C	9.200492	12.215575	-14.999788	C	11.531084	8.835589	-12.599752
H	9.833501	12.811094	-14.337957	H	12.293971	8.92121	-11.823023
C	2.644647	2.861667	-15.231823	C	7.16509	8.28342	-23.063802
H	2.295381	2.049500	-14.591153	H	6.949259	8.817912	-23.991637
C	5.654658	0.781575	-18.641386	C	6.88348	8.896369	-21.828465
H	5.077769	0.154978	-19.325580	H	6.45115	9.899446	-21.809224
C	9.472599	2.705765	-20.357830	C	1.822879	3.390758	-16.228058
H	9.861413	1.802190	-19.882405	H	0.823719	2.977435	-16.383728
C	9.436504	2.794289	-21.762518	C	3.817096	7.024464	-10.721539
H	9.795012	1.955799	-22.363961	C	3.432844	5.558455	-11.078375
C	2.259591	4.466509	-17.024792	C	4.671438	6.066349	-9.855235
H	1.596003	4.877886	-17.789043	H	3.057097	7.617936	-10.196532
C	10.983235	9.990467	-13.163119	H	4.35919	7.58649	-11.491255
H	11.313967	10.979836	-12.839266	C	4.943945	5.197272	-11.105595
C	6.362661	2.165171	-12.379041	H	5.199593	4.147104	-10.926069
H	6.505684	1.439721	-11.575238	H	5.541248	5.649964	-11.901378
C	6.414456	0.210129	-17.618979	C	3.406701	5.221074	-9.561458
H	6.426887	-0.874867	-17.492856	H	3.572147	4.170013	-9.29076
C	7.710742	6.999416	-23.113122	H	2.621752	5.701046	-8.962377
H	7.918453	6.517032	-24.071068	C	2.419252	5.144973	-12.105051
C	4.047989	5.297020	-21.121356	F	2.35669	3.805368	-12.215429
H	3.658777	6.158296	-21.669390	F	2.72864	5.645046	-13.314592
C	3.677309	3.996280	-21.511857	F	1.189232	5.58405	-11.786476
H	3.006124	3.859764	-22.362829	S	5.855887	6.624861	-8.561412
C	7.396917	3.066702	-12.689886	C	7.036781	5.291341	-8.499291
H	8.332483	3.025243	-12.129561	C	6.709167	7.965477	-9.367711
C	5.158099	2.194952	-13.084614	C	8.026283	5.115683	-9.484278
H	4.353546	1.495805	-12.847412	C	6.892654	4.394752	-7.43094
C	11.107632	7.558548	-13.016703	C	7.691802	7.75591	-10.349717
H	11.545022	6.668945	-12.558615	C	6.28952	9.255108	-9.009503

S	8.293089	6.17575	-10.8787
C	8.862292	3.990458	-9.381107
C	7.720221	3.275698	-7.359482
H	6.126657	4.568689	-6.672454
C	8.224707	8.880404	-10.999468
C	6.826275	10.357728	-9.671821
H	5.535886	9.387597	-8.230429
C	8.698525	3.076164	-8.340685
H	9.656473	3.84295	-10.116128
H	7.604435	2.566144	-6.538515
C	7.786525	10.162191	-10.671864
H	8.990158	8.745624	-11.761648
H	6.494784	11.363453	-9.407789
H	9.357385	2.206985	-8.286984
H	8.213686	11.017973	-11.199244
C	-0.242570	-0.145833	0.028614
O	-1.628856	-0.015488	-0.360183
H	-1.992384	0.561478	0.333680
O	0.098794	0.439630	1.101879
O	0.093404	-1.538143	-0.083316
H	0.377356	-1.774342	0.819906

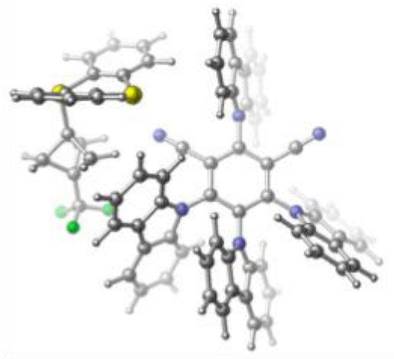

Zero-point correction= 1.004193 (Hartree/Particle)

Thermal correction to Energy= 1.069812

Thermal correction to Enthalpy= 1.070756

Thermal correction to Gibbs Free Energy= 0.900053

Sum of electronic and zero-point Energies= -4268.297899

Sum of electronic and thermal Energies= -4268.232280

Sum of electronic and thermal Enthalpies= -4268.231336

Sum of electronic and thermal Free Energies= -4268.402039

uB3LYP-D3/def2tzvpp-CPCM(DCM)

E(scf)= -4273.33221648

uM06-d3/def2tzvpp-CPCM(DCM)

E(scf)= -4270.66109917

uM06-2X/def2tzvpp-CPCM(DCM)

E(scf)= -4271.77965451

uWb97xd/def2tzvpp-CPCM(DCM)

E(scf)= -4271.97267976

uPE1PBE/def2tzvpp-CPCM(DMSO)

E(scf)= -4268.96337986

[1]PC-B(4)

E(scf) -4269.30795784 a.u.

ν_{min} = 17.3251 cm^{-1}

N	8.226912	5.131117	-12.316907	C	10.122705	9.423377	-13.485698
N	9.967882	8.830781	-17.883552	C	5.402388	3.205176	-15.068434
N	9.496157	7.530378	-14.620700	C	11.365597	6.012429	-13.830854
N	7.605093	6.426507	-18.962897	H	11.049842	5.134597	-14.396416
N	6.371665	4.099947	-17.853566	C	6.971375	7.594422	-19.437764
N	6.776672	3.514314	-15.095422	C	9.341556	7.982087	-17.400767
C	8.144767	5.509390	-14.878640	C	5.219309	4.482914	-18.562227
C	8.588934	6.922082	-16.800427	C	8.509815	4.349869	-20.095505
C	7.165380	4.941228	-17.055102	H	8.931881	3.903197	-19.196240
C	7.773159	6.096816	-17.609881	C	6.782324	7.471277	-20.836644
C	9.170299	8.878074	-14.382794	C	11.071100	8.368130	-13.178096
C	7.370922	4.639077	-15.687074	C	6.547493	1.507006	-14.001348
C	6.613305	2.736842	-18.101631	C	7.664519	1.927302	-17.665142
C	8.755954	6.653108	-15.427121	H	8.458128	2.313492	-17.027358
C	8.201008	5.292634	-13.465106	C	7.464762	5.533171	-22.463994
C	10.655877	7.214441	-13.889223	H	7.078069	5.993269	-23.376193
C	4.723842	3.358804	-19.269523	C	8.081265	9.619394	-14.846000
C	7.478220	2.477049	-14.446769	H	7.335756	9.182392	-15.508167
C	7.844304	5.576576	-20.058765	C	5.608975	2.247262	-18.974015
C	7.339665	6.190116	-21.232137	C	4.312226	3.942110	-15.536929
C	4.553771	5.710793	-18.579146	H	4.439135	4.908316	-16.016845
H	4.910198	6.562142	-18.002495	C	6.510751	8.707358	-18.730045

H	6.646439	8.797448	-17.653310	C	3.561118	3.484037	-20.04227
C	7.959528	10.938660	-14.405455	H	3.171206	2.62481	-20.592902
H	7.109039	11.534065	-14.744427	C	8.899835	11.501455	-13.52033
C	5.228576	1.972850	-14.388382	H	8.773764	12.53407	-13.187742
C	8.855140	2.307788	-14.275805	C	7.677563	0.595516	-18.086419
H	9.573433	3.042221	-14.642063	H	8.48604	-0.058062	-17.750481
C	9.980538	10.749940	-13.054504	C	12.922199	7.106672	-12.294278
H	10.696292	11.178736	-12.350484	H	13.813738	7.041593	-11.666421
C	3.936759	1.463693	-14.192747	C	5.668954	9.609935	-20.8395
H	3.792067	0.519485	-13.663113	H	5.156138	10.412766	-21.373787
C	5.642682	0.908355	-19.387451	C	5.859271	9.712733	-19.449012
H	4.873925	0.520401	-20.059781	H	5.495742	10.594707	-18.916601
C	8.618280	3.709185	-21.331827	C	2.845037	2.185159	-14.67888
H	9.122371	2.741373	-21.384888	H	1.832765	1.801171	-14.535054
C	8.094797	4.287834	-22.503641	C	6.839073	7.318742	-9.915488
H	8.195090	3.760743	-23.455167	C	7.923037	8.413452	-10.141825
C	3.035191	3.412285	-15.341388	C	6.174664	8.534972	-10.603593
H	2.170187	3.972863	-15.702806	H	6.548343	7.122578	-8.875355
C	12.215117	8.306708	-12.371604	H	6.939762	6.408287	-10.518363
H	12.538256	9.184090	-11.807757	C	7.370876	8.628192	-11.577887
C	8.378011	0.185811	-13.158748	H	7.555563	9.606815	-12.032772
H	8.750768	-0.706999	-12.651807	H	7.498946	7.797196	-12.276683
C	6.675109	0.085840	-18.933174	C	6.918535	9.481741	-9.628447
H	6.712849	-0.960693	-19.243952	H	7.080604	10.511131	-9.973692
C	6.126845	8.491741	-21.538907	H	6.632993	9.421962	-8.570059
H	5.971435	8.405340	-22.616751	C	9.373256	8.291381	-9.772125
C	3.398218	5.815225	-19.356640	F	10.066451	9.38559	-10.138666
H	2.868423	6.769964	-19.391391	F	9.935268	7.229214	-10.372206
C	2.908815	4.717545	-20.089148	F	9.526883	8.139642	-8.445085
H	2.003721	4.830980	-20.690100	S	4.370528	8.679623	-11.000212
C	9.290371	1.151908	-13.623219	C	4.351519	9.195785	-12.708599
H	10.361740	0.998556	-13.474937	C	3.854982	6.972001	-11.040773
C	7.005634	0.354470	-13.348454	C	4.65753	8.306332	-13.753925
H	6.296354	-0.400882	-13.002631	C	4.022433	10.534963	-12.956848
C	12.502113	5.974841	-13.019520	C	4.222185	6.107432	-12.087181
H	13.074455	5.046792	-12.950169	C	3.126754	6.51818	-9.933251

S	5.116659	6.610882	-13.533234
C	4.638676	8.796395	-15.069615
C	4.034102	11.008483	-14.268562
H	3.771719	11.199984	-12.12805
C	3.879124	4.750246	-11.980138
C	2.794297	5.166496	-9.844189
H	2.841534	7.214644	-9.142189
C	4.342674	10.136661	-15.319364
H	4.84684	8.115087	-15.896555
H	3.794228	12.053922	-14.469155
C	3.182798	4.288431	-10.862231
H	4.145445	4.059077	-12.780274
H	2.236559	4.801929	-8.979869
H	4.338276	10.498739	-16.349401
H	2.926468	3.228813	-10.799297
C	-0.242570	-0.145833	0.028614
O	-1.628856	-0.015488	-0.360183
H	-1.992384	0.561478	0.333680
O	0.098794	0.439630	1.101879
O	0.093404	-1.538143	-0.083316
H	0.377356	-1.774342	0.819906

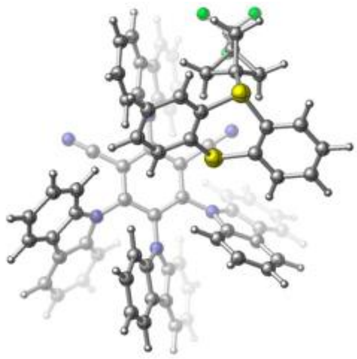

Zero-point correction= 1.004823 (Hartree/Particle)

Thermal correction to Energy= 1.070148

Thermal correction to Enthalpy= 1.071092

Thermal correction to Gibbs Free Energy= 0.902695

Sum of electronic and zero-point Energies= -4268.303135

Sum of electronic and thermal Energies= -4268.237810

Sum of electronic and thermal Enthalpies= -4268.236866

Sum of electronic and thermal Free Energies= -4268.405263

uB3LYP-D3/def2tzvpp-CPCM(DCM)

E(scf)= -4273.33689582

uM06-d3/def2tzvpp-CPCM(DCM)

E(scf)= -4270.66560086

uM06-2X/def2tzvpp-CPCM(DCM)

E(scf)= -4271.80563390

uWb97xd/def2tzvpp-CPCM(DCM)

E(scf)= -4271.97587848

uPE1PBE/def2tzvpp-CPCM(DMSO)

E(scf)= -4268.96741063

[1]PC-B(5)

E(scf) -4269.30131423 a.u.

ν_{min} = 1.2825 cm^{-1}

N	5.104890	7.728110	13.704930	C	7.838780	11.255930	15.065020
N	10.005460	8.993390	18.389940	C	3.981810	4.467050	16.469210
N	7.776280	9.037750	15.644890	C	8.751900	8.087450	13.510180
N	8.303380	6.071070	19.468740	H	8.712090	7.042700	13.821630
N	6.385260	4.276220	18.360650	C	8.179060	6.850340	20.637280
N	5.165380	4.931880	15.865600	C	9.126910	8.334050	18.017200
C	6.454860	6.987100	15.788670	C	5.839440	4.188740	19.653450
C	8.048740	7.521970	17.538470	C	9.425280	3.859620	18.955280
C	6.725440	5.462460	17.688580	H	9.218290	3.870950	17.886040
C	7.680530	6.350860	18.243450	C	8.788880	6.156490	21.711880
C	7.490350	10.338870	16.090210	C	8.381050	10.478450	13.964840
C	6.120570	5.778400	16.446350	C	4.189070	3.446160	14.409990
C	6.545990	2.973340	17.856830	C	7.096390	2.551790	16.644350
C	7.432550	7.852190	16.317740	H	7.476590	3.260980	15.910710
C	5.715230	7.387310	14.630520	C	10.024860	3.844980	21.737920
C	8.313000	9.115670	14.348330	H	10.268770	3.845490	22.802780
C	5.653680	2.821580	19.978530	C	6.915120	10.759970	17.291870
C	5.292730	4.313090	14.604080	H	6.671030	10.053080	18.085160
C	9.005810	4.895930	19.791570	C	6.100250	2.048420	18.834310
C	9.315700	4.914780	21.174380	C	3.408120	4.827400	17.688860
C	5.443010	5.209630	20.520260	H	3.872580	5.564120	18.339670
H	5.543230	6.259750	20.250760	C	7.526800	8.071510	20.825060

H	7.032870	8.589150	20.002530	C	5.098540	2.473850	21.217840
C	6.654620	12.124530	17.444530	H	4.951790	1.423520	21.480110
H	6.195870	12.477090	18.370870	C	6.971130	13.046390	16.428860
C	3.353990	3.546710	15.593190	H	6.752440	14.106140	16.576860
C	6.320810	4.416180	13.663230	C	7.158640	1.177620	16.401560
H	7.183170	5.063510	13.824320	H	7.576910	0.825610	15.455630
C	7.570160	12.621060	15.241110	C	9.317210	9.794630	11.852510
H	7.817340	13.338140	14.455060	H	9.712600	10.041380	10.864770
C	2.140020	2.953350	15.966400	C	8.122060	7.938180	23.192410
H	1.645830	2.243820	15.298870	H	8.091160	8.382690	24.189690
C	6.175490	0.673490	18.572040	C	7.509650	8.606560	22.115680
H	5.834650	-0.048600	19.317660	H	7.007600	9.561480	22.287370
C	10.124550	2.800400	19.538310	C	1.575730	3.286560	17.199160
H	10.451760	1.970360	18.907760	H	0.633010	2.828300	17.506430
C	10.415610	2.786360	20.915760	C	2.883000	13.106290	16.470890
H	10.963740	1.942730	21.341370	C	2.266910	14.017190	15.371150
C	2.203930	4.217970	18.047240	C	3.216970	12.492460	15.088010
H	1.742950	4.478150	19.002960	H	3.726010	13.519440	17.039400
C	8.891950	10.815820	12.703030	H	2.177560	12.545760	17.097910
H	8.950780	11.861410	12.392020	C	1.755360	12.741690	14.641380
C	5.119620	2.793330	12.284590	H	1.579800	12.828860	13.562030
H	5.067620	2.205970	11.365140	H	0.979490	12.158730	15.153140
C	6.697550	0.245300	17.350150	C	3.598730	13.903800	14.575590
H	6.758870	-0.822860	17.129870	H	3.539670	14.063600	13.491170
C	8.759960	6.711620	22.998480	H	4.485450	14.365380	15.028600
H	9.223130	6.184250	23.835640	C	1.481680	15.275740	15.606040
C	4.894050	4.841590	21.750770	F	1.090160	15.827410	14.444850
H	4.585950	5.624020	22.448220	F	0.379720	15.030100	16.335090
C	4.729920	3.488610	22.102960	F	2.214230	16.188750	16.266580
H	4.299200	3.232790	23.073670	S	4.274610	11.018820	14.861910
C	6.216820	3.647840	12.501000	C	4.229040	10.765760	13.096760
H	7.006930	3.714070	11.749530	C	3.284410	9.704820	15.553870
C	4.104580	2.684370	13.236540	C	3.138210	10.170850	12.441330
H	3.259410	2.010690	13.077510	C	5.354990	11.207910	12.392740
C	9.248850	8.446610	12.255380	C	2.213810	9.115770	14.860380
H	9.593880	7.663170	11.576710	C	3.636470	9.291350	16.845400

S	1.668840	9.579670	13.238220	H	0.667640	7.609270	14.977460
C	3.194890	10.048440	11.043900	H	3.164420	7.975130	18.482120
C	5.391270	11.084400	11.005200	H	4.328480	10.399510	9.249500
H	6.190730	11.651840	12.930620	H	1.256440	6.897500	17.273100
C	1.490010	8.098160	15.504140	C	-0.242570	-0.145833	0.028614
C	2.896970	8.291780	17.472870	O	-1.628856	-0.015488	-0.360183
H	4.477230	9.761210	17.355080	H	-1.992384	0.561478	0.333680
C	4.306290	10.508440	10.335880	O	0.098794	0.439630	1.101879
H	2.366470	9.574570	10.512960	O	0.093404	-1.538143	-0.083316
H	6.267440	11.430870	10.454800	H	0.377356	-1.774342	0.819906
C	1.824630	7.698430	16.797640				

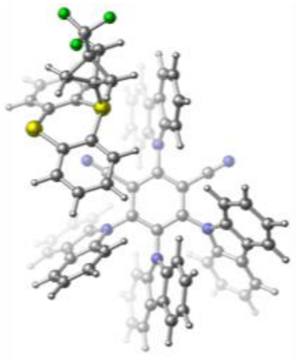

Zero-point correction= 1.004046 (Hartree/Particle)

Thermal correction to Energy= 1.069821

Thermal correction to Enthalpy= 1.070765

Thermal correction to Gibbs Free Energy= 0.895222

Sum of electronic and zero-point Energies= -4268.297269

Sum of electronic and thermal Energies= -4268.231493

Sum of electronic and thermal Enthalpies= -4268.230549

Sum of electronic and thermal Free Energies= -4268.406092

uB3LYP-D3/def2tzvpp-CPCM(DCM)

E(scf)= -4273.33552521

uM06-d3/def2tzvpp-CPCM(DCM)

E(scf)= -4270.66149986

uM06-2X/def2tzvpp-CPCM(DCM)

E(scf)= -4271.77981828

uWb97xd/def2tzvpp-CPCM(DCM)

E(scf)= -4271.97504974

uPE1PBE/def2tzvpp-CPCM(DMSO)

E(scf)= -4268.96617390

8. NMR Data

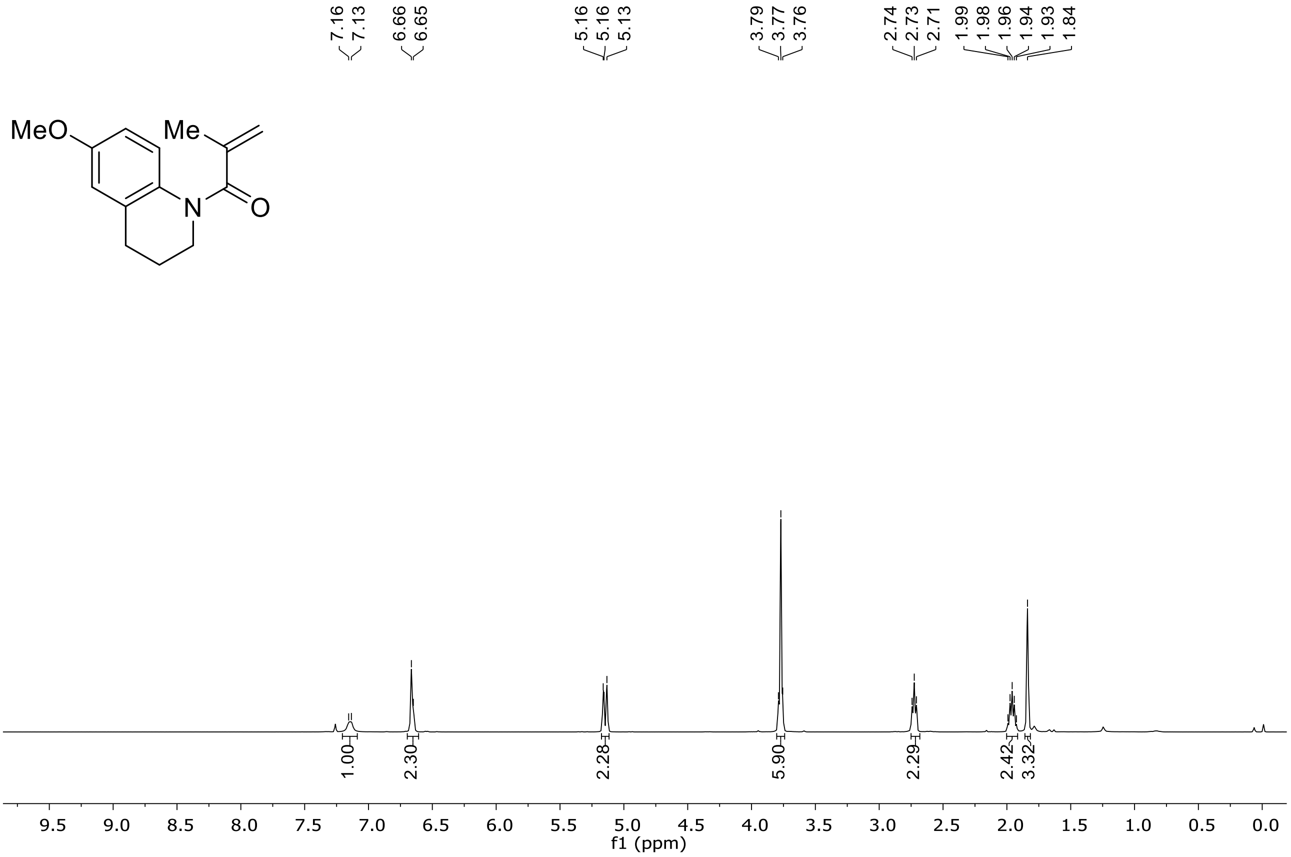

^{1}H NMR ($CDCl_3$, 400 MHz) of compound **2s.**

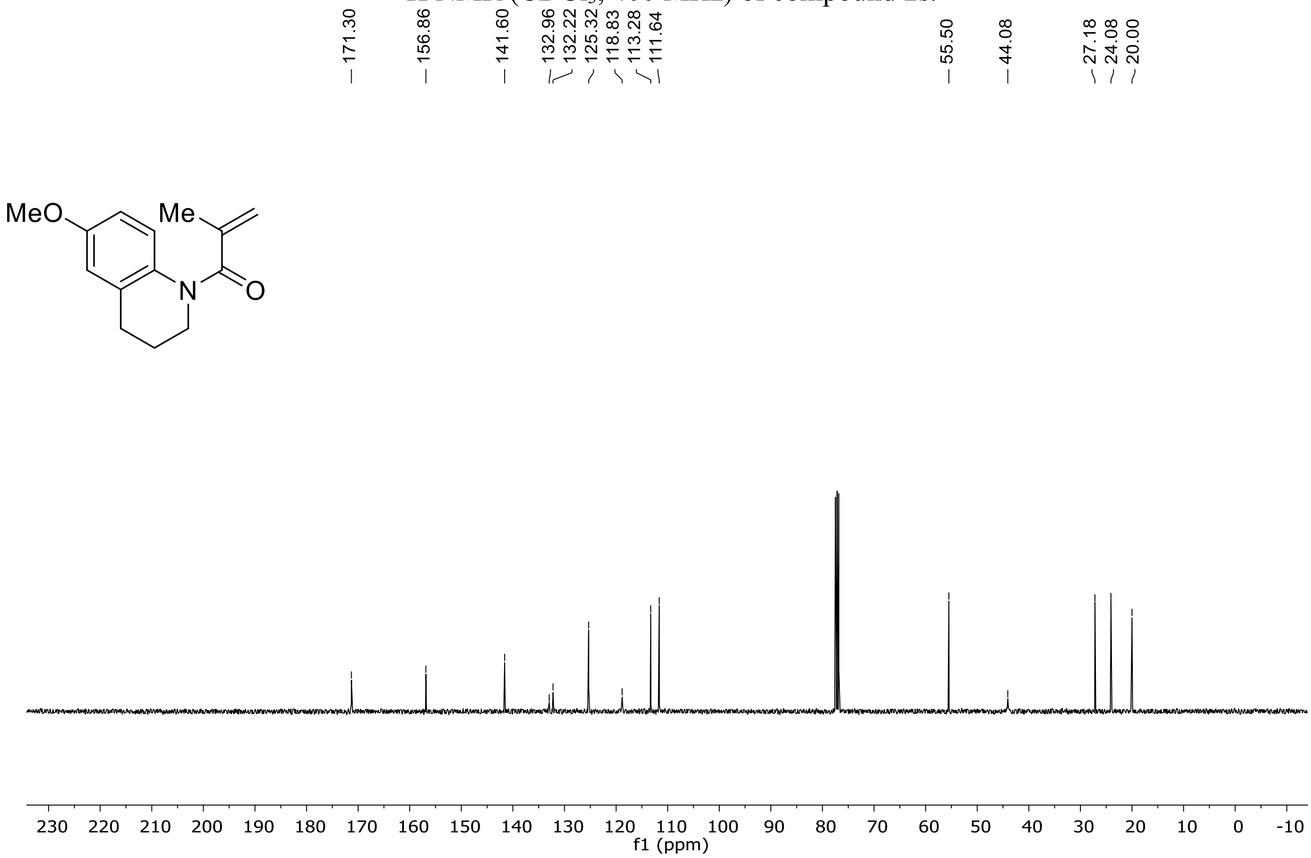

^{13}C NMR ($CDCl_3$, 101 MHz) of compound **2s.**

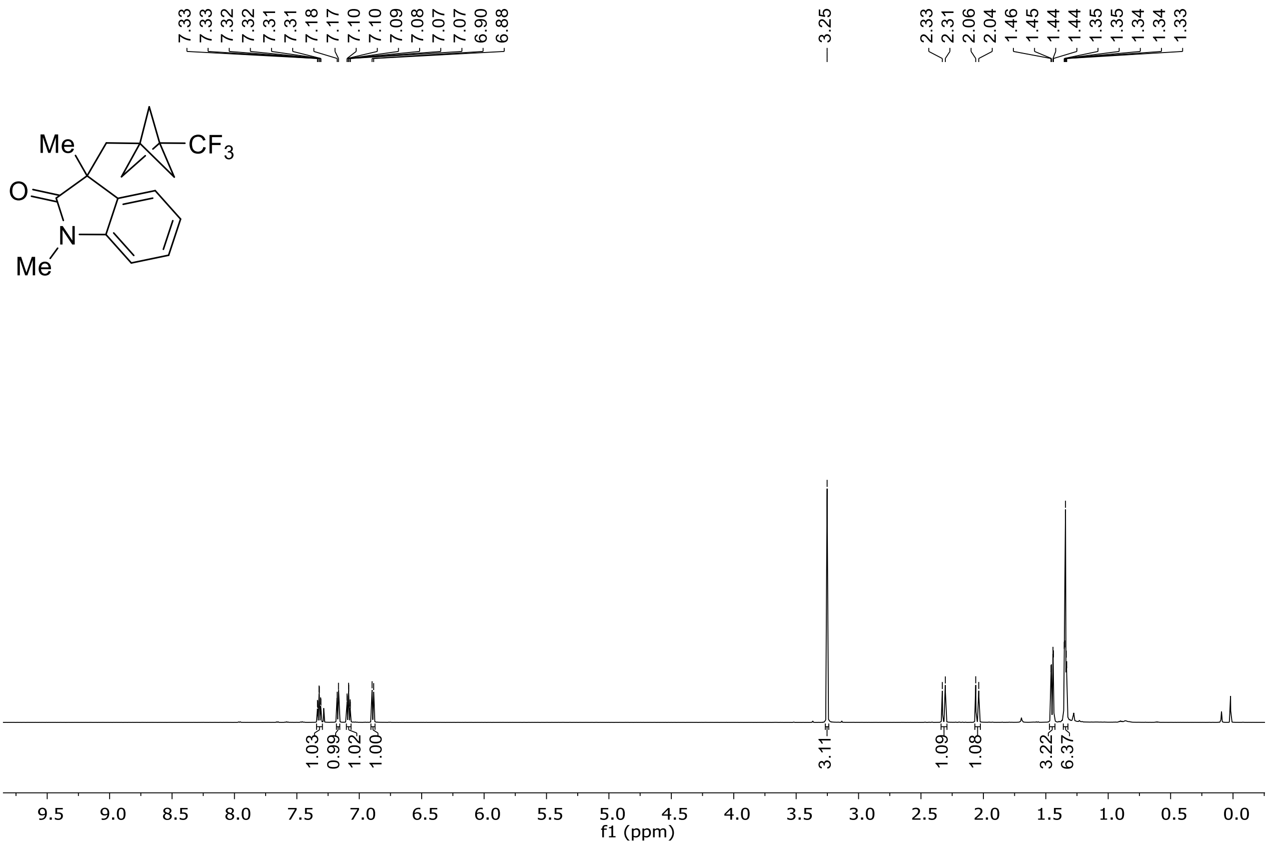

^{1}H NMR ($CDCl_3$, 600 MHz) of compound **3.**

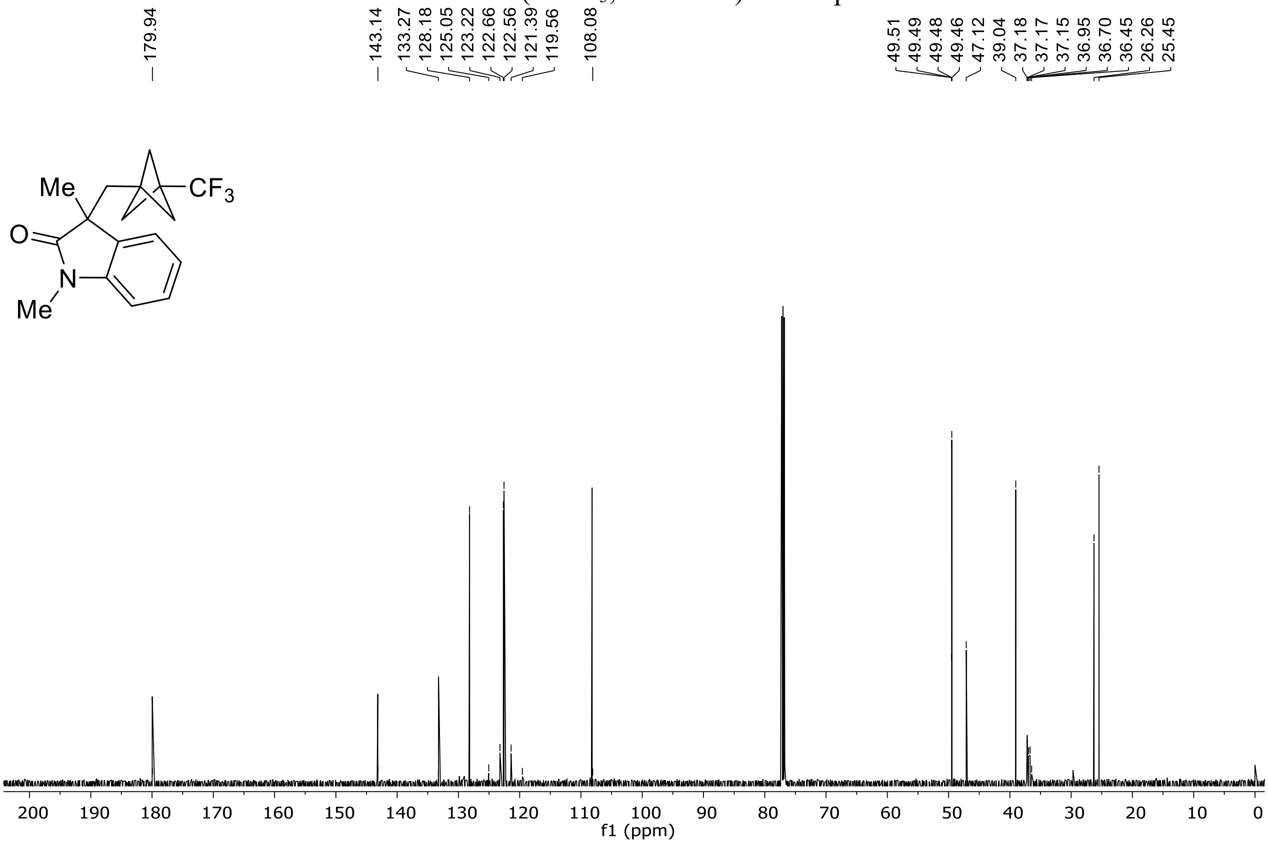

^{13}C NMR ($CDCl_3$, 126 MHz) of compound **3.**

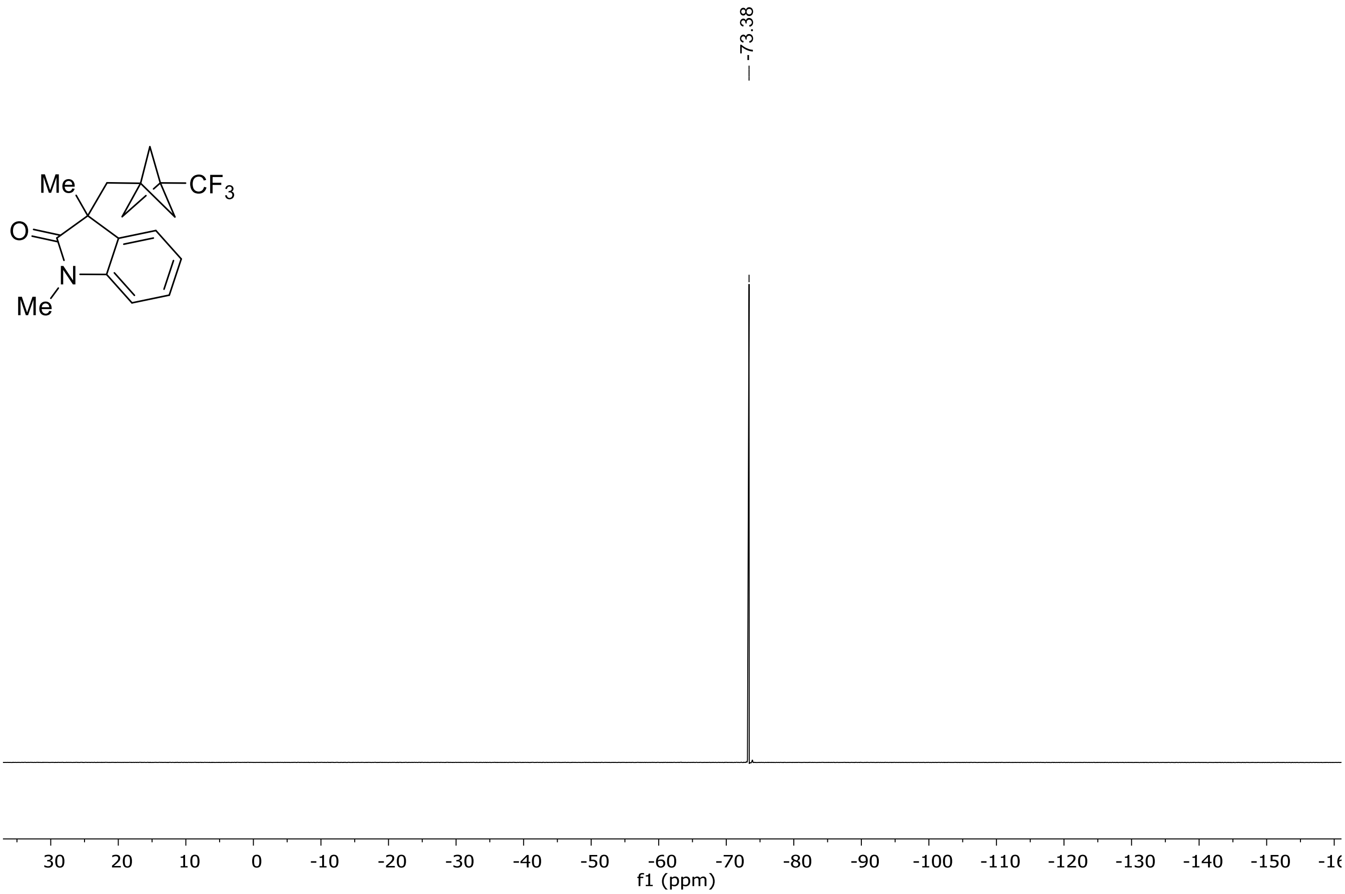

^{19}F NMR ($CDCl_3$, 376 MHz) of compound **3.**

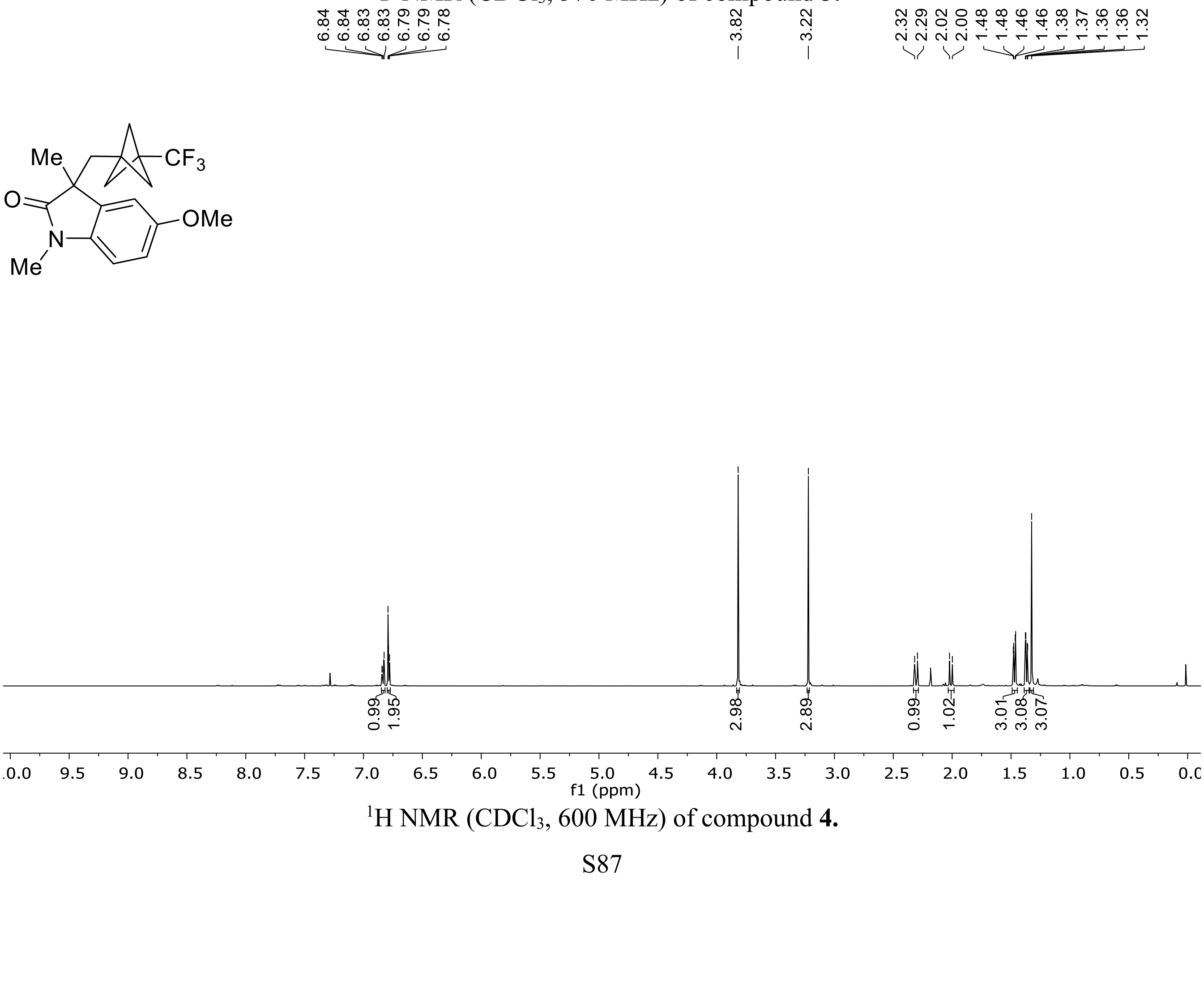

^{1}H NMR ($CDCl_3$, 600 MHz) of compound **4.**

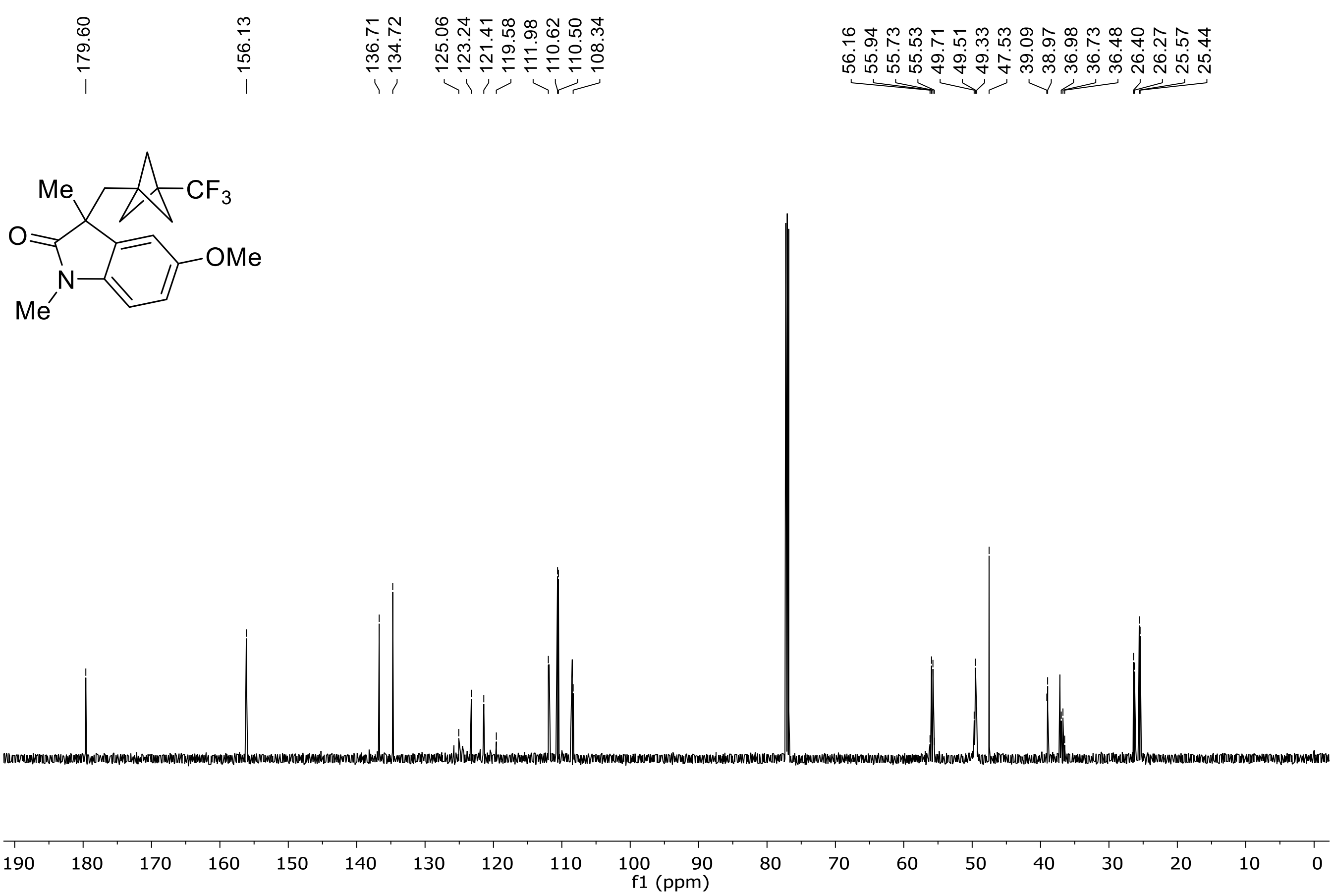

^{13}C NMR ($CDCl_3$, 151 MHz) of compound **4.**

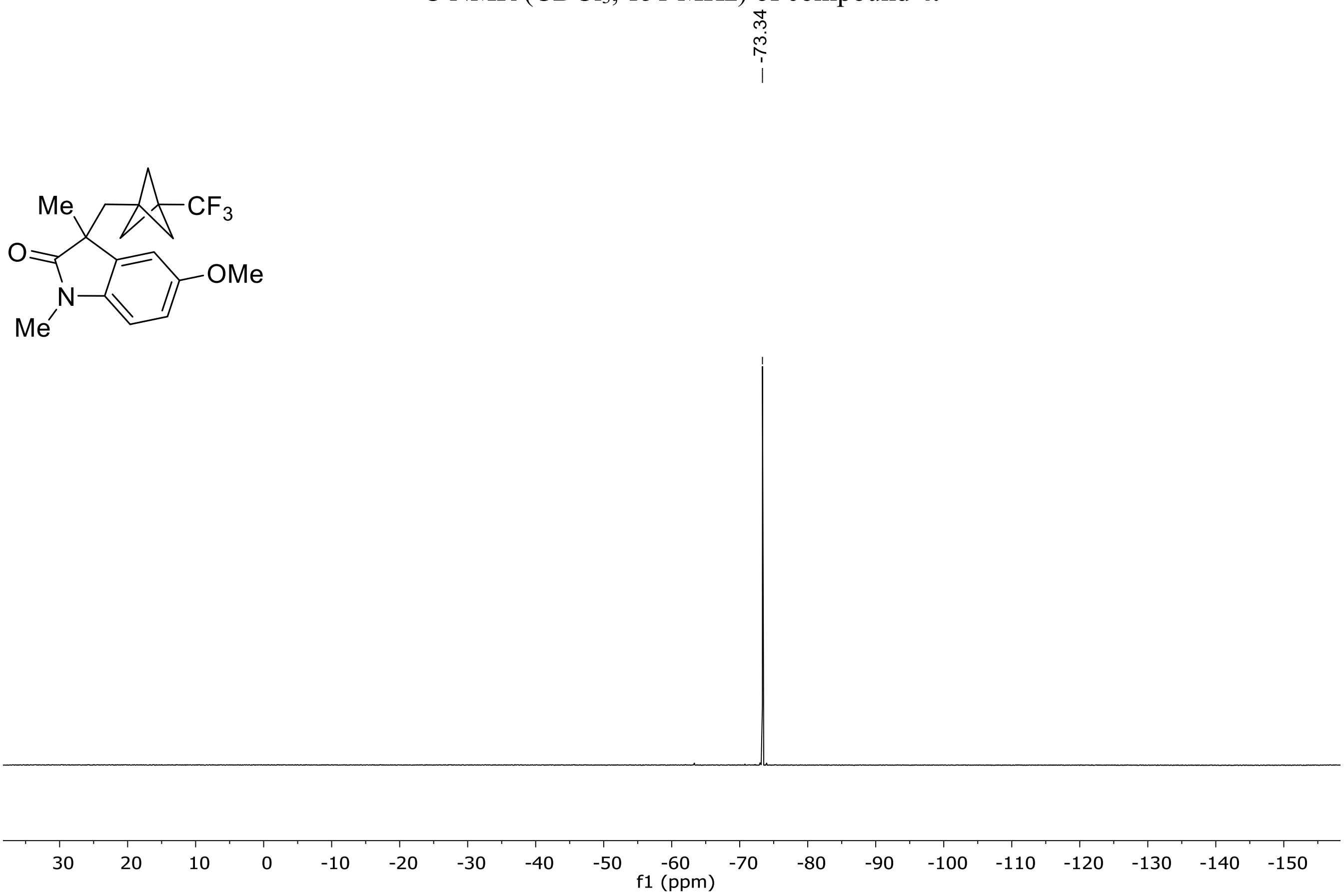

^{19}F NMR ($CDCl_3$, 376 MHz) of compound **4.**

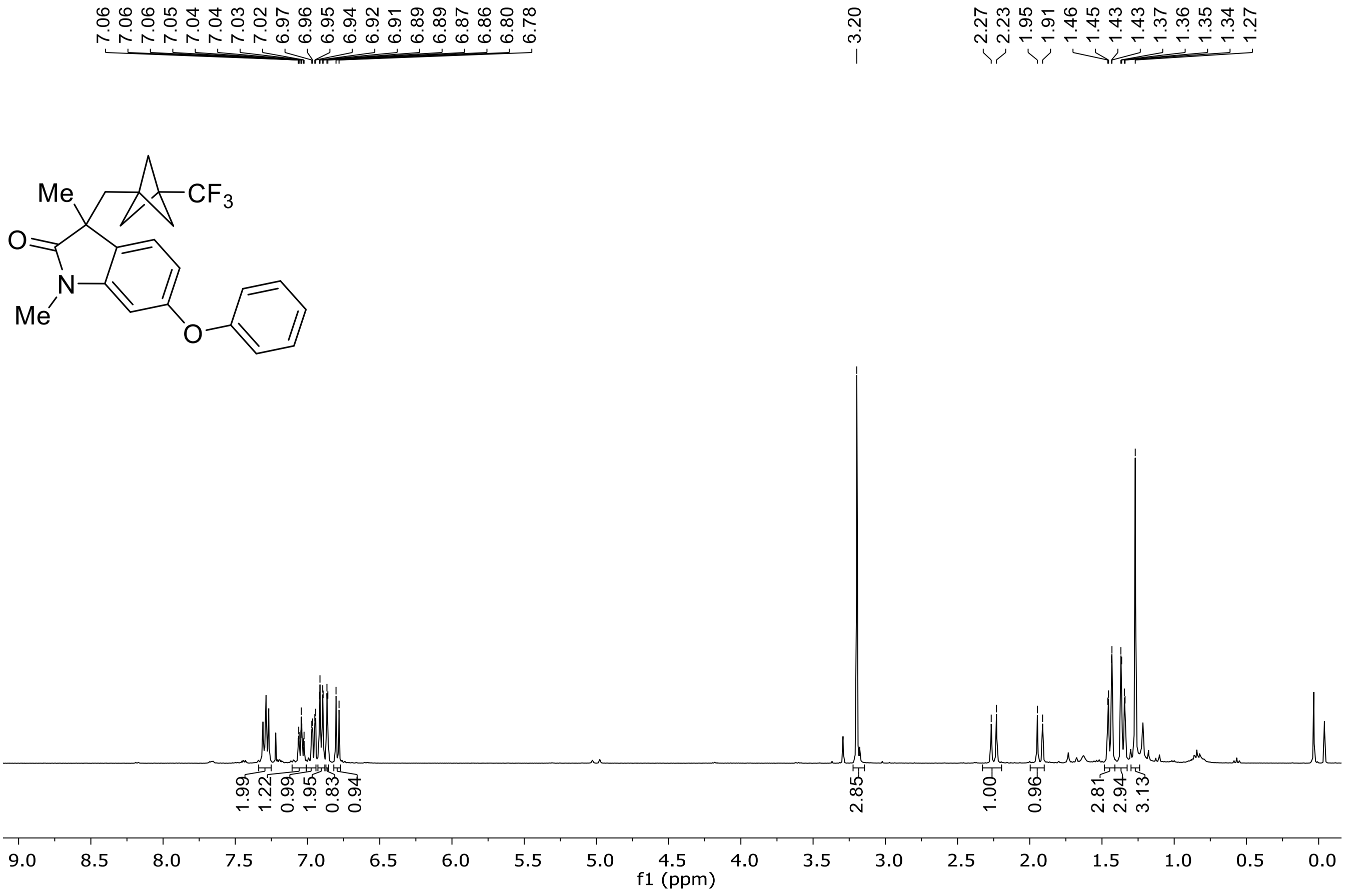

^{1}H NMR ($CDCl_3$, 400 MHz) of compound **5.**

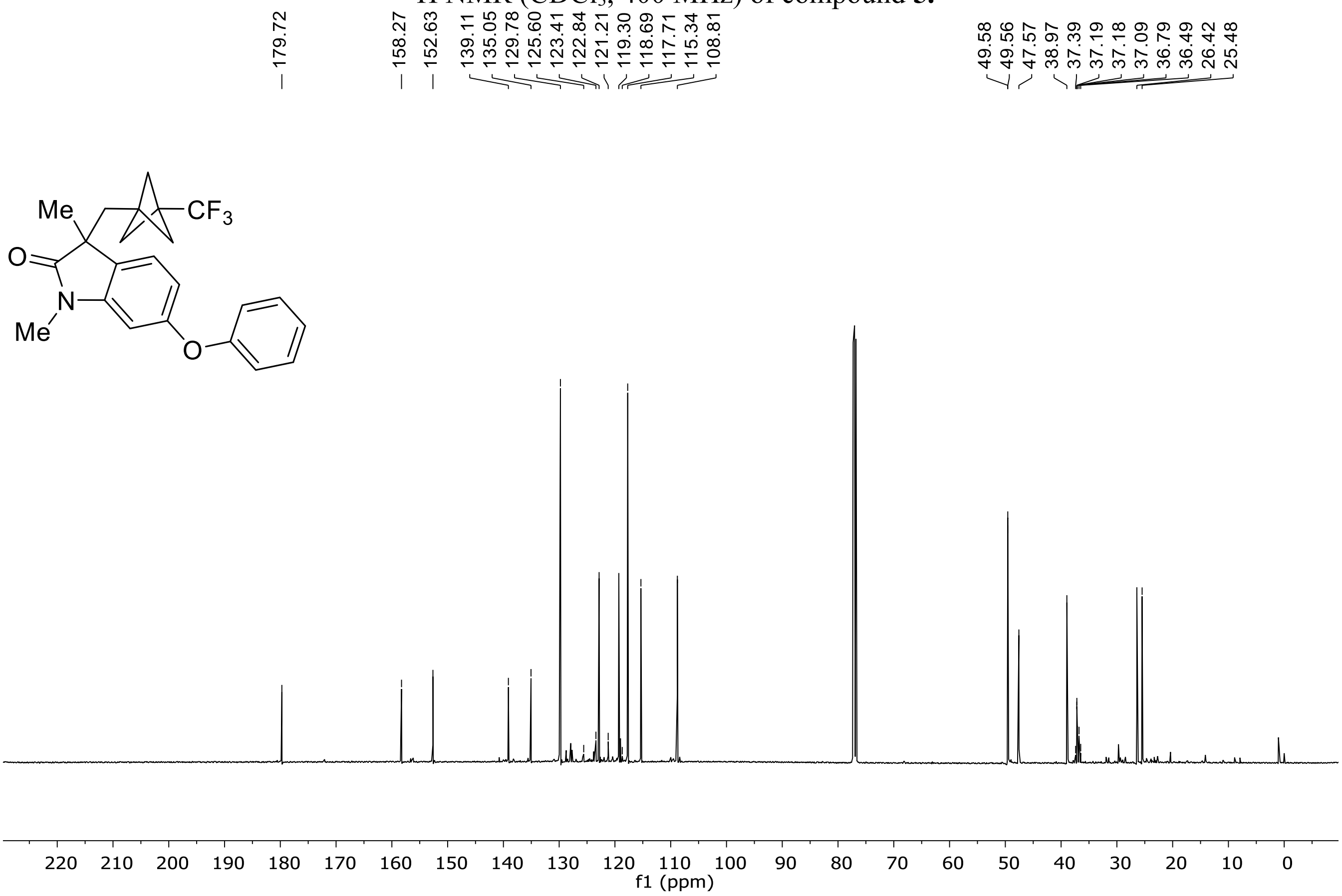

^{13}C NMR ($CDCl_3$, 126 MHz) of compound **5.**

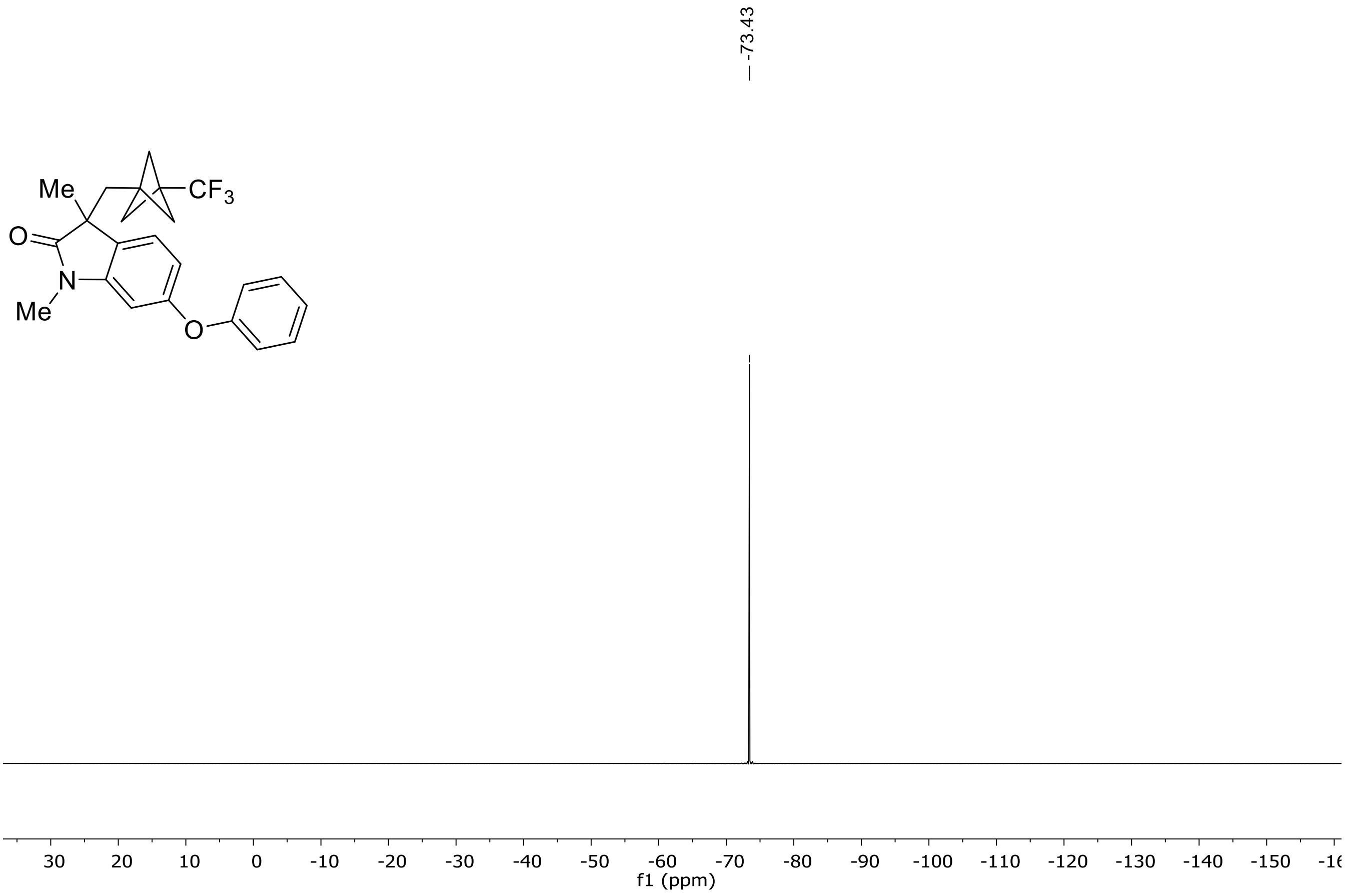

^{19}F NMR (CDCl$_3$, 282 MHz) of compound **5**.

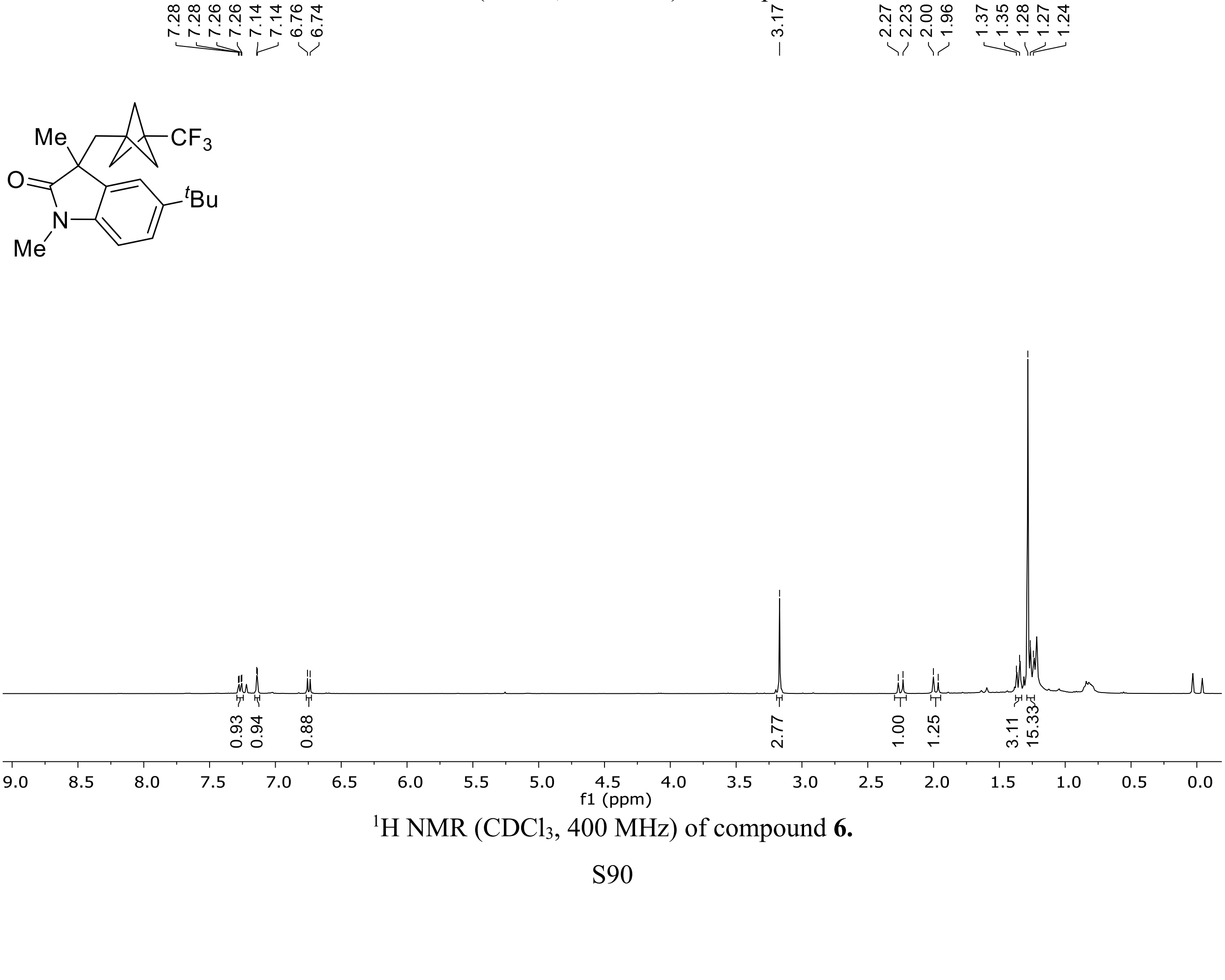

^{1}H NMR (CDCl$_3$, 400 MHz) of compound **6.**

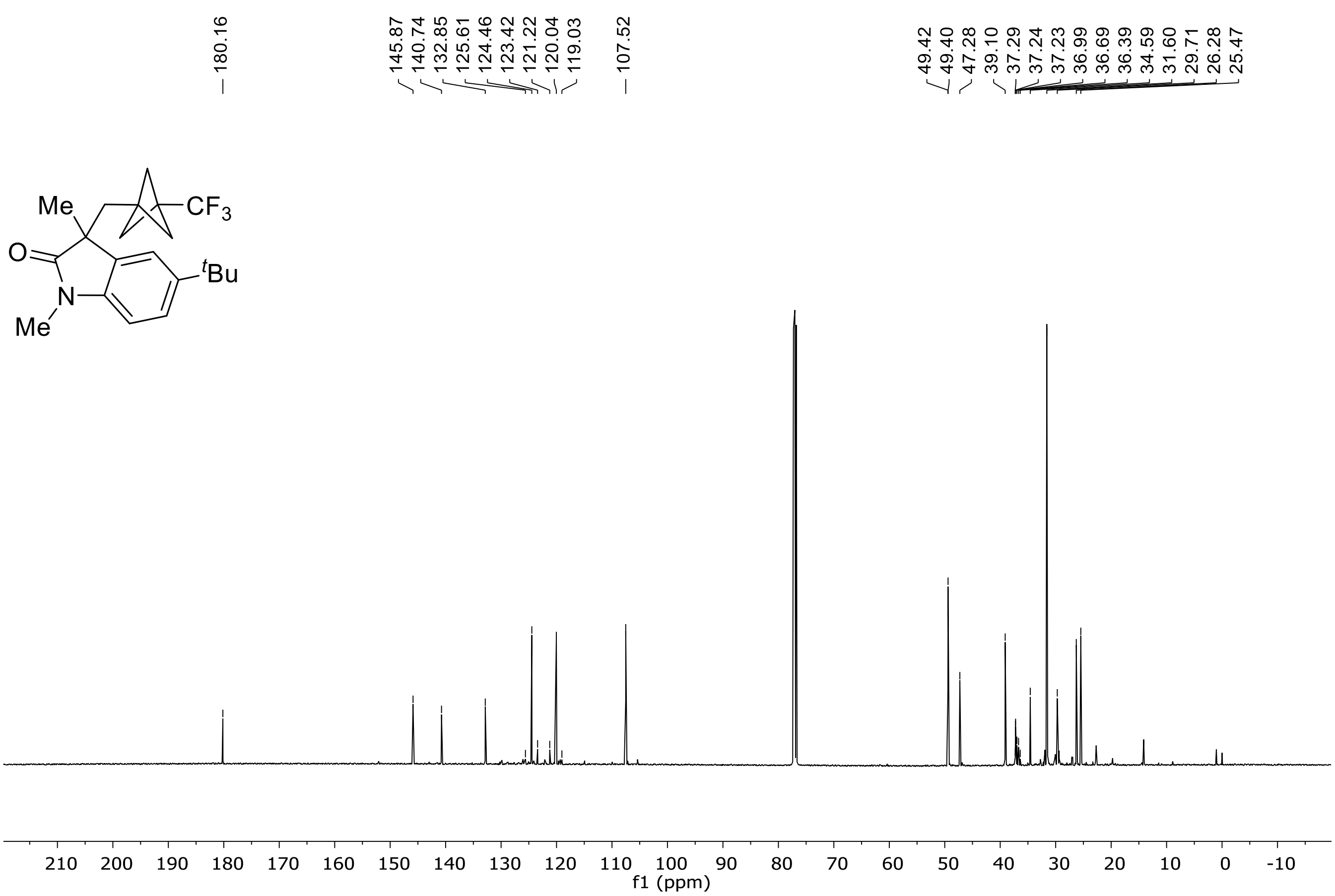

^{13}C NMR ($CDCl_3$, 126 MHz) of compound **6.**

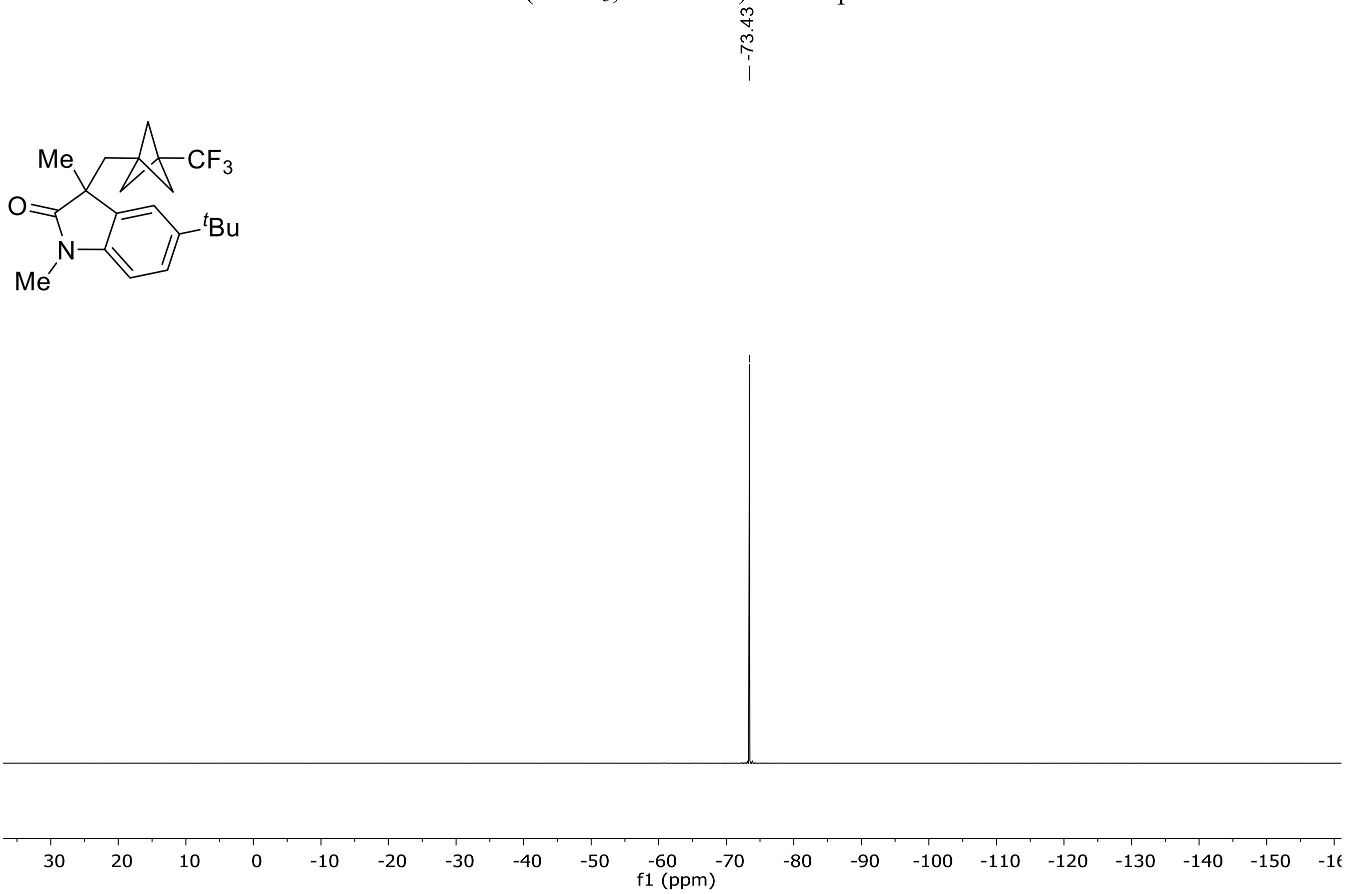

^{19}F NMR ($CDCl_3$, 286 MHz) of compound **6.**

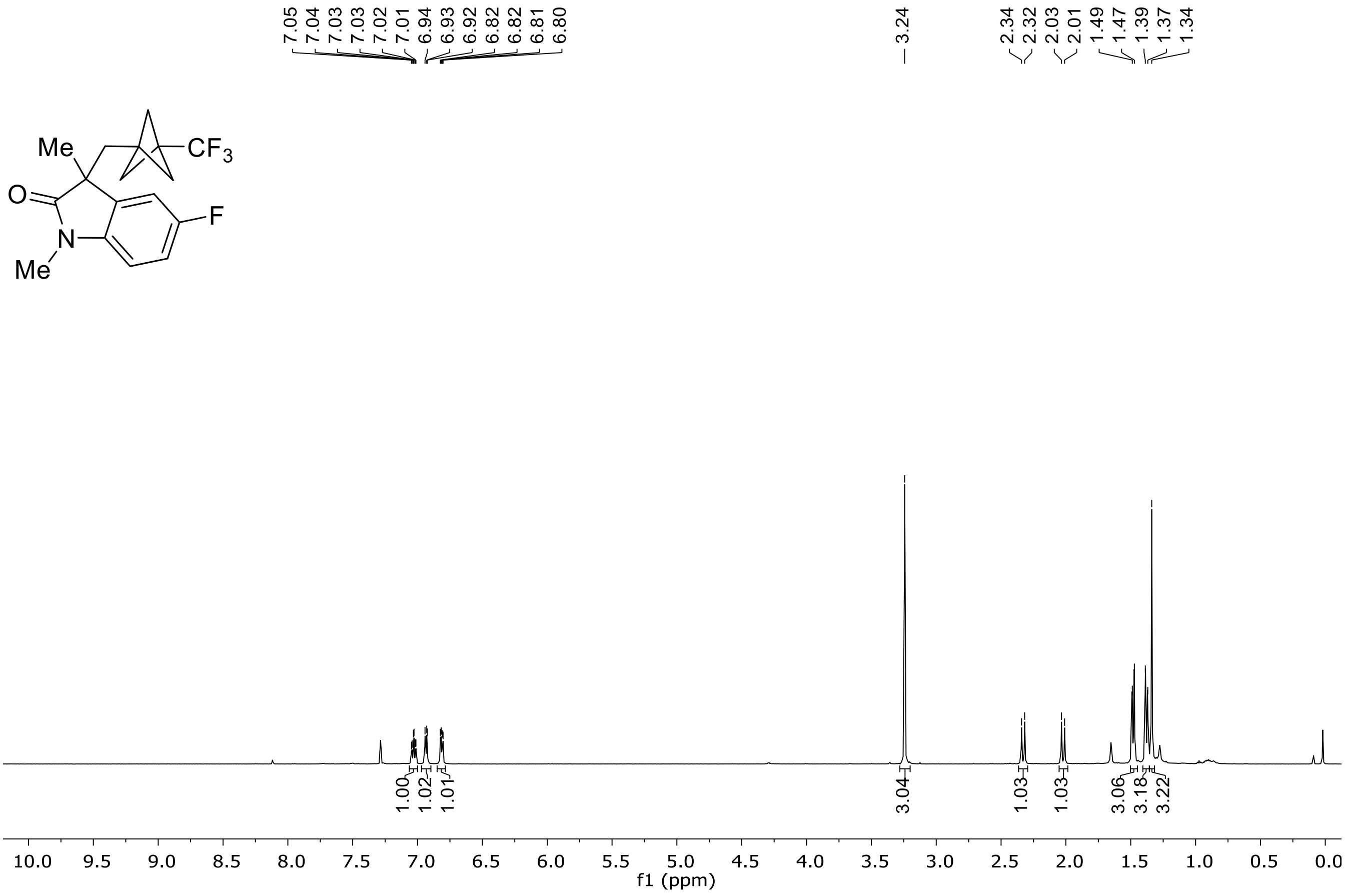

^{1}H NMR ($CDCl_3$, 300 MHz) of compound **7.**

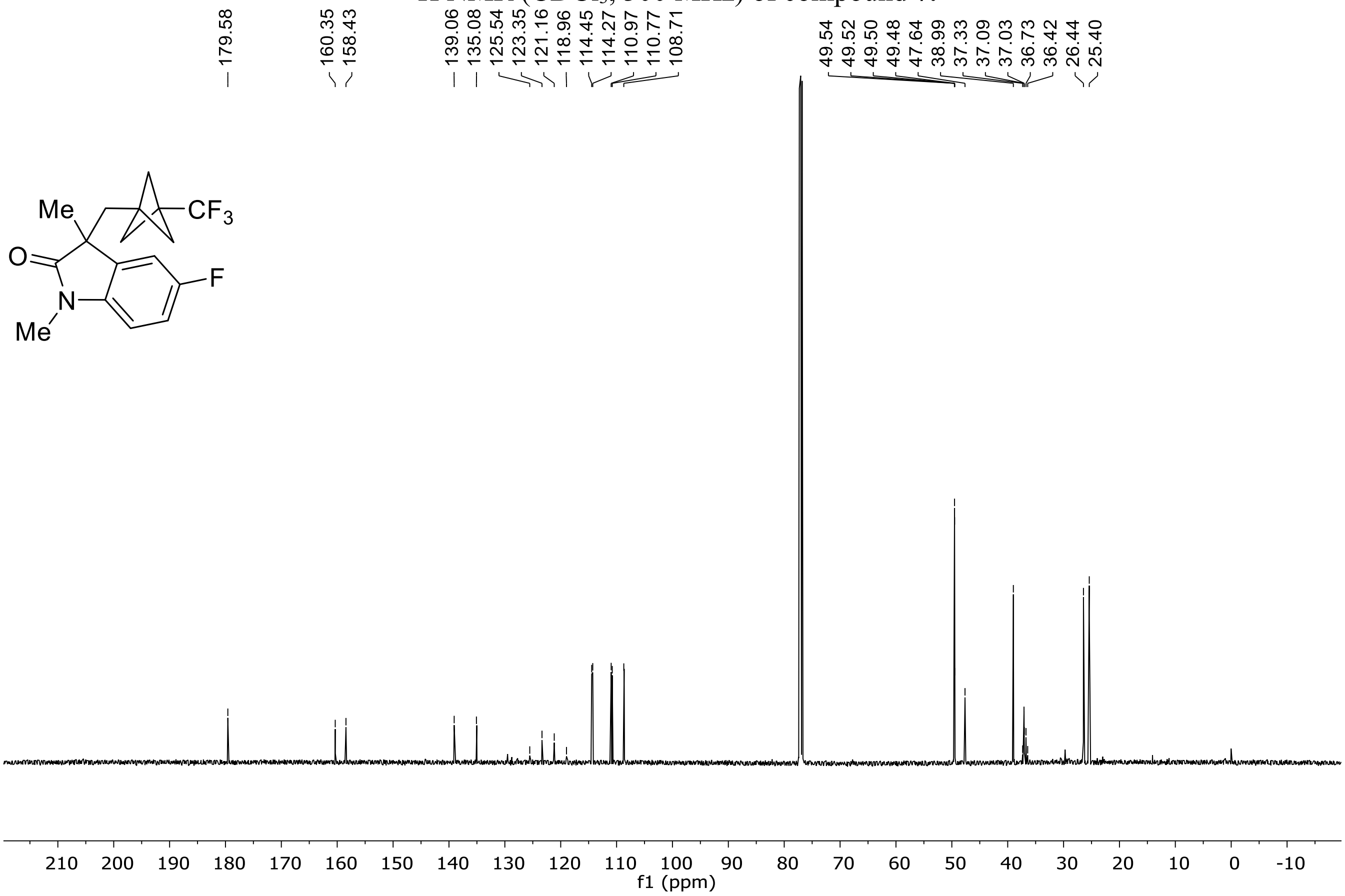

^{13}C NMR ($CDCl_3$, 126 MHz) of compound **7.**

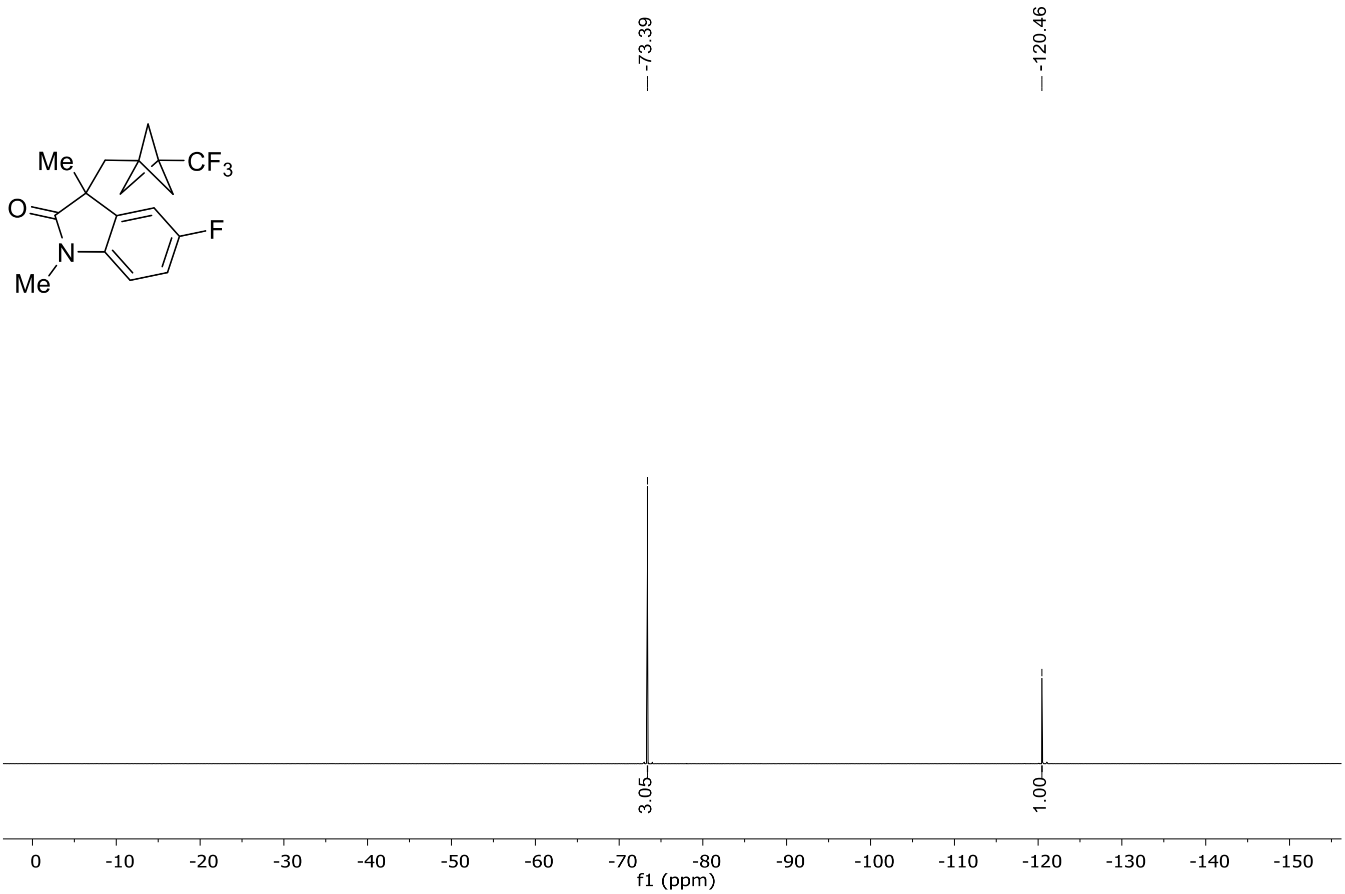

^{19}F NMR ($CDCl_3$, 282 MHz) of compound **7.**

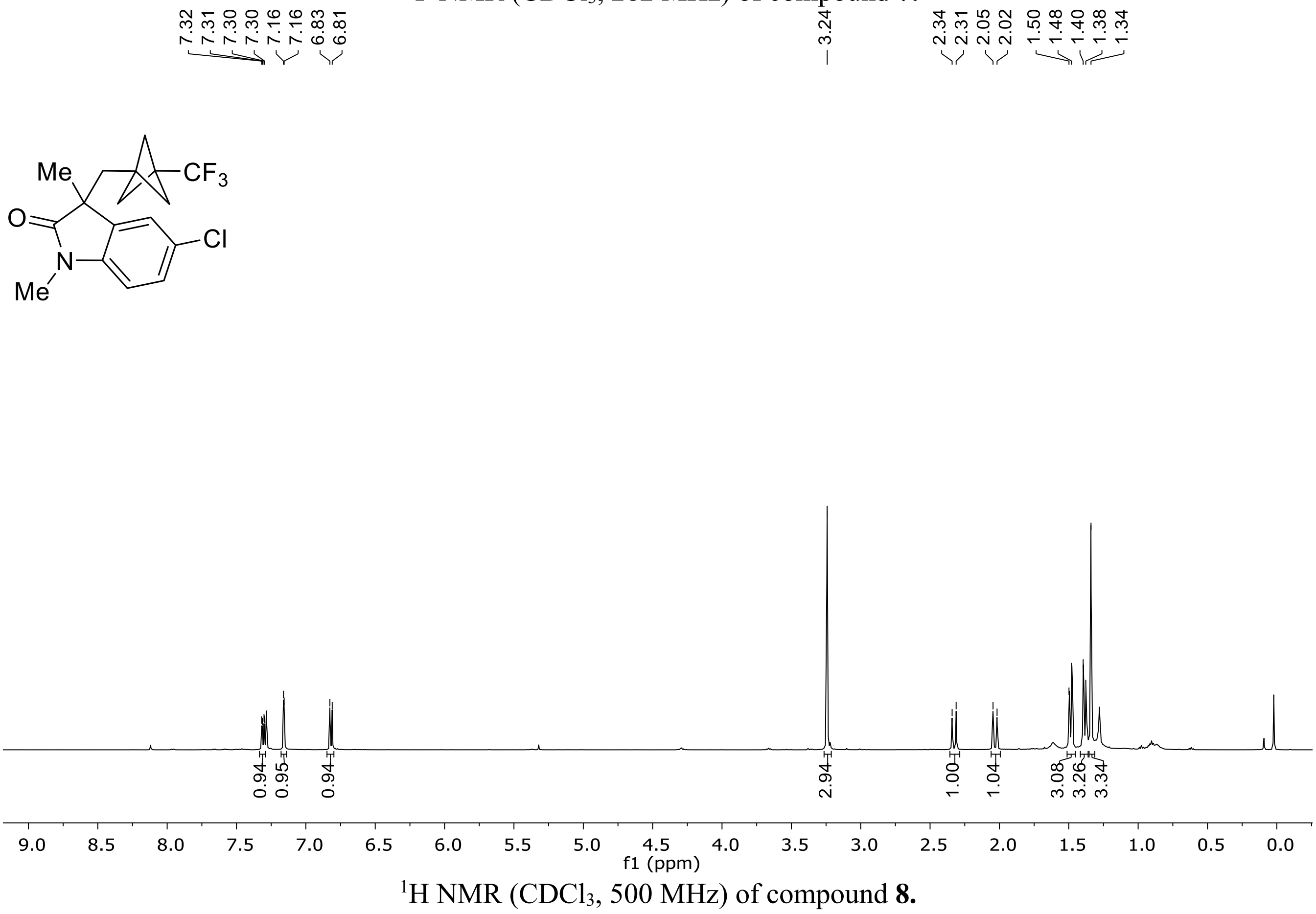

^{1}H NMR ($CDCl_3$, 500 MHz) of compound **8.**

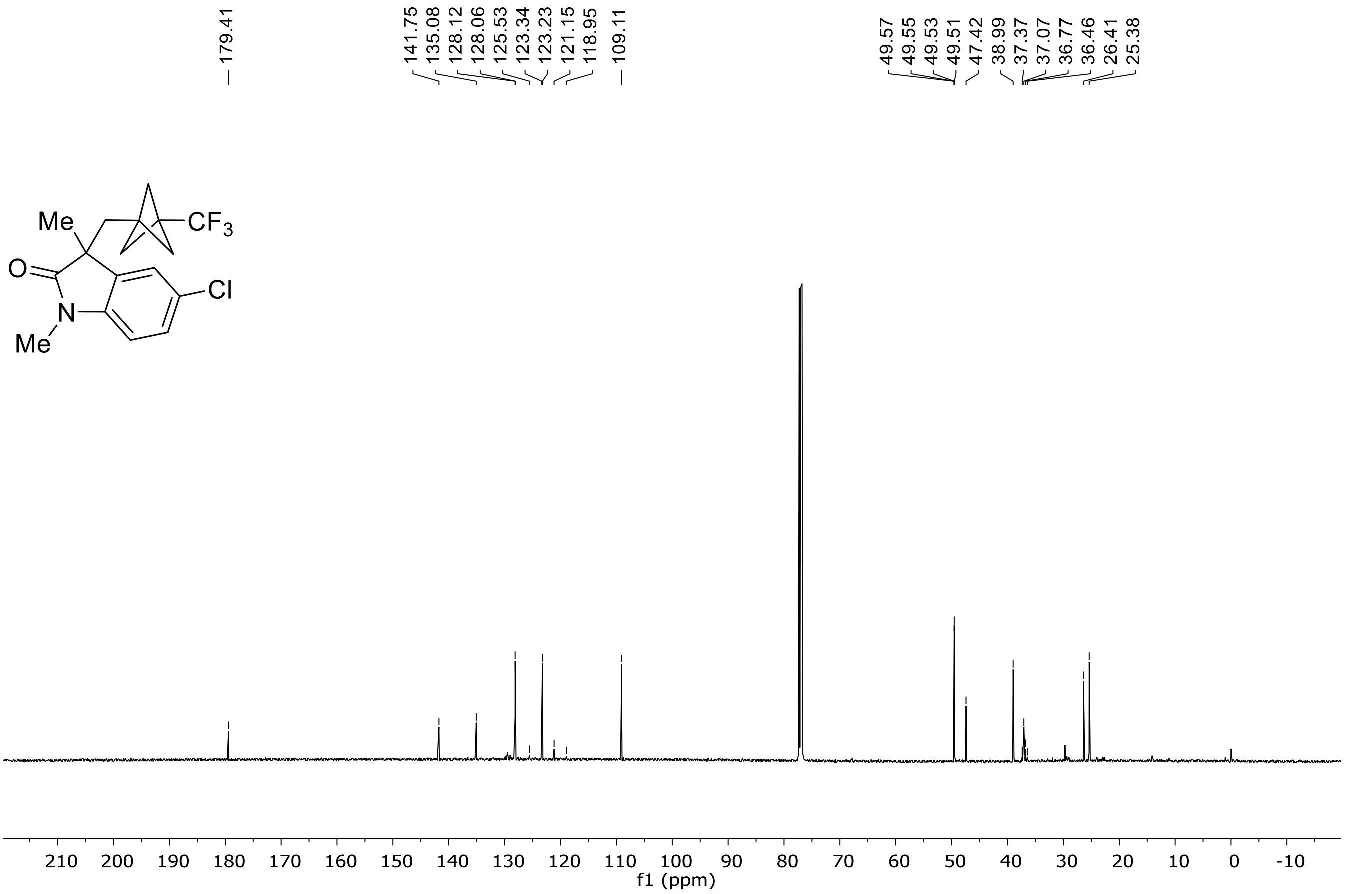

^{13}C NMR ($CDCl_3$, 126 MHz) of compound **8.**

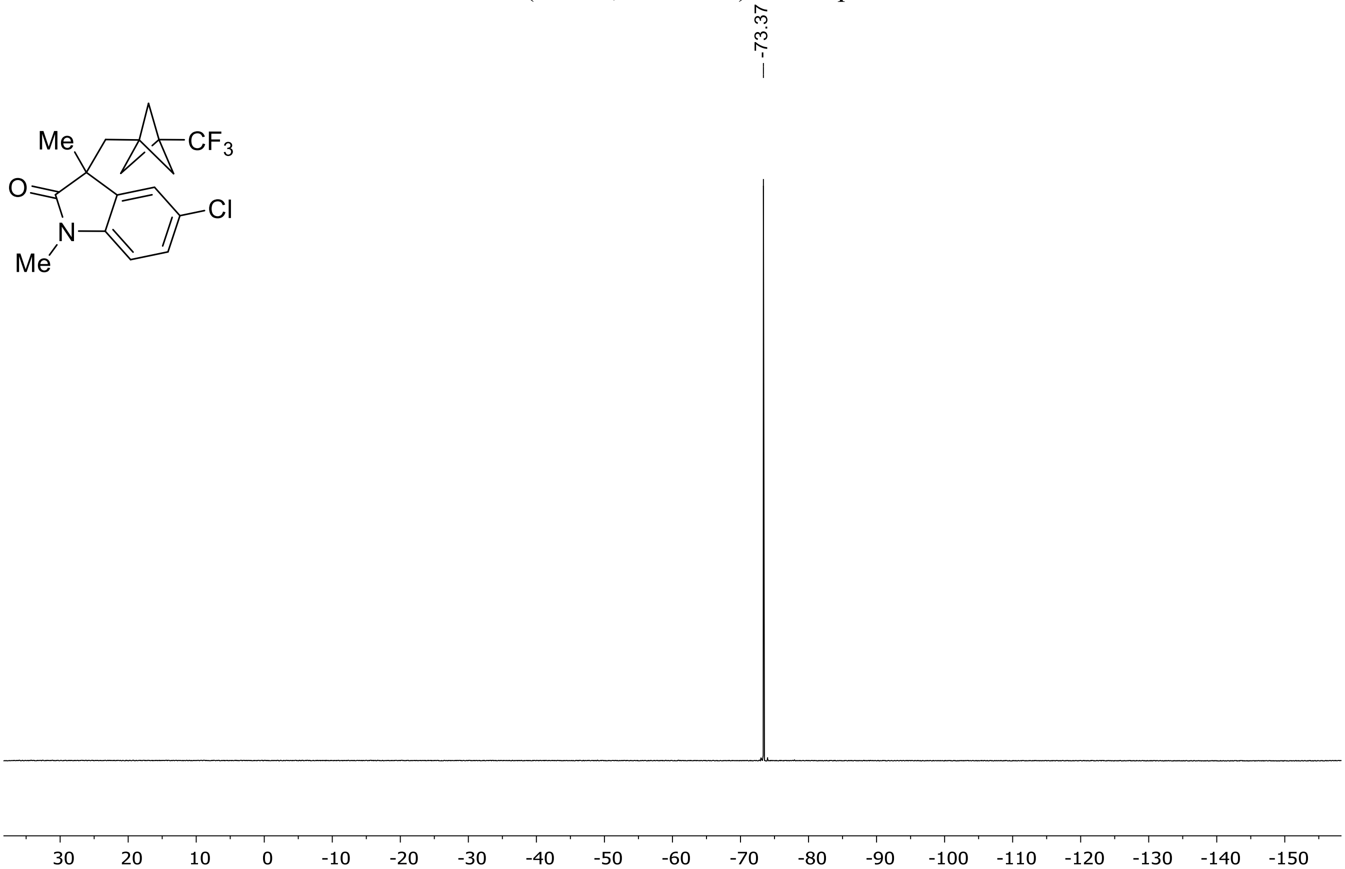

^{19}F NMR ($CDCl_3$, 282 MHz) of compound **8.**

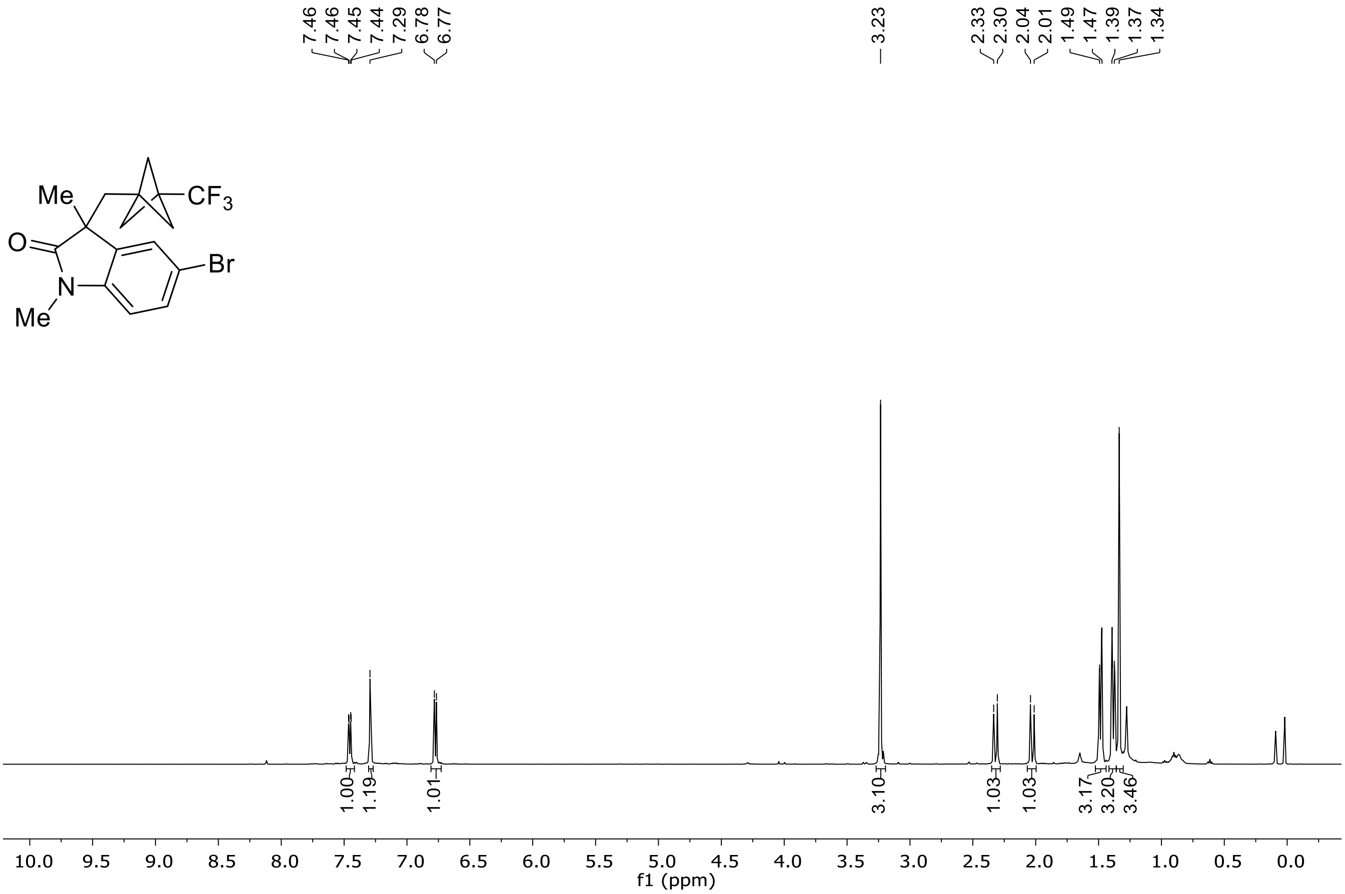

^{1}H NMR ($CDCl_3$, 500 MHz) of compound **9.**

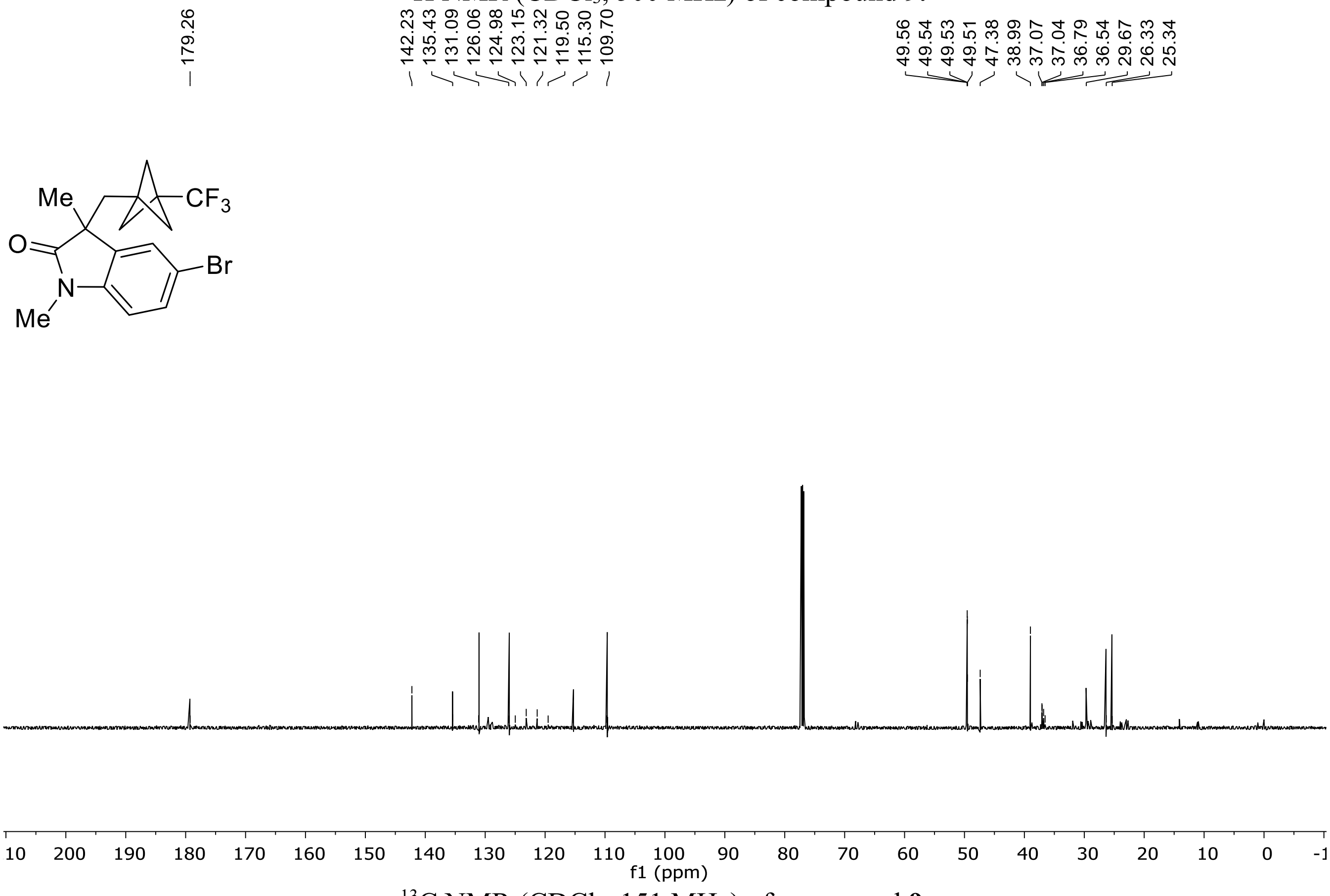

^{13}C NMR ($CDCl_3$, 151 MHz) of compound **9.**

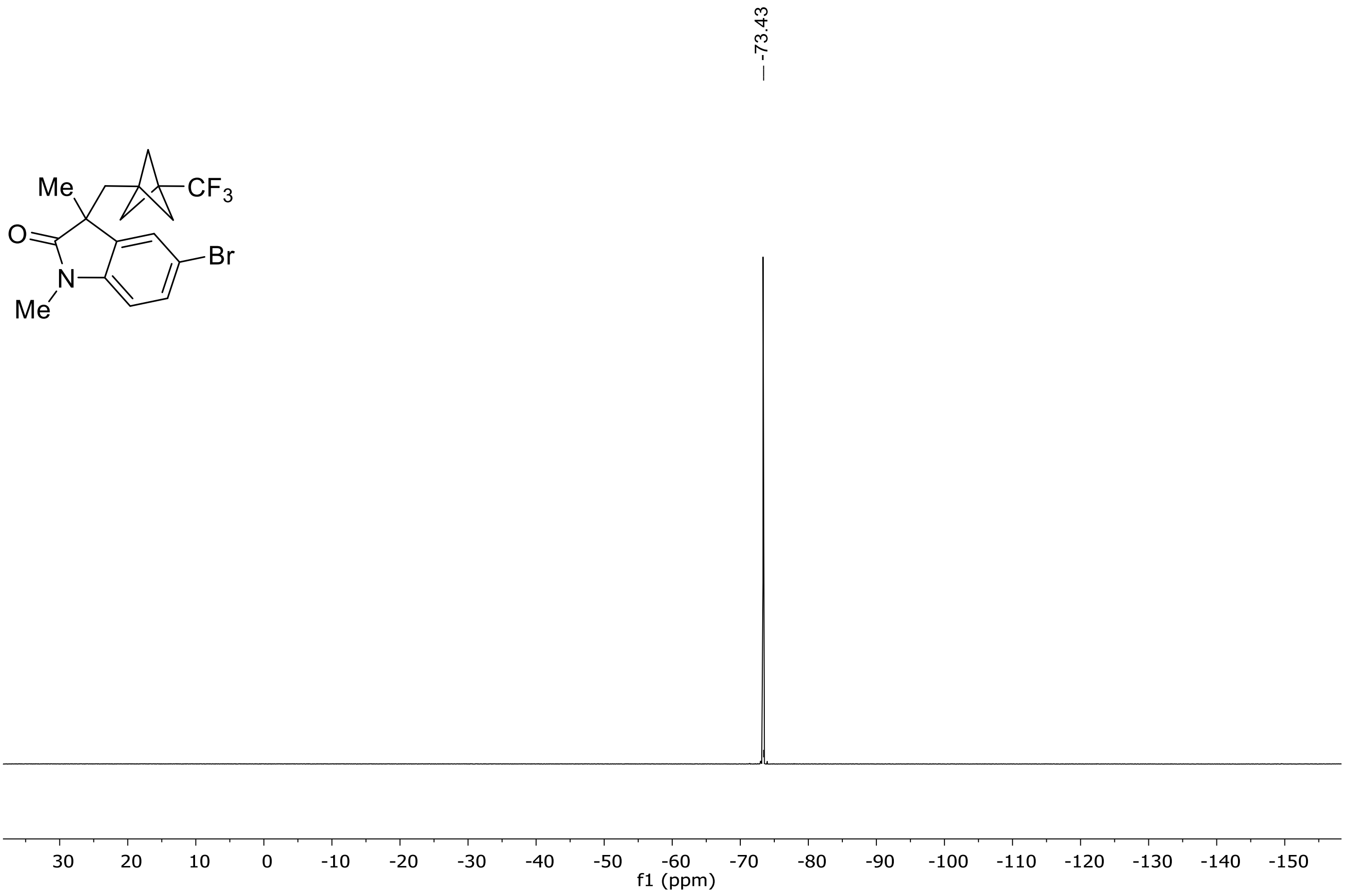

^{19}F NMR ($CDCl_3$, 282 MHz) of compound **9.**

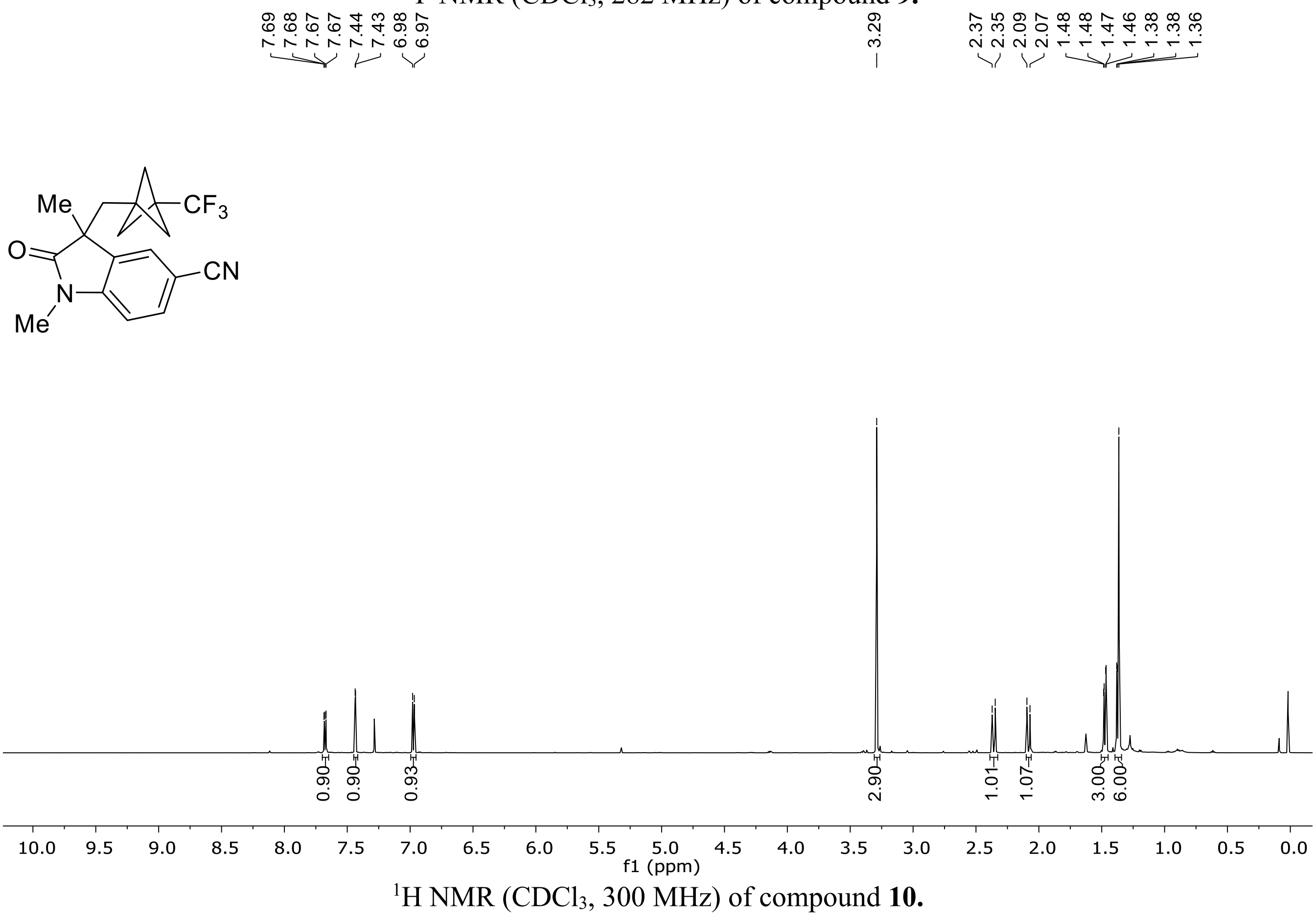

^{1}H NMR ($CDCl_3$, 300 MHz) of compound **10.**

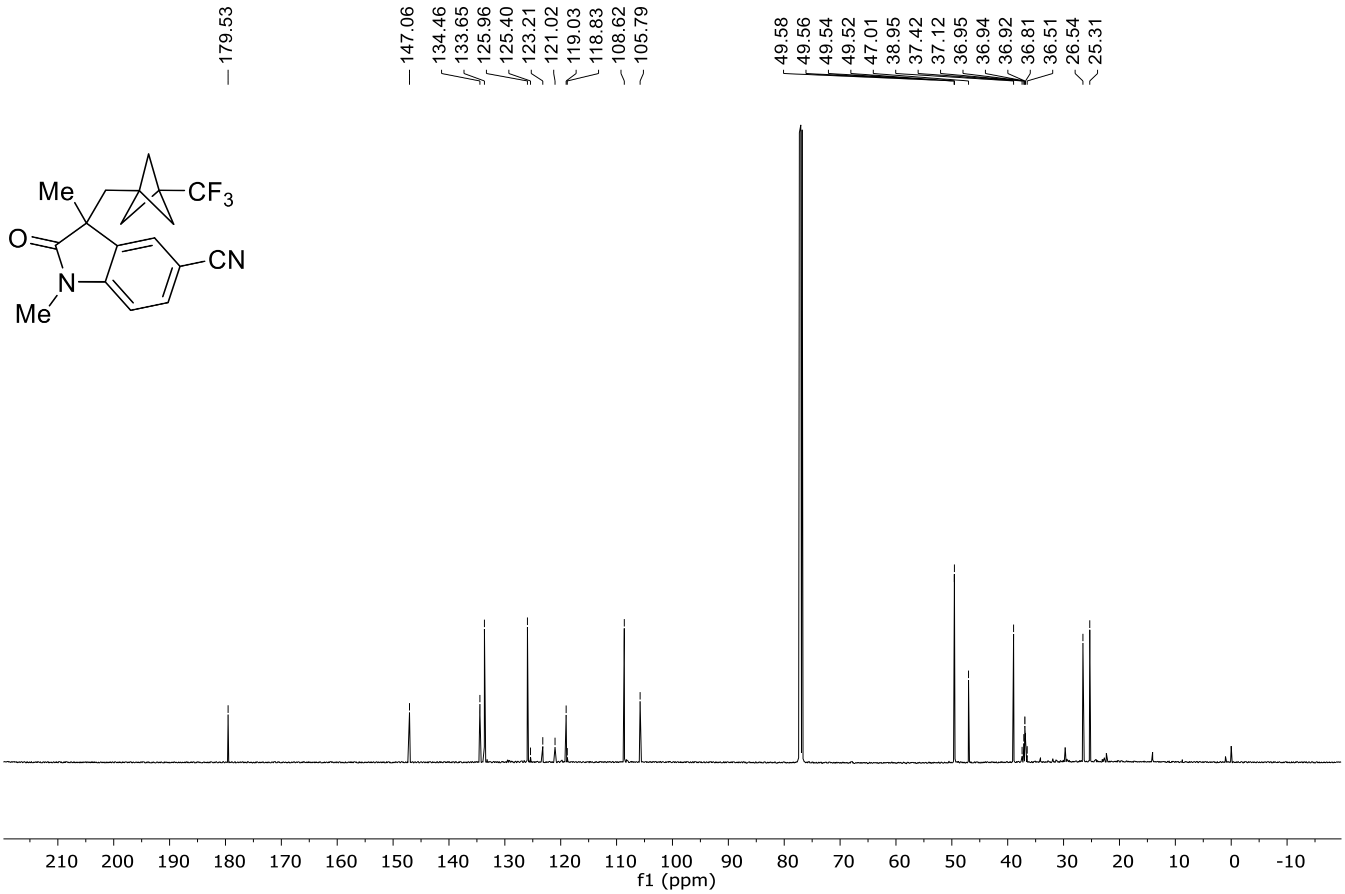

^{13}C NMR (CDCl$_3$, 126 MHz) of compound **10.**

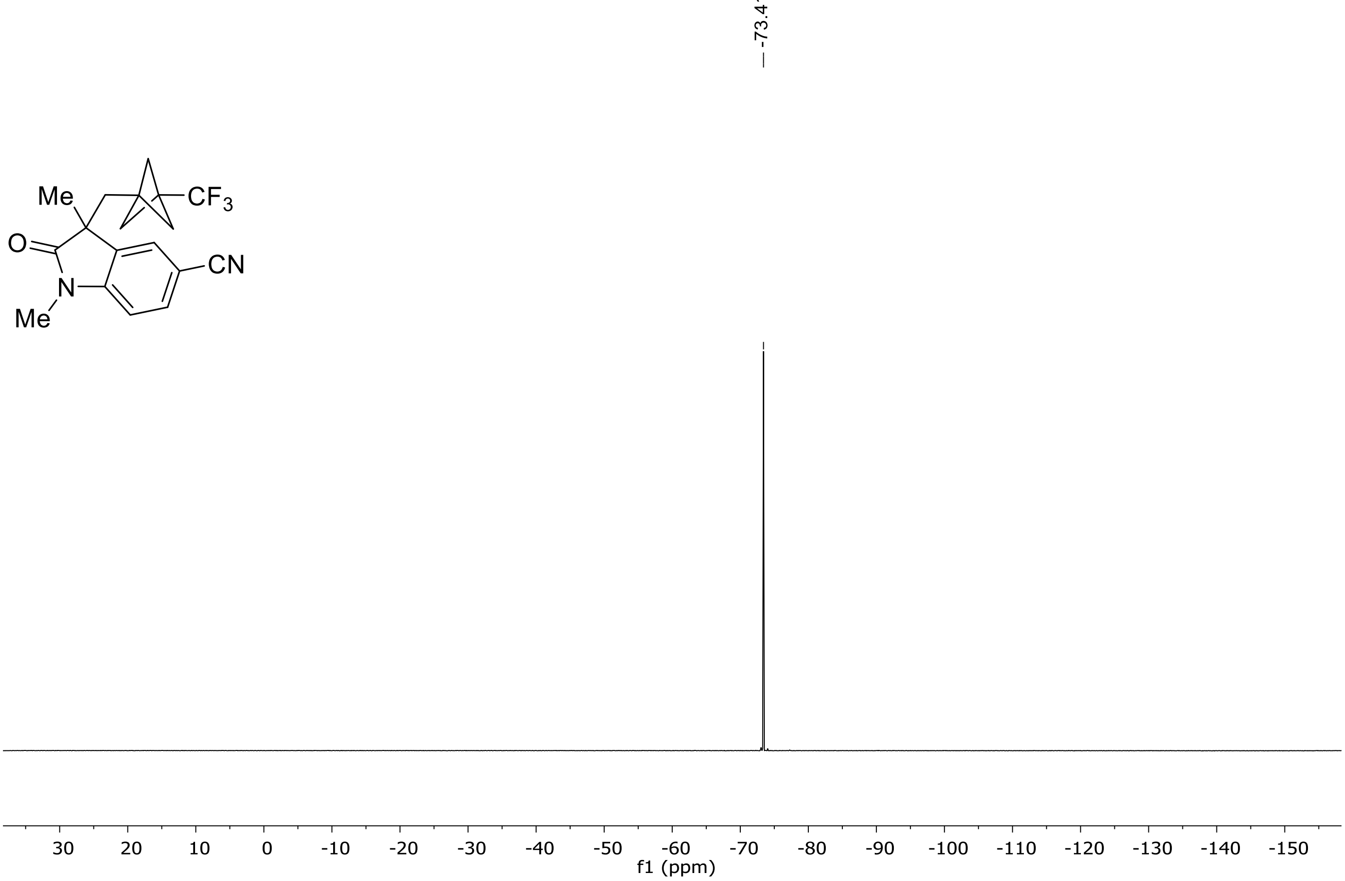

^{19}F NMR (CDCl$_3$, 282 MHz) of compound **10.**

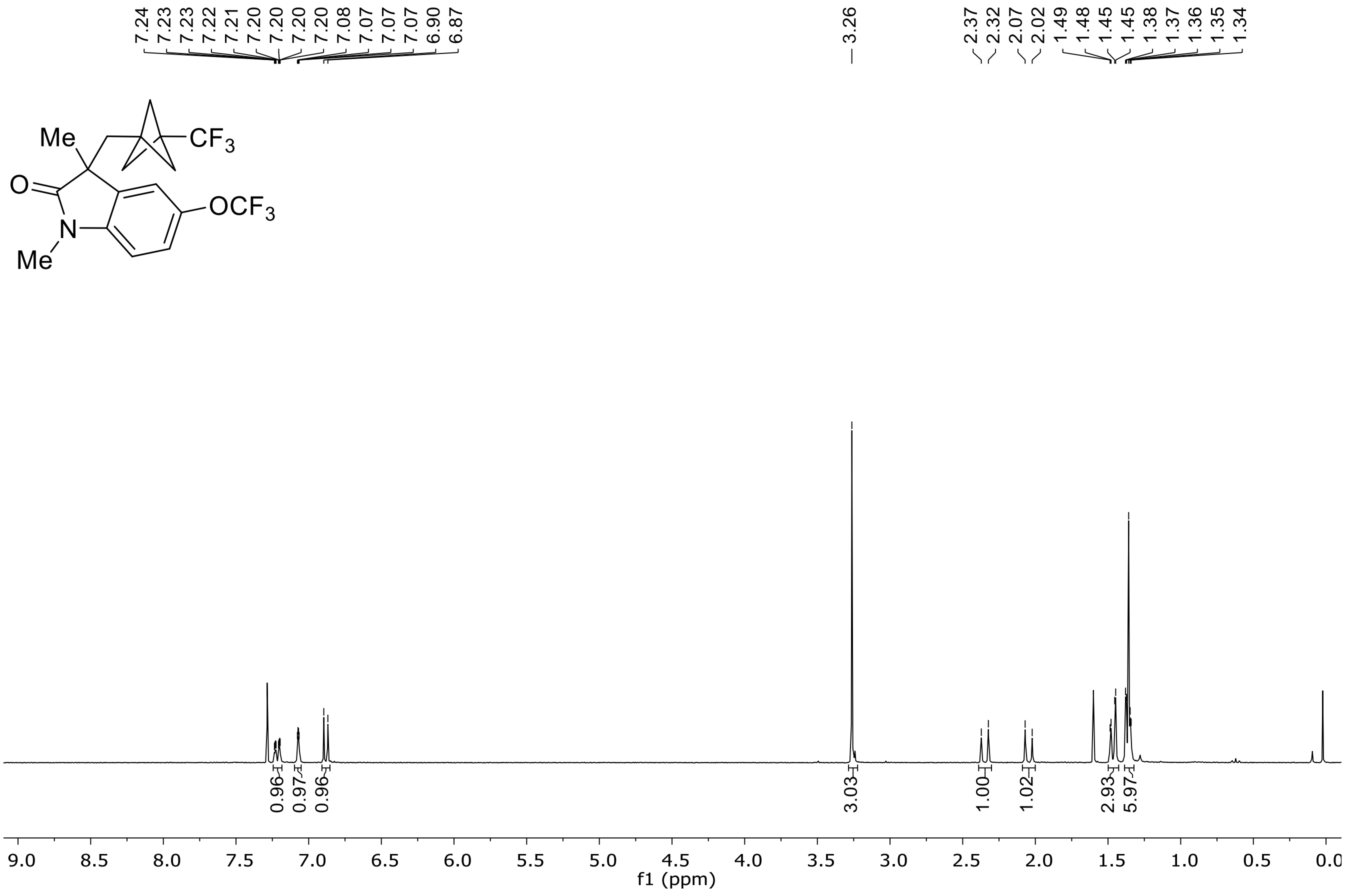

^{1}H NMR ($CDCl_3$, 300 MHz) of compound **11.**

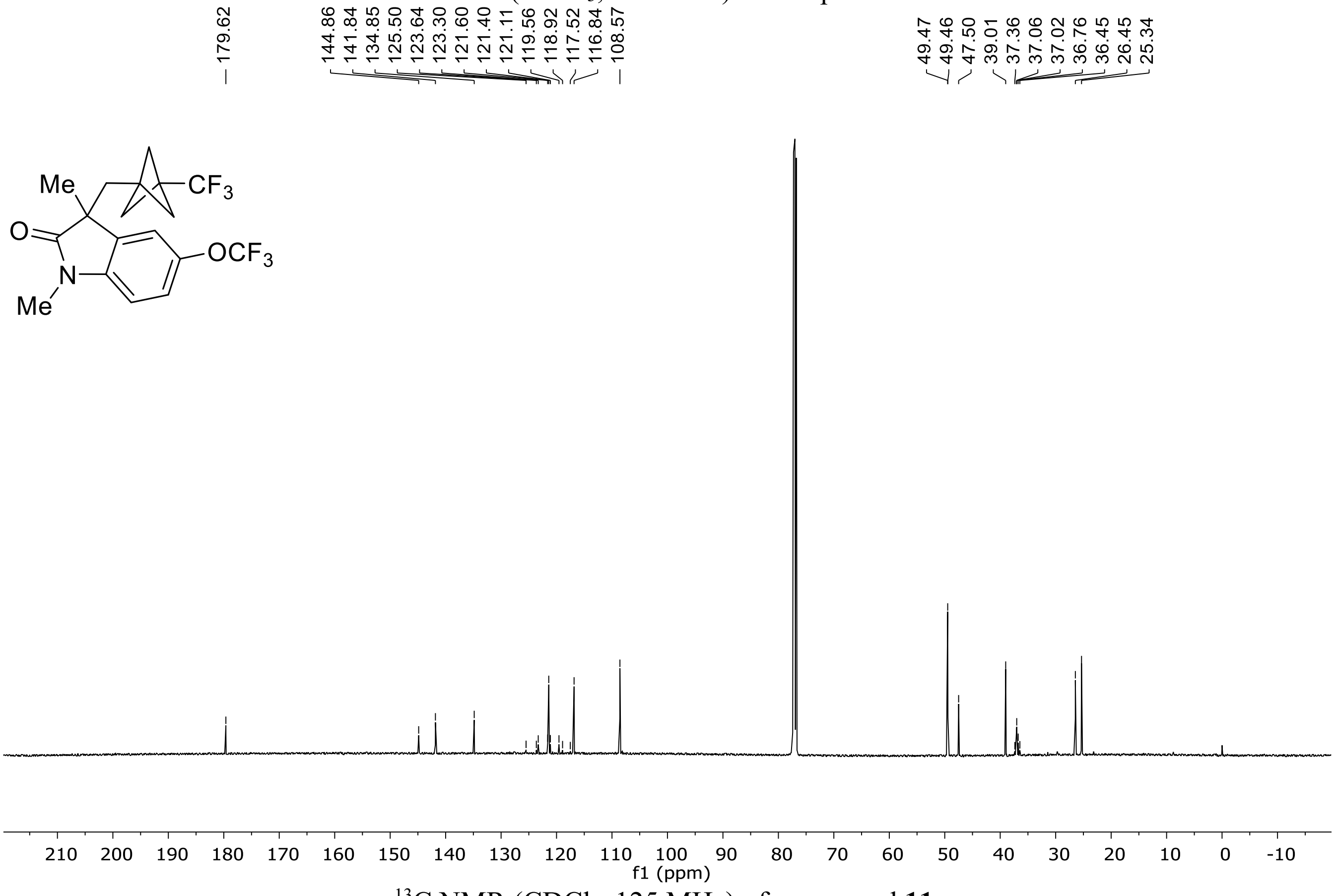

^{13}C NMR ($CDCl_3$, 125 MHz) of compound **11.**

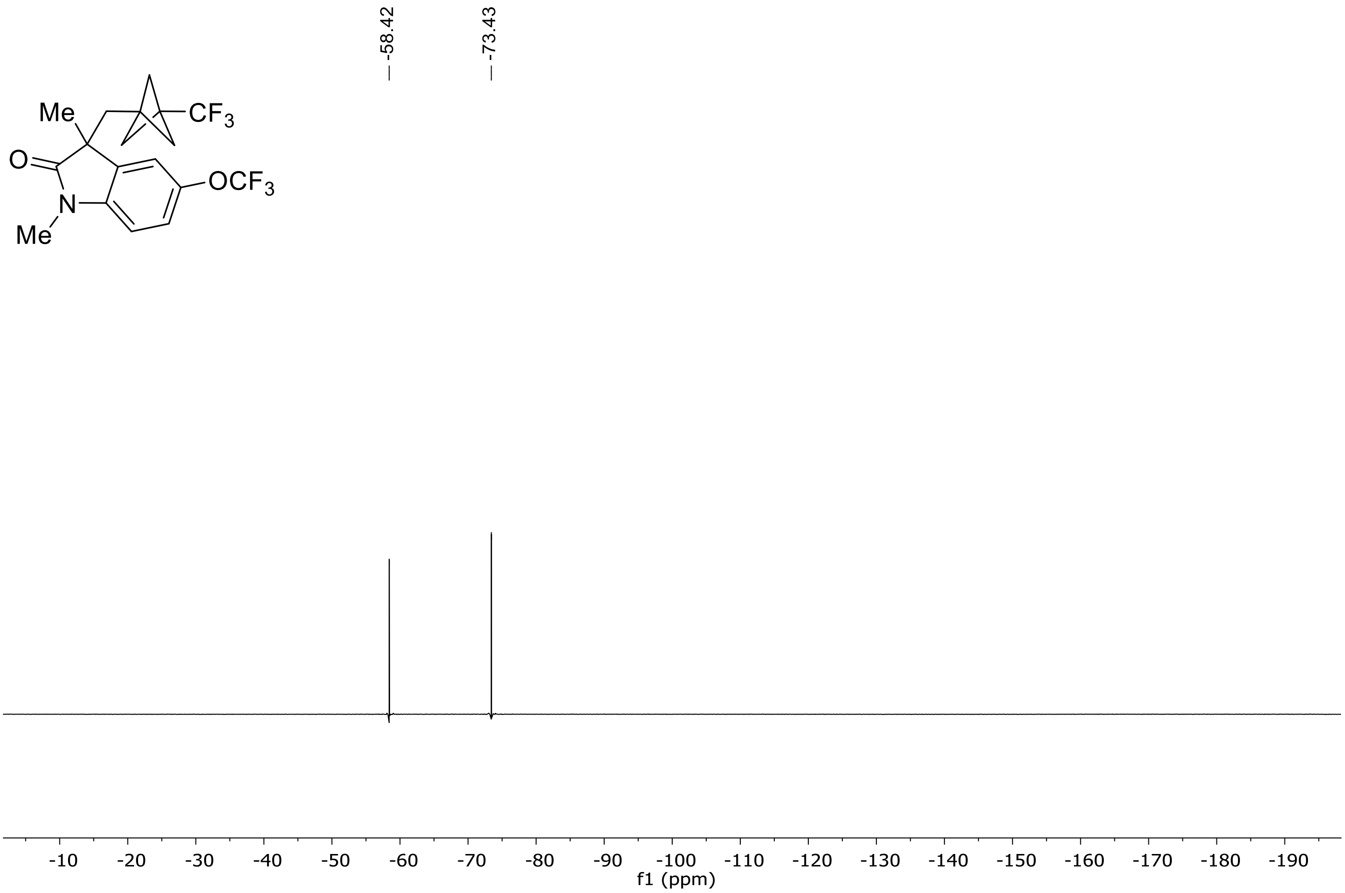

^{19}F NMR ($CDCl_3$, 282 MHz) of compound **11.**

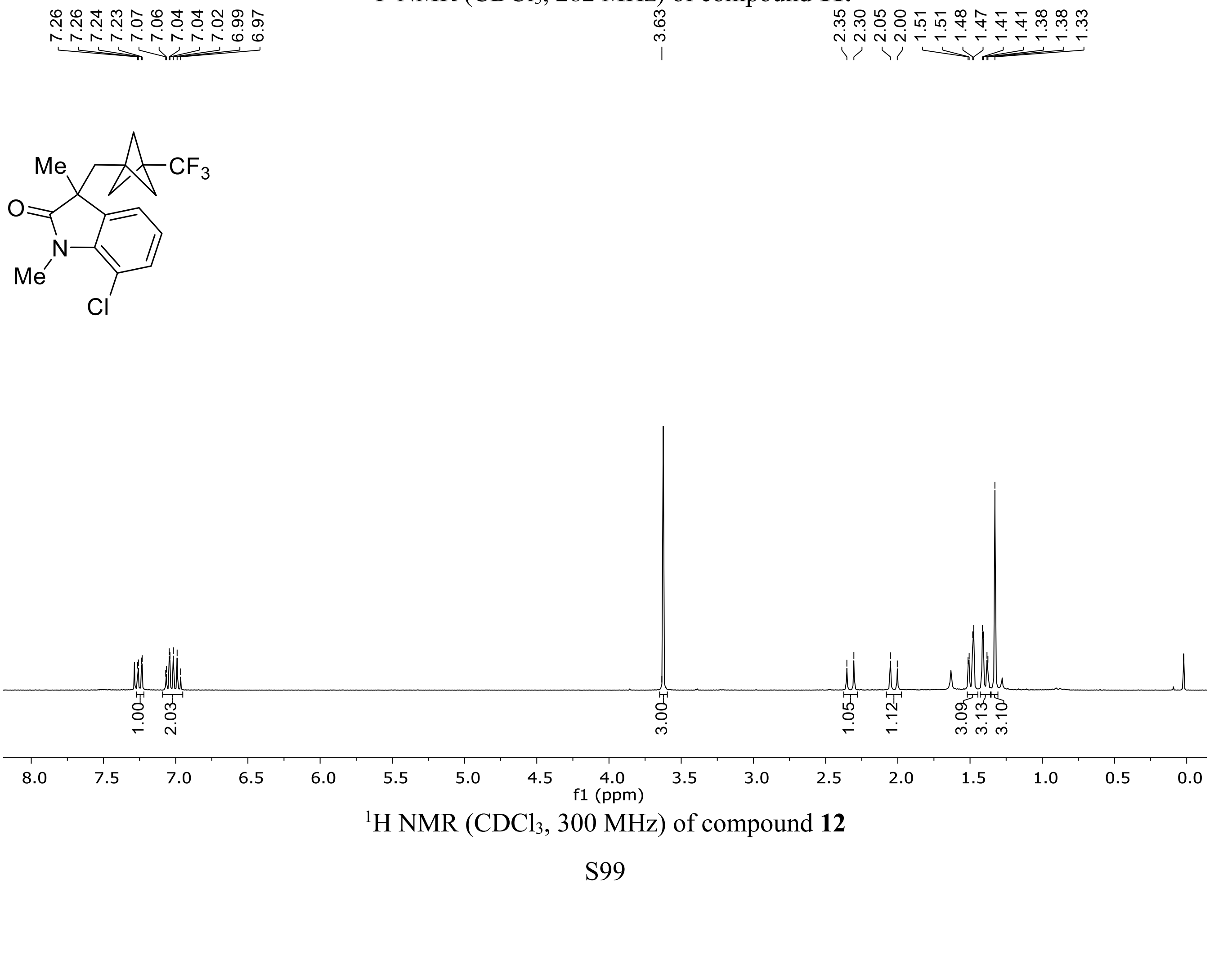

^{1}H NMR ($CDCl_3$, 300 MHz) of compound **12**

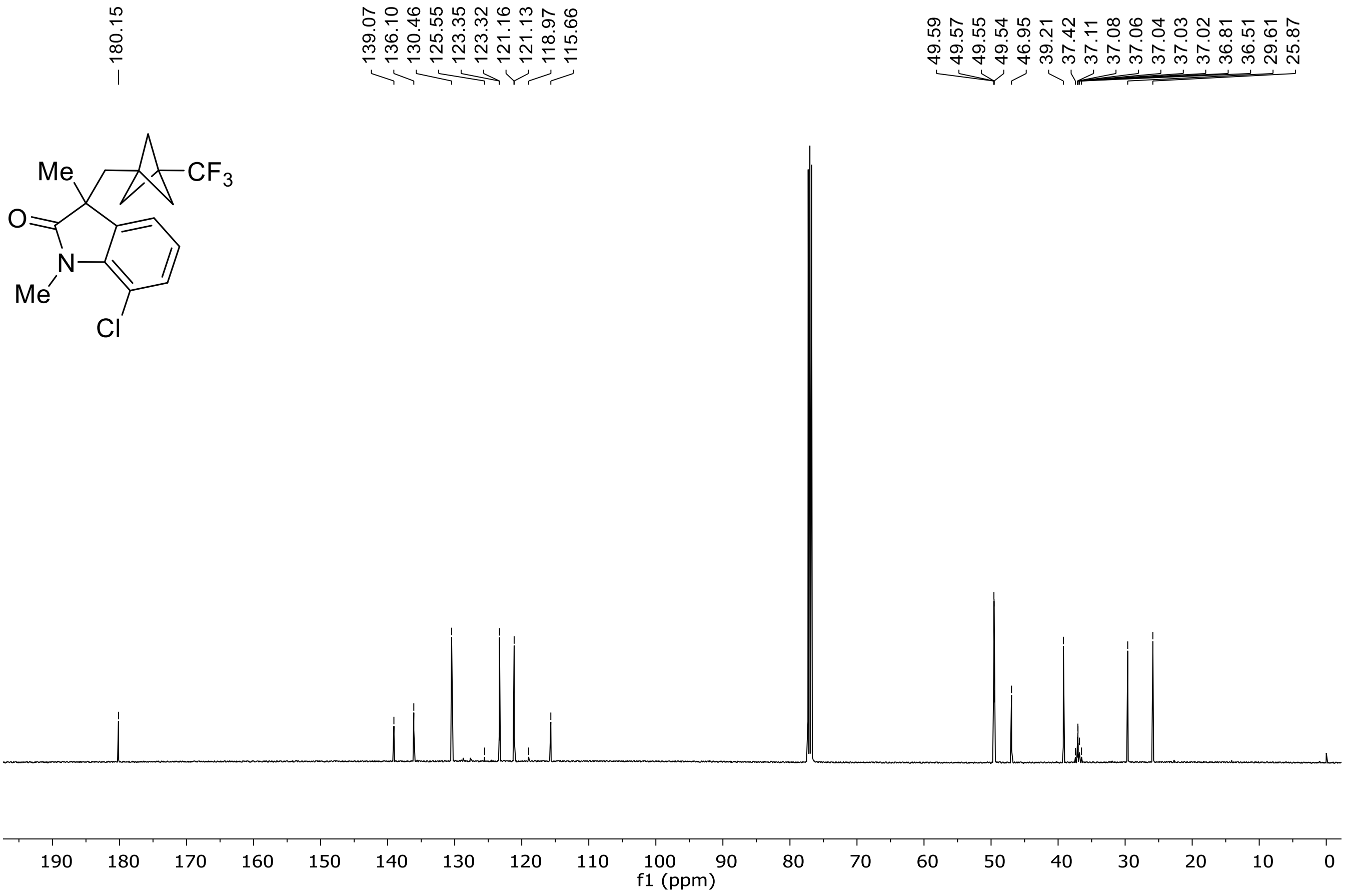

^{13}C NMR ($CDCl_3$, 126 MHz) of compound **12.**

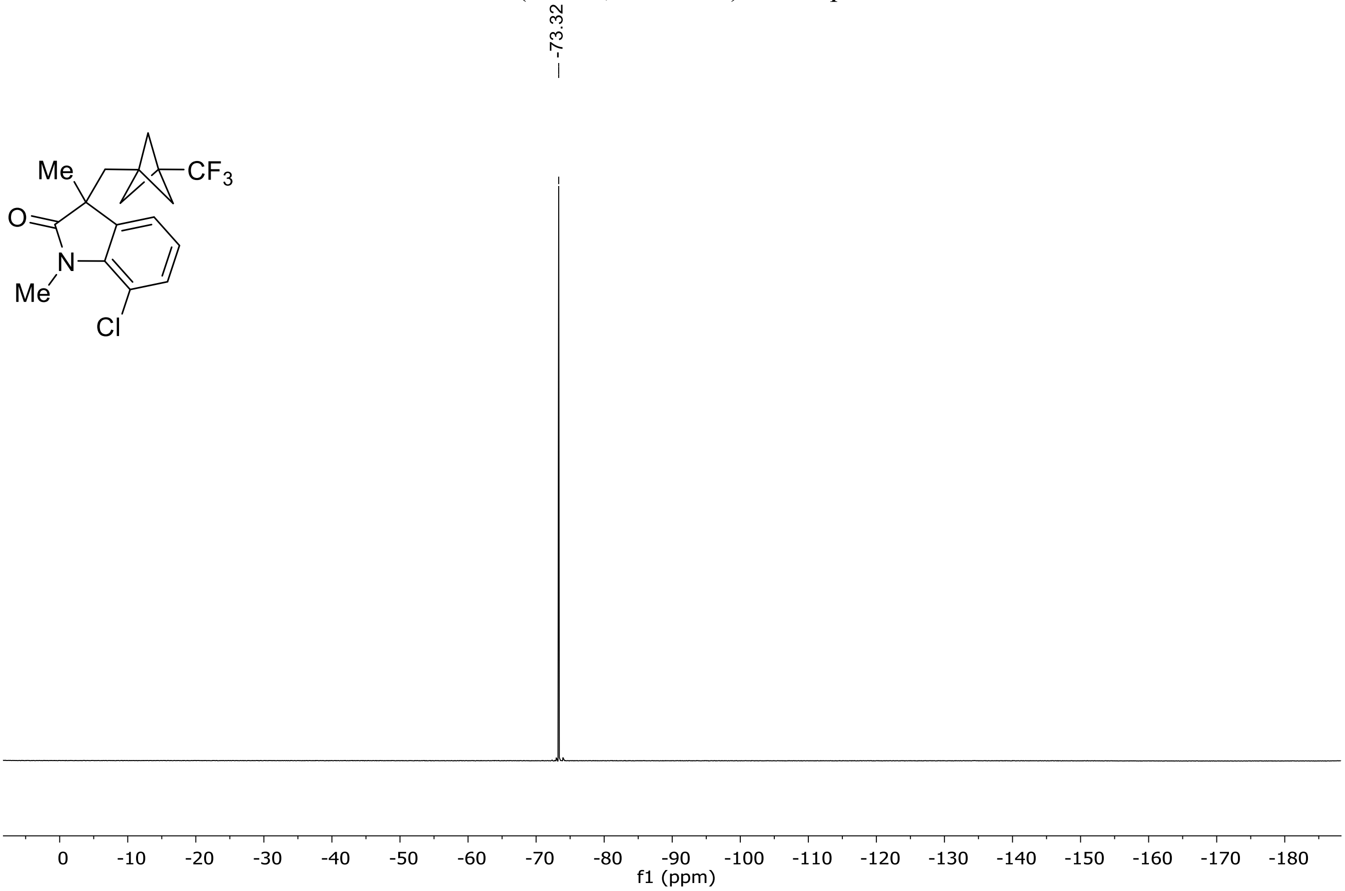

^{19}F NMR ($CDCl_3$, 282 MHz) of compound **12.**

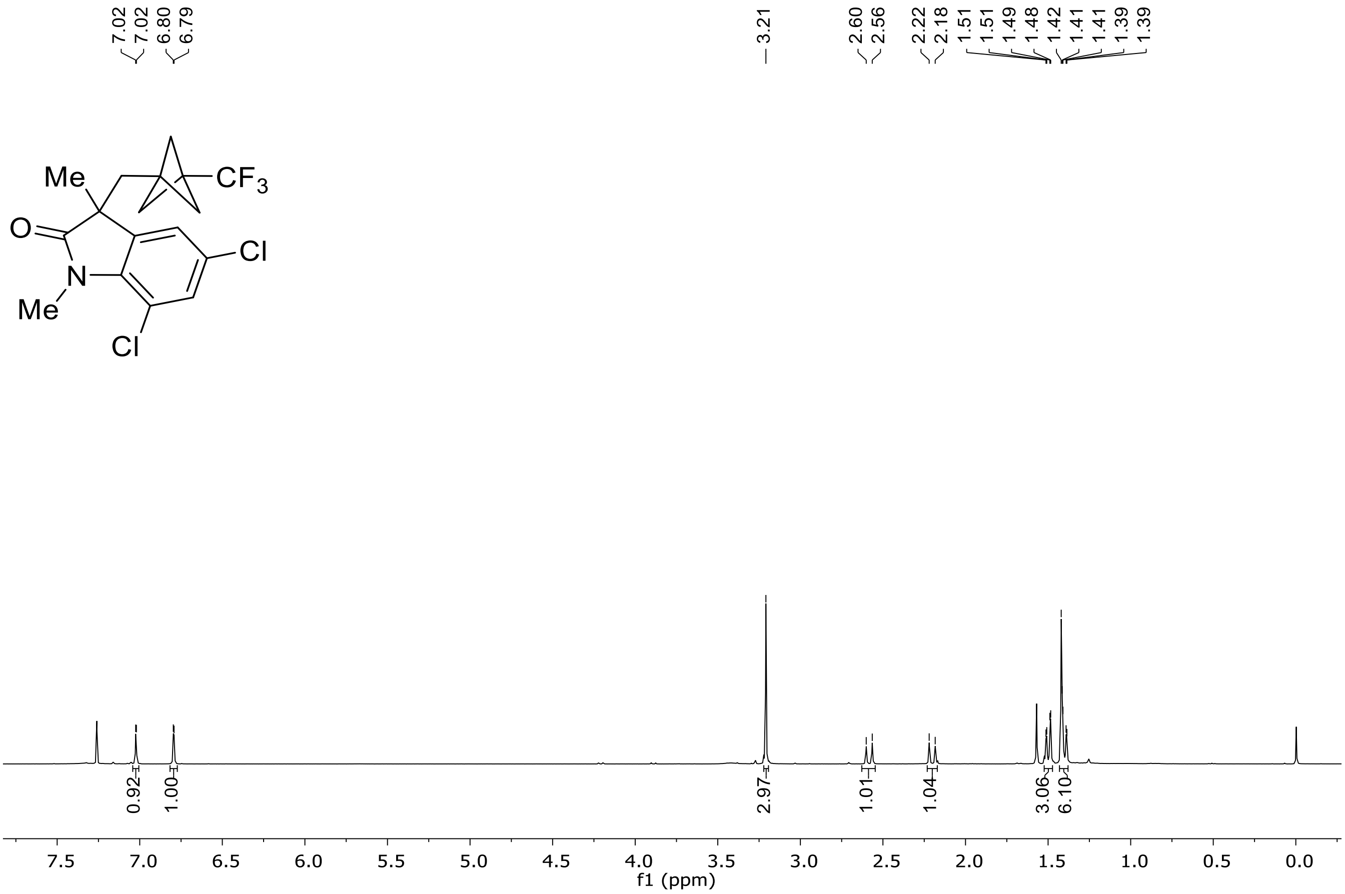

^{1}H NMR ($CDCl_3$, 400 MHz) of compound **13**

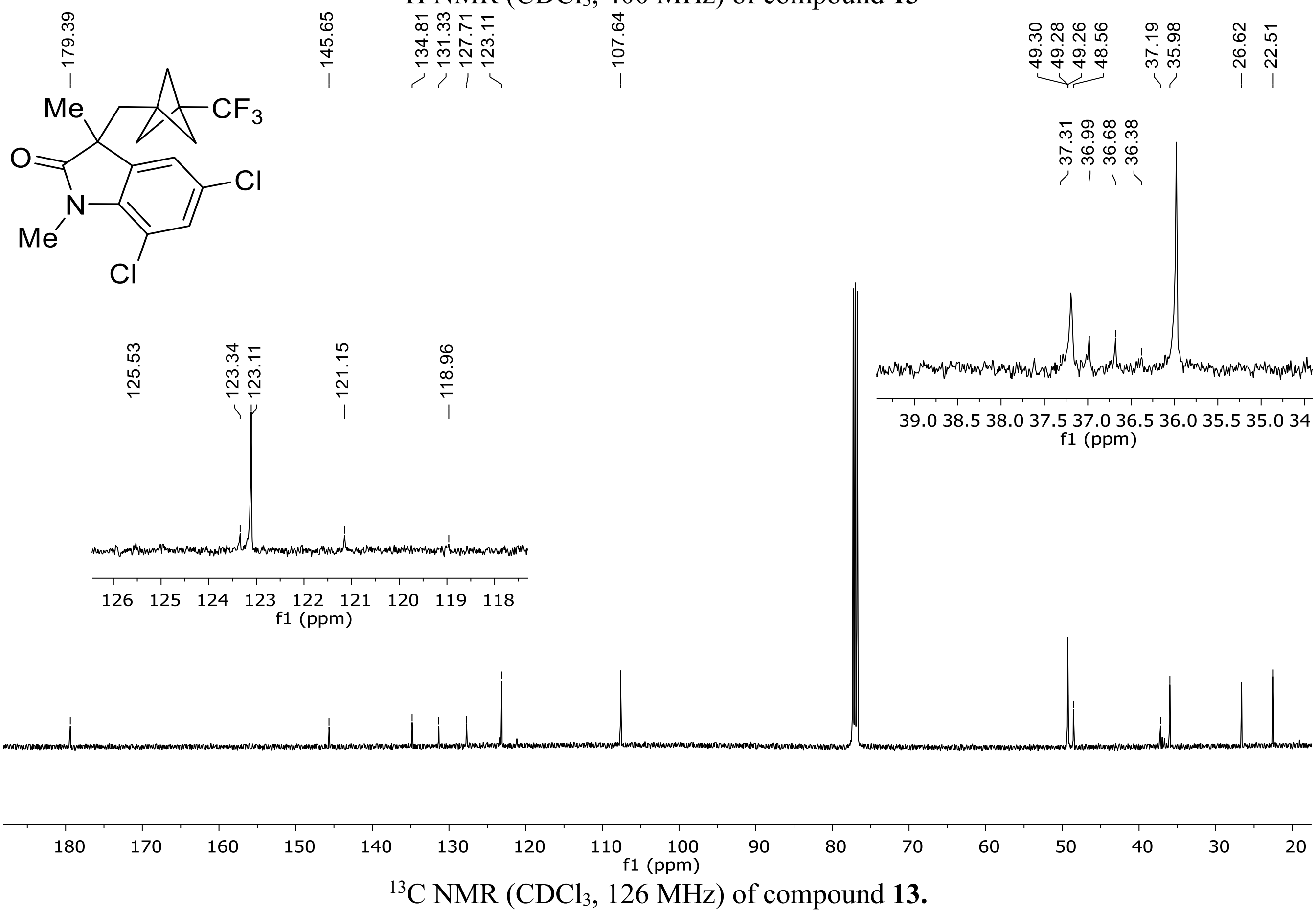

^{13}C NMR ($CDCl_3$, 126 MHz) of compound **13.**

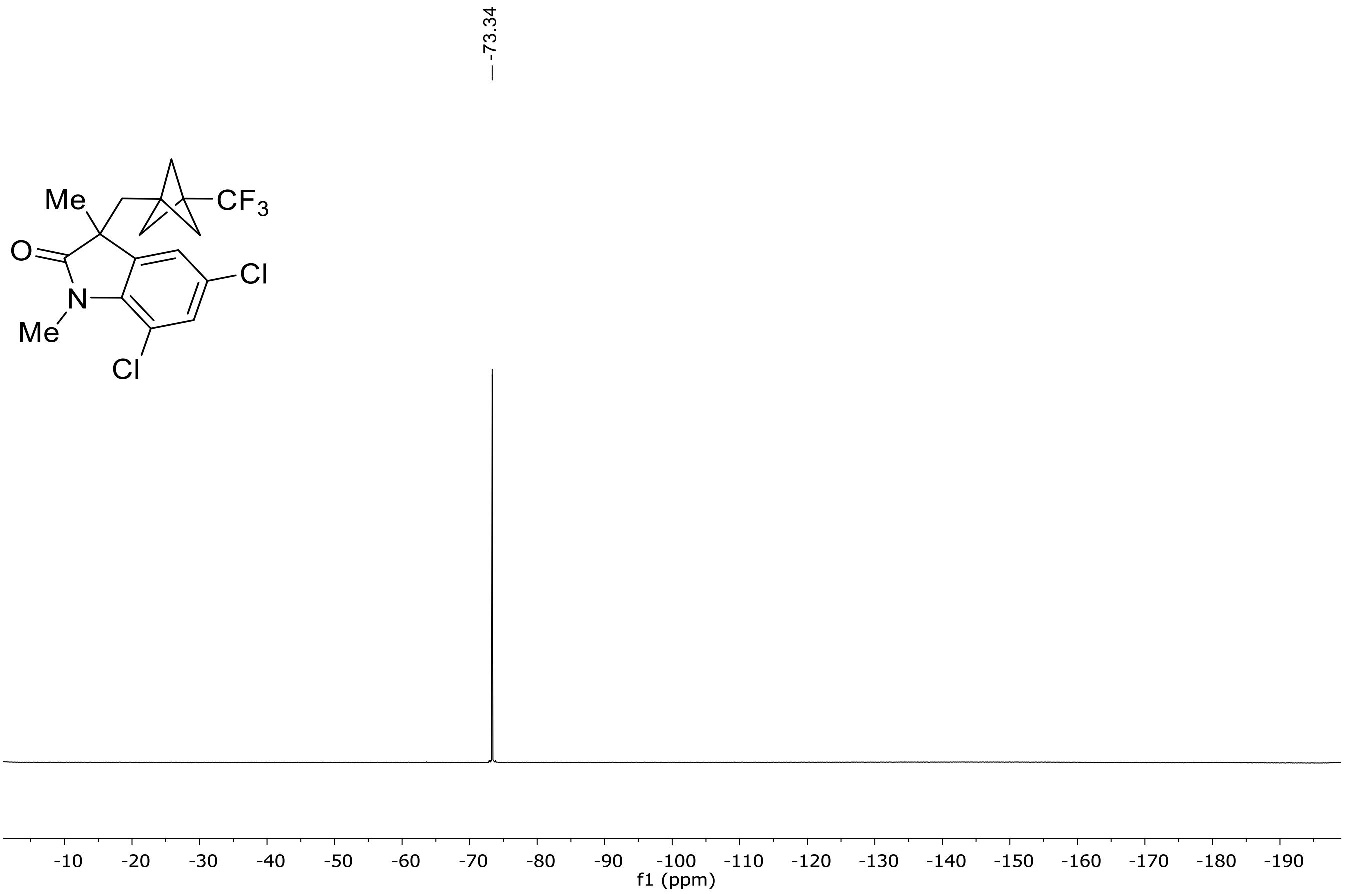

^{19}F NMR (CDCl$_3$, 377 MHz) of compound **13.**

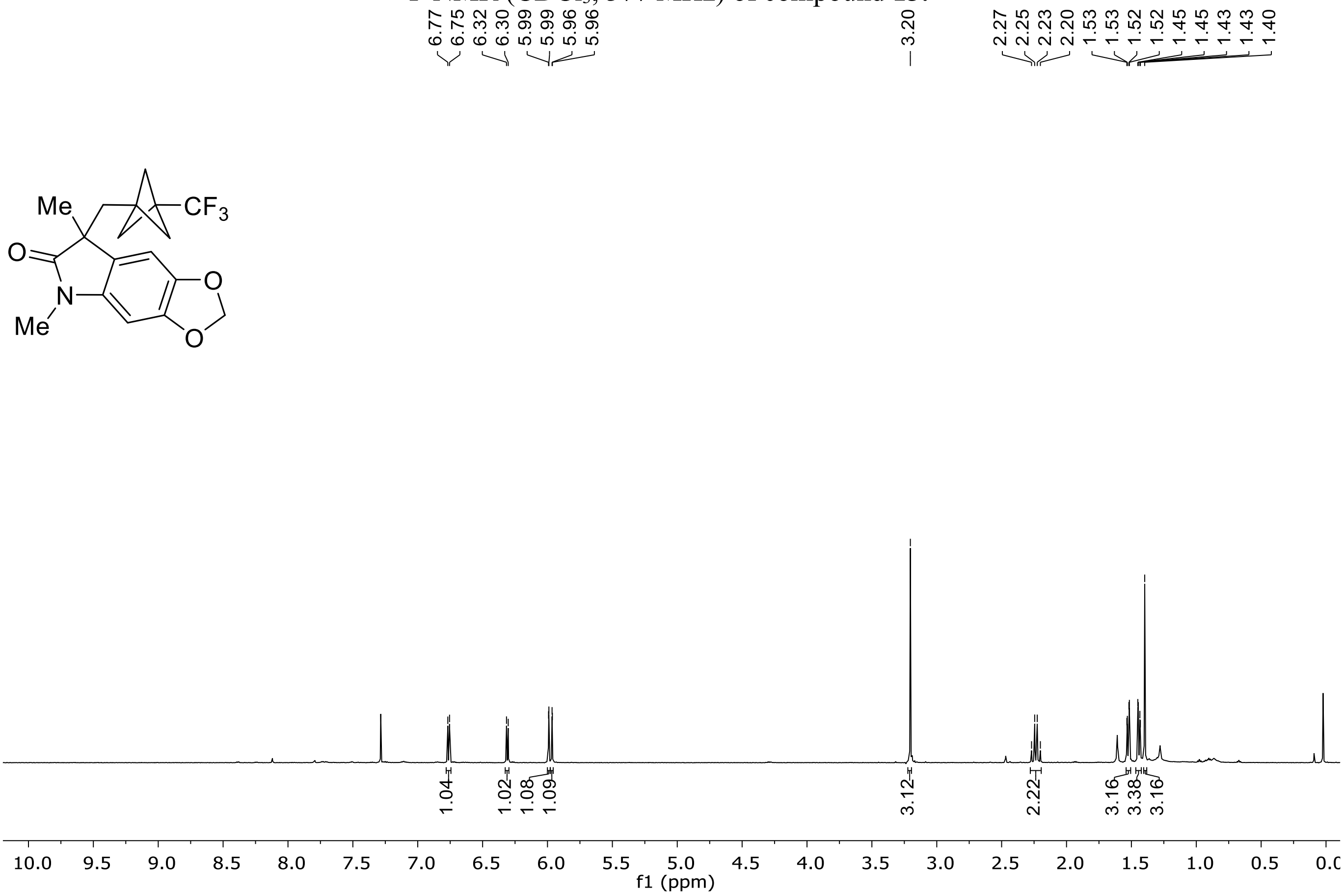

^{1}H NMR (CDCl$_3$, 600 MHz) of compound **14.**

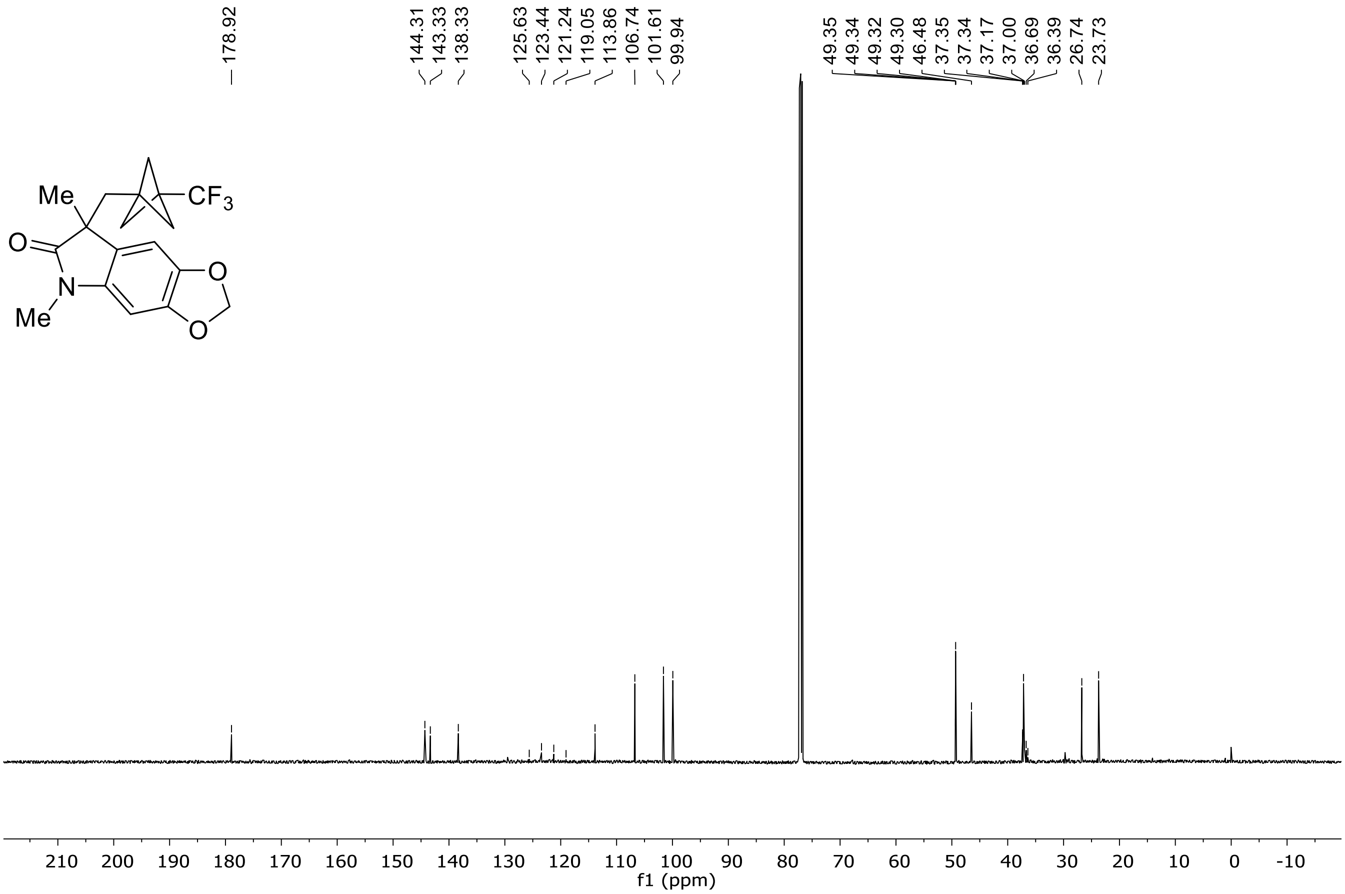

^{13}C NMR ($CDCl_3$, 126 MHz) of compound **14.**

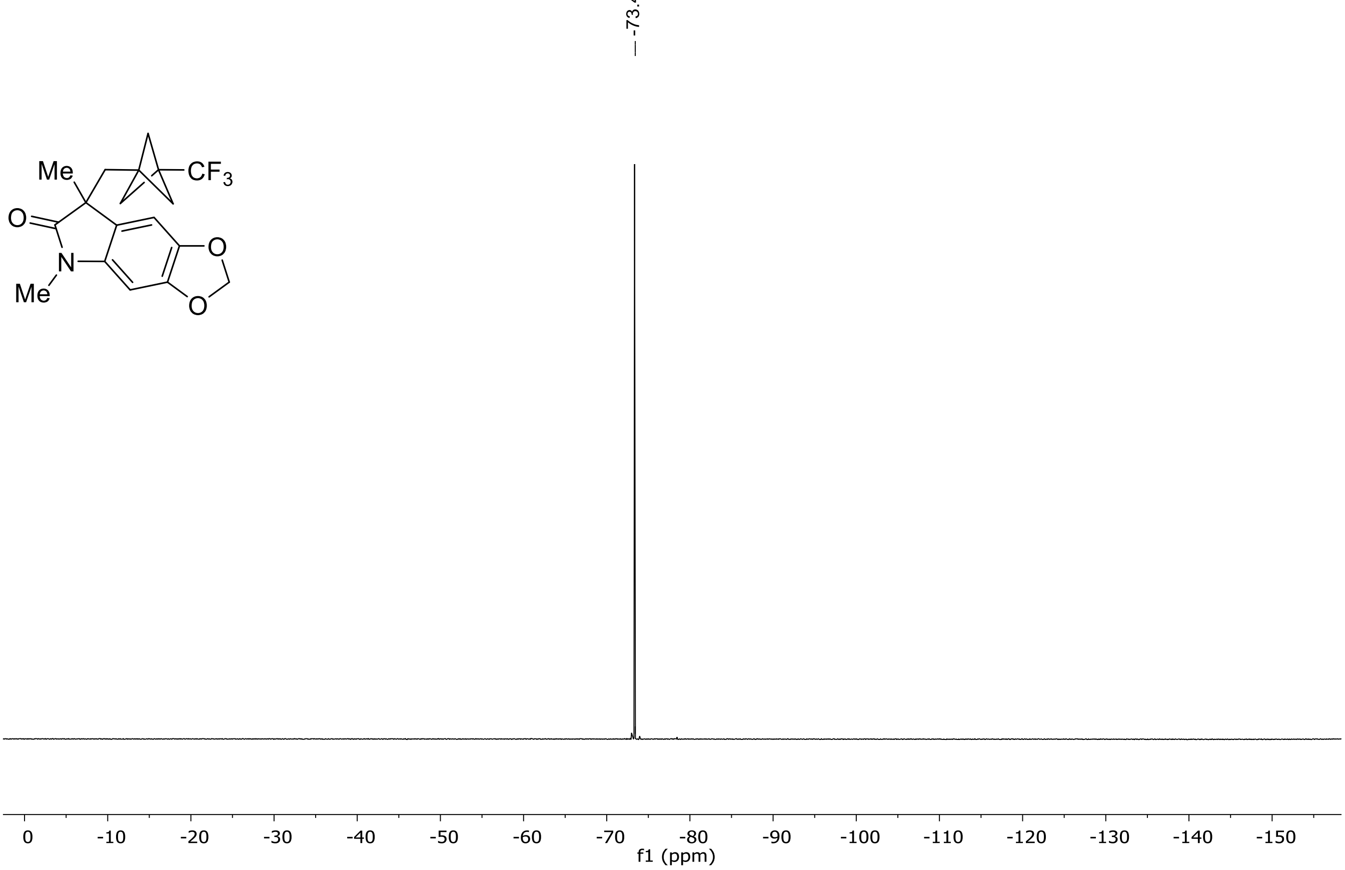

^{19}F NMR ($CDCl_3$, 282 MHz) of compound **14.**

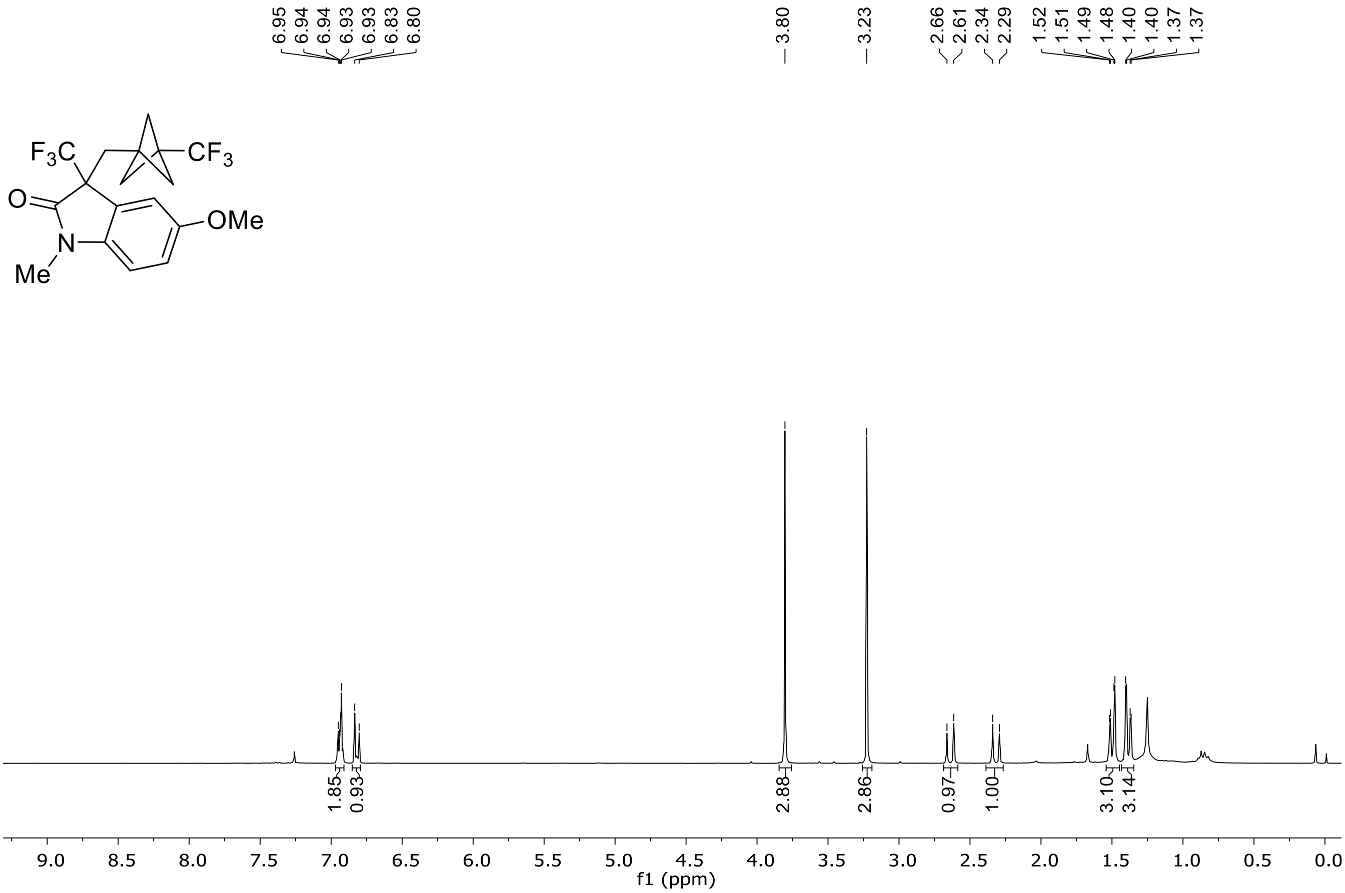

^{1}H NMR ($CDCl_3$, 300 MHz) of compound **15.**

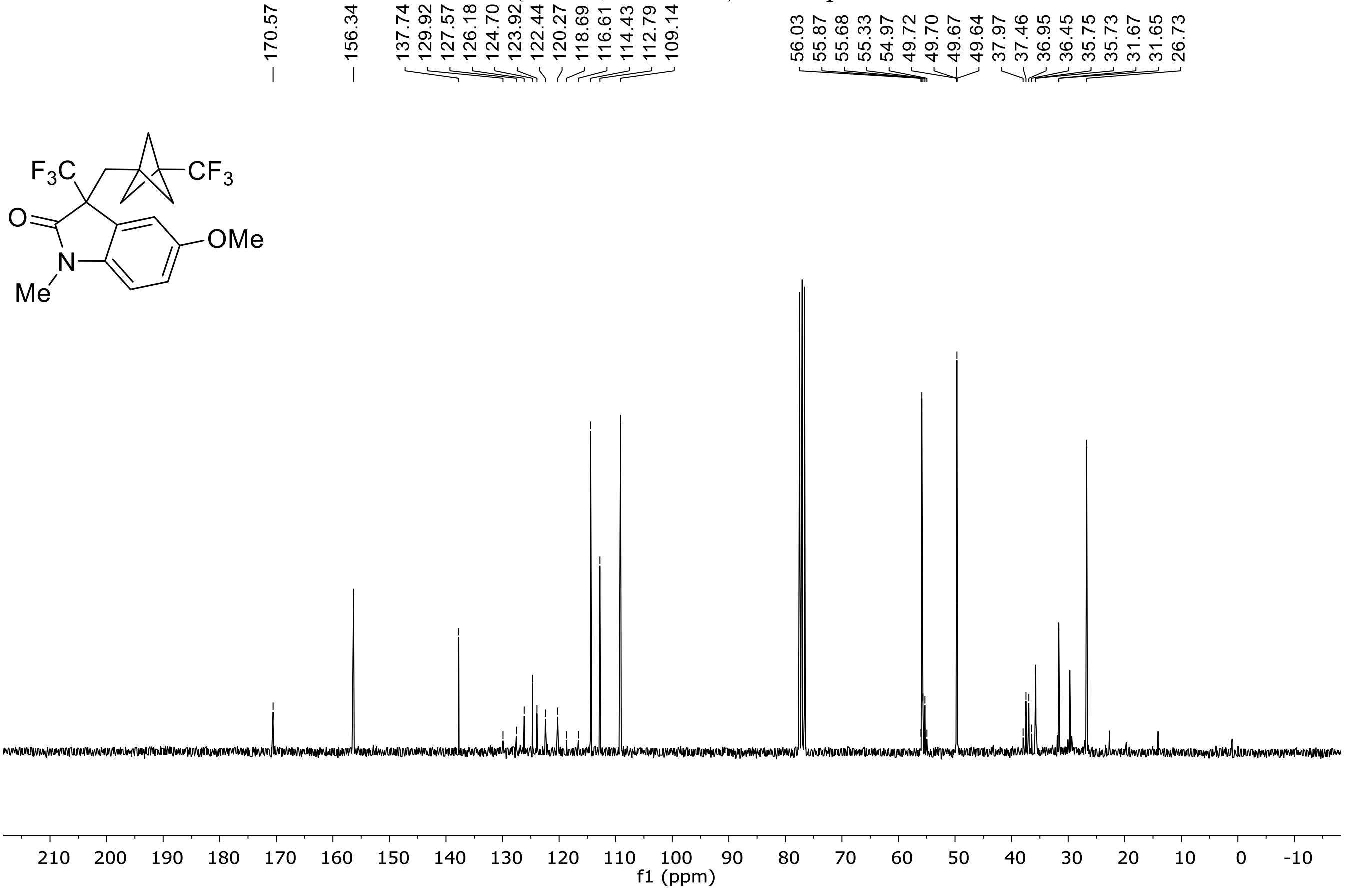

^{13}C NMR ($CDCl_3$, 75 MHz) of compound **15.**

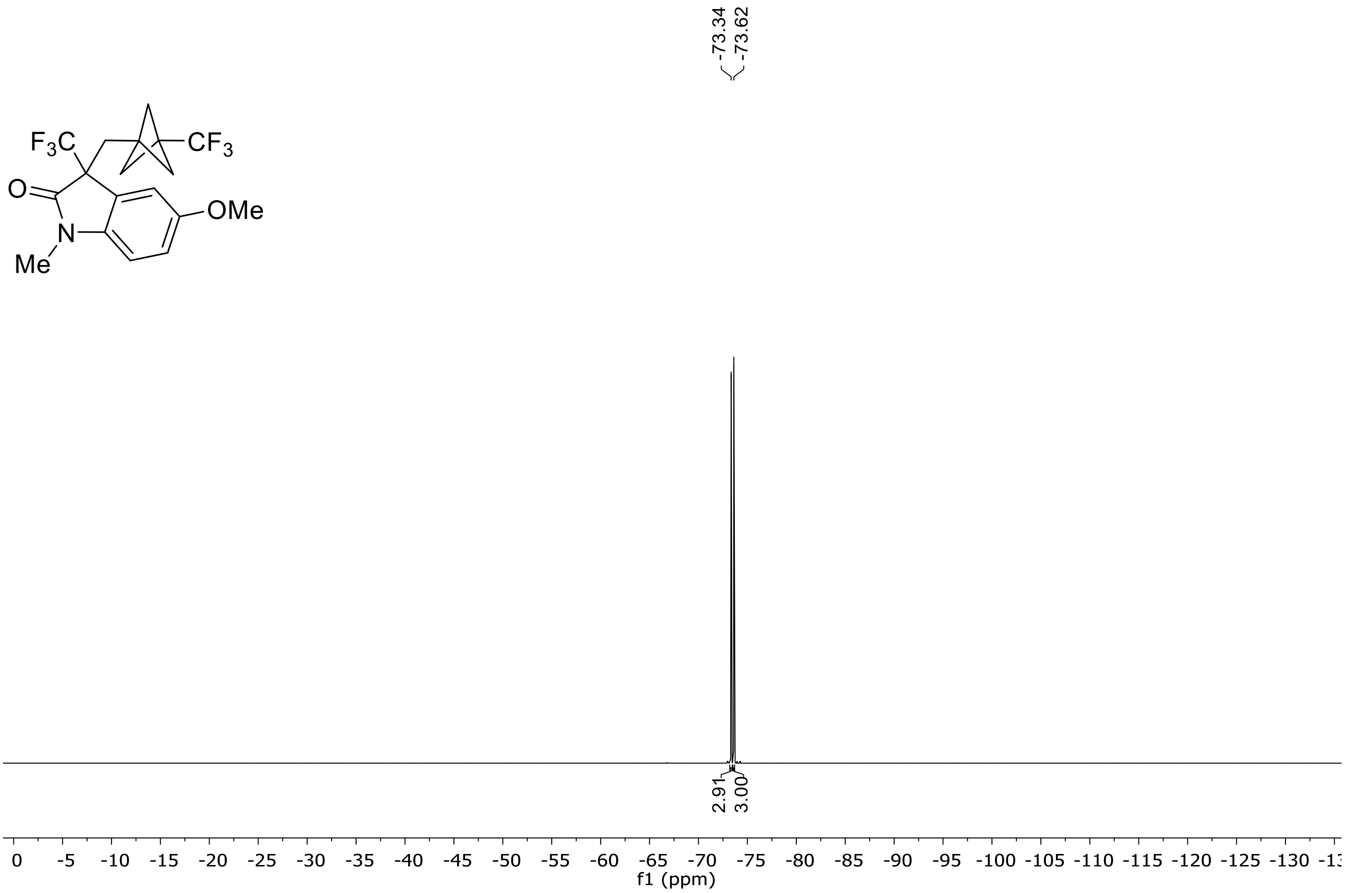

^{19}F NMR (CDCl$_3$, 282 MHz) of compound **15.**

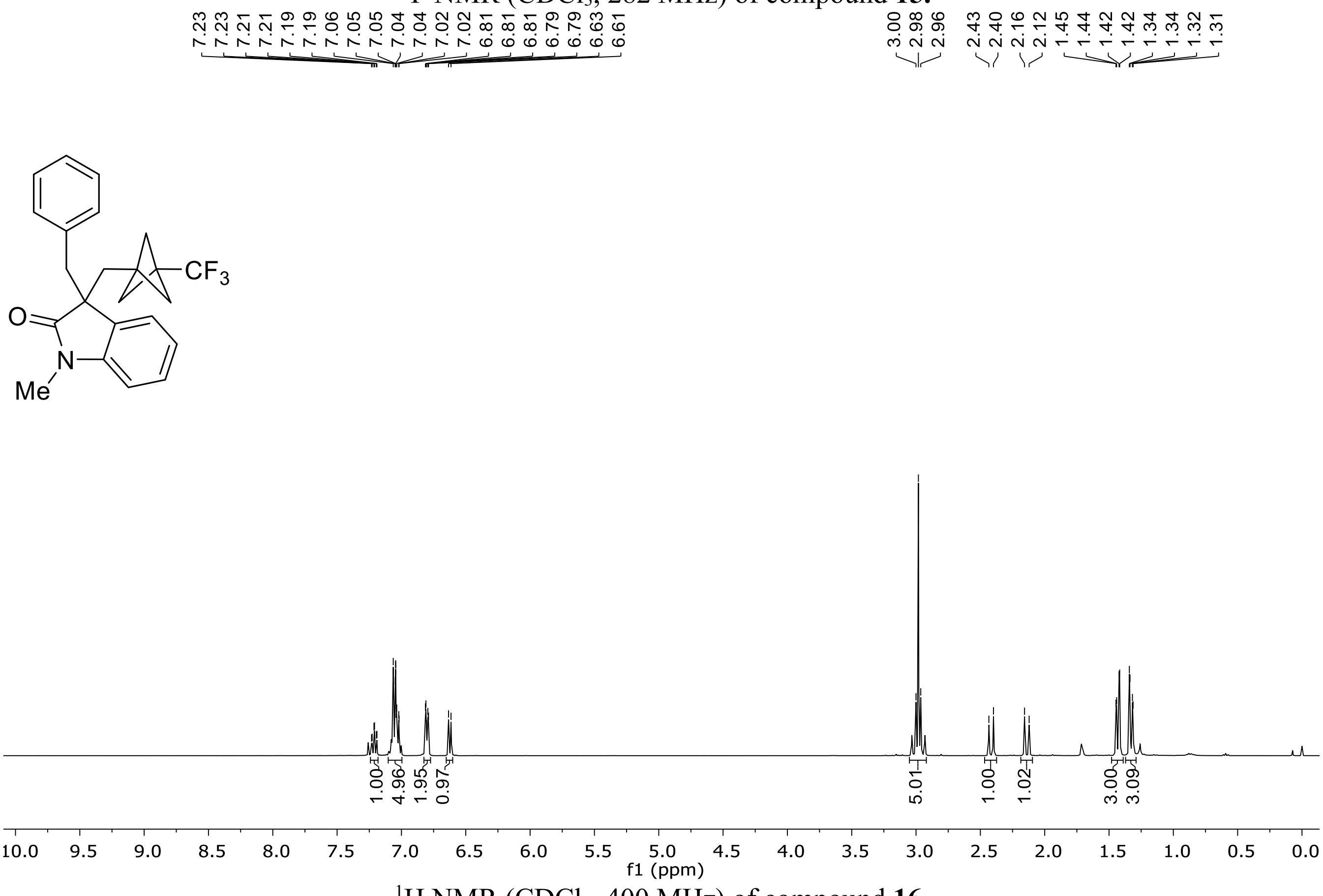

^{1}H NMR (CDCl$_3$, 400 MHz) of compound **16.**

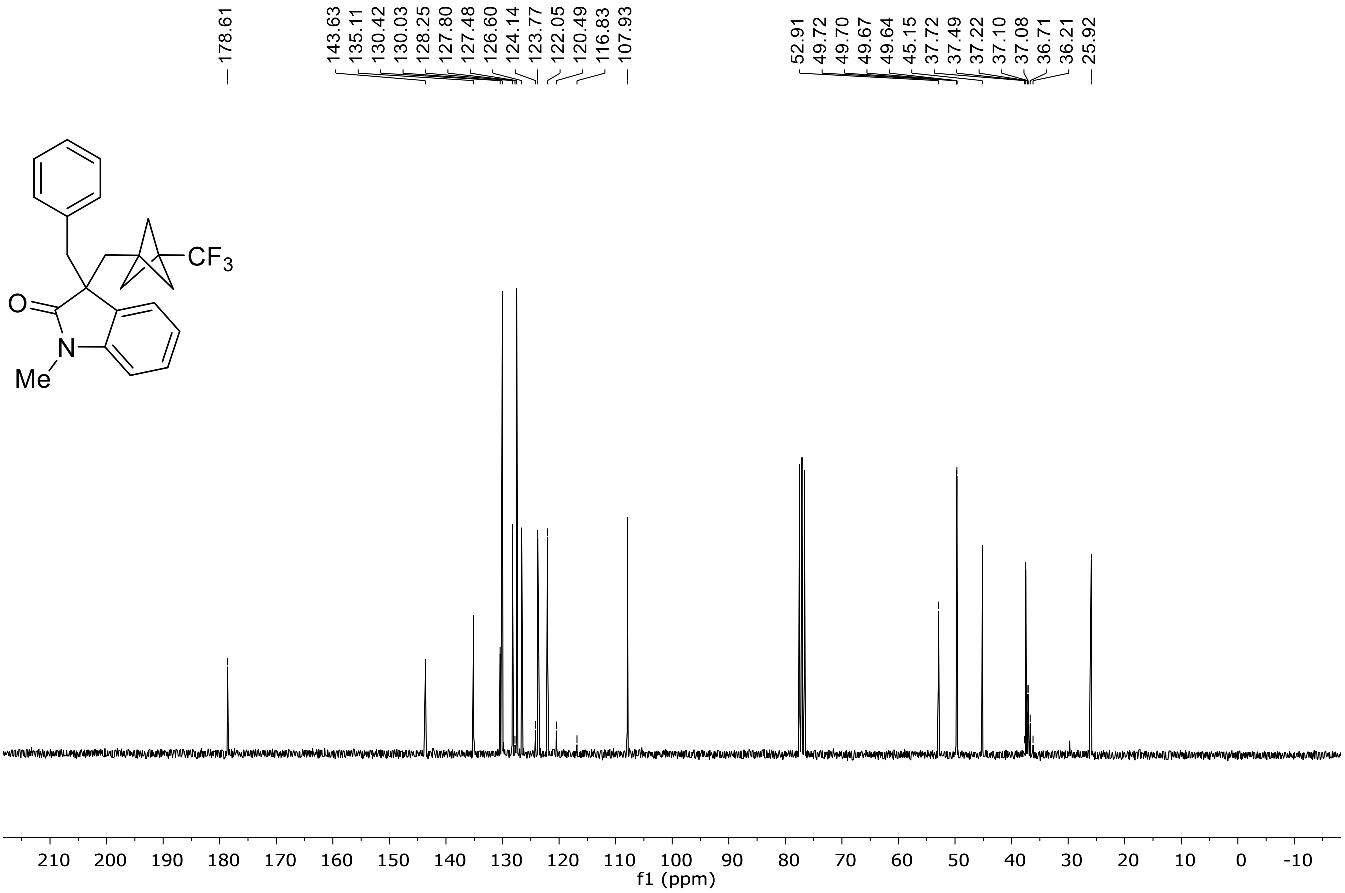

^{13}C NMR ($CDCl_3$, 75 MHz) of compound **16.**

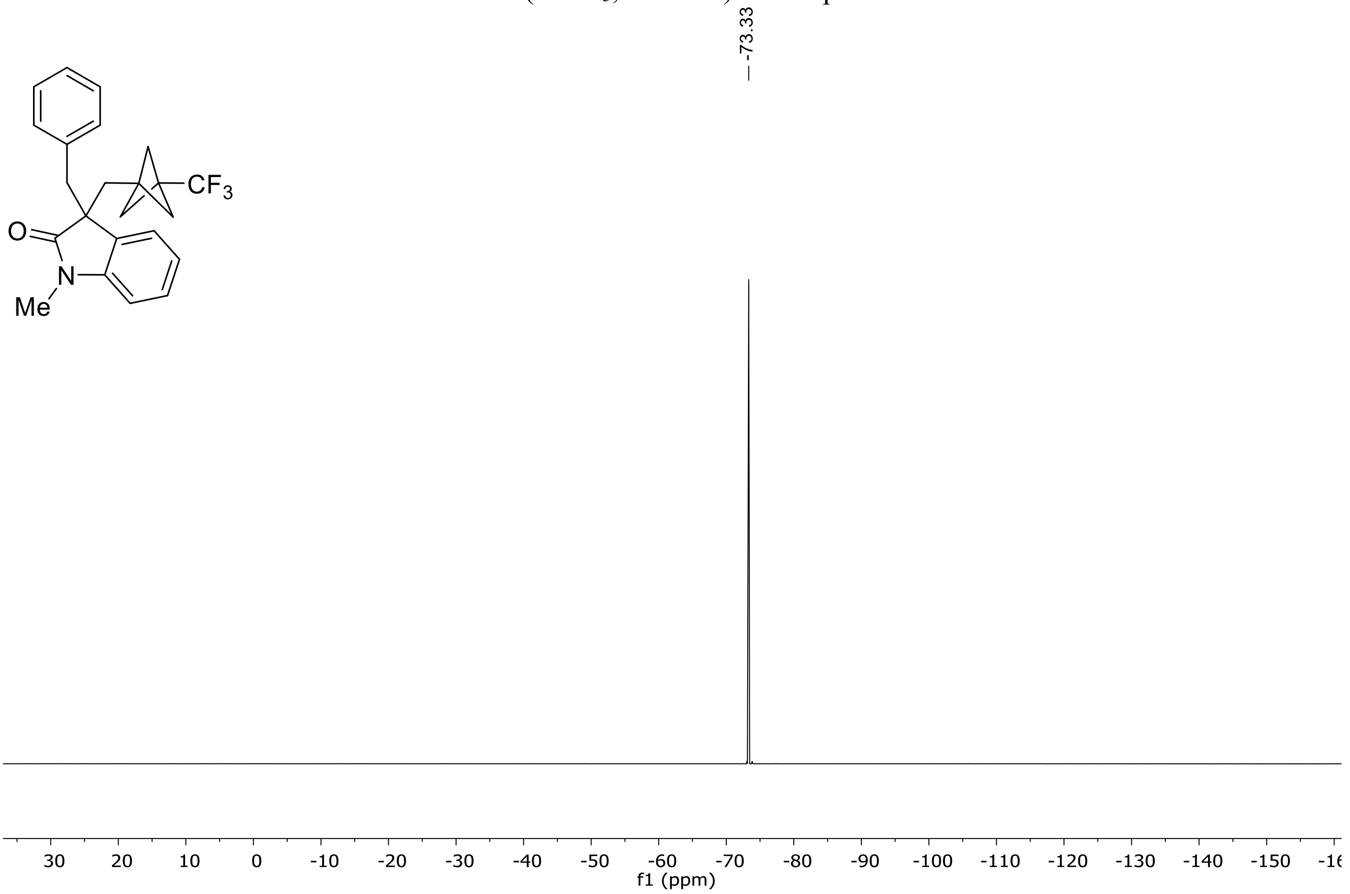

^{19}F NMR ($CDCl_3$, 376 MHz) of compound **16.**

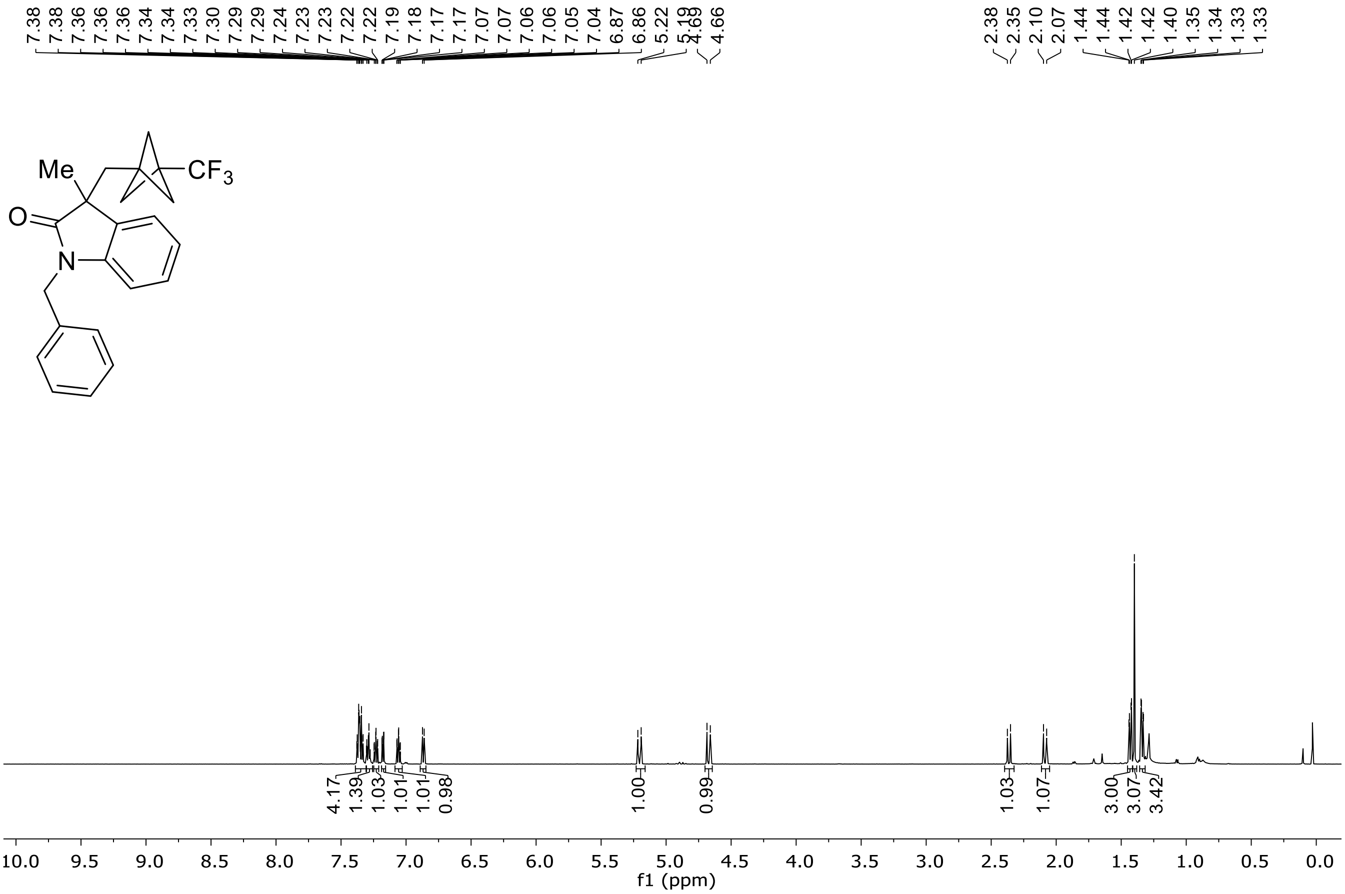

^{1}H NMR ($CDCl_3$, 600 MHz) of compound **17.**

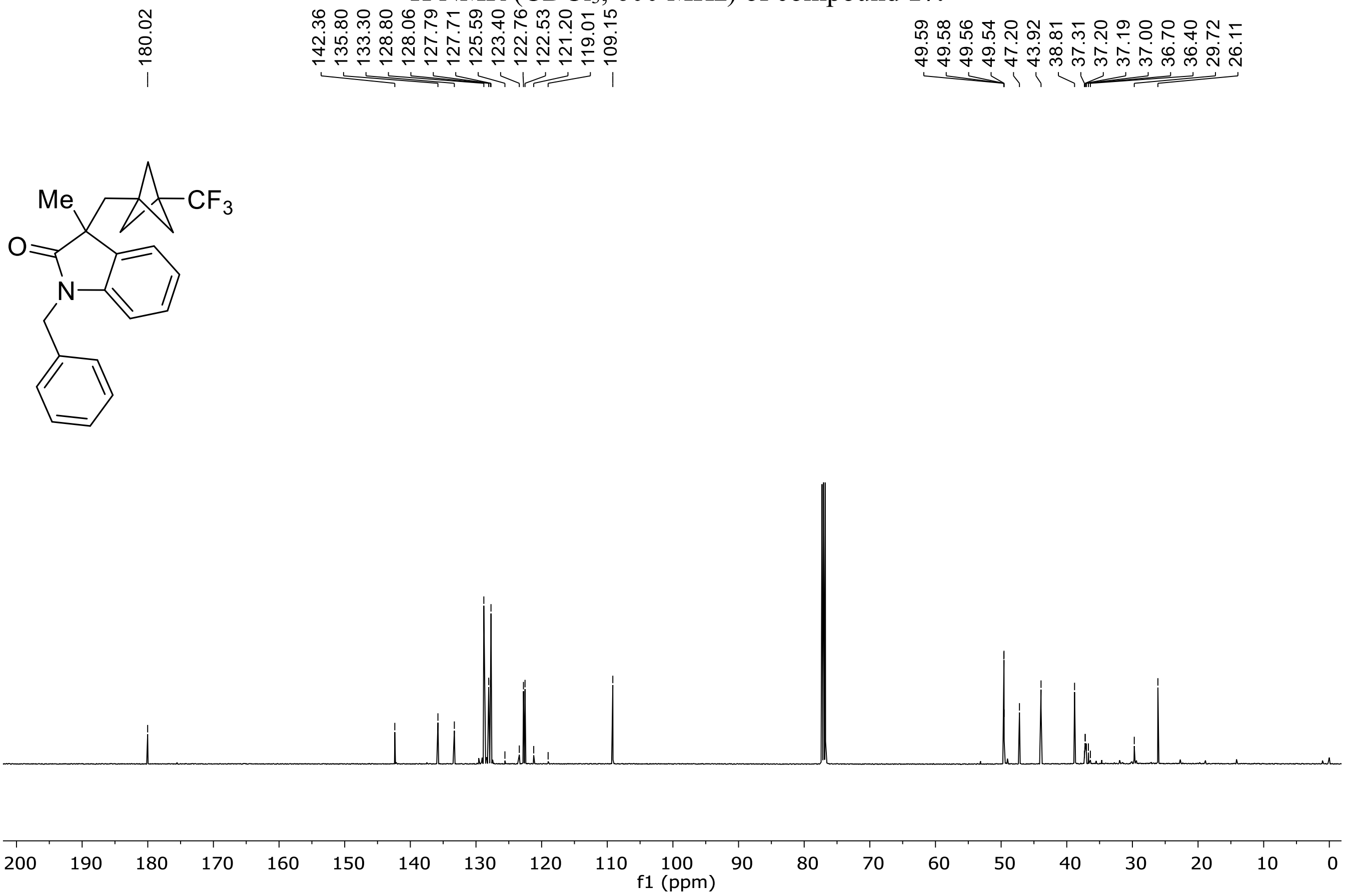

^{13}C NMR ($CDCl_3$, 126 MHz) of compound **17.**

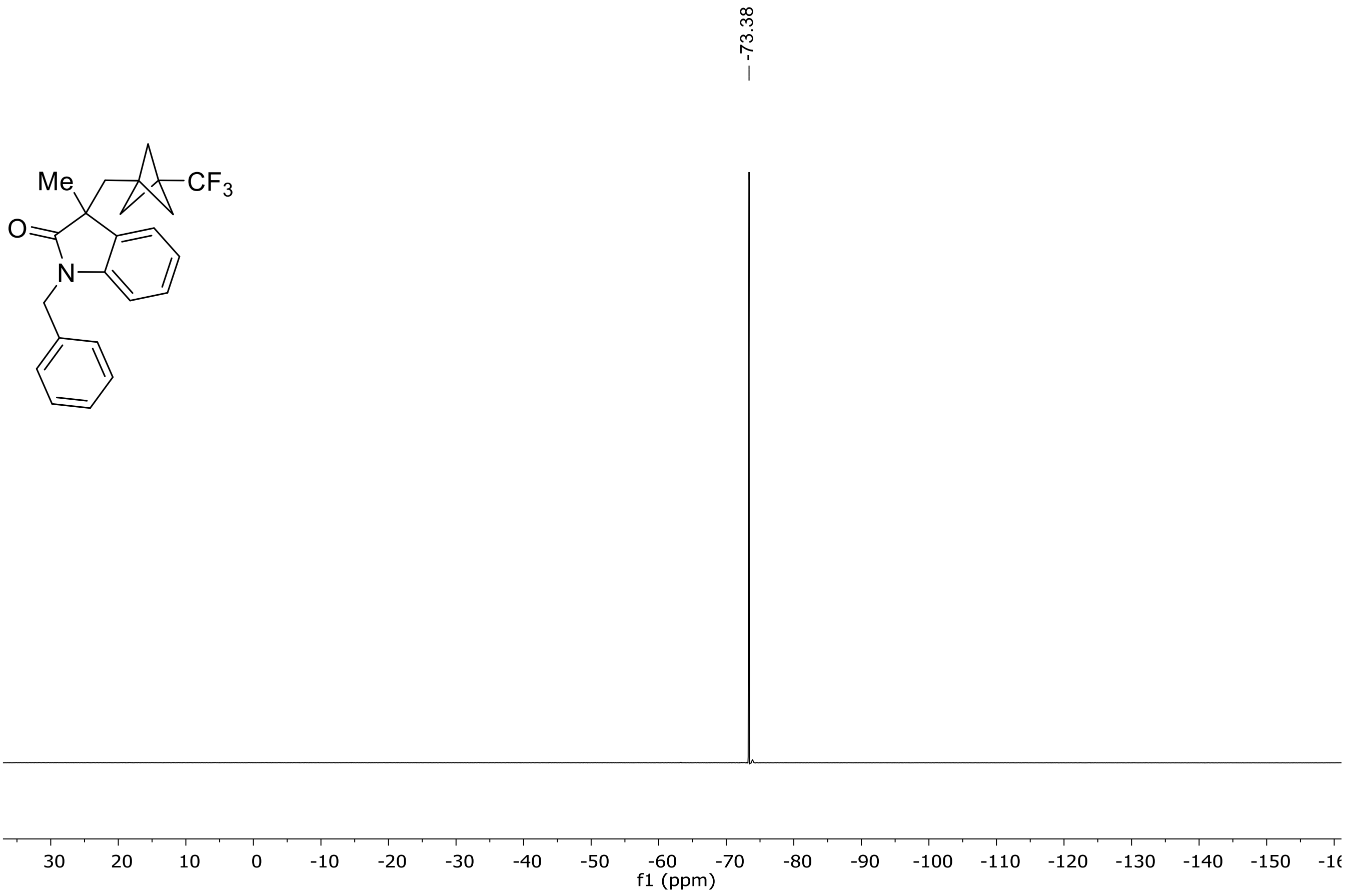

^{19}F NMR ($CDCl_3$, 376 MHz) of compound **17.**

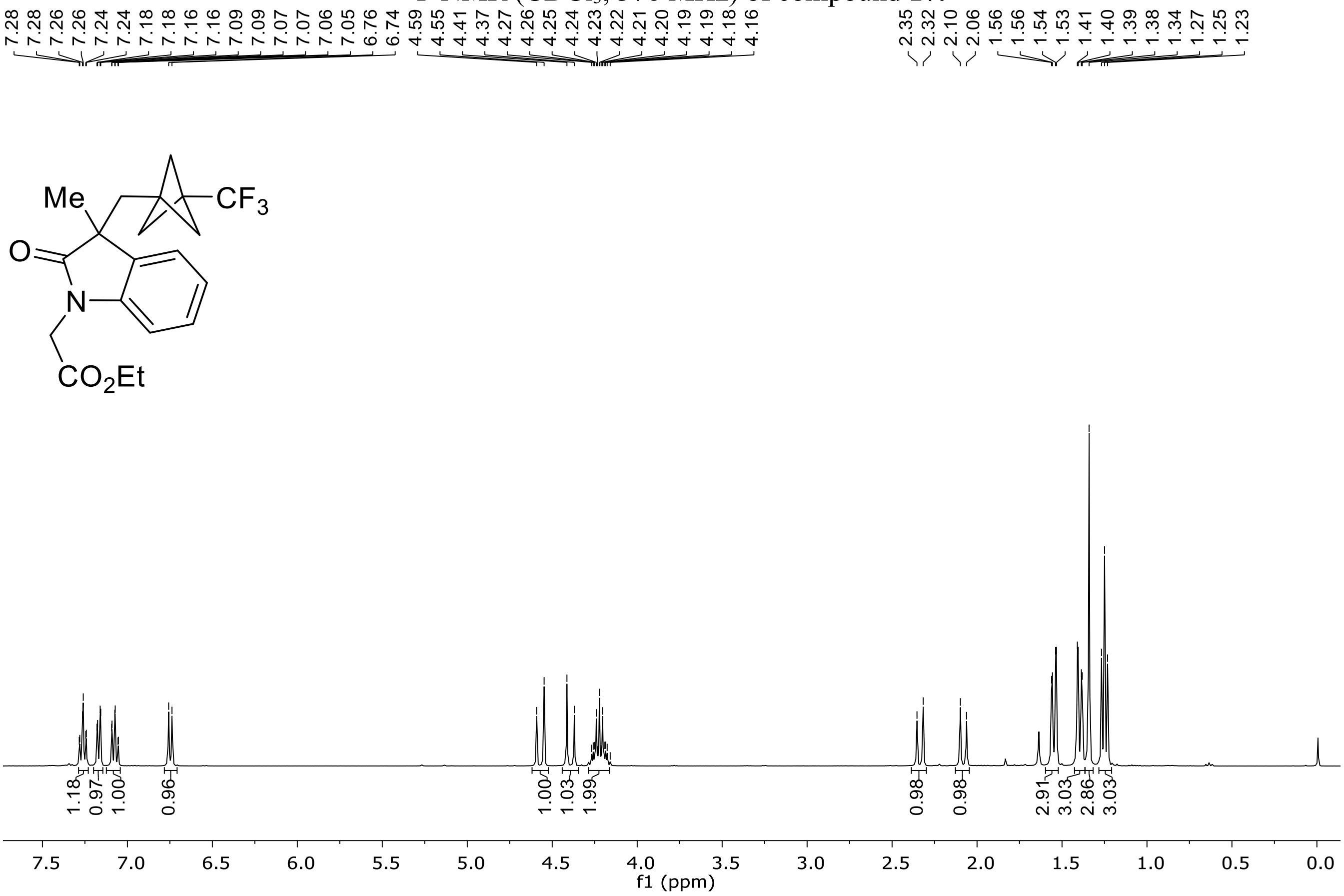

^{1}H NMR ($CDCl_3$, 400 MHz) of compound **18.**

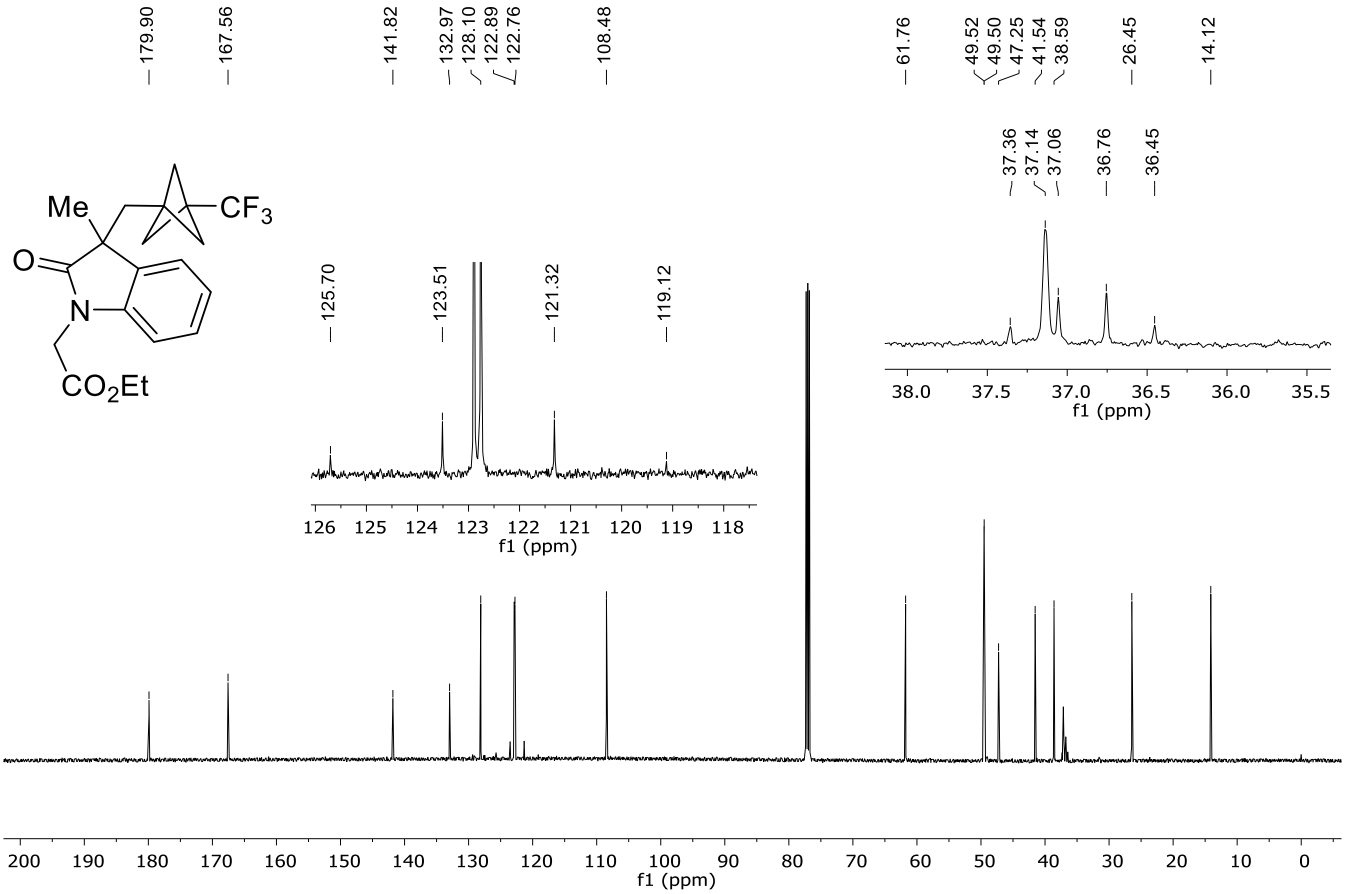

^{13}C NMR ($CDCl_3$, 126 MHz) of compound **18.**

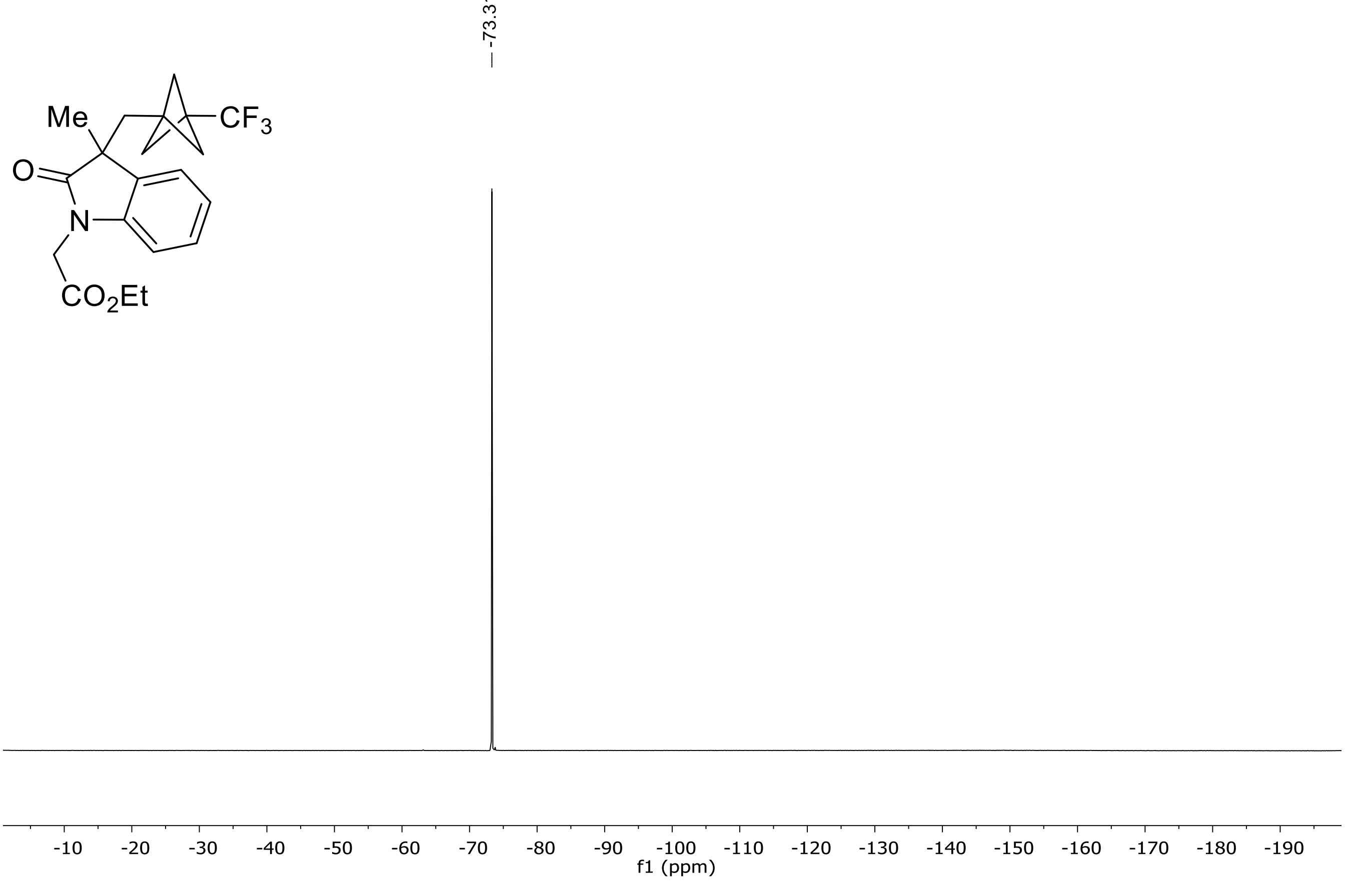

^{19}F NMR ($CDCl_3$, 377 MHz) of compound **18.**

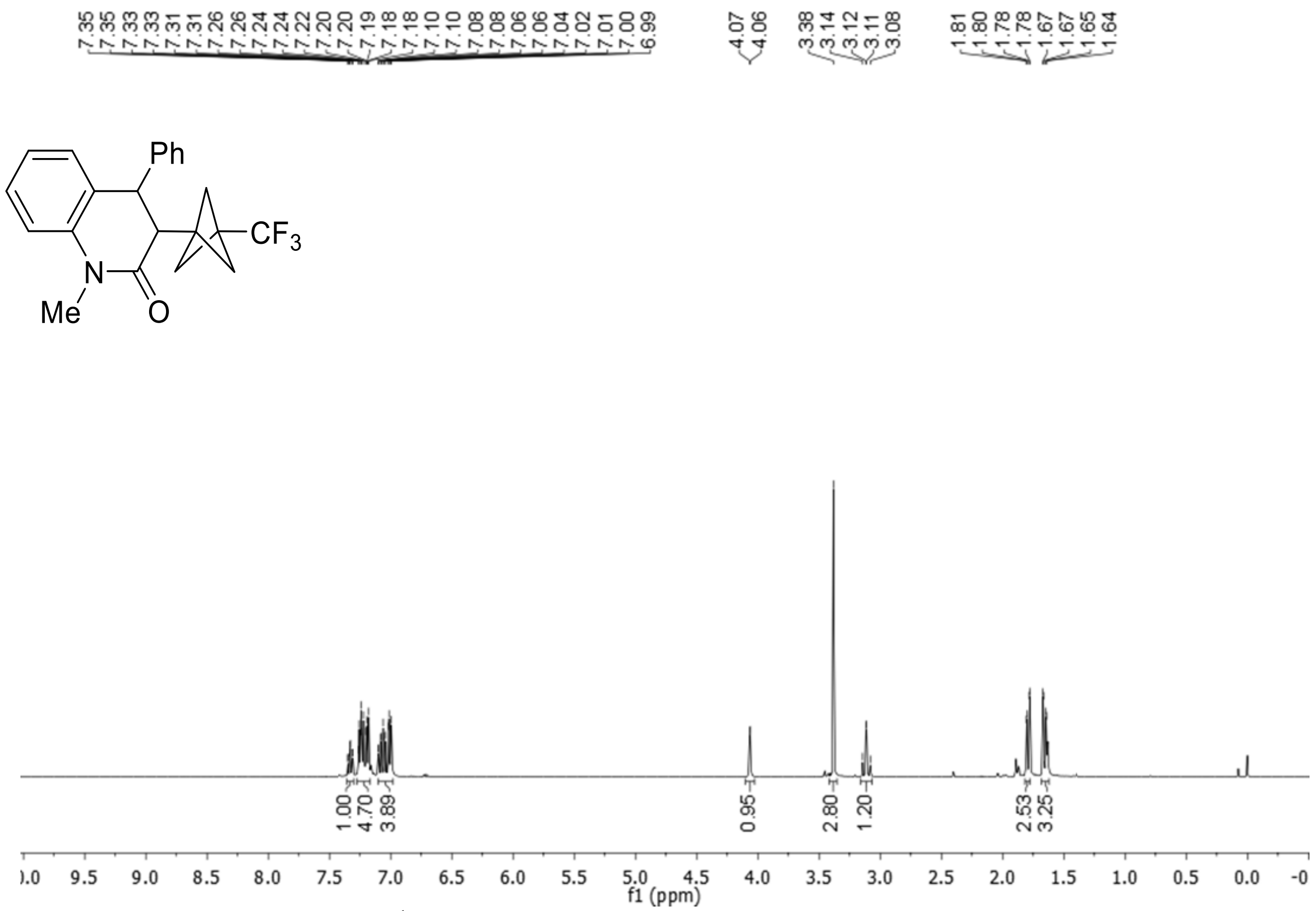

^{1}H NMR ($CDCl_3$, 300 MHz) of compound **19**

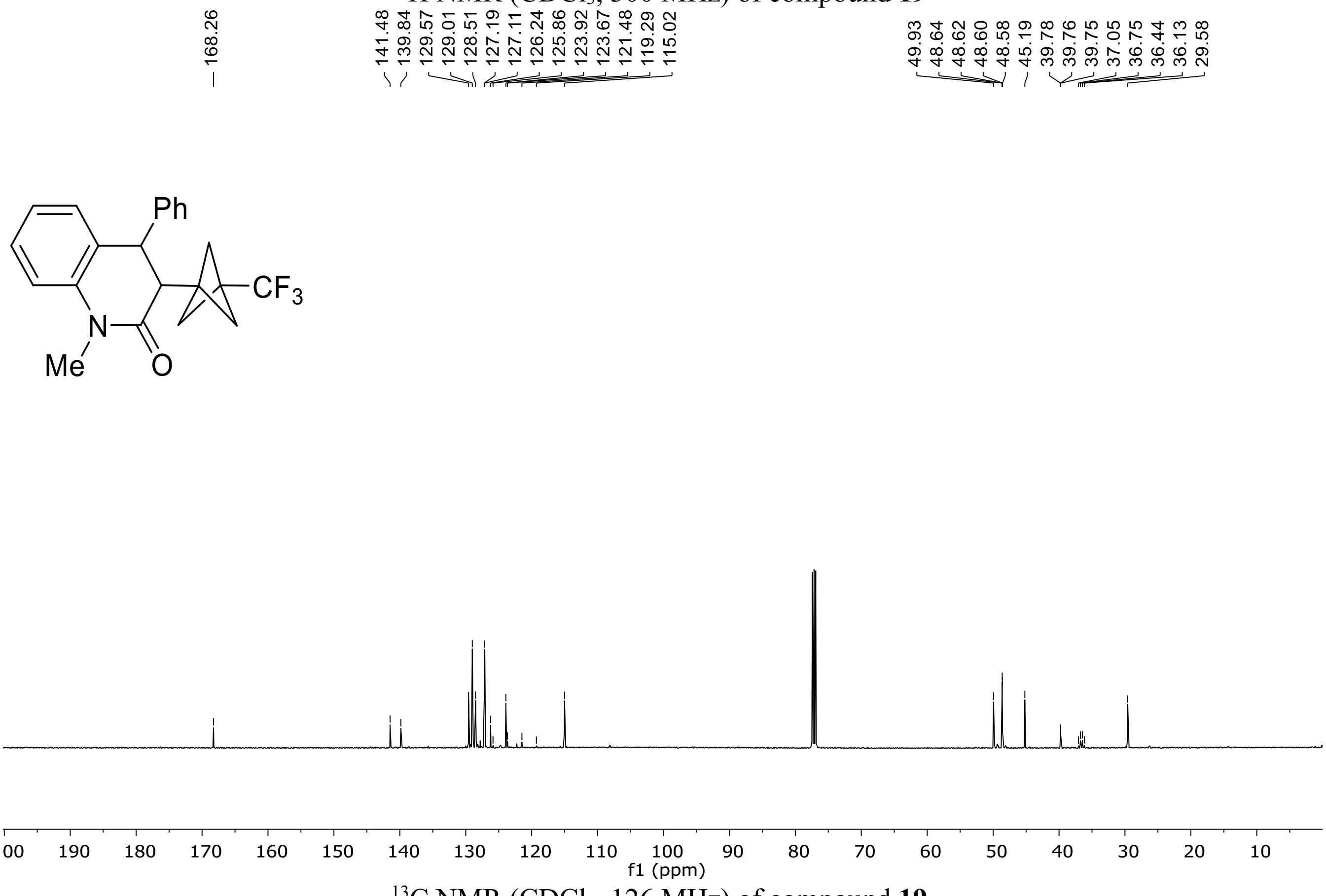

^{13}C NMR ($CDCl_3$, 126 MHz) of compound **19.**

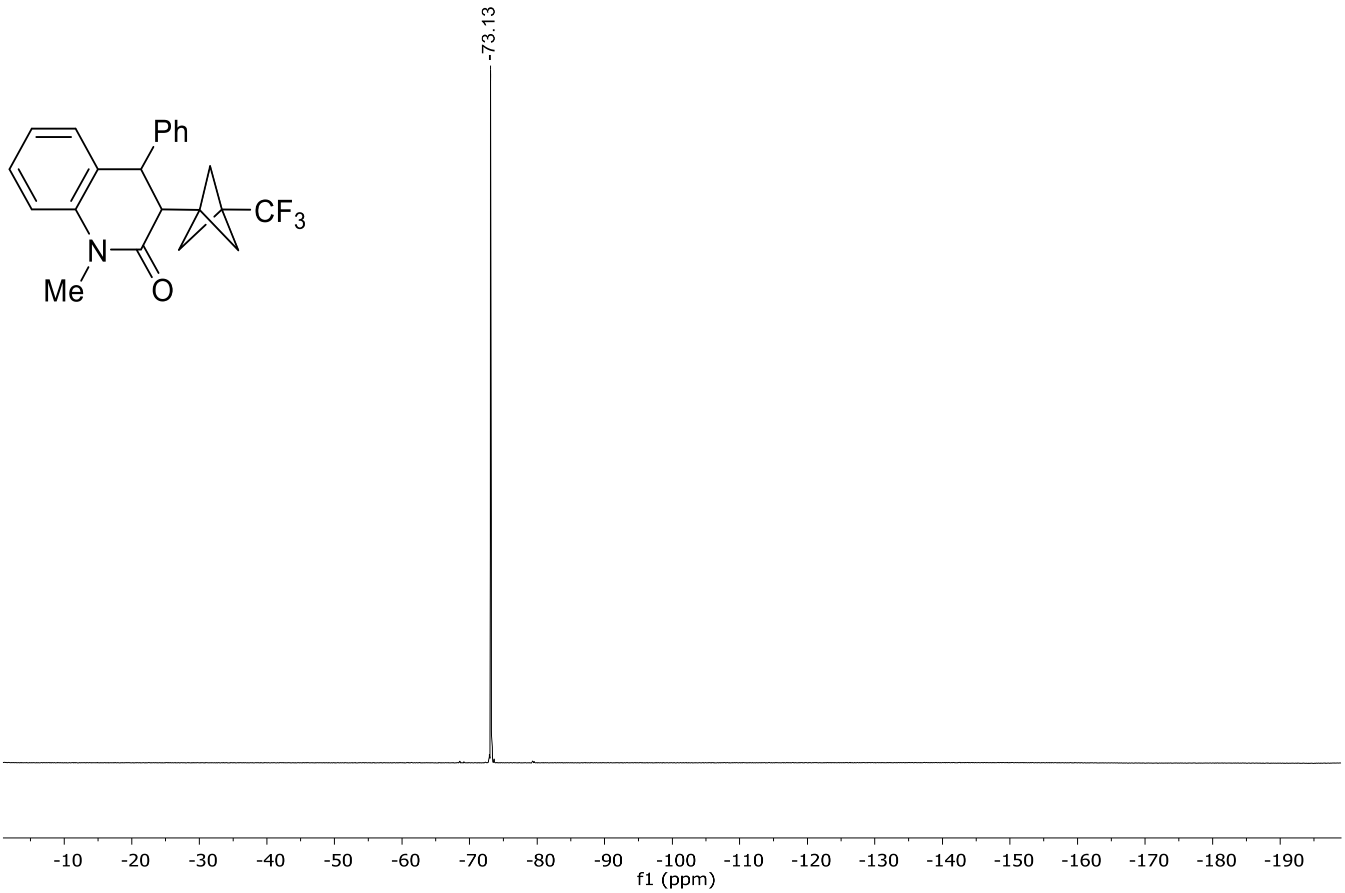

^{19}F NMR ($CDCl_3$, 282 MHz) of compound **19.**

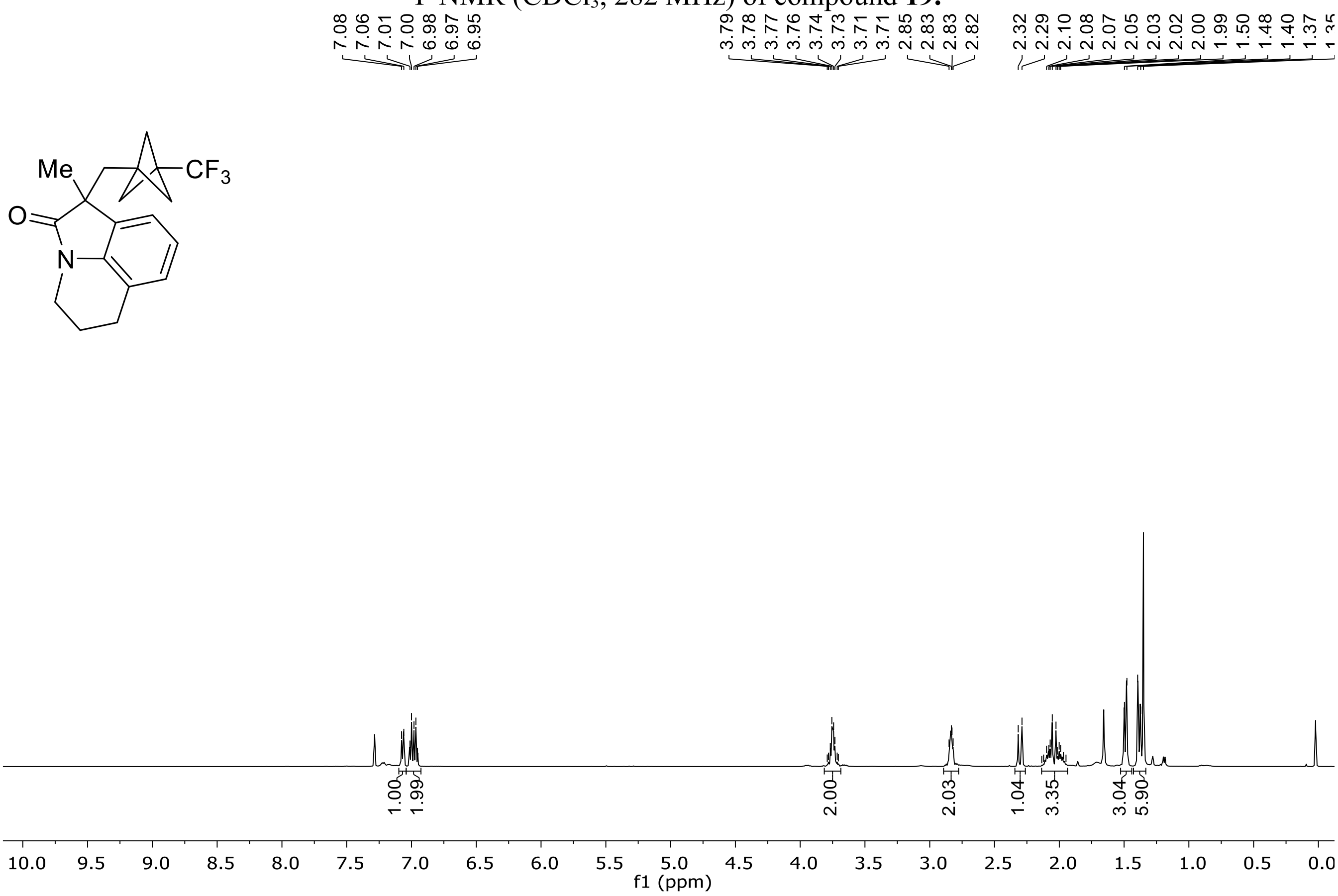

^{1}H NMR ($CDCl_3$, 500 MHz) of compound **20.**

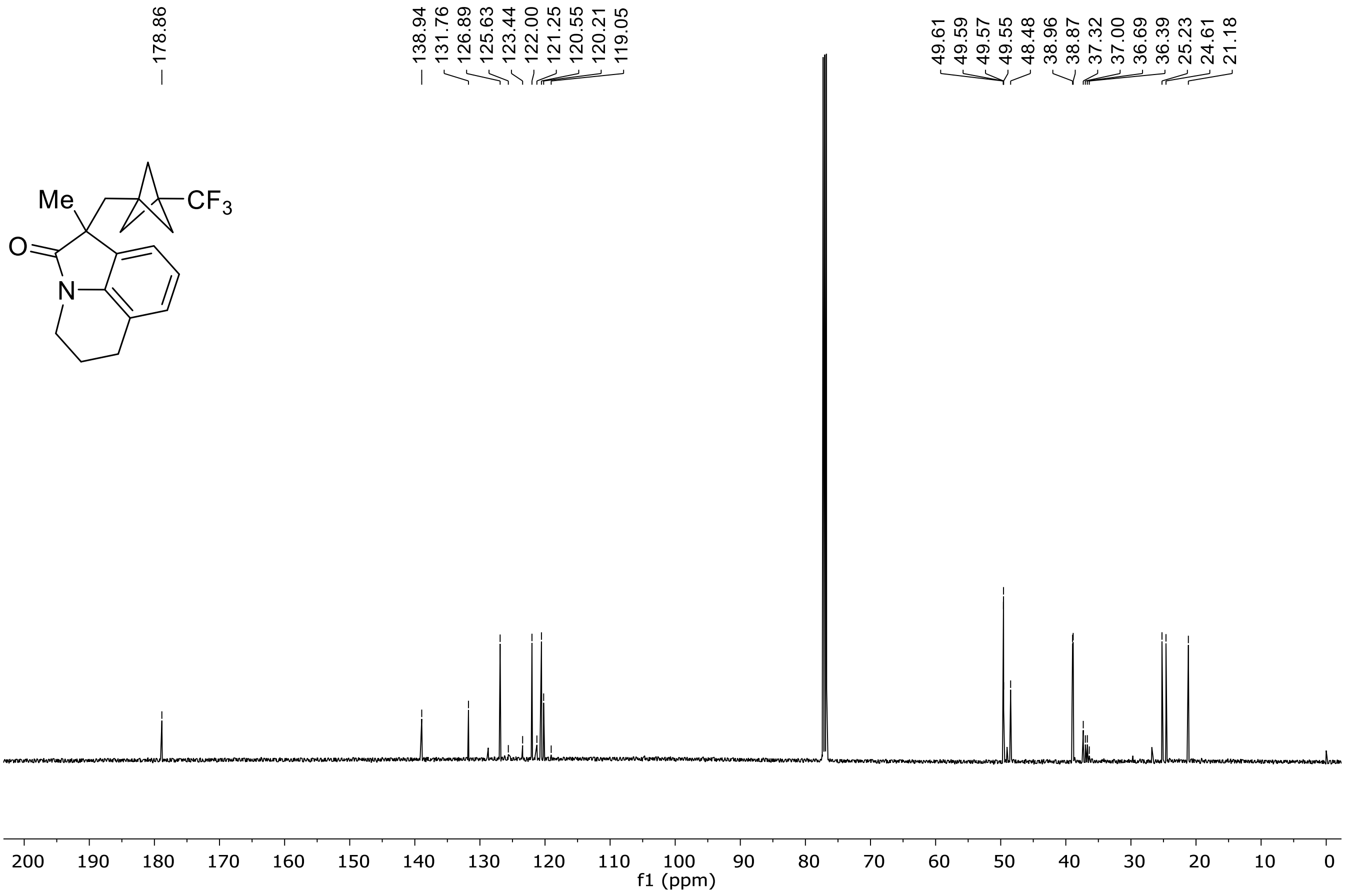

^{13}C NMR ($CDCl_3$, 126 MHz) of compound **20.**

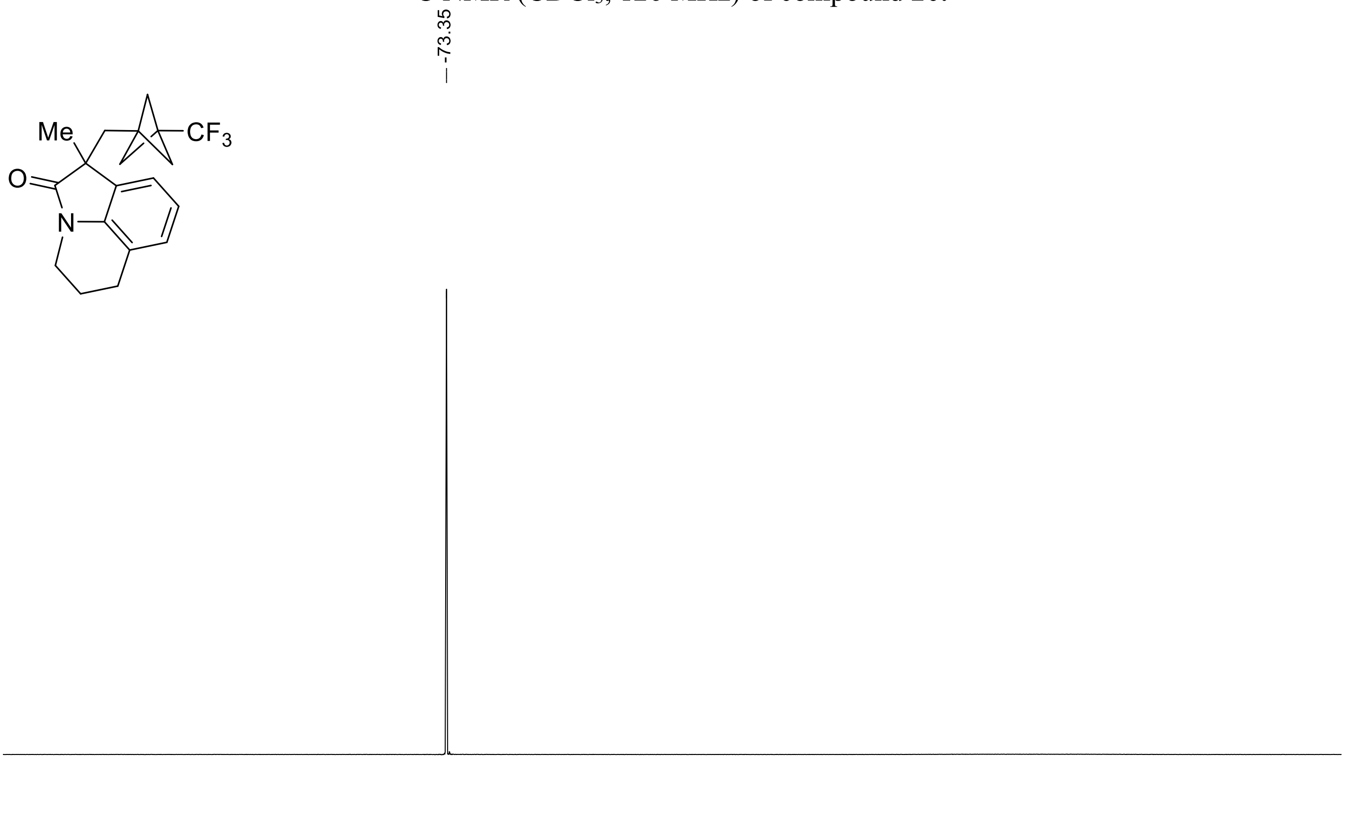

^{19}F NMR ($CDCl_3$, 282 MHz) of compound **20.**

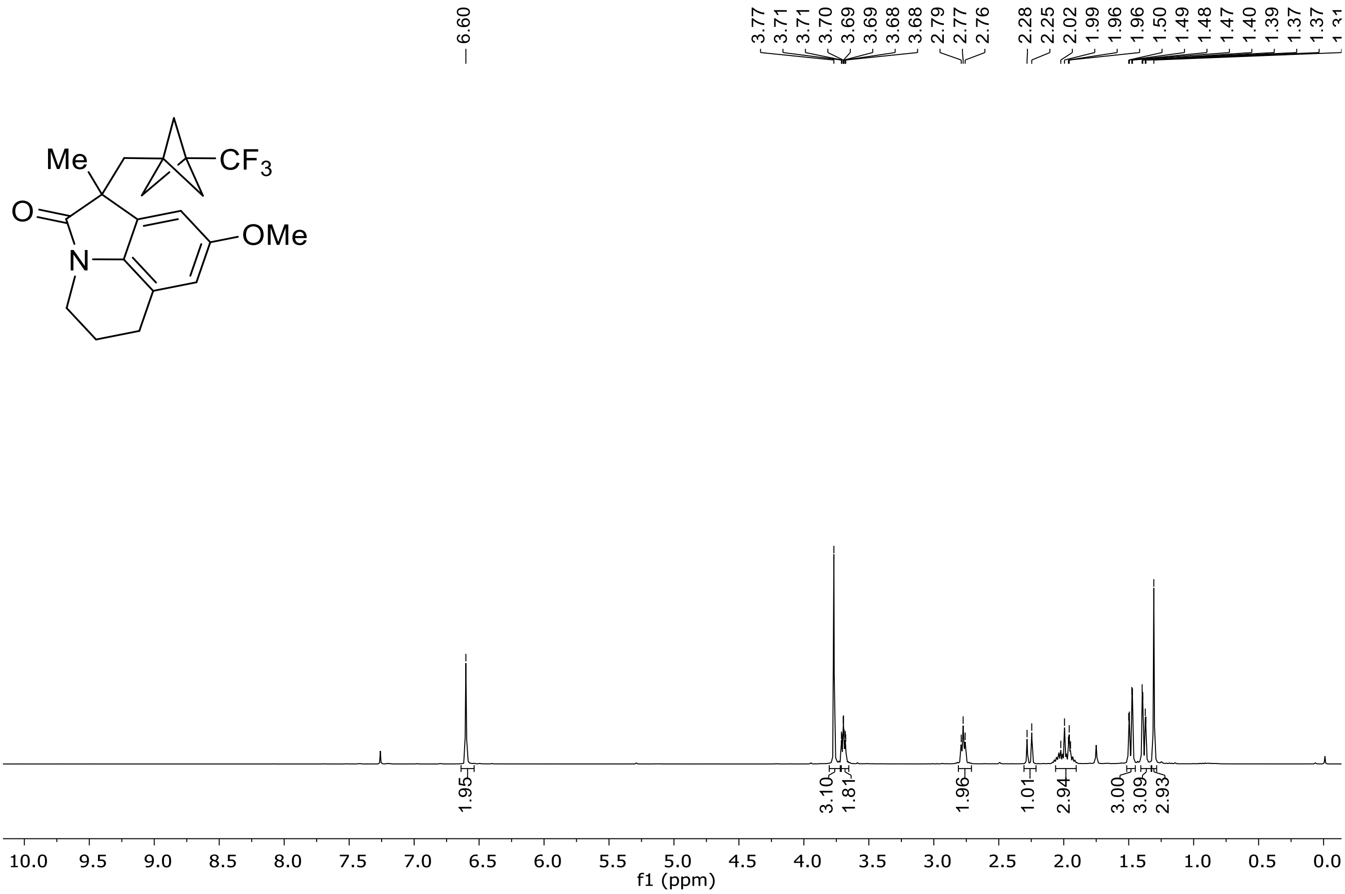

^{1}H NMR (CDCl$_3$, 400 MHz) of compound **21.**

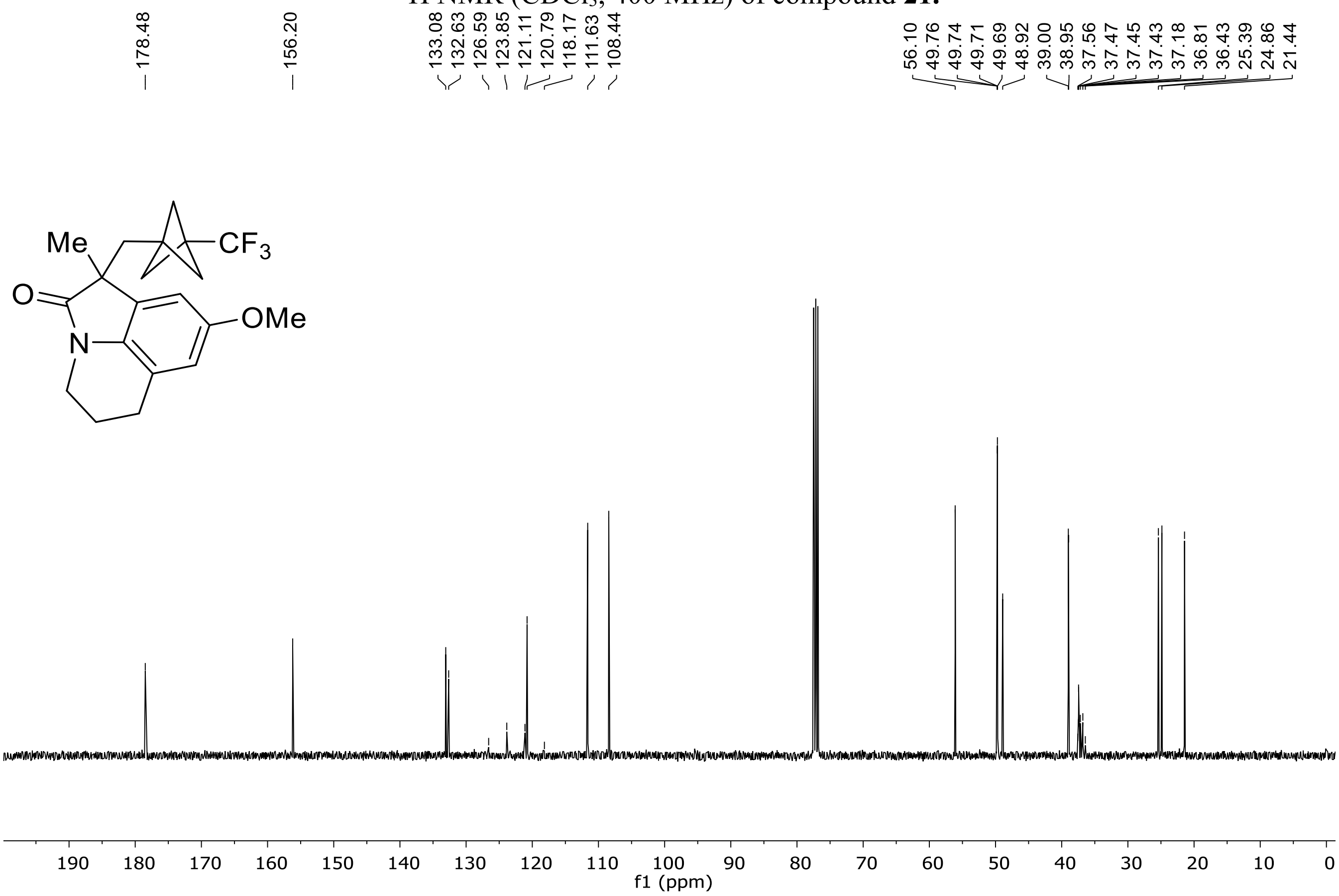

^{13}C NMR (CDCl$_3$, 101 MHz) of compound **21.**

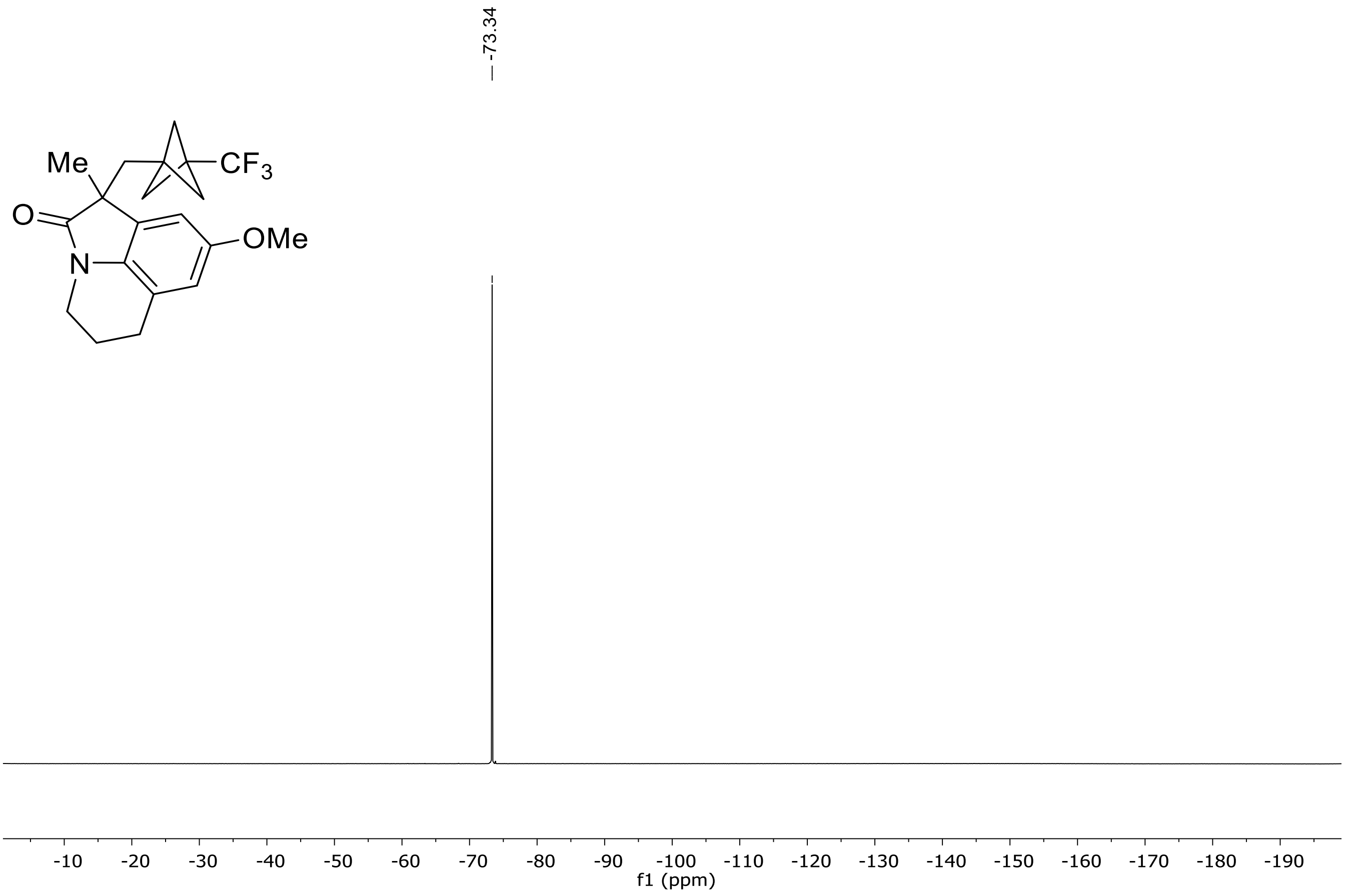

^{19}F NMR ($CDCl_3$, 282 MHz) of compound **21.**

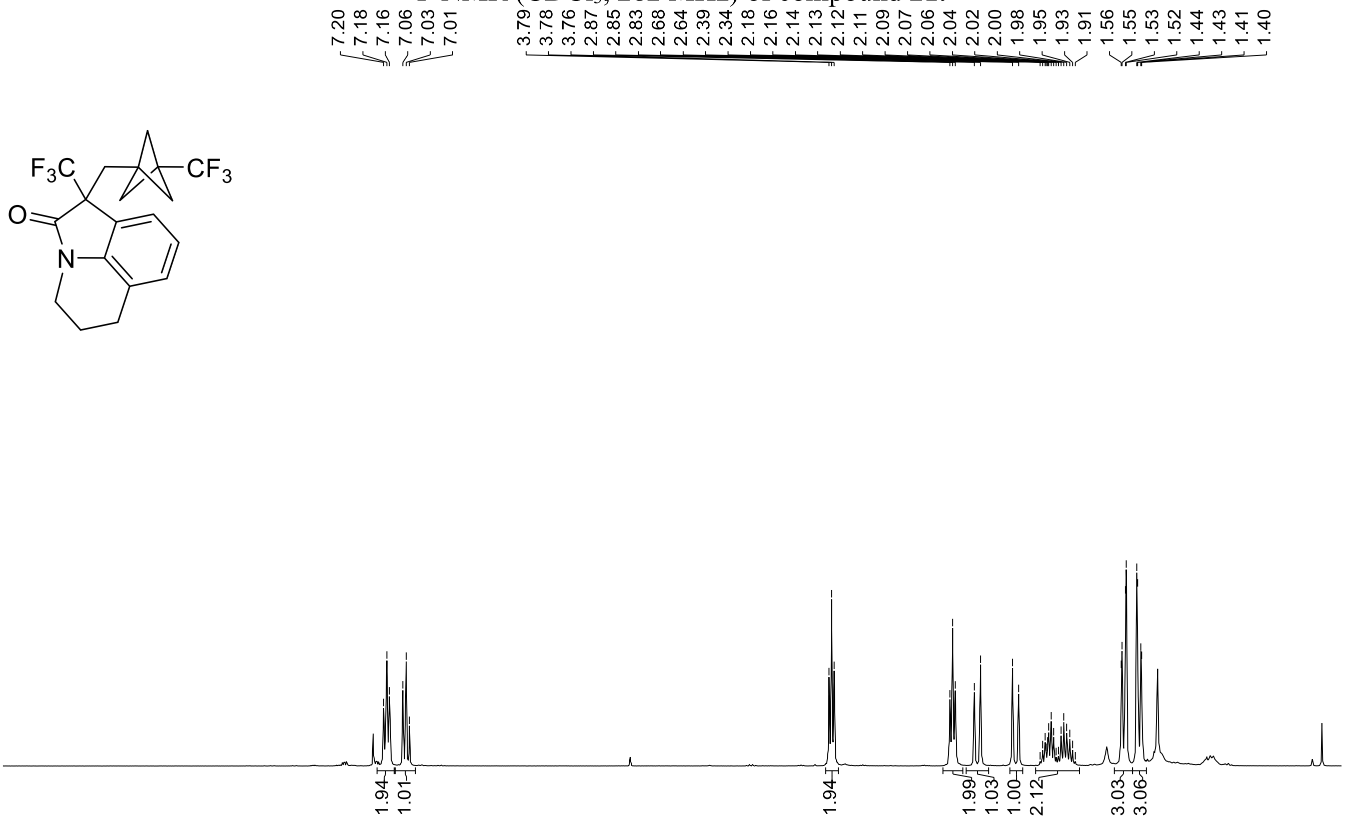

^{1}H NMR ($CDCl_3$, 300 MHz) of compound **22.**

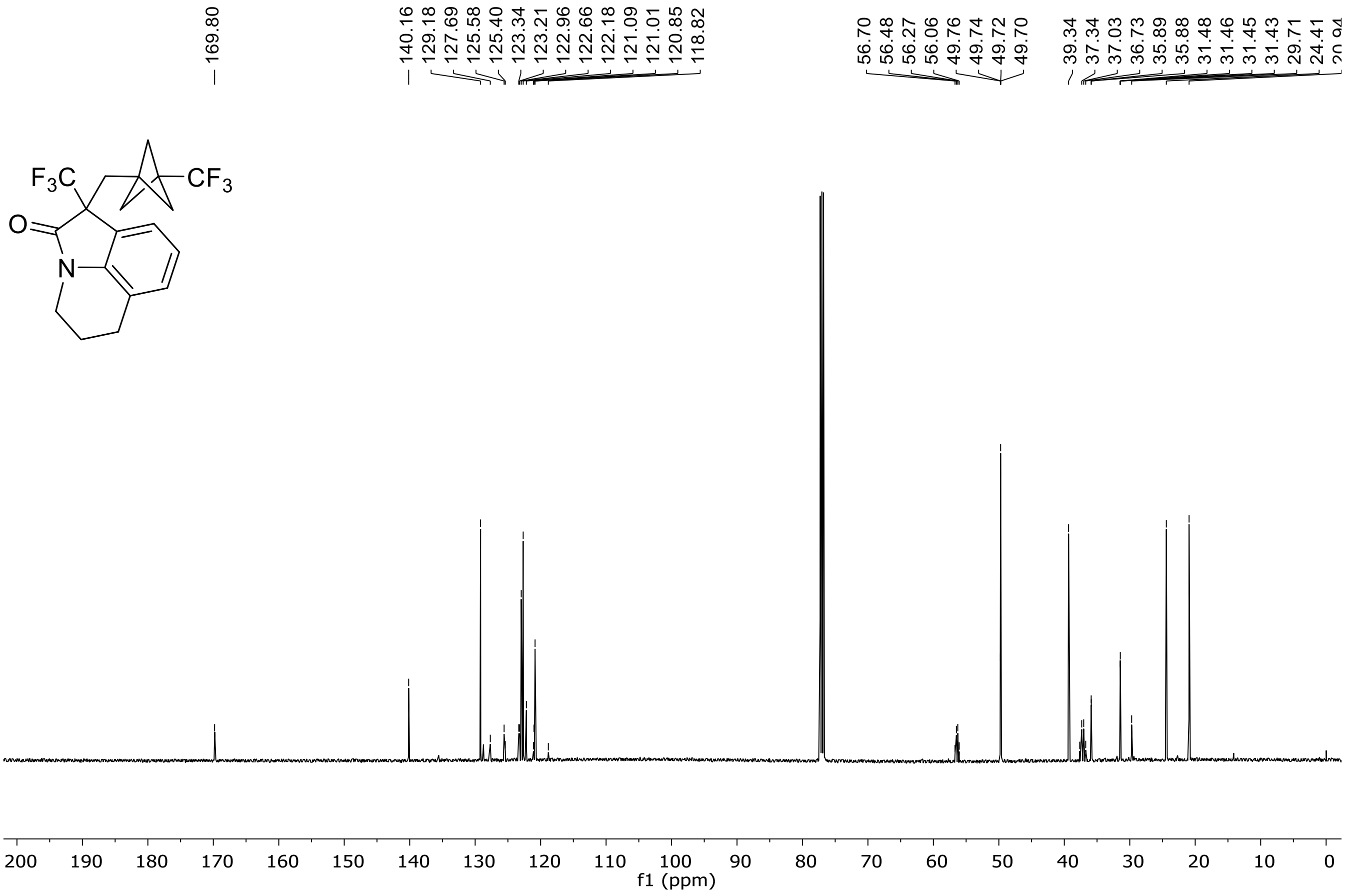

^{13}C NMR (CDCl$_3$, 126 MHz) of compound **22.**

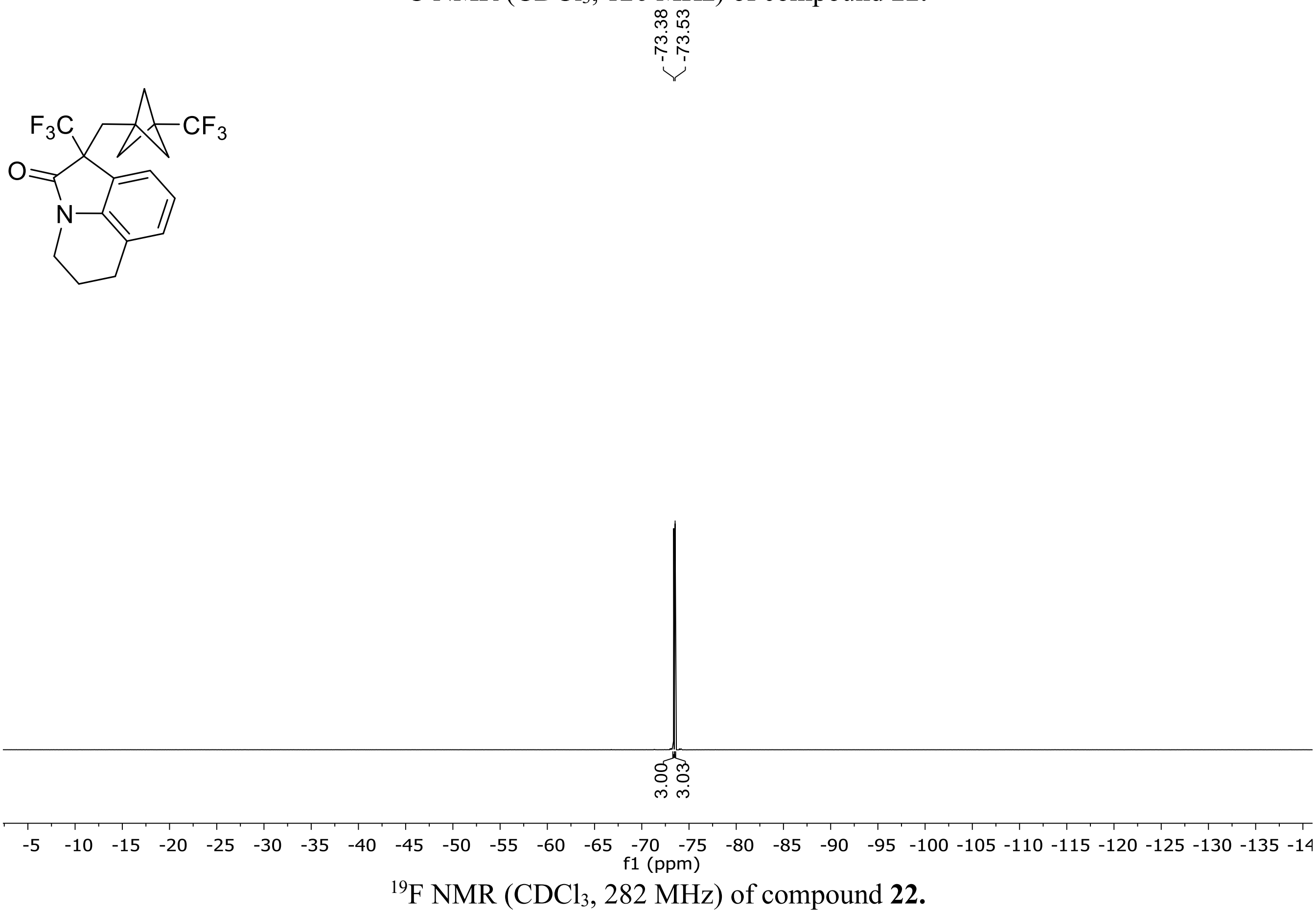

^{19}F NMR (CDCl$_3$, 282 MHz) of compound **22.**

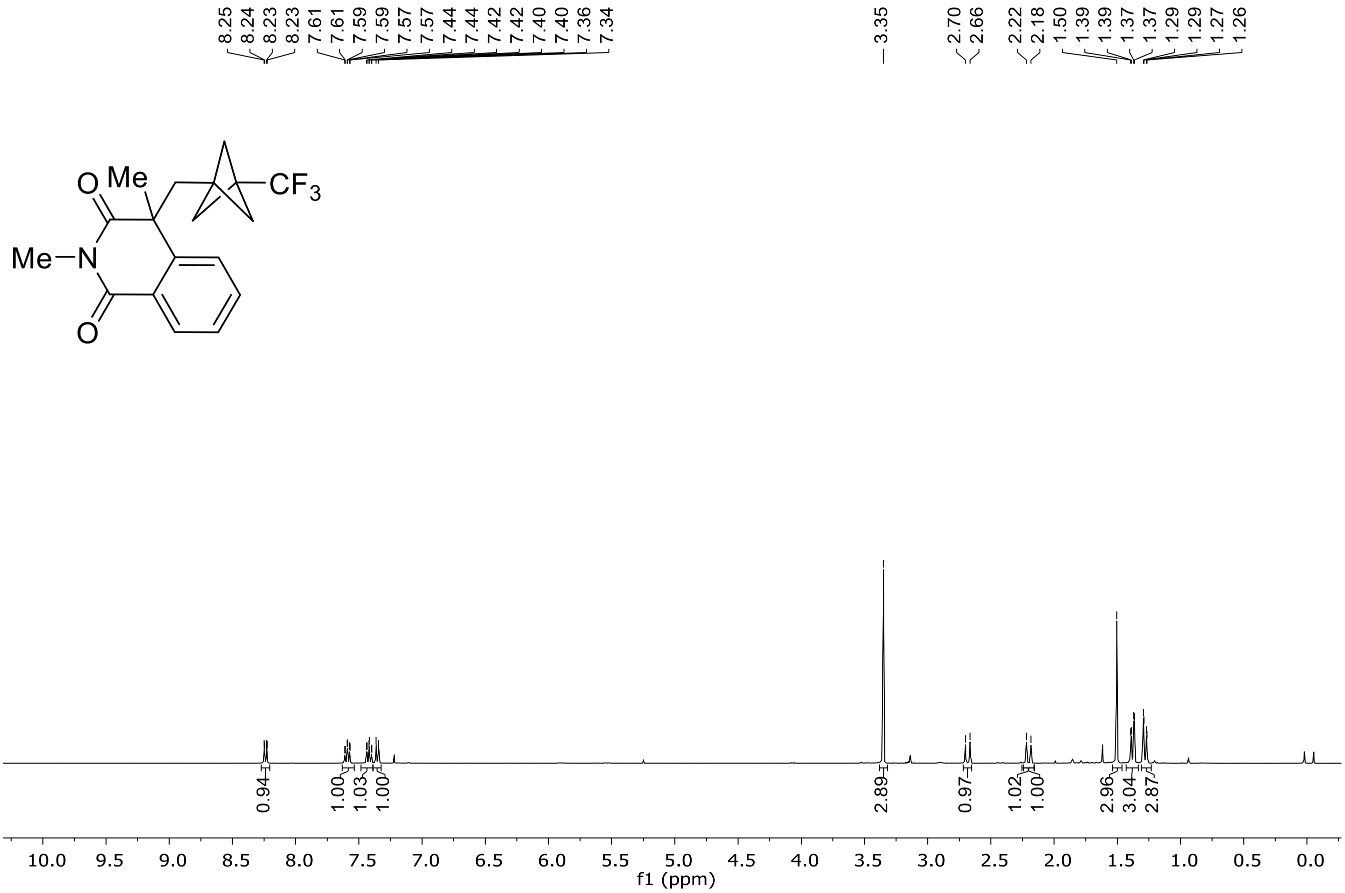

^{1}H NMR ($CDCl_3$, 400 MHz) of compound **23.**

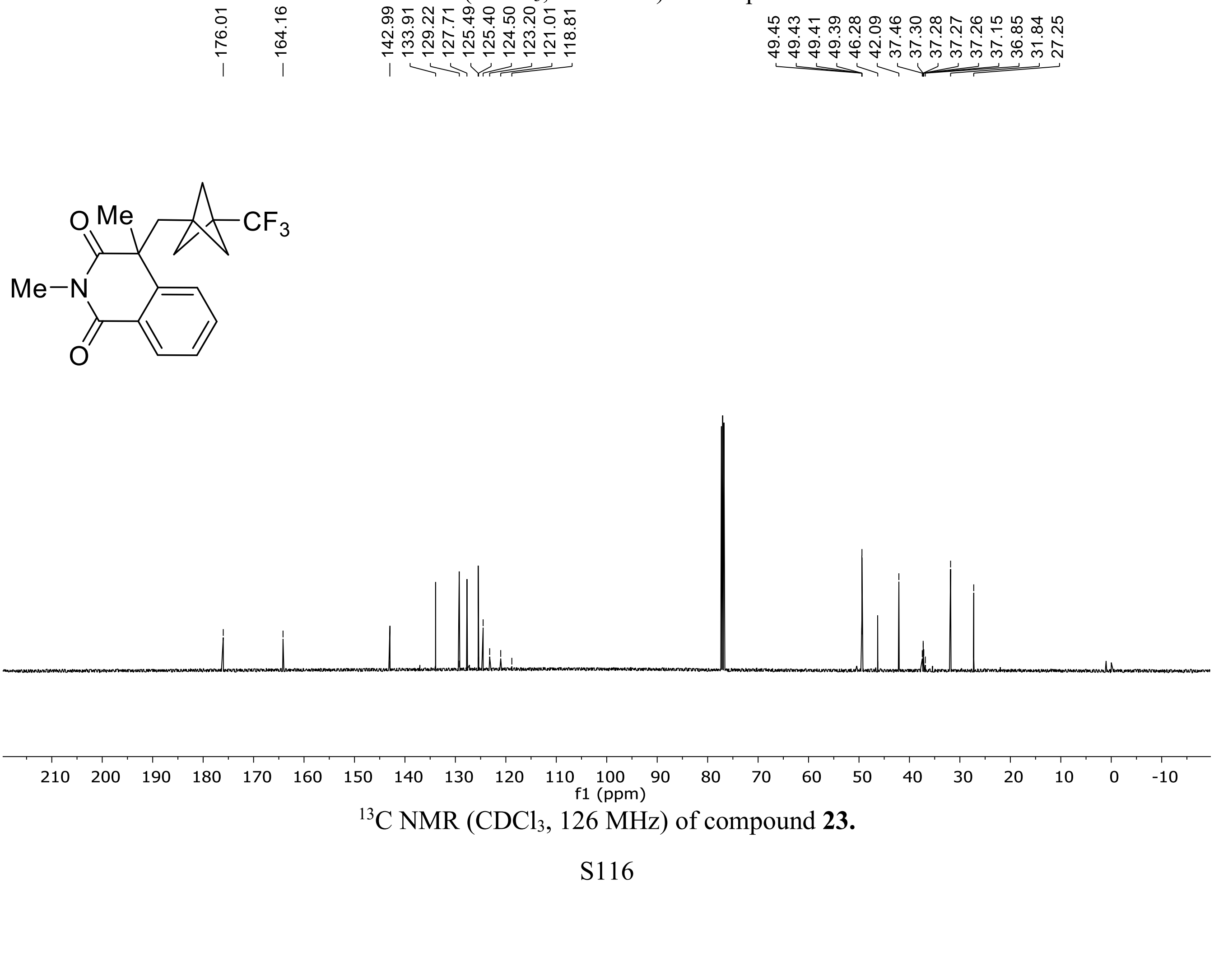

^{13}C NMR ($CDCl_3$, 126 MHz) of compound **23.**

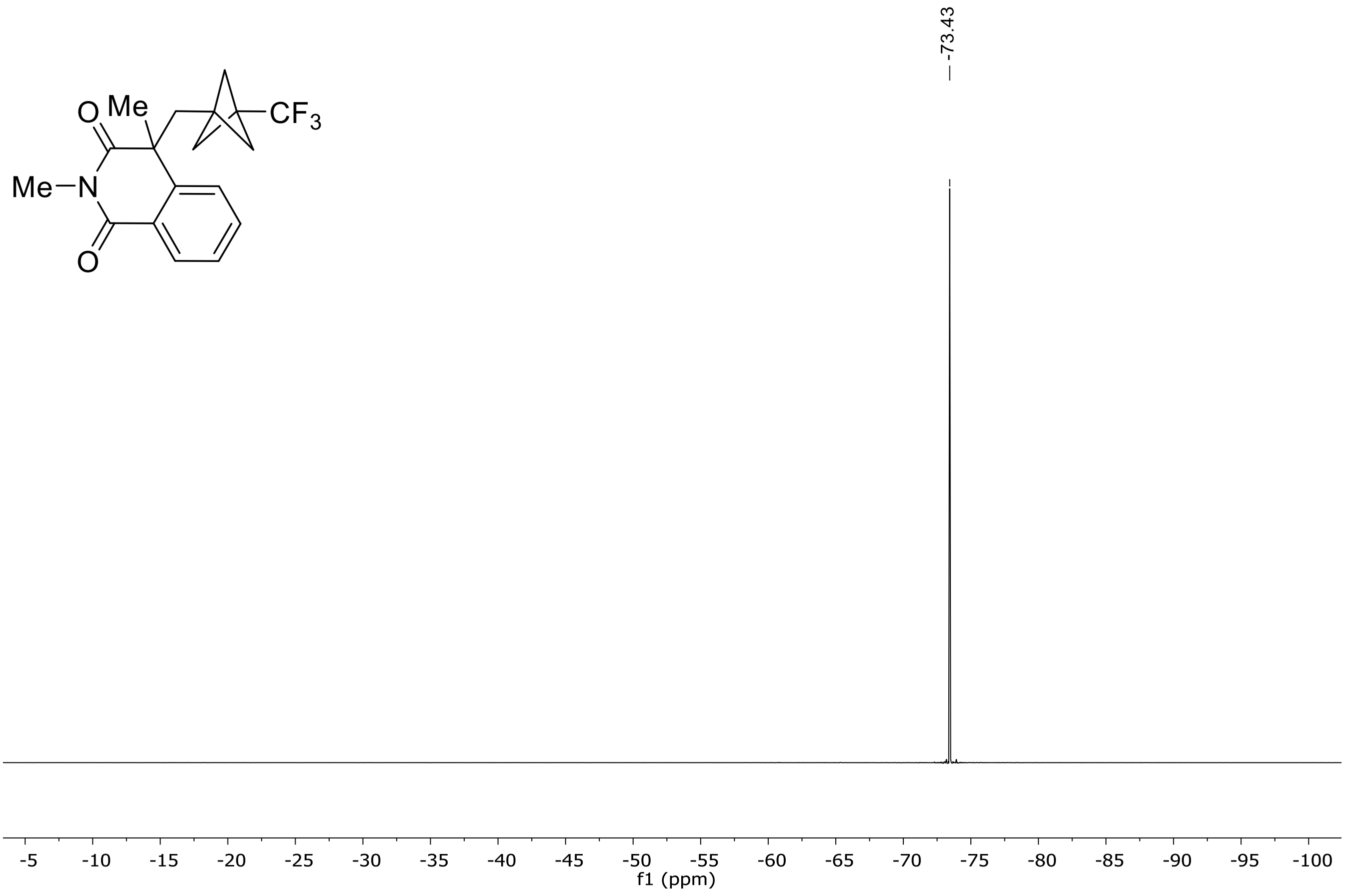

^{19}F NMR ($CDCl_3$, 377 MHz) of compound **23.**

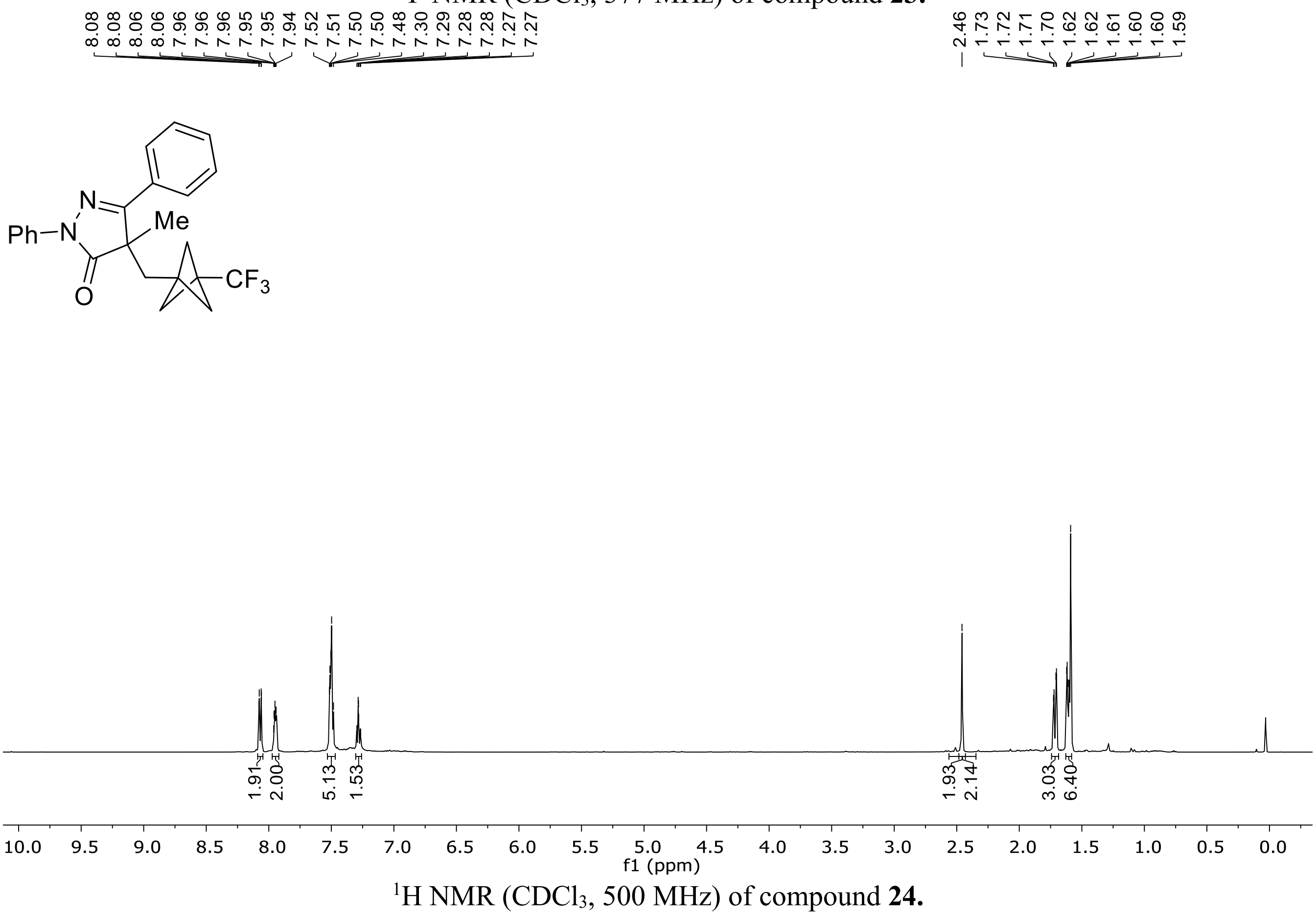

^{1}H NMR ($CDCl_3$, 500 MHz) of compound **24.**

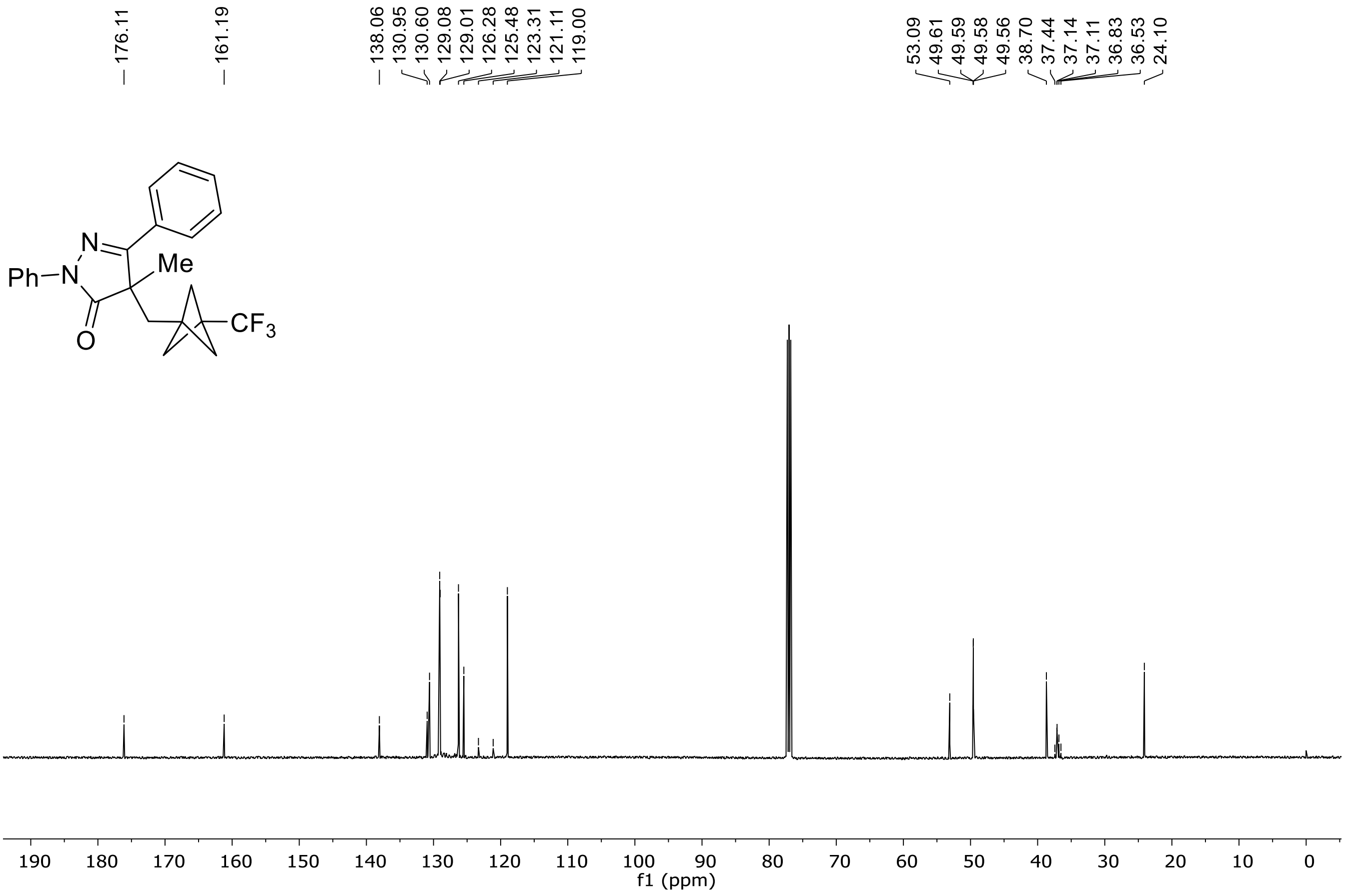

^{13}C NMR (CDCl$_3$, 126 MHz) of compound **24.**

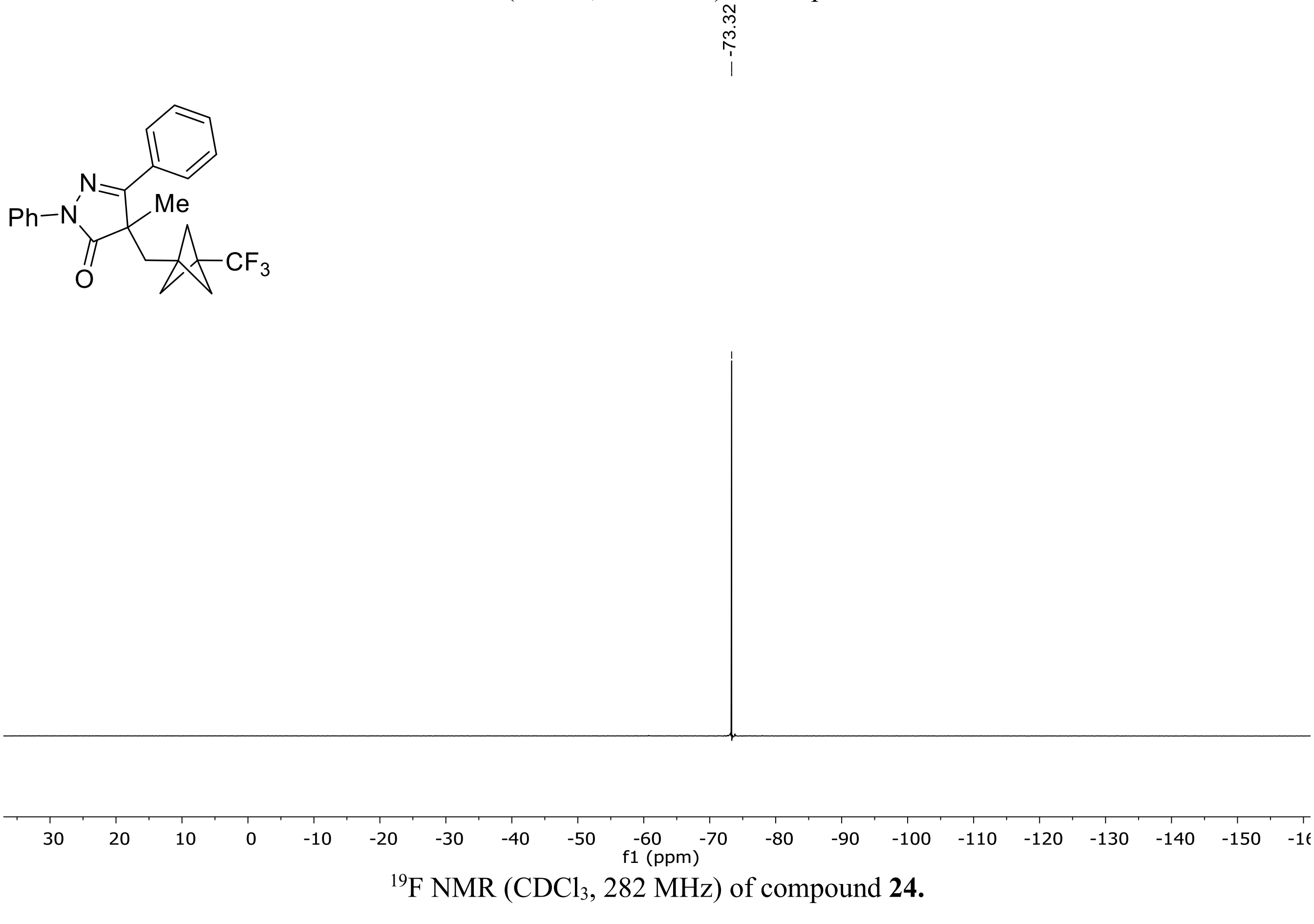

^{19}F NMR (CDCl$_3$, 282 MHz) of compound **24.**

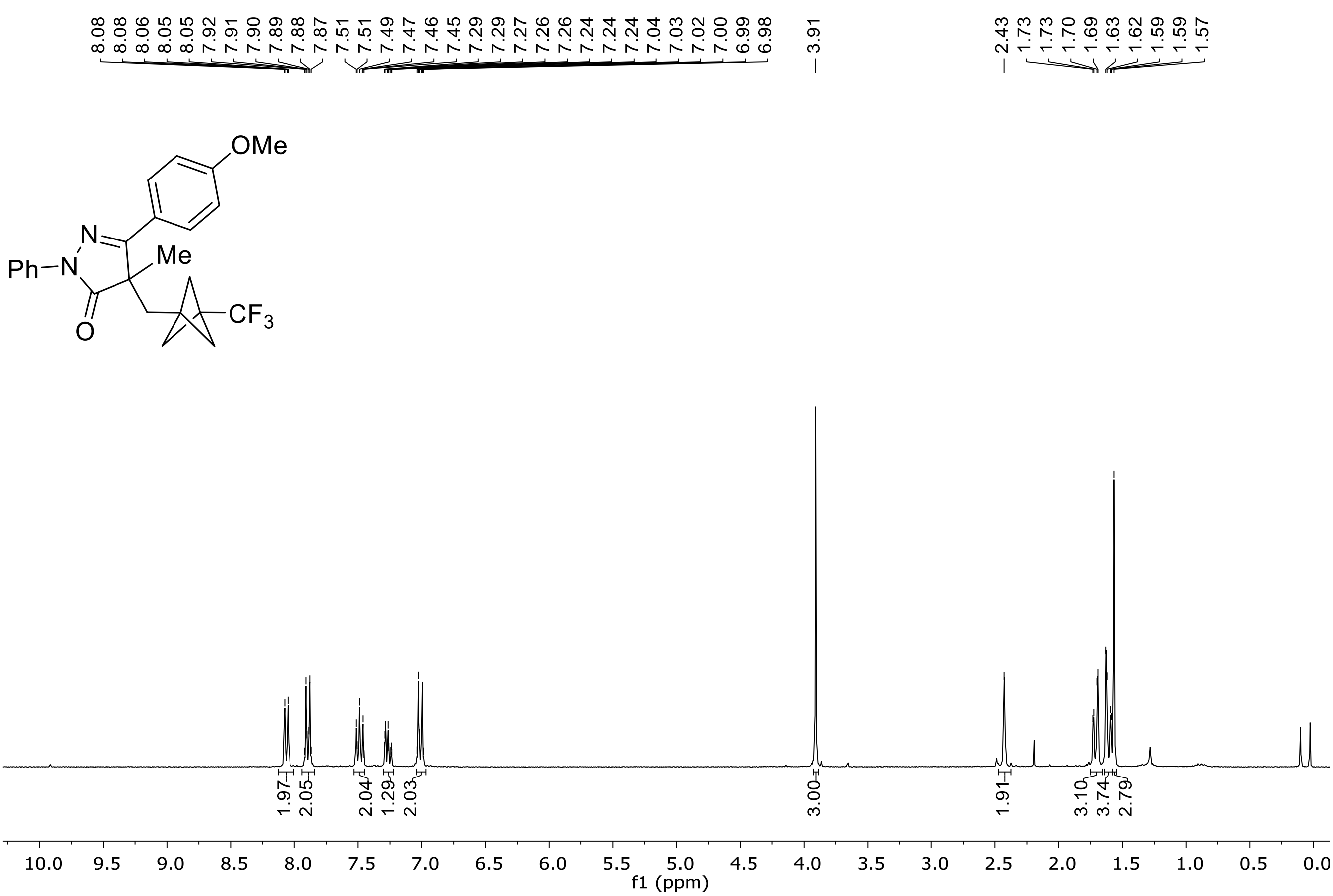

^{1}H NMR (CDCl$_3$, 300 MHz) of compound **25.**

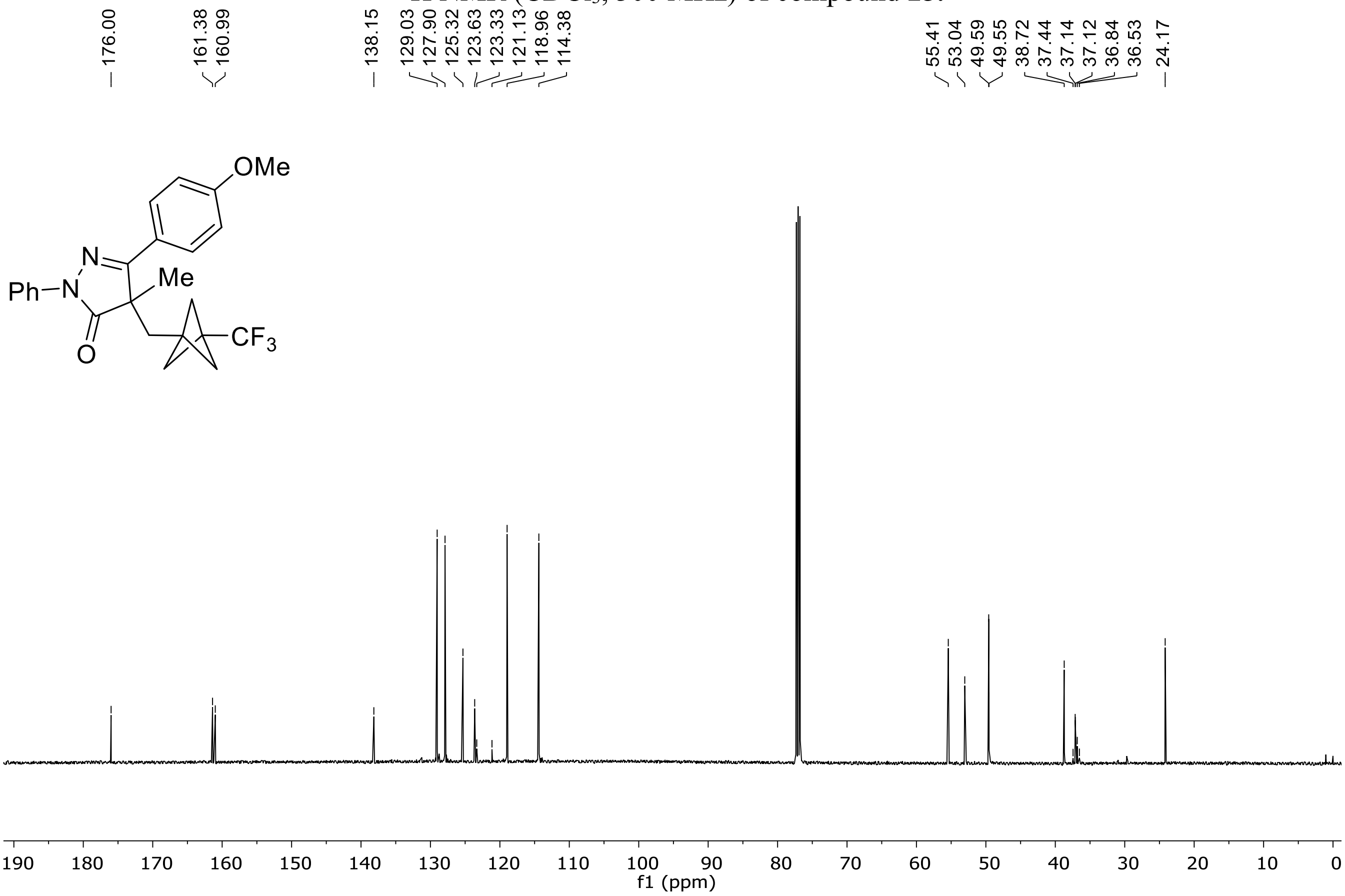

^{13}C NMR (CDCl$_3$, 126 MHz) of compound **25.**

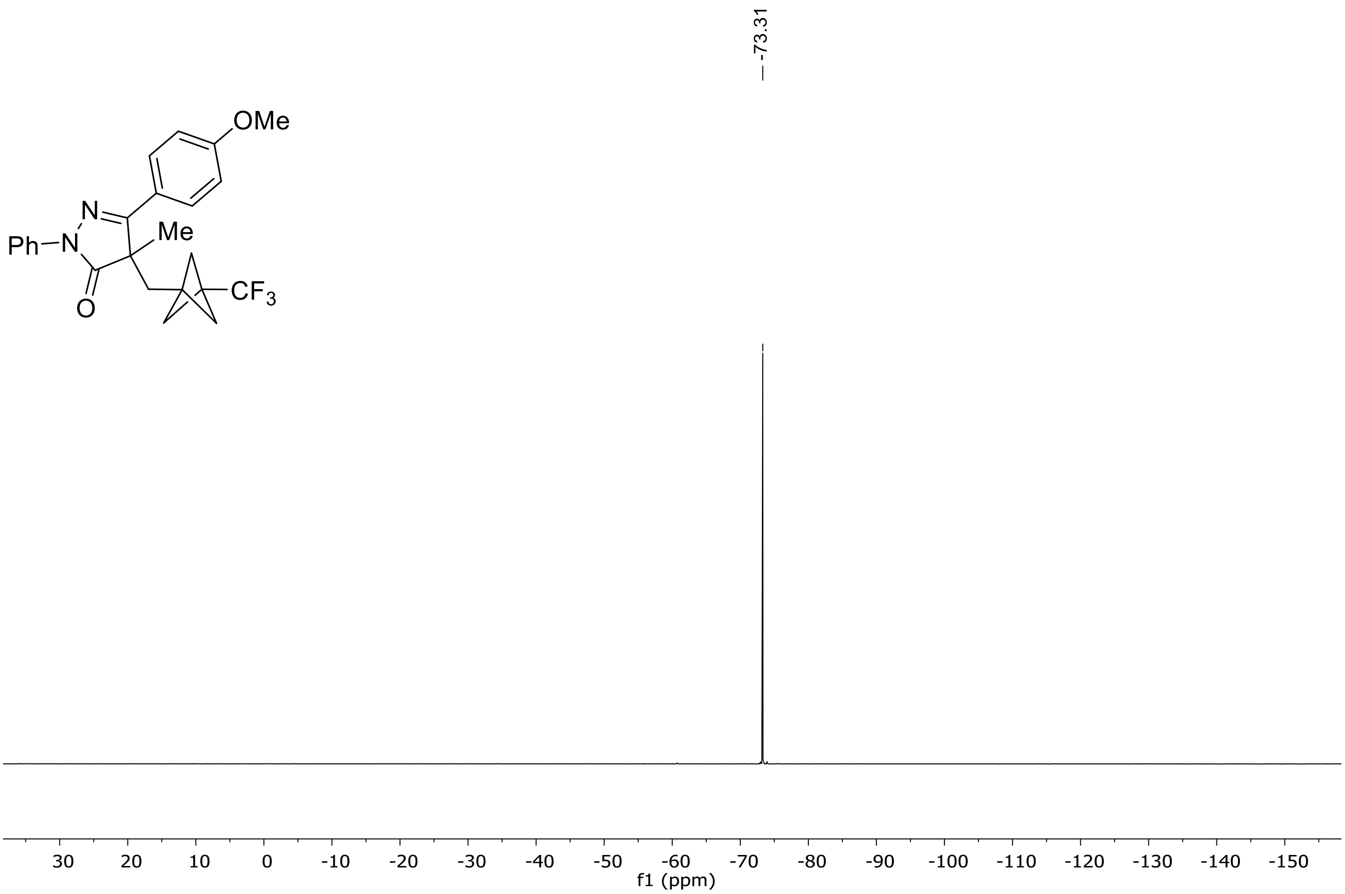

^{19}F NMR ($CDCl_3$, 282 MHz) of compound **25.**

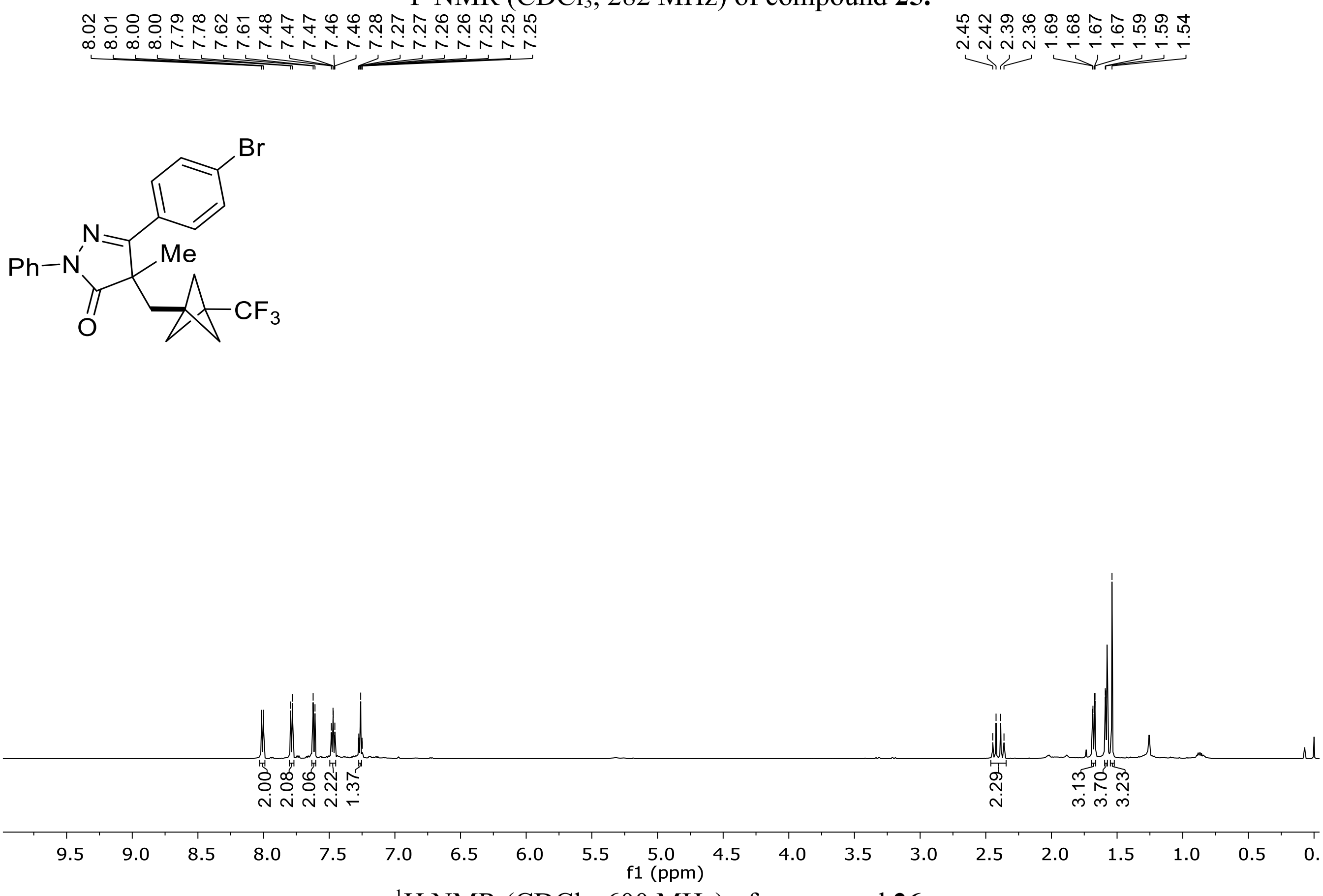

^{1}H NMR ($CDCl_3$, 600 MHz) of compound **26.**

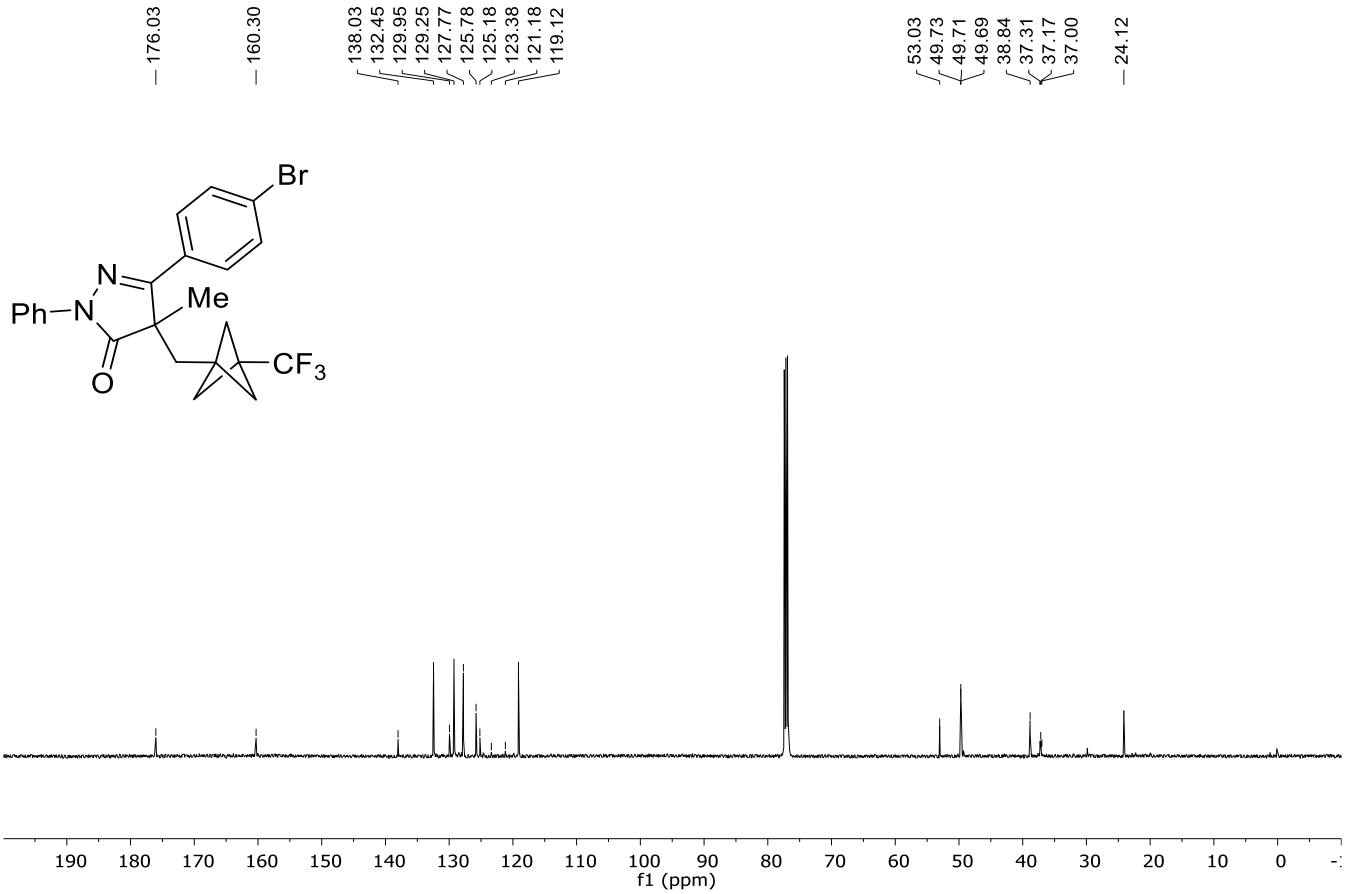

^{13}C NMR ($CDCl_3$, 126 MHz) of compound **26.**

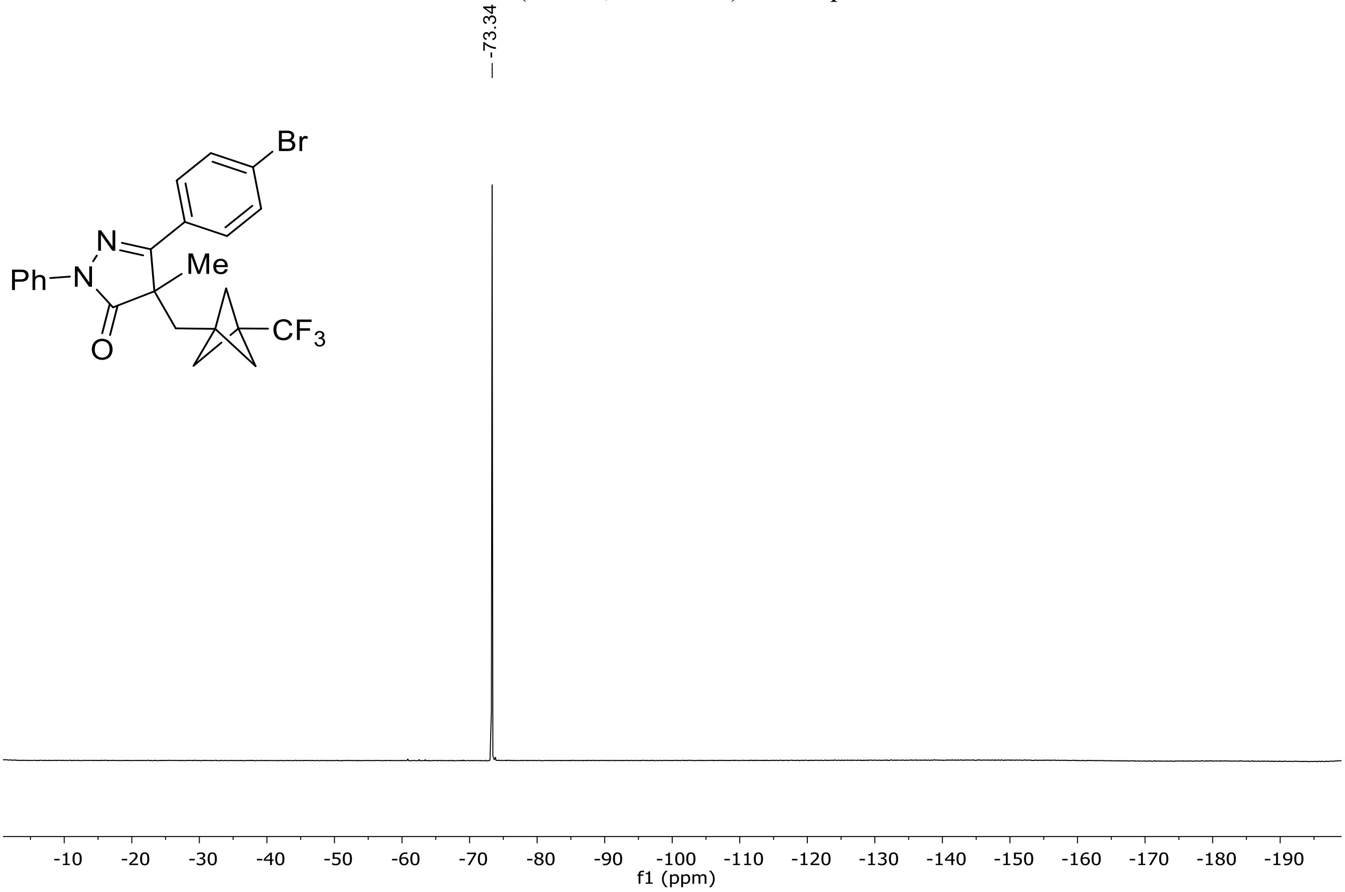

^{19}F NMR ($CDCl_3$, 377 MHz) of compound **26.**
